# Selective and Potent Proteomimetic Inhibitors of Intracellular Protein–Protein Interactions**

**DOI:** 10.1002/anie.201410810

**Published:** 2015-02-04

**Authors:** Anna Barnard, Kérya Long, Heather L Martin, Jennifer A Miles, Thomas A Edwards, Darren C Tomlinson, Andrew Macdonald, Andrew J Wilson

**Affiliations:** School of Chemistry, University of Leeds, Woodhouse LaneLeeds LS2 9JT (UK); Astbury Centre For Structural Molecular Biology, University of LeedsWoodhouse Lane, Leeds LS2 9JT (UK); Leeds Institute of Biomedical and Clinical SciencesWellcome Trust Brenner Building, University of Leeds, St. James's University Hospital, Leeds LS9 7TF (UK); School of Molecular and Cellular Biology, University of LeedsWoodhouse Lane, Leeds LS2 9JT (UK)

**Keywords:** apoptosis, foldamers, helical structures, peptidomimetics, protein–protein interactions

## Abstract

Inhibition of protein–protein interactions (PPIs) represents a major challenge in chemical biology and drug discovery. α-Helix mediated PPIs may be amenable to modulation using generic chemotypes, termed “proteomimetics”, which can be assembled in a modular manner to reproduce the vectoral presentation of key side chains found on a helical motif from one partner within the PPI. In this work, it is demonstrated that by using a library of N-alkylated aromatic oligoamide helix mimetics, potent helix mimetics which reproduce their biophysical binding selectivity in a cellular context can be identified.

Protein–protein interactions (PPIs) mediate all biological processes and thus are actively involved in the development and progression of disease.[1] Studies of the protein interactome have estimated that there may be as many as 650 000 pairwise interactions,[2] hence there is considerable therapeutic potential in being able to modulate these interactions. Despite this clear need, it has historically been considered challenging to identify small molecules which selectively recognize their protein targets based on the type of surface involved in PPIs.[3]–[5] Although, high-throughput screening (HTS),[6] fragment-based approaches,[7] and computer aided ligand ID/optimization[8] have afforded small-molecule modulators of PPIs, generic approaches which target particular classes of PPI are desirable. Helix-mediated PPIs[9] have received considerable attention[10] as the secondary structure motif represents a generic pharmacophore. Constrained peptides[11], [12] and ligands which mimic the helical topography of the helix (e.g. α/β and β-peptides)[13]–[15] are proven successful approaches and have entered clinical development.[16] An alternative small-molecule approach has been postulated whereby a generic scaffold is used to mimic the spatial and angular projection of “hot-spot” side chains found on the key helix mediating the PPI of interest.[17] Such ligands have been termed proteomimetics,[18] α-helix mimetics,[19]–[22] and topographical mimics.[23] Several studies on this general class of ligand have illustrated that they can be used to selectively recognize their target protein in biophysical assays,[19], [24], [25] that they act in cells upon the pathway in which the PPI is found,[23], [26], [27], [52] and that they exhibit the anticipated phenotypic effects in animals.[23] In this work we performed biophysical and cellular experiments on a library of N-alkylated aromatic oligoamide proteomimetics (Figure 1). Our purpose was to study the correlation between biophysical and cellular selectivity, and to highlight the potential for off-target effects, which have not been described for proteomimetics. Although strictly speaking our goal was not to identify inhibitors of a specific PPI, we identified potent inhibitors of p53/*h*DM2 and the B-cell lymphoma-2 (Bcl-2) family PPIs which induce apoptosis, and this may represent a novel avenue for anticancer therapeutics development.

**Figure 1:**
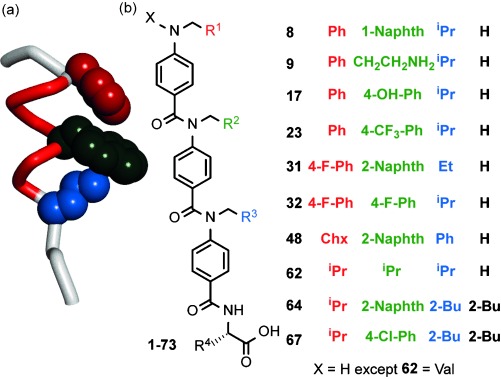
N-alkylated helix mimetics. a) The p53 helix illustrating key side chains. b) Structures of principle compounds discussed in this work.

The cellular levels of the transcription factor p53 are controlled by a negative feedback loop involving *h*DM2.[28] In normal cells, binding of the helical p53 N-terminal transactivation domain to a cleft on *h*DM2 results in its polyubiquitination and subsequent degradation.[29] In response to cellular stress p53 is activated and initiates apoptosis to eliminate the damaged cell. This target has seen the development of several small-molecule inhibitors as potential anticancer agents.[30] Similarly, the Bcl-2 family plays a central role in the regulation of apoptosis through control of mitochondrial outer membrane permeabilization.[31] Proteins within this family include the anti-apoptotic members (Bcl-2, Bcl-x_L_ and Mcl-1), pro-apoptotic members (BAK, BAX), and effector proteins (BID, BIM, PUMA and NOXA-B). The anti-apoptotic proteins contain a hydrophobic groove into which an α-helical BH3 domain of effector or pro-apoptotic proteins can bind. Although the exact mechanism by which these proteins coordinate to determine cell fate remains unclear,[32] in certain cancers, anti-apoptotic members are overexpressed and sequester the activity of the pro-apoptotic proteins, thus preventing apoptosis from taking place.

Building on our prior work[24], [33] on oligobenzamide foldamers,[34], [35] we synthesized a library of N-alkylated helix mimetics using a microwave-assisted solid-phase synthesis method which affords compounds in about 4 hours and in greater than 90 % purity suitable for screening (representative compounds shown in Figure 1; see Schemes S1 and S2 and Table S1 in the Supporting Information).[36], [37] In this instance, the library of 77 members was purified further by HPLC where appropriate. We initially selected p53/*h*DM2 as a model target. The library composition was tailored to reflect the key binding residues on the p53 helix, therefore members were furnished with mostly hydrophobic aliphatic and aromatic side chains to imitate Phe19, Trp23, and Leu26.[28] The 73 trimeric oligobenzamides were obtained alongside four dimers (trimers comprise three monomers linked by amides with dimers comprising two monomers; see the Supporting Information), which were designed to act as negative controls incapable of effective mimicry of the full p53 hot-spot region.

To test the behavior of aromatic oligoamides in cells, a high-content imaging screen was developed (Figure 2 a; see Figure S2 in the Supporting Information for additional images). Initially U2OS osteosarcoma cells were treated with 20 and 10 μm helix mimetics in low-serum media. The former concentration allowed the DMSO concentration to be kept at less than 0.2 % starting from 10 mm stocks whilst the second concentration was used to confirm statistical significance. Four endpoints were assessed 48 hours after addition of the mimetic with hits defined as described in the Supporting Information. Firstly, cell number was measured by nuclear counting. Secondly an antibody against caspase 3 (which is common to both the extrinsic and intrinsic apoptotic pathways),[38] was used to identify cells which may be apoptotic. The third endpoint assessed was autophagy, a self-digestion process that cells in stressed conditions undergo as part of their normal life cycle,[39] but which has been linked to apoptosis.[40] Although autophagy has also been implicated in cell survival, an apoptosis-inducing drug has been previously shown to increase autophagosome numbers in osteosarcoma cells,[41] whilst p53 has been shown to activate autophagy so as to increase the efficiency of apoptosis.[42] To probe for cells undergoing autophagy, cells were stained with the anti-LC3B antibody, which is known to bind to autophagosomal membranes.[43] Finally, the arrangement of actin filaments in cells treated with the helix mimetics was investigated using a fluorescently labelled phalloidin.[44] To promote cell motility and invasion, cancer cells alter the cytoskeleton and a number of reports have indicated a relationship between this modification and the activity of p53.[44] To provide a qualitative interpretation of the data, the data are shown in Figure 2 b as a heat map (a summary of all the data from the apoptosis assay for the full library of compounds can be found in Table S2 in the Supporting Information). A range of different activities were found across the library with some mimetics having no effect in any of the four categories, for example, **9**, **17**, **23, 32**, and **62**. Some compounds provided a hit with one or two of the markers, for example **8**, whilst a small number, including the compounds **64** and **67**, proved to be effective in three or four areas.

**Figure 2:**
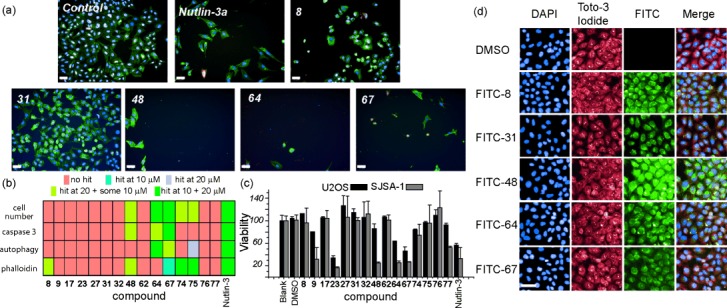
HCS summary for mimetic library. a) Example images of cells treated with five compounds (20 μm) and controls of 0.2 % DMSO and Nutlin-3a (5 μm) scale=50 μm. DAPI staining is shown in the blue channel, caspase 3 is shown in red, LC3B antibody in yellow, F-actin stained with AlexaFluor488 conjugated phalloidin shown in green. b) Heat map illustrating results of HCS. c) Summary of cellular toxicity of the mimetics added to U2OS (black) and SJSA-1 (grey) cells at a concentration of 50 μm. d) Cells were treated with FITC-labelled trimers and imaged using high-content imaging and co-stained with Toto-3 iodide as a cytoplasmic stain. Scale=50 μm.

An MTT cell toxicity assay using U2OS and SJSA-1 osteosarcoma cells was also used to identify compounds leading to an increase in cell death. The SJSA-1 cell line has been frequently used in studies on p53/*h*DM2 inhibitors because it overexpresses *h*DM2.[45] Cells were treated with a compound and incubated for 18 hours, after which time cell viability was measured using a Cell Titre 96® assay (Promega; Figure 2 c). There is generally good agreement between the two cell lines for the entire library (see Figure S3 in the Supporting Information). A number of compounds, including **23**, **64**, and **67**, show a similar level of toxicity to that of Nutlin-3. The MTT result for **23** is inconsistent with the HCS, however MTT reports on mitochondrial activity (biomass) rather than cell number. The reduced viability observed with **23** may therefore arise through its interaction with targets other than *h*DM2. Several compounds with little response in the HCS demonstrated little toxicity, for example, the compounds **9**, **17**, **32**, and **62**. The dimers **74**–**77** had little effect on cell population and fits well with the notion that these compounds act as poor p53 helix mimetics.

To confirm that the observed effects were due to the mimetics entering the cell, we labelled five mimetics with a fluorescein tag using previously reported click chemistry (see the Supporting Information for syntheses).[36] U2OS cells were treated with FITC-labelled trimers (Figure 2 d), and co-stained with DAPI and Toto-3 iodide. The modified mimetics co-localize with the cytoplasmic stain. An alternate method using biotinylated trimers confirmed this result and is described in the Supporting Information (Figure S4). Both sets of compounds retained *h*DM2 affinity, although they exhibited diminished activity in the HCS (see Figures S22 and S23 and Table S2a–d in the Supporting Information).

To test for inhibition of the p53/*h*DM2 interaction, selected helix mimetics were screened in a fluorescence anisotropy (FA) competition assay. Six of the proteomimetics [**8**, **23** (see Figure S5 in the Supporting Information), **31**, **48**, **64**, and **67**] displace p53 from *h*DM2 with low μm IC_50_ values (Figure 3 a). Despite containing a range of different side chains, all six mimetics possess a large aromatic group on the central residue, which is consistent with effective mimicry of the central tryptophan residue on the p53 helix. Four compounds (**9**, **17**, **32**, and **62**) showed no inhibition of the p53/*h*DM2 interaction up to a concentration of 100 μm (Figure 3 b). These compounds have either a small or polar side chain in the central position which is likely to abrogate inhibition. The dimers were generally poor inhibitors of the p53/*h*DM2 interaction (see Figure S6 in the Supporting Information). The compound **64** was tested for its ability to bind to ^15^N-labelled *h*DM2 (Figure 3 c). ^1^H-^15^N HSQC spectra were recorded in the absence and presence of compound (see Figures S7 and S8 in the Supporting Information). Upon addition of **64**, crosspeaks in the ^1^H-^15^N-HSQC spectrum shifted and decreased in volume, thus indicating a direct interaction with *h*DM2. The chemical shifts were mapped onto the structure of *h*DM2 using a published NMR assignment.[46] The compound **64** binds to *h*DM2, shown by significant movement of the crosspeaks for Phe55 and His73, at either side of the p53 binding site. These shifts are similar in nature to those observed upon titration of the p53 peptide against ^15^N-labelled *h*DM2,[33] thus further suggesting that the compounds act as effective p53 mimetics. Similar results were obtained for **67** (see Figures S9–S11 and Supporting Information).

**Figure 3:**
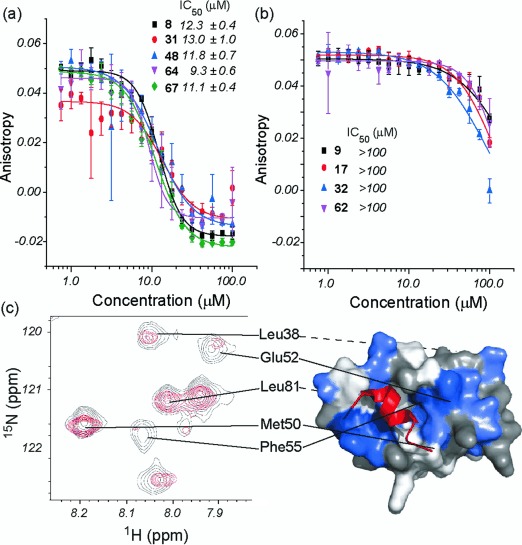
Biophysical analyses of helix mimetics. Dose-response curves for the inhibition of the p53/*h*DM2 interaction measured by fluorescence anisotropy for a) 8, 31, 48, 64, and 67 and b) 9, 17, 32, and 62. c) ^1^H-^15^N HSQC spectra of ^15^N-labelled *h*DM2. Spectra was recorded in the absence (in black) and the presence (in red) of 64 (crosspeaks that move or change in volume are mapped onto the surface of *h*DM2 and shown in blue).

Collectively, the HCS, MTT, and FA experiments identified library members most likely to act as effective mimics of p53 function. Five promising trimers were taken forward for further investigation along with four negative controls identified above. These five potent compounds were subjected to the HCS in U2OS and SJSA-1 cells at a range of different concentrations to determine dose-response curves for the mimetics and establish an active concentration (Figure 4 a and see Figure S12a in the Supporting Information). The mimetics **64** and **67** proved to be the most effective, presenting IC_50_ values of around 4 μm in both cell lines (Nutlin-3 gives a value of 1.4 μm; see Figure S12b, the data of which compares well with the literature[47] and a recently described compound which has entered clinical development).[47] We then sought to directly determine whether the helix mimetics were able to act on the p53/*h*DM2 interaction in cells. Western-blot analysis was carried out on both U2OS and SJSA-1 cells. In both cases the activity of p53 as a transcription factor was demonstrated by increased expression of its downstream target p21 (Figure 4 b; see Figure S13 in the Supporting Information). Trimers shown to inhibit the interaction in the FA assay and promote cell death through an apoptotic mechanism induce p21 expression, whereas dimers were ineffective (see Figure S14 in the Supporting Information). Biotinylated trimers were then used in a streptavidin pull-down experiment. In both U2OS and SJSA-1 cells, the mimetics with good p53/*h*DM2 potency pull-down *h*DM2 (Figure 4 c; full uncropped gel images see Figure S15 in the Supporting Information). To assess the selectivity of the helix mimetics, we performed an additional screen in Saos-2 cells which are p53-null; compounds selective for p53/*h*DM2 should be inactive in this cell line. The mimetics were found to have activity in the Saos-2 cell line (Figure 4 d) comparable to that observed in the U2OS and SJSA-1 cell lines. This activity indicated that these compounds may act on additional targets in the apoptosis pathway.

**Figure 4:**
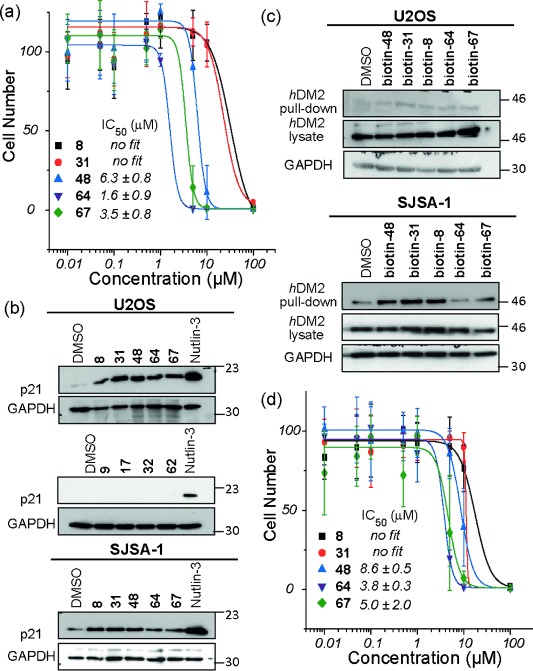
Cellular response to mimetics. a) Dose-response curves based on analysis of cell number by nuclear staining from U2OS cells treated with different concentrations of mimetics. b) U2OS and SJSA-1 cell lines were incubated with mimetics (50 μm) or Nutlin-3 (10 μm) for 4 h and lysates analyzed by western blotting for p21 and GAPDH. c) U2OS and SJSA-1 cells were treated with biotinylated mimetics (10 μm) for 4 h and cell lysates were subjected to Streptavidin pull-down followed by analysis by western blotting for *h*DM2. d) Same as for (a) except for the use of Saos-2 cells.

Expression of p53 in Saos-2 cells has been shown to result in potent induction of apoptosis, independent of its function as a transcription factor,[48], [49] whilst several studies have shown p53 is capable of binding to Bcl-2 family members.[50] The subfamily of trimers was therefore tested in FA competition assays against Bcl-2 family interactions: Mcl-1/NOXA-B and Bcl-x_L_/BAK (Figure 5 a,b). The compounds **64** and **67** showed micromolar inhibition of the Mcl-1/NOXA-B interaction, whereas **8**, **31**, and **48** were weak inhibitors. Significantly, none of the mimetics were able to disrupt the Bcl-x_L_/BAK interaction despite the similarity in the BH3 binding clefts of Mcl-1 and Bcl-x_L_. The compound **64** (Figure 5 c) was tested for binding to ^15^N-labelled Mcl-1. ^1^H-^15^N HSQC spectra were recorded in the absence and presence of compound (see Figure S16 and S17 in the Supporting Information). Upon addition of **64**, crosspeaks in the ^1^H-^15^N-HSQC spectrum both shifted and decreased in volume, thus indicating direct interaction between the compound and Mcl-1. These chemical shifts were mapped onto the structure of Mcl-1 using a recently published NMR assignment.[51] The analysis suggests **64** binds in the BH3 binding cleft of Mcl-1, as shown by significant movement of the crosspeaks for Phe270 and Arg263 along peptide binding site. Similar results were obtained for **67** (see Figure S18–20 in the Supporting Information). Finally we probed the ability of the trimers to bind Mcl-1 and Bcl-x_L_ in cells using the biotintylated trimers and streptavidin pull-down on cell lysate from U2OS and Saos-2 cell lines. In all cases biotinylated trimers were capable of pulling down Mcl-1 from cell lysate (the pull-down bands for biotin/**64** and biotin/**67** were both more intense than the other compounds reflecting their greater potency). We observe no evidence of Bcl-x_L_ pull-down, thus indicating that the selectivity observed in the biophysical assay is replicated in the cellular environment. Full, un-cropped gel images are available in the Supporting Information (Figure S21)

**Figure 5:**
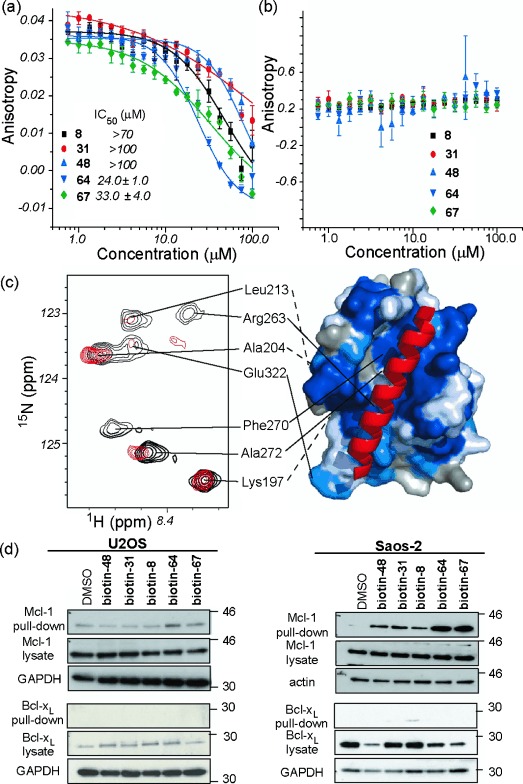
Bcl-2 family binding properties of mimetics. Dose-response curves of the inhibition of the a) Mcl-1/NOXA-B and b) Bcl-xL/BAK interactions measured with fluorescence anisotropy. c) ^1^H-^15^N HSQC spectra of ^15^N-labelled Mcl-1. Spectra recorded in the absence (in black) and presence (in red) of 64. Crosspeaks that move or change in volume are mapped onto the surface of Mcl-1 and shown in blue. d) U2OS and Saos-2 cells were treated with of biotinylated mimetics (10 μm) for 4 h and cell lysates were subjected to Streptavidin pull-down followed by analysis by western blotting for Mcl-1 or Bcl-x_L_ (GAPDH or actin used as loading controls).

In conclusion, we have described the design, synthesis, and testing of a library of N-alkylated helix mimetics. Whilst some correlation between biophysical and cellular potency is evident, the results reveal the interplay between the two is complex, and emphasize the need to perform multiple analyses on libraries of helix mimetics in order to a) identify (and ultimately select for) desirable structure function behavior and b) to understand strengths and areas for further development (e.g. off target effects) of α-helix mimetics as PPI inhibitors. For instance, **31** is a reasonable inhibitor of the p53/*h*DM2 interaction in the FA screen, but does not score highly for induction of apoptosis or cell viability. Similarly, **23** reduced cell viability and acts as an inhibitor of p53/*h*DM2 in the FA experiment but only reduced cell number in the HCS to a limited extent. Most significantly, the proteomimetics **64** and **67** were active in all three experiments, and shown to bind endogenous *h*DM2 and elicit the downstream effects of p53 function. Moreover **64** and **67** were shown to act in a potent and selective manner on Mcl-1/NOXA-B over Bcl-x_L_/BH3 in both biophysical assays and a cellular context—a selectivity profile which is challenging to achieve. Thus the results establish that these helix mimetics can reproduce their binding selectivity in cells, whilst dual inhibition of *h*DM2 and Mcl-1 may also represent a novel approach for elaboration of anticancer chemotherapeutics.
