# Supplementary material for: Selective and Potent Proteomimetic Inhibitors of Intracellular Protein–Protein Interactions
Source: Angew Chem Int Ed Engl. 2015 Feb 4;54(10):2960–5. doi: 10.1002/anie.201410810 (PMC4506525; doi:10.1002/anie.201410810)
Supplement: Supplementary file 1 [file anie0054-2960-sd1.pdf]

Angewandte Chemie
Eine Zeitschrift der Gesellschaft Deutscher Chemiker

Supporting Information

© Wiley-VCH 2015

69451 Weinheim, Germany

Selective and Potent Proteomimetic Inhibitors of Intracellular Protein–Protein Interactions**

Anna Barnard, Kérya Long, Heather L. Martin, Jennifer A. Miles, Thomas A. Edwards, Darren C. Tomlinson, Andrew Macdonald, and Andrew J. Wilson**

anie_201410810_sm_miscellaneous_information.pdf

Contents

General Procedures 2
Synthetic Chemistry 2
Cellular and Biophysical Analysis 9
Detailed Synthetic Procedures and Characterization 40

General Considerations

All chemicals and solvents were purchased and used without further purification. ^{1}H, ^{13}C and 2D NMR Spectra were recorded with a Bruker DRX 500 MHz or DPX 300 MHz spectrometer. ^{1}H NMR spectra are referenced to tetramethylsilane (TMS) and chemical shifts are given as parts per million downfield from TMS. Coupling constants are reported to the nearest 0.1 Hz. Melting points were determined using a Griffin D5 variable temperature apparatus and are uncorrected. Microanalyses were obtained on a Carlo Erba Elemental Analyser MOD 1106 instrument. IR spectra were recorded with a Perkin–Elmer FTIR spectrometer and samples were analysed in the solid phase. Mass spectra (HRMS) were obtained with a Bruker maxis impact 3000 spectrometer using electrospray ionisation. LC-MS experiments were run on a Waters Micromass ZQ spectrometer. Analytical HPLC analysis was carried out on an Agilent Technologies 1260 Infinity on a gradient of 95-5% acetonitrile in water. Analytical TLC was performed on 0.2 mm silica gel 60 F254 precoated aluminium sheets (Merck) and visualised by using UV irradiation or, in the case of amine intermediates, by staining with ninhydrin solution. Flash chromatography was carried out on silica gel 60 (35–70 micron particles, FluoroChem). The convention used to assign the spectroscopic data and for naming compounds for this series of aromatic oligoamides has been described previously.[1] Additional side chain functionalities were assigned in the NMR as 2-3° denoting the central aromatic ring functionalised at the 3-position on an oxygen atom.

Synthetic Chemistry

Helix mimetics and associated monomer building blocks were synthesized following previously reported methodology according to Scheme S1 and is described in the following section whilst characterization is described from pg 40 and ^{1}H NMR spectra and LC-MS analyses are provided from pg 71. Helix mimetics used in biophysical and cell based screens described in this work are given in table S1. Helix mimetics labelled with biotin or fluorescein are annotated as such and given the parent compounds number from which they were derived.

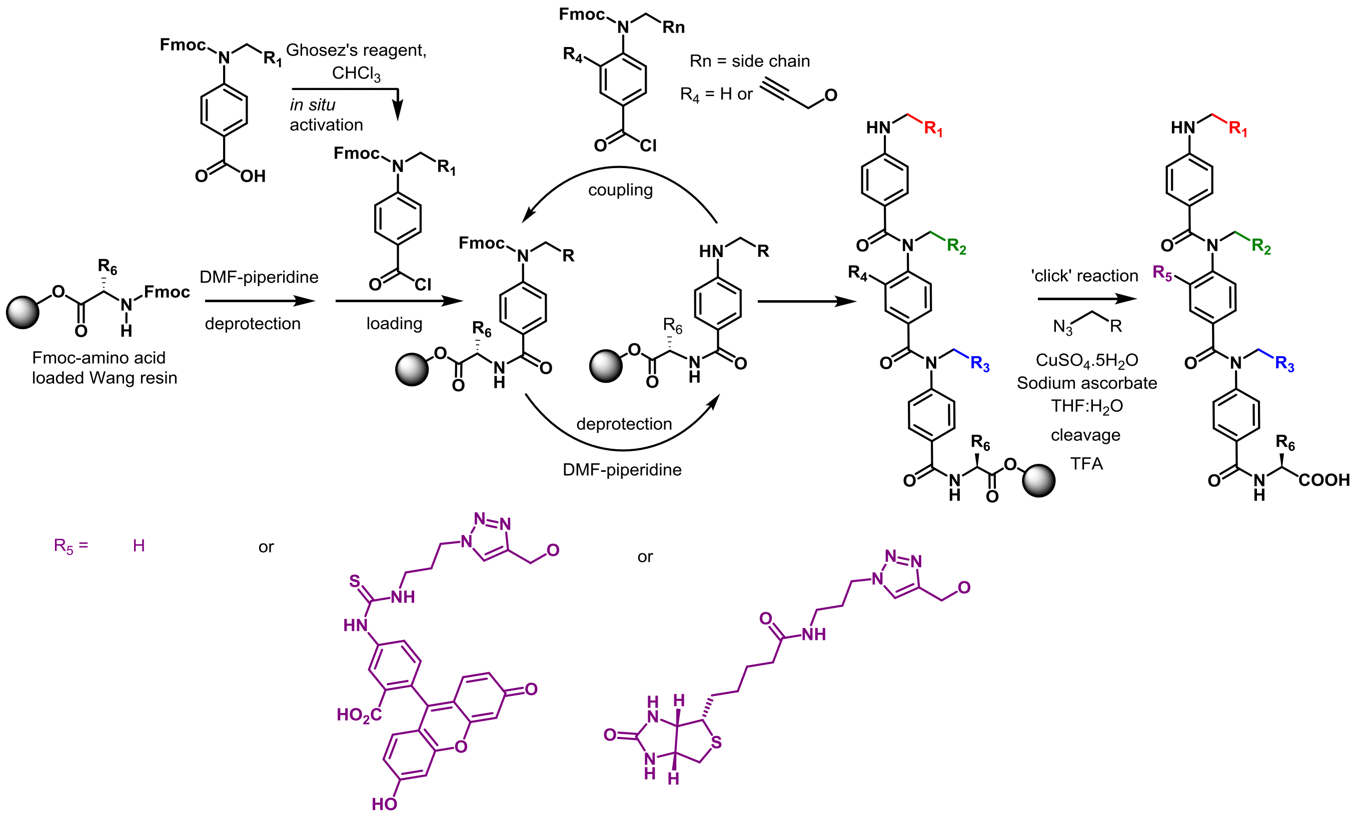

Scheme S1 Synthesis of helix mimetics on solid-phase.

To label mimetics with a biotin probe, a synthesis of an appropriate tag was developed as illustrated in Scheme S2.

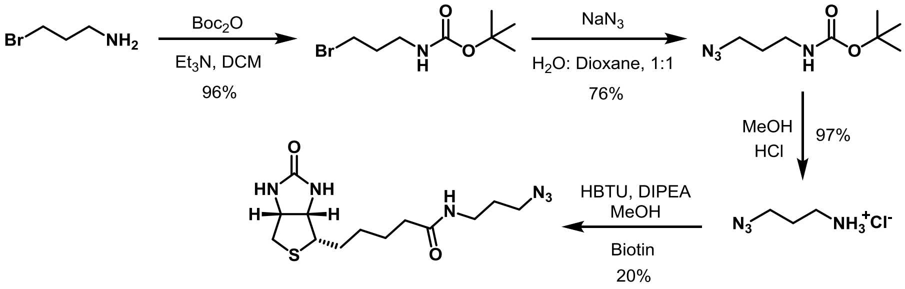

Scheme S2 Synthesis of a novel biotin azide for use in solid phase 'click' chemistry.

It was also necessary to obtain three additional alkyne tagged monomers (Fig. S1). These were synthesized using established methods.

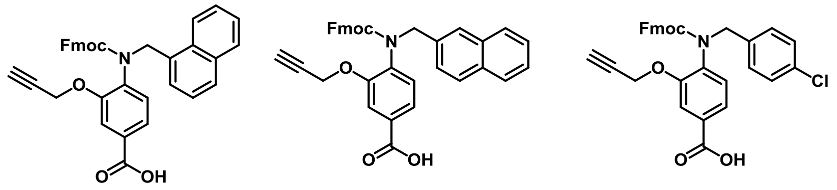

u **v** **w**

Figure S1 – Structures of the alkyne-functionalised monomers used in 'click' chemistry reactions.

Solid-Phase Trimer Synthesis

Amino acid-loaded Wang resin (0.1 mmol) was swelled in anhydrous DMF (5 ml) 15 minutes prior to reaction. The monomers (0.5 mmol) were each dissolved in anhydrous $CHCl_3$ (10 ml) and pre-activated for coupling with Ghosez's reagent (315 µl, 20% in $CHCl_3$, 0.48 mmol) for 1 hour at room temperature. The coupling reactions were carried out on a CEM Liberty microwave assisted automated peptide synthesiser. A small sample was removed and cleaved from the resin with TFA: CH_2Cl_2 (1:1, 1 ml) and analysed by LC-MS to confirm formation of the desired trimer; coupling reactions were assumed to have gone to completion.

'Click' Chemistry

The trimers on amino acid-loaded Wang resin were suspended in THF: H_2O (1:1, 1 ml) and the azide was added (1 equivalent) along with $CuSO_4.5H_2O$ (10 mol%) and sodium ascorbate (20 mol%). The reaction mixture was stirred overnight at room temperature. The resin was then washed with H_2O (1 ml, 5 mins) and subjected to the cleavage protocol.

Cleavage

The cleavage step was carried out manually, in 1.5 ml 'Extract-Clean' polypropylene reservoirs fitted with 20 µm polyethylene frits (Alltech). The resin was transferred to the reservoir and washed with CH_2Cl_2 (1 ml, 5 mins) and diethyl ether (1 ml, 5 mins). A 1:1 mixture of TFA: CH_2Cl_2 was added and the mixture was stirred for 30 mins at room temperature and the contents collected and the procedure repeated. The resulting solution was concentrated affording the target compound.

Table S1 – Structures of the *N*-alkylated helix mimetics synthesised for screening.

Compound No.	N terminus (R_1)	Middle residue (R_2)	C terminus (R_3)	Amino Acid (R_4)
**1**	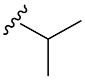 **a**	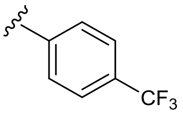 **b**	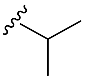 **a**	Gly
**2**	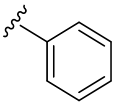 **c**	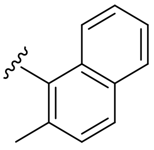 **d**	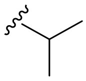 **a**	Gly
**3**	**e** 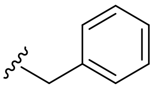	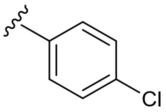 **f**	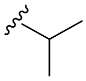 **a**	Gly
**4**	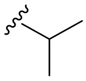 **a**	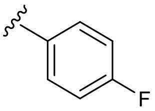 **g**	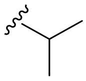 **a**	Gly
**5**	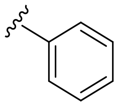 **c**	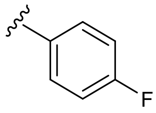 **g**	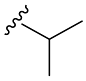 **a**	Gly

Compound No.	N terminus (R_1)	Middle residue (R_2)	C terminus (R_3)	Amino Acid (R_4)
**6**	**c** 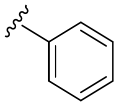	**h** 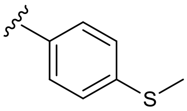	**a** 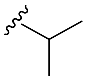	Gly
**7**	**c** 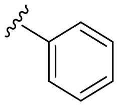	**i** 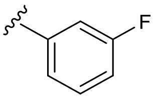	**a** 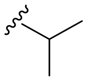	Gly
**8**	**c** 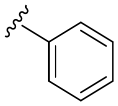	**j** 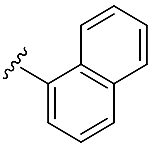	**a** 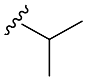	Gly
**9**	**c** 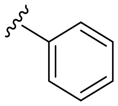	**k** 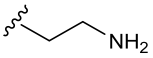	**a** 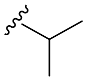	Gly
**10**	**c** 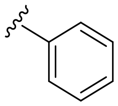	**l** 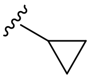	**a** 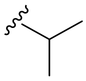	Gly
**11**	**c** 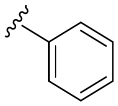	**g** 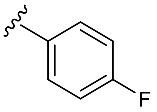	**m** 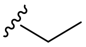	Gly
**12**	**i** 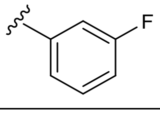	**n** 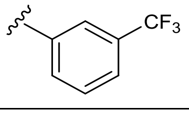	**a** 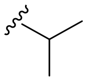	Gly
**13**	**f** 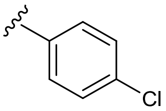	**n** 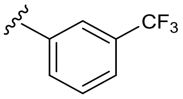	**a** 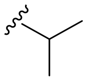	Gly
**14**	**g** 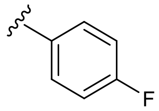	**n** 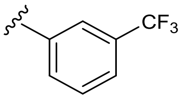	**a** 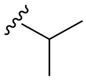	Gly
**15**	**c** 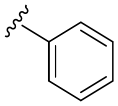	**o** 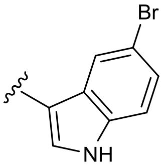	**a** 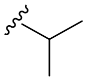	Gly
**16**	**c** 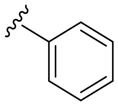	**p** 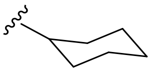	**a** 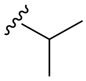	Gly
**17**	**c** 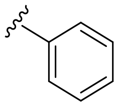	**q** 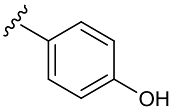	**a** 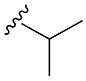	Gly
**18**	**c** 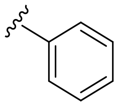	**e** 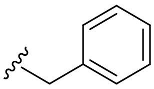	**m** 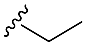	Gly
**19**	**c** 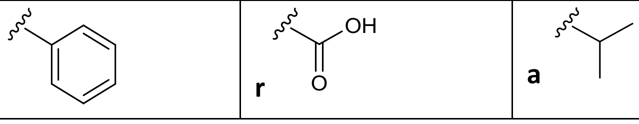	**r**	**a**	Gly

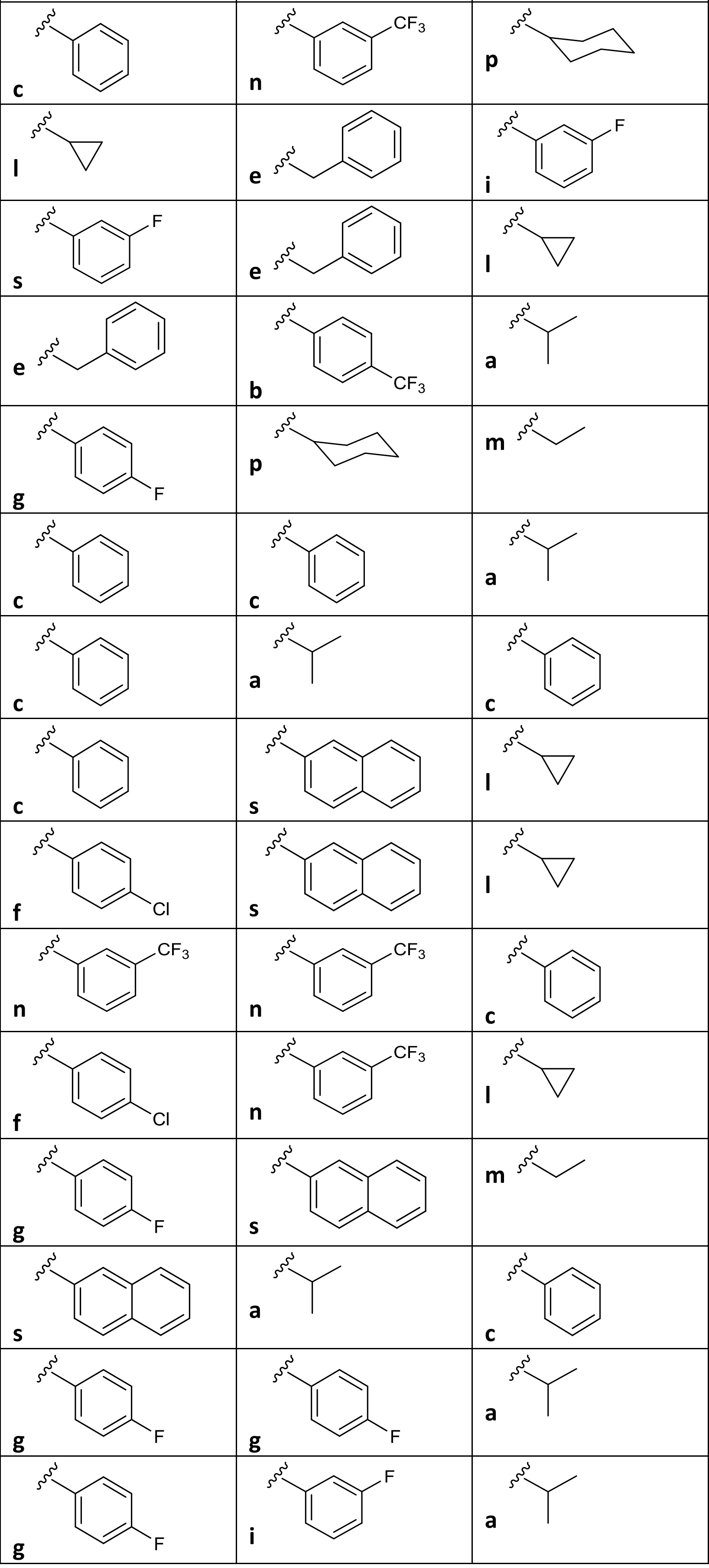

Compound No.	N terminus (R_1)	Middle residue (R_2)	C terminus (R_3)	Amino Acid (R_4)
**20**	**c**	**n**	**p**	Gly
**21**	**l**	**e**	**i**	Gly
**22**	**s**	**e**	**l**	Gly
**23**	**e**	**b**	**a**	Gly
**24**	**g**	**p**	**m**	Gly
**25**	**c**	**c**	**a**	Gly
**26**	**c**	**a**	**c**	Gly
**27**	**c**	**s**	**l**	Gly
**28**	**f**	**s**	**l**	Gly
**29**	**n**	**n**	**c**	Gly
**30**	**f**	**n**	**l**	Gly
**31**	**g**	**s**	**m**	Gly
**32**	**s**	**a**	**c**	Gly
**33**	**g**	**g**	**a**	Gly
**34**	**g**	**i**	**a**	Gly

Compound No.	N terminus (R_1)	Middle residue (R_2)	C terminus (R_3)	Amino Acid (R_4)
**35**	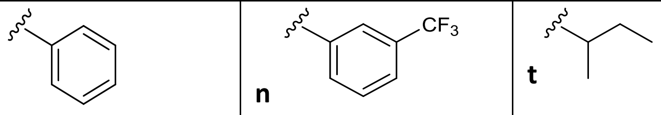 **c**	**n**	**t**	Gly
**36**	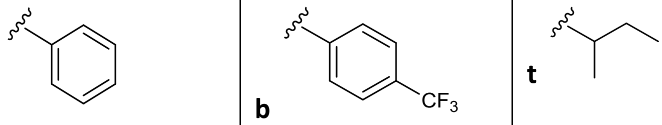 **c**	**b**	**t**	Gly
**37**	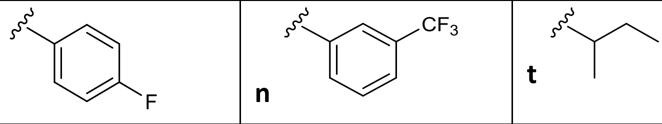 **g**	**n**	**t**	Gly
**38**	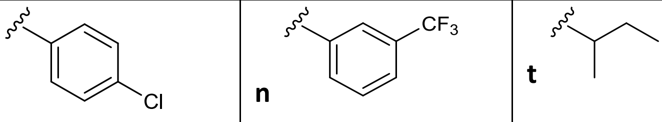 **f**	**n**	**t**	Gly
**39**	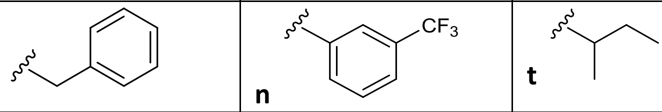 **e**	**n**	**t**	Gly
**40**	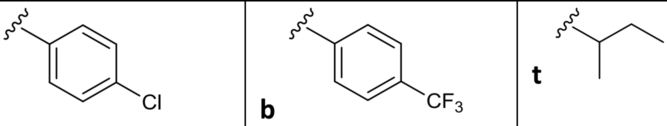 **f**	**b**	**t**	Gly
**41**	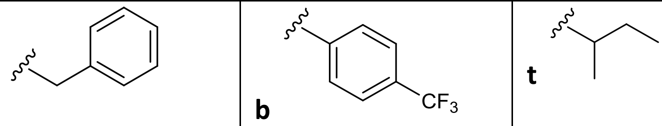 **e**	**b**	**t**	Gly
**42**	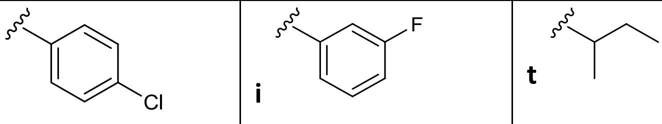 **f**	**i**	**t**	Gly
**43**	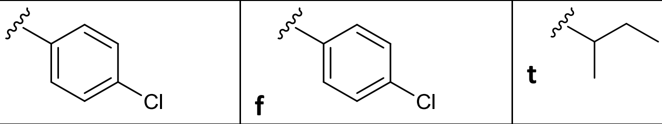 **f**	**f**	**t**	Gly
**44**	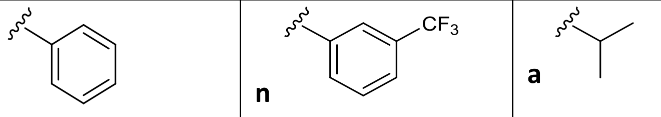 **c**	**n**	**a**	Gly
**45**	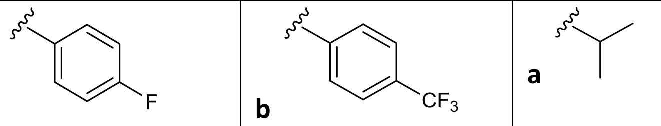 **g**	**b**	**a**	Gly
**46**	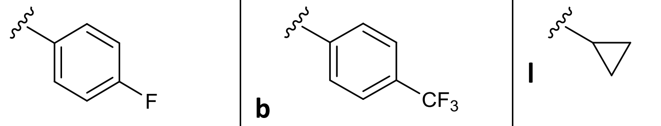 **g**	**b**	**l**	Gly
**47**	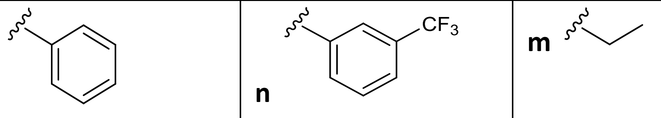 **c**	**n**	**m**	Gly
**48**	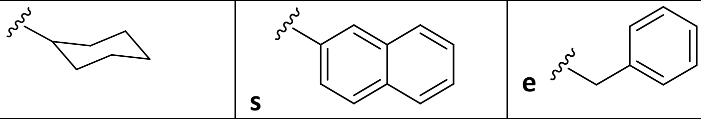 **p**	**s**	**e**	Gly
**49**	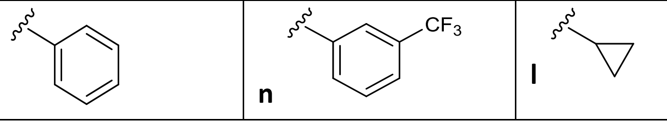 **c**	**n**	**l**	Gly

Compound No.	N terminus (R_1)	Middle residue (R_2)	C terminus (R_3)	Amino Acid (R_4)
**50**	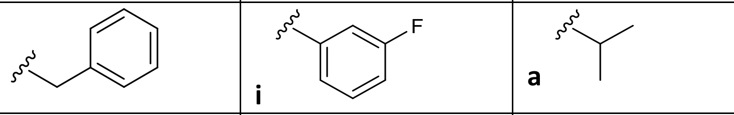 **e**	**i**	**a**	Gly
**51**	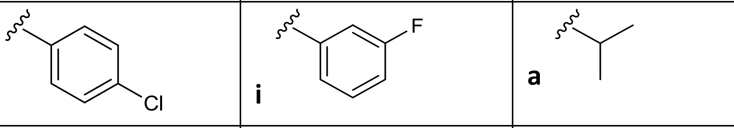 **f**	**i**	**a**	Gly
**52**	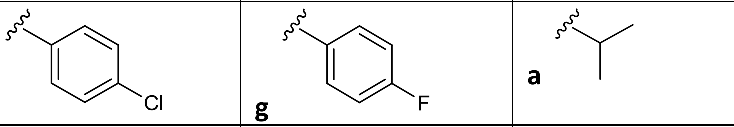 **f**	**g**	**a**	Gly
**53**	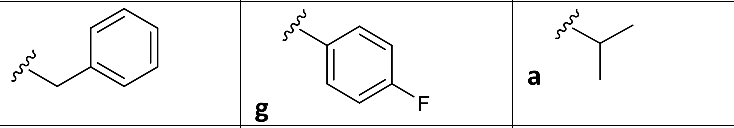 **e**	**g**	**a**	Gly
**54**	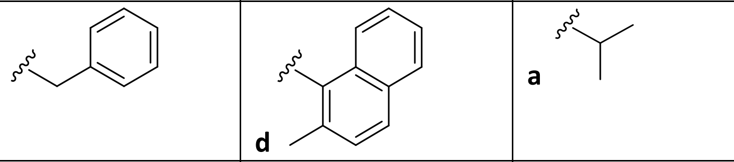 **e**	**d**	**a**	Gly
**55**	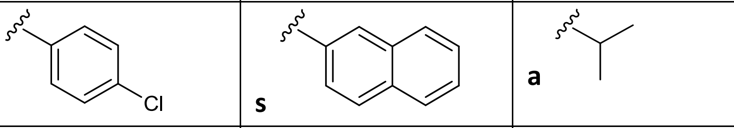 **f**	**s**	**a**	Gly
**56**	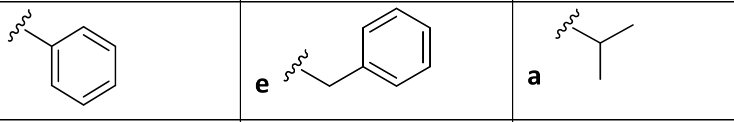 **c**	**e**	**a**	Gly
**57**	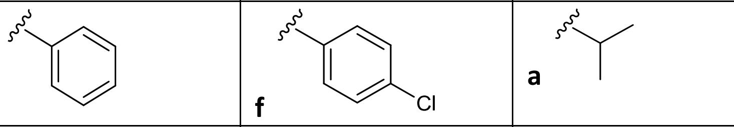 **c**	**f**	**a**	Gly
**58**	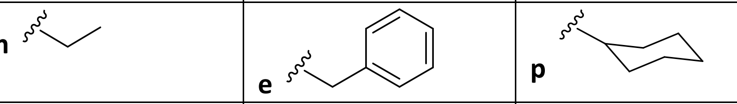 **m**	**e**	**p**	Val
**59**	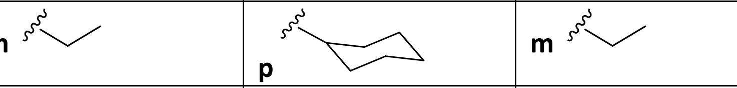 **m**	**p**	**m**	Ile
**60**	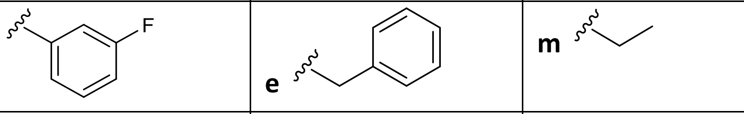 **i**	**e**	**m**	Leu
**61**	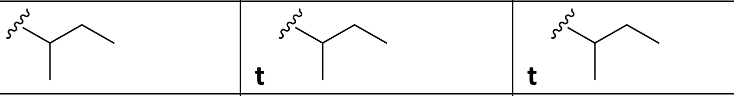 **t**	**t**	**t**	Ile
**62**	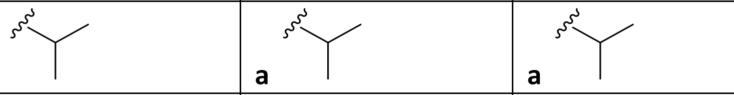 **a**	**a**	**a**	Gly (Val on top)
**63**	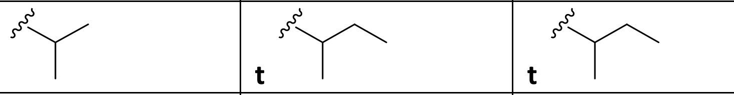 **a**	**t**	**t**	Ile
**64**	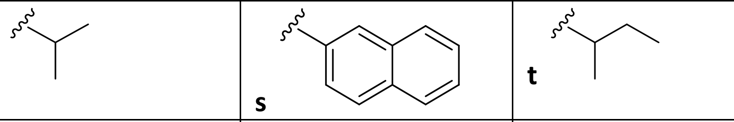 **a**	**s**	**t**	Ile
**65**	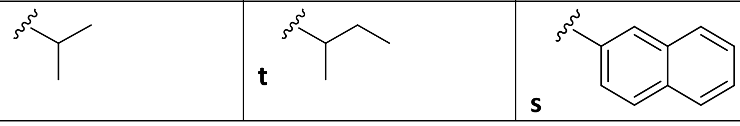 **a**	**t**	**s**	Val

Compound No.	N terminus (R_1)	Middle residue (R_2)	C terminus (R_3)	Amino Acid (R_4)
**66**	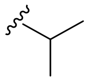 **a**	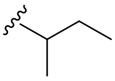 **t**	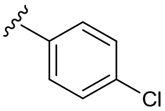 **f**	Val
**67**	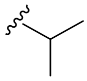 **a**	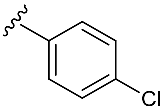 **f**	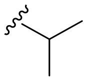 **a**	Ile
**68**	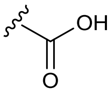 **r**	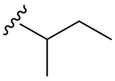 **t**	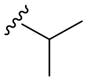 **a**	Val
**69**	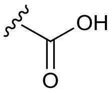 **r**	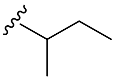 **t**	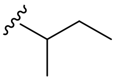 **t**	Val
**70**	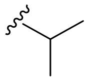 **a**	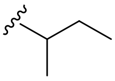 **t**	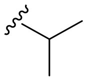 **a**	Asp
**71**	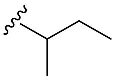 **t**	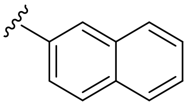 **s**	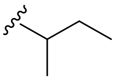 **t**	Ile
**72**	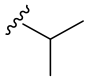 **a**	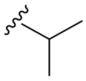 **a**	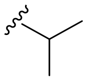 **a**	Gly
**73**	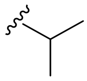 **a**	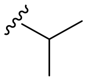 **a**	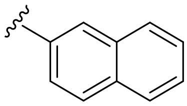 **s**	Leu
**74**	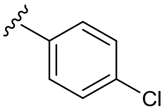 **f**	**N/A**	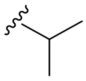 **a**	Ile
**75**	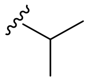 **a**	**N/A**	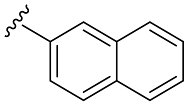 **s**	Ile
**76**	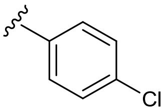 **f**	**N/A**	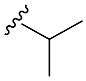 **a**	Gly
**77**	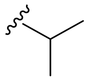 **a**	**N/A**	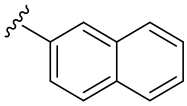 **s**	Gly

Biophysical and Cellular Analyses

Protein Expression and Fluorescence Anisotropy

Expression of *h*DM2 and fluorescence anisotropy assays were performed as described previously.[2] Expression of Bcl-x_L and fluorescence anisotropy assays were performed as described previously.[3] Expression of Mcl-1 and fluorescence anisotropy assays were performed as described previously.[4] Direct binding assays were performed as described previously.[4] Fluorescence anisotropy assays were performed in 384-well plates (Greiner Bio-one). Each

experiment was run in triplicate and the fluorescence anisotropy measured using a Perkin Elmer EnVisionTM 2103 MultiLabel plate reader, with excitation at 480 nm and emission at 535 nm (5 nM bandwidth). All experiments were performed in assay buffer: 40 mM phosphate buffer at pH 7.50, containing 200 mM NaCl and 0.02 mg mL^{-1} *bovine serum albumin* (BSA) and data analysed following previously published methods.[5, 6]

^{15}N HSQC NMR binding experiments

^{15}N HSQC NMR binding experiments were performed as described previously with 160 µM protein in phosphate buffer (100 mM sodium phosphate, 1 mM DTT, 2.5% glycerol and 5% DMSO).[6]

Cell culture

U2OS, SJSA-1 and Saos-2 cells were cultured in Dulbecco's Modified Eagle's Medium (DMEM) and RPMI-1640 media respectively supplemented with 10% foetal bovine serum, penicillin-streptomycin and 2 mM L-glutamine.

Cell Viability

Cells were plated in 96-well plates at 10^4 cells/well 24 hours before compound addition. Compounds were added as 50 µM stocks in serum-free media (Opti-MEM, 100 µl) and the cells were incubated for 18 hours. 15 µl of Dye Solution (Promega Cell Titre 96®) was added and the cells were incubated for 4 hours. 100 µl of Stop Solution (Promega Cell Titre 96®) was added and the solutions were mixed using a multi-channel pipette. After incubating for 1 hour the absorbance of each well was measured at 570 and 600 nm.

High Content Screening Assay

Cells were plated into 96-well plates (Viewpoint plates, Perkin Elmer, MA) at 4000 cells/well using an xrd-384 Fluid X dispenser fitted with an 8 nozzle resin dispensing cassette at 300 rpm (Fluid X Ltd, Nether Alderley, UK). Plates were allowed to equilibrate at room temperature in a hood for 1 hr then incubated for 24 hrs at 37°C and 5% CO_2 before media was replaced with serum-free media (Opti-MEM) prior to compound addition. Compound stocks (10 mM in DMSO) were diluted 1:100 with Opti-MEM and added to the plated cells at final concentrations of 10 and 20 µM giving a final DMSO percentage of 0.2%. Library addition was performed using a Bravo SRT liquid handling platform (Agilent Technologies, Wokingham, UK). Plates were assayed 48 hrs after compound addition using immunofluorescent staining and high-content imaging approaches as detailed below. Compound precipitation was visually assessed on addition and at imaging. Assays were developed and validated using Nutlin 3a and ABT-737 at a variety of concentrations.

Immunofluorescent staining of assay plates was carried out as follows. Media was discarded and cells rinsed in phosphate buffered saline (PBS), before fixation in 4% paraformaldehyde (SigmaAldrich) for 15 mins. Cells were permeabilized with 0.1% Triton X-100 (VWR) in PBS for 5 mins, cells were then rinsed in PBS and blocked in 1% milk (Marvel, Premier Foods, St Albans, UK) for 5 mins before the addition of antibodies against LC3B (5F10, Enzo Life Sciences) and caspase 3 (Abcam, ab13847) diluted in 1% milk for 1 hr at room temperature. Following PBS rinses,

cells were incubated at room temperature for an hour in the dark with 1% milk containing goat anti-mouse AlexaFluor 568 (Molecular Probes), goat anti-rabbit AlexaFluor 633 (Molecular Probes), 1 µg/mL DAPI (Molecular Probes) and AlexaFluor-488 conjugated Phalloidin (Molecular Probes). Following a final set of PBS washes, plates were scanned and images collected with an Operetta HTS imaging system (PerkinElmer) at 20x magnification with 11 fields of view (510 x 675 µm)/well. Images were then analysed with Columbus 2.2 (PerkinElmer).

During assay development statistical analysis (Student T-tests, Mann-Whitney U tests and Spermann Rank correlation) was carried out using GraphPad Prism 6.00 software (GraphPad Software, La Jolla, CA). Screening hits were identified by Z-scores calculated using the following formula where *s* is the positive control and *c* the negative controls:

$$Z = (\textit{sample-value} - \mu_c)/\sigma_c$$

with hits determined as an absolute Z-score of 2 or more, as this correlates to a *P* value of 0.045, and thus statistical significance.[7, 8] False positives were defined as any DMSO-treated well in which the absolute Z score was greater than 2 whilst false negatives were Nutlin 3a-treated wells which had an absolute Z score of less than 2 on three independent, randomly distributed test plates.

Cell Lysis and Western blotting

Cells were lysed with 100 µl of lysis buffer (50 mM Tris HCl pH 8.0, 150 mM NaCl, 1 mM EDTA and 0.5 % Nonidet P-40) and the lysate was analysed by SDS PAGE electrophoresis and probed with antibodies against p21 (C-19, Santa Cruz, sc-397), and GAPDH (6C5, Abcam, ab8245).

Biotin-tag assays

Cells were plated in 10 cm dishes 24 hours before compound addition. Cells were treated with 10 µM of the biotinylated trimers in Opti-MEM and incubated for 4 hours. Cells were lysed with 100 µl of lysis buffer (50 mM Tris HCl pH 8.0, 150 mM NaCl, 1 mM EDTA and 0.5 % Nonidet P-40). Cell lysate was incubated with Streptavidin-coated magnetic beads (Sigma Aldrich) for 2 hours at 4 °C, followed by extensive washing of the protein complexes. Complexes were denatured by SDS PAGE electrophoresis and probed with antibodies against *h*DM2 (C-18, Santa Cruz, sc-812), Mcl-1 (S-19, Santa Cruz, sc-819) and Bcl-x_L (H-62, Santa Cruz, sc-7195).

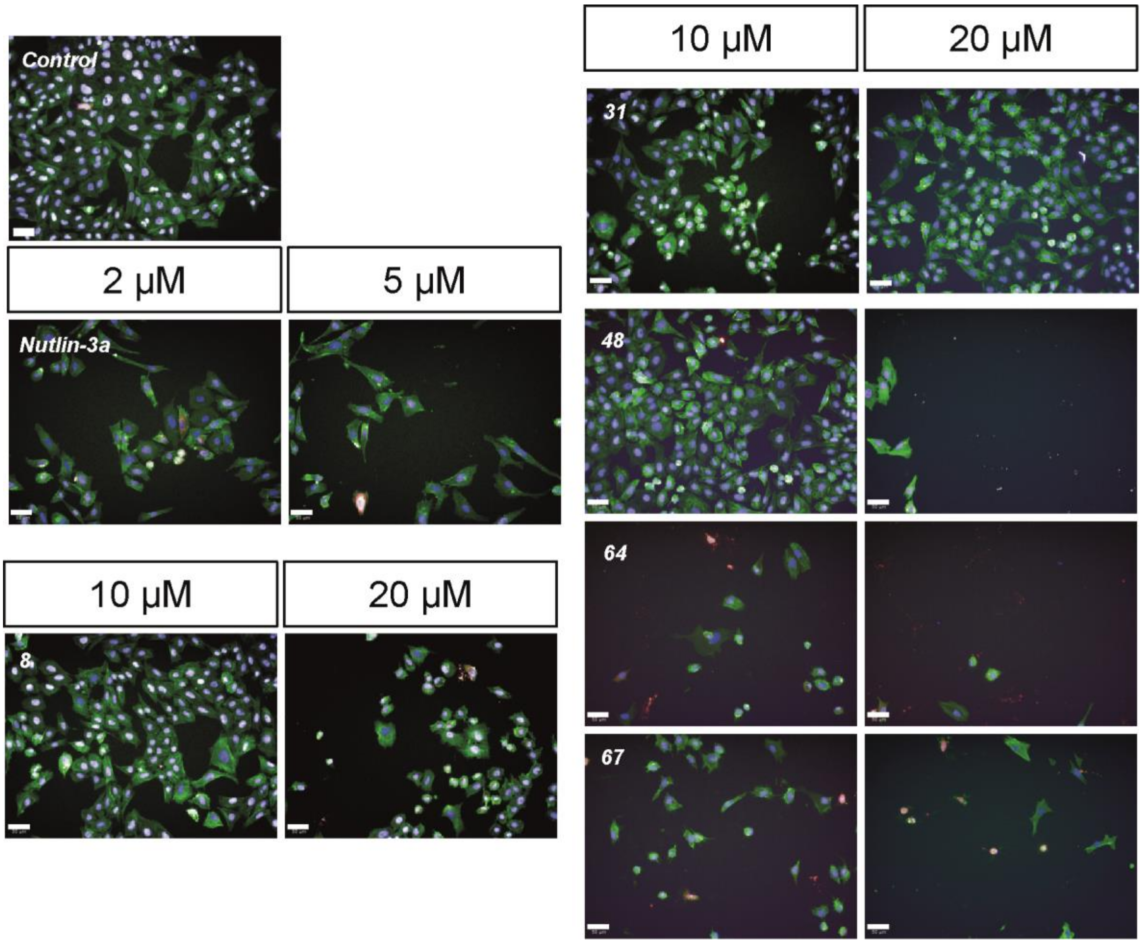

Figure S2 Additional HCS summary for mimetic library (a). Example images of the five compounds (10 and 20 µM) and controls of 0.2% DMSO and Nutlin-3a (2 and 5 µM) scale = 50 µm. DAPI staining is shown in the blue channel, caspase 3 is shown in red, LC3B as yellow and conjugated phalloidin shown in green.

Table S2a - Summary of the results from the high-content assays for helix mimetics **1-77.** U2OS cells were treated with 2 different concentrations of the helix mimetics (10 and 20 µM) and cell number assessed

		**Cell Number**							
		**Z-score**							
		**10 µM**				**20 µM**			
**Compound Number**	**Summary**	**1**	**2**	**3**	**4**	**1**	**2**	**3**	**4**
**1**	**-**	-0.09	-1.82	**-5.06**	**-3.37**	-1.92	-1.95	**-2.43**	**-3.94**
**2**	**20 µM, some 10µM**	**-2.51**	-1.28	**-4.41**	**-3.18**	**-5.17**	**-4.79**	**-8.50**	**-8.17**
**3**	**-**	1.08	0.40	1.93	-0.53	-0.08	-0.59	0.29	-0.86
**4**	**Hit in all replicates**	**-3.92**	**-2.50**	**-5.46**	**-3.78**	**-2.34**	**-2.87**	**-5.45**	**-4.63**
**5**	**-**	-1.00	-1.17	**-7.81**	**-3.93**	-0.70	-1.28	-0.77	**-4.18**
**6**	**-**	-0.33	-1.11	-1.90	**-3.74**	-1.15	-0.96	**-2.15**	**-3.66**
**7**	**-**	-1.95	**-2.25**	**-4.46**	**-2.09**	-1.90	-1.28	**-2.35**	-1.85
**8**	**-**	-0.95	-0.13	**-3.08**	**-2.02**	**-4.27**	**-2.34**	**-5.23**	-1.21
**9**	**-**	0.54	-1.90	-1.03	**-2.94**	0.13	-1.12	**-3.18**	**-3.52**
**10**	**-**	0.01	-1.00	1.62	-0.52	-1.08	-1.93	**-2.44**	**-2.76**
**11**	**-**	0.47	0.68	0.51	-1.32	0.54	-0.49	-0.74	**-2.58**
**12**	**-**	1.25	0.92	1.48	-0.81	-0.67	-0.42	-1.10	-0.83
**13**	**-**	-0.46	-1.11	1.44	-0.67	**-2.44**	-1.13	**-2.88**	**-2.22**
**14**	**-**	-0.40	1.05	0.19	0.08	-0.60	-0.71	-1.96	**-2.12**
**15**	**Hit in all replicates**	**-3.53**	**-3.00**	**-3.64**	**-2.34**	**-3.83**	**-3.03**	**-8.06**	**-4.12**
**16**	**-**	0.44	-1.32	-1.89	**-2.90**	-0.55	-0.71	-1.35	**-2.46**
**17**	**-**	-0.34	**-2.74**	**-2.32**	**-4.20**	-1.17	-1.81	**-3.06**	**-4.48**
**18**	**-**	-0.98	-1.67	**-4.56**	**-2.56**	-1.01	-1.23	-1.75	**-4.85**
**19**	**-**	0.83	0.74	**2.74**	**2.39**	1.18	-0.62	-0.93	-0.76
**20**	**20 µM only**	-1.98	-1.07	**-3.41**	-1.78	**-4.96**	**-3.02**	**-8.56**	**-7.22**
**21**	**-**	0.99	0.71	-0.12	0.99	0.50	-0.50	-0.81	-0.59
**22**	**-**	0.43	-1.48	1.72	0.49	0.15	1.07	-0.45	0.21
**23**	**-**	-0.56	0.18	**-2.54**	-0.26	**-5.30**	-1.13	**-9.07**	**-2.71**
**24**	**-**	0.70	-0.01	**-2.42**	-0.80	-0.10	-0.21	-1.51	**-2.34**
**25**	**Hit in all replicates**	**-2.25**	**-3.30**	**-4.79**	**-5.61**	**-2.57**	**-2.22**	**-5.16**	**-5.49**
**26**	**-**	1.55	-0.74	-1.04	**-2.31**	-0.52	-1.61	0.53	**-2.96**
**27**	**-**	0.28	0.60	-0.81	**-4.00**	-1.55	-0.51	**-3.20**	-1.70
**28**	**20 µM only**	0.40	**2.04**	0.69	0.79	**-5.19**	**-2.01**	**-9.61**	-1.99

		Cell Number							
		Z-score							
		10 µM				20 µM			
**Compound Number**	**Summary**	**1**	**2**	**3**	**4**	**1**	**2**	**3**	**4**
**29**	**20 µM, some 10µM**	**-3.06**	-0.99	**-4.79**	-1.77	**-5.28**	**-3.34**	**-10.02**	**-7.94**
**30**	**20 µM only**	-0.03	1.50	**-3.56**	-0.62	**-5.30**	**-2.30**	**-9.93**	**-5.80**
**31**	-	0.67	0.04	**-2.11**	-0.59	-0.36	-0.40	**-2.55**	-1.01
**32**	-	1.35	0.35	**-3.30**	-0.73	-0.11	0.04	-0.05	-1.38
**33**	**20 µM, some 10µM**	-1.82	**-2.62**	**-6.17**	**-4.88**	-1.42	**-2.68**	**-4.37**	**-3.66**
**34**	-	-1.04	-0.57	0.81	-1.97	0.31	-1.30	0.31	-1.23
**35**	-	**2.82**	-1.57	-0.42	**-2.58**	0.34	-0.49	0.61	-0.83
**36**	-	-0.30	0.32	-1.05	**-2.39**	**-5.08**	-1.28	**-9.38**	**-2.84**
**37**	**20 µM, some 10µM**	**-2.72**	0.73	0.06	0.04	**-5.29**	**-4.38**	**-9.97**	**-6.32**
**38**	**20 µM, some 10µM**	**-5.44**	-1.49	**-6.30**	-1.70	**-5.29**	**-3.89**	**-9.31**	**-6.30**
**39**	**20 µM, some 10µM**	**-5.24**	**-3.58**	**-5.59**	-1.79	**-5.28**	**-5.17**	**-10.03**	**-9.97**
**40**	**20 µM, some 10µM**	**-4.91**	-1.11	-0.76	**-2.29**	**-5.19**	**-3.36**	**-9.88**	**-3.63**
**41**	**Hit in all replicates**	**-4.23**	**-4.19**	**-6.45**	**-5.30**	**-6.40**	**-6.42**	**-9.09**	**-9.05**
**42**	**20 µM only**	-0.02	0.52	0.88	-1.82	**-3.17**	**-2.22**	**-3.13**	**-2.24**
**43**	-	-0.37	0.09	-0.27	-1.29	**-3.86**	-1.94	**-3.61**	-1.62
**44**	-	1.41	0.10	**2.02**	-1.04	-0.82	-0.54	-1.71	-0.91
**45**	**20 µM, some 10µM**	0.32	-0.50	-1.95	**-2.28**	**-2.24**	-1.52	**-4.10**	**-3.12**
**46**	-	0.32	0.32	**-2.18**	-0.22	**-2.73**	-1.41	**-5.01**	**-2.99**
**47**	-	0.21	-0.52	-1.72	-0.87	-1.10	-1.46	**-2.01**	**-3.00**
**48**	**20 µM, some 10µM**	-1.75	-0.39	**-6.95**	**-2.93**	**-6.40**	**-6.13**	**-8.98**	**-6.25**
**49**	**20 µM only**	-0.45	-1.48	-1.20	-1.09	**-3.13**	**-3.11**	**-5.96**	**-3.04**
**50**	-	0.37	0.46	**-2.53**	0.29	-0.73	-0.44	**-3.02**	-0.98
**51**	**20 µM only**	1.28	-0.29	-0.02	-0.33	**-2.69**	**-4.48**	**-5.04**	**-6.24**
**52**	-	1.13	-1.42	**-3.86**	**-2.41**	-1.27	-1.90	**-3.12**	**-2.05**
**53**	**20 µM only**	0.51	0.48	-1.81	0.87	**-2.48**	**-2.76**	**-3.65**	**-3.15**
**54**	**Hit in all replicates**	**-4.92**	**-3.78**	**-9.22**	**-8.22**	**-6.41**	**-6.37**	**-9.05**	**-9.03**
**55**	-	-0.14	**-2.14**	**-2.12**	-0.36	**-4.23**	**-3.41**	**-4.07**	**-2.69**
**56**	-	1.46	0.13	1.82	-0.27	0.56	0.13	-0.99	0.47
**57**	-	0.55	-0.30	1.87	1.06	1.33	-0.33	-0.61	-1.55
**58**	**Hit in all replicates**	**-5.97**	**-4.88**	**-11.64**	**-7.49**	**-6.39**	**-6.23**	**-9.05**	**-8.91**
**59**	**20 µM, some 10µM**	**-3.59**	**-3.43**	**-9.27**	**-4.29**	**-6.41**	**-5.56**	**-8.75**	**-5.91**

		Cell Number							
		Z-score							
		10 µM				20 µM			
**Compound Number**	**Summary**	**1**	**2**	**3**	**4**	**1**	**2**	**3**	**4**
**60**	20 µM, some 10µM	**-2.15**	-1.63	**-6.44**	**-2.55**	**-6.27**	**-5.01**	**-8.00**	**-4.82**
**61**	Hit in all replicates	**-6.57**	**-6.10**	**-12.33**	**-10.37**	**-6.42**	**-6.38**	**-9.06**	**-8.99**
**62**	-	0.84	-0.16	0.42	**2.28**	0.29	-0.95	0.16	0.26
**63**	Hit in all replicates	**-6.40**	**-5.55**	**-11.73**	**-8.14**	**-6.34**	**-6.33**	**-9.02**	**-8.59**
**64**	Hit in all replicates	**-6.43**	**-6.21**	**-11.44**	**-11.33**	**-6.30**	**-6.40**	**-9.07**	**-8.83**
**65**	Hit in all replicates	**-6.58**	**-5.73**	**-12.35**	**-11.40**	**-6.39**	**-6.39**	**-8.96**	**-8.99**
**66**	Hit in all replicates	**-6.53**	**-6.50**	**-12.25**	**-12.06**	**-6.35**	**-6.33**	**-8.93**	**-8.99**
**67**	Hit in all replicates	**-5.84**	**-5.24**	**-11.16**	**-9.70**	**-6.35**	**-6.24**	**-8.93**	**-8.59**
**68**	-	-0.17	-0.41	**-2.09**	**-2.82**	-1.31	-1.75	-1.73	-0.42
**69**	20 µM, some 10µM	-1.84	**-2.38**	**-4.03**	**-2.25**	**-3.96**	**-3.12**	**-4.76**	**-3.74**
**70**	-	-0.26	-1.09	0.13	0.51	0.15	-1.28	-0.56	0.06
**71**	Hit in all replicates	**-6.37**	**-5.45**	**-12.17**	**-9.06**	**-6.32**	**-6.39**	**-9.01**	**-7.20**
**72**	-	0.38	-0.47	1.67	-0.51	0.80	-0.41	-0.58	0.49
**73**	Hit in all replicates	**-6.41**	**-4.99**	**-10.05**	**-7.26**	**-6.39**	**-6.34**	**-8.89**	**-8.50**
**74**	20 µM, some 10µM	**-2.58**	-1.79	-1.95	**-2.82**	**-4.13**	**-3.94**	**-4.28**	**-4.69**
**75**	20 µM, some 10µM	**-2.71**	-0.55	-1.84	-1.31	**-4.94**	**-5.16**	**-5.16**	**-5.19**
**76**	-	-0.45	-0.41	-1.39	-1.72	0.71	0.16	-0.09	-0.91
**77**	-	0.16	1.07	-1.09	-0.29	0.08	0.99	-0.51	-0.98

Table S2b - Summary of the results from the high-content assays for helix mimetics **1-77**. U2OS cells were treated with 2 different concentrations of the helix mimetics (10 and 20 µM) and cells active for Caspase-3 quantified

		Caspase-3 positive cells							
		Z-score							
		10 µM				20 µM			
**Compound Number**	**Summary**	**1**	**2**	**3**	**4**	**1**	**2**	**3**	**4**
**1**	-	-0.24	-0.94	**2.75**	-1.97	-0.45	-0.13	-0.48	-0.78
**2**	-	-0.21	1.38	**8.80**	-0.24	**26.66**	**6.09**	**12.35**	0.00
**3**	-	0.26	0.84	1.19	**2.15**	1.04	-0.14	0.93	-0.86
**4**	**20 µM, some 10µM**	**2.11**	**4.38**	-0.34	-1.89	**13.91**	**4.12**	**8.08**	0.54
**5**	-	0.47	**2.59**	**37.37**	**4.81**	**3.88**	**4.07**	1.05	0.14
**6**	-	0.25	0.82	0.53	**2.77**	0.40	0.55	1.01	-0.29
**7**	-	-0.20	1.36	**4.32**	0.60	1.87	-0.22	1.01	-0.49
**8**	-	-0.01	0.19	-1.59	-0.22	**22.56**	0.65	**3.88**	-0.84
**9**	-	-0.63	-1.62	-1.96	0.54	-0.53	0.14	0.12	-1.20
**10**	-	1.26	0.78	**2.48**	-1.33	**2.57**	-0.04	0.28	-1.10
**11**	-	1.73	-0.91	**3.95**	**3.29**	0.04	-0.38	0.68	-0.53
**12**	-	**3.47**	**2.05**	**-2.57**	0.15	0.71	0.24	1.74	-0.97
**13**	-	1.89	0.35	**3.46**	**2.22**	**14.40**	1.72	1.93	-0.69
**14**	-	1.14	0.02	0.19	-0.11	0.53	0.81	0.71	-0.82
**15**	-	-0.04	-1.49	1.32	-0.11	0.64	0.29	0.40	-0.04
**16**	-	0.56	1.40	**2.86**	1.80	0.72	0.66	0.00	-0.35
**17**	-	-0.46	-0.93	**2.31**	1.63	-0.34	-0.59	-0.12	-0.72
**18**	-	-0.15	0.56	**3.90**	1.62	0.18	-0.57	-1.03	-0.85
**19**	-	-0.18	-0.41	**4.53**	1.04	-0.34	-0.35	0.62	-1.03
**20**	-	**6.53**	0.50	**3.90**	0.87	**3.05**	**2.69**	**4.45**	0.52
**21**	-	-0.71	**4.31**	1.29	0.89	1.72	0.63	-0.87	-0.93
**22**	-	1.49	-0.01	1.55	-1.19	-0.22	-1.08	-0.94	-0.98
**23**	-	1.50	1.19	**3.68**	0.32	-1.67	1.22	**3.61**	-1.10
**24**	-	0.11	-0.25	-0.49	0.50	-0.35	-0.45	-0.48	-0.74
**25**	-	-0.66	0.09	-0.09	**2.06**	0.84	-0.33	-1.10	-1.28
**26**	-	-0.09	-0.82	0.59	**-2.15**	-0.01	-0.09	-0.49	-1.36
**27**	-	-0.05	-1.42	**2.64**	1.99	-0.48	-0.02	**2.09**	-0.97
**28**	-	0.84	0.37	-0.28	**3.80**	**26.29**	0.84	**10.98**	-1.07

Compound Number	Summary	Caspase-3 positive cells Z-score 10 µM 1	2	3	4	20 µM 1	2	3	4
**29**	-	**16.78**	-0.62	**6.21**	1.64	**28.83**	**6.35**	-1.49	1.18
**30**	-	**3.34**	-0.44	-0.11	0.09	**10.19**	-0.75	**8.80**	-0.93
**31**	-	-0.02	-0.95	1.01	-1.67	0.42	-0.83	-0.91	-0.96
**32**	-	0.31	0.06	**3.82**	**4.01**	-0.14	0.27	-0.77	-0.83
**33**	-	-0.59	-0.56	**3.15**	-0.58	-0.61	-0.75	-0.99	-1.04
**34**	-	0.33	-0.85	1.58	-1.80	-0.56	-0.98	-1.12	-1.22
**35**	-	-0.23	-0.31	1.06	-0.86	1.15	-0.88	-0.36	-0.13
**36**	-	0.53	-0.99	-0.50	**41.98**	**23.62**	-0.04	**14.49**	-0.89
**37**	**20 µM, some 10µM**	**7.59**	-0.29	**2.22**	**4.56**	**6.54**	**4.89**	**8.49**	-0.21
**38**	**20 µM, some 10µM**		**3.80**	**2.00**	**3.52**	**39.39**	1.71	**5.13**	-0.60
**39**	-	**7.42**	-0.48	**3.78**	-0.67	**19.68**	**17.94**	**4.17**	1.84
**40**	-	**8.14**	0.20	-2.00	**3.11**	**21.21**	-0.79	**2.70**	-0.97
**41**	-	1.91	-0.14	-1.99	-0.32	**20.62**	-1.74	**-3.00**	**-3.00**
**42**	-	-0.10	-0.75	0.66	-0.29	**3.92**	0.63	**4.66**	0.42
**43**	-	0.46	1.08	0.62	0.55	**4.42**	**2.86**	**3.76**	0.96
**44**	-	0.69	0.67	-0.64	1.03	0.76	1.02	0.18	0.77
**45**	-	1.96	1.42	0.75	**2.32**	**14.21**	**6.98**	**6.40**	0.93
**46**	-	0.18	0.58	0.00	0.26	**7.44**	**6.86**	**4.86**	1.04
**47**	-	0.83	-1.26	1.07	-1.59	0.12	-0.70	**-2.13**	-1.79
**48**	**20 µM, some 10µM**	**2.53**	0.03	**4.63**	-0.91	**8.20**	**9.28**	**25.62**	**7.38**
**49**	-	-0.50	-1.32	-0.80	**-2.13**	-0.51	-0.63	0.14	-0.35
**50**	-	1.14	0.61	**3.10**	-0.69	1.62	0.78	0.05	0.65
**51**	**20 µM, some 10µM**	**2.73**	**3.34**	-0.13	0.46	**24.29**	**20.15**	**13.14**	**6.06**
**52**	**20 µM, some 10µM**	1.94	0.12	**6.46**	0.06	**16.73**	**6.00**	**2.78**	1.56
**53**	-	-0.08	-0.94	-1.70	-1.26	**2.64**	0.67	-0.96	**5.72**
**54**	**20 µM, some 10µM**	**17.21**	**3.14**	**9.76**	**5.33**	-1.74	-1.74	**39.93**	**28.22**
**55**	-	-1.32	-0.85	1.83	-1.46	**9.31**	1.07	**2.89**	-0.30
**56**	-	-0.09	0.76	1.18	0.62	-0.16	-0.34	-0.10	-0.68
**57**	-	0.86	1.51	**2.21**	-0.02	0.49	0.56	-0.52	-0.22
**58**	**20 µM, some 10µM**	**19.43**	**2.31**	**7.47**	**4.14**	**25.09**	**23.30**	**-3.00**	**21.53**
**59**	**20 µM, some 10µM**	**16.99**	**7.02**	-0.04	**2.52**	-1.74	**5.04**	**-3.00**	**6.67**

		Caspase-3 positive cells							
		Z-score							
		10 µM				20 µM			
**Compound Number**	**Summary**	**1**	**2**	**3**	**4**	**1**	**2**	**3**	**4**
**60**	-	1.14	-0.33	**2.96**	-0.33	**24.44**	1.12	1.46	**3.06**
**61**	**20 µM, some 10µM**	**-2.38**	**5.30**	**111.48**	**16.57**	-1.74	**4.65**	**21.53**	**27.30**
**62**	-	-1.28	-0.69	0.45	-0.24	0.67	0.82	1.54	1.89
**63**	**20 µM, some 10µM**	**38.72**	**8.34**	**8.72**	**7.64**	**2.32**	**18.90**	**-3.00**	**25.62**
**64**	**20 µM, some 10µM**	**44.37**	**7.35**	-0.63	**14.00**	**25.36**	-1.74	**31.34**	**45.43**
**65**	**Hit in all replicates**	**9.78**	**8.22**	**31.04**	**5.20**	**38.92**	**20.62**	**21.53**	**39.93**
**66**	**Hit in all replicates**	**52.11**	**54.38**	**15.86**	**28.23**	**15.29**	**30.46**	**11.31**	**39.93**
**67**	**Hit in all replicates**	**29.77**	**3.05**	**11.82**	**5.74**	**2.73**	**9.44**	**65.69**	**25.62**
**68**	-	-0.79	-0.29	1.20	0.40	0.28	-0.40	**2.87**	1.27
**69**	-	-0.24	-0.77	-1.43	**-2.07**	0.65	0.48	-0.72	-0.93
**70**	-	-0.23	1.08	-1.51	0.10	0.50	0.26	0.19	-0.98
**71**	**20 µM, some 10µM**	**42.16**	**5.15**	**17.92**	**6.67**	**4.65**	**14.52**	**-3.00**	**10.61**
**72**	-	1.43	-0.16	1.68	-0.20	1.37	-0.21	0.92	-0.94
**73**	**20 µM, some 10µM**	**31.07**	**2.62**	0.82	**6.30**	-1.74	**18.59**	**8.45**	**23.26**
**74**	-	1.13	-0.89	-0.36	0.69	0.92	0.59	**3.94**	1.83
**75**	-	-0.39	-1.02	-1.13	-0.95	**4.96**	-1.37	-1.37	-1.37
**76**	-	-1.20	-0.86	-0.94	-1.13	-0.95	-1.37	-0.57	-1.18
**77**	-	0.00	-0.18	**4.99**	0.63	0.02	-0.71	-0.50	-0.79

Table S2c - Summary of the results from the high-content assays for helix mimetics **1-77**. U2OS cells were treated with 2 different concentrations of the helix mimetics (10 and 20 µM) and autophagic cells quantified.

		**Autophagic cells**							
		**Z-score**							
		**10 µM**				**20 µM**			
**Compound Number**	**Summary**	**1**	**2**	**3**	**4**	**1**	**2**	**3**	**4**
**1**	-	-0.11	-0.69	0.00	-0.61	0.66	-1.04	-0.23	0.17
**2**	**20 µM, some 10µM**	1.19	0.43	**3.10**	0.53	**70.85**	**11.61**	**23.60**	**12.73**
**3**	-	-0.33	-0.61	0.22	-0.12	1.42	-0.37	-0.06	-0.85
**4**	-	-1.63	0.32	**6.92**	1.60	1.02	-0.12	**7.60**	**2.73**
**5**	-	-0.83	0.16	**5.61**	1.15	0.61	**4.54**	0.82	**5.04**
**6**	-	-1.57	-0.34	0.17	0.13	-1.20	-0.57	-0.69	0.57
**7**	-	-0.93	-1.72	-0.85	-0.43	-1.47	-0.82	0.16	0.41
**8**	-	**2.89**	-0.94	-0.39	0.18	-1.89	-1.41	**8.48**	-0.10
**9**	-	-1.21	-1.47	-0.28	**2.30**	-1.10	-1.20	-0.97	-0.92
**10**	-	-1.37	-1.53	-0.54	-0.49	-0.19	**2.80**	**3.06**	**7.58**
**11**	-	-0.81	-0.65	-0.51	-0.07	-1.40	-0.70	-1.21	0.64
**12**	-	-1.09	-1.43	-0.38	-0.48	-1.27	-0.71	-0.48	-0.52
**13**	-	-1.34	-1.52	0.10	0.07	-0.89	-0.51	-1.01	-1.08
**14**	-	-1.13	0.05	-0.16	1.24	-1.28	-1.27	-0.72	-0.30
**15**	-	-1.31	-1.06	-0.85	-0.85	-1.89	-1.89	-1.88	-1.88
**16**	-	-1.80	-1.75	-0.44	-0.63	1.14	**8.76**	-1.52	**3.90**
**17**	-	-1.56	**2.50**	-0.85	0.18	-1.20	-1.48	-0.54	-0.76
**18**	-	-0.83	-1.49	-0.31	-0.63	-0.55	-1.19	-1.12	-0.08
**19**	-	-1.62	-1.04	-0.70	-0.41	-1.67	-1.28	-1.19	-1.54
**20**	-	-2.00	-1.29	0.10	-0.04	**2.05**	-0.01	**8.30**	1.40
**21**	-	-1.25	-1.61	-0.85	-0.36	0.10	-1.29	-0.86	-1.22
**22**	-	-0.61	-0.75	-0.54	-0.18	-0.84	-0.08	-1.23	-0.35
**23**	-	-2.00	**3.30**	-0.42	0.04	-1.89	-0.17	**7.18**	-1.03
**24**	-	-1.23	-0.96	0.01	-0.48	-1.34	-1.04	-0.41	0.96
**25**	-	-1.44	**5.27**	-0.85	0.37	-1.37	-1.43	0.03	0.86
**26**	-	-1.47	-1.54	-0.66	-0.64	-0.99	0.05	-1.88	0.77
**27**	-	-1.59	-1.22	-0.66	-0.10	-0.74	-0.39	-0.97	0.75
**28**	-	-1.40	-1.83	-0.18	-0.35	**8.72**	-1.46	-1.88	0.84

		Autophagic cells							
		Z-score							
		10 µM				20 µM			
**Compound Number**	**Summary**	**1**	**2**	**3**	**4**	**1**	**2**	**3**	**4**
**29**	-	-1.05	-0.83	-0.30	-0.85	**61.76**	**3.20**	-1.88	**3.92**
**30**	-	-1.37	-1.29	-0.61	-0.85	**47.61**	-1.89	-1.88	**2.52**
**31**	-	-1.61	-0.96	-0.43	-0.48	-1.60	-1.30	-1.04	-1.53
**32**	-	-1.46	-1.60	0.79	-0.29	-1.06	-1.08	-0.31	-0.07
**33**	-	-0.95	-1.11	-0.19	-0.57	-1.89	-1.89	-1.33	-1.39
**34**	-	-1.29	-1.11	-0.19	-0.03	-1.89	-1.17	-0.36	-1.17
**35**	-	-0.89	0.78	-0.85	-0.85	-0.61	-1.29	-1.29	-0.18
**36**	-	-1.14	-1.19	-0.85	1.07	-1.89	-1.53	**11.13**	1.60
**37**	-	-0.80	-1.61	-0.33	-0.15	**32.38**	-0.37	**19.79**	**2.29**
**38**	-	**26.37**	-0.75	-0.51	-0.65	-1.89	1.13	**5.96**	1.43
**39**	-	-2.00	-0.95	-0.55	-0.65	-1.89	-1.89	-1.88	**19.79**
**40**	-	-1.07	-0.57	-0.29	-0.43	-1.89	-0.42	**11.77**	0.56
**41**	-	**-3.58**	-1.18	**4.06**	-1.14	**13.33**	-1.14	**-2.42**	**-2.42**
**42**	-	-1.36	**-2.76**	-0.57	1.64	-0.55	-0.68	-0.70	-0.93
**43**	-	-1.24	-1.84	-0.85	**-2.25**	-0.76	-0.28	-0.55	-1.73
**44**	-	**-2.49**	0.34	-0.75	-0.72	-0.79	-0.49	**-2.42**	-1.17
**45**	-	**-3.16**	**-2.15**	1.13	0.11	-0.79	-0.84	**-2.42**	-0.28
**46**	-	**-2.74**	-1.89	**-2.21**	**-2.78**	0.16	-0.37	-0.54	0.51
**47**	-	**-2.72**	-1.66	**-2.78**	-0.75	-0.24	-0.65	-0.61	-1.16
**48**	-	0.02	0.17	0.41	-0.93	-1.14	0.45	**-2.42**	**34.30**
**49**	-	**-2.16**	**-2.44**	**-2.25**	-0.71	-0.27	-0.70	**5.72**	**11.11**
**50**	-	**-2.32**	-1.51	**-2.18**	-1.39	-0.88	-0.90	-1.58	-1.47
**51**	-	**-2.84**	-1.73	**-2.30**	**-2.29**	-1.01	-0.40	2.00	0.26
**52**	-	**-2.83**	**-2.45**	-0.05	-1.02	-0.58	-0.40	-0.28	1.21
**53**	-	-1.94	-1.11	**-2.78**	-1.45	-0.77	-0.74	-1.95	-1.56
**54**	-	**-3.58**	-1.53	0.84	-1.39	-1.14	-1.14	**42.03**	**29.91**
**55**	-	**-2.23**	-0.32	-0.50	-0.83	**IA***	0.13	**10.30**	**6.37**
**56**	-	**-3.58**	-1.85	**-2.36**	-1.33	-1.07	-0.99	-0.84	-1.61
**57**	-	**-2.36**	-0.34	-1.96	-1.04	-0.95	-0.74	-1.21	-1.40
**58**	-	**9.74**	1.43	**18.25**	**9.01**	-1.14	1.18	**86.48**	**10.28**
**59**	-	1.24	1.90	**2.72**	0.10	-1.14	1.06	**-2.42**	**10.40**

Compound Number	Summary	Autophagic cells Z-score 10 µM 1	2	3	4	20 µM 1	2	3	4
60	20 µM only	**-2.93**	-0.65	-0.83	1.37	**4.51**	**2.56**	**69.16**	**26.27**
61	10 µM only	**44.97**	**7.37**	**272.69**	**8.41**	-1.14	-1.14	**-2.42**	**60.33**
62	-	**-2.40**	-1.31	-0.04	-1.18	-0.35	-0.70	**-2.14**	**-2.14**
63	20 µM, some 10µM	**-3.58**	**15.30**	**5.09**	**4.01**	**4.12**	-1.14	**-2.42**	**27.21**
64	Hit in all replicates	**24.98**	**44.97**	**8.59**	**38.15**	**9.38**	**13.33**	**68.70**	**15.82**
65	20 µM, some 10µM	**48.43**	**12.60**	**-2.78**	**8.11**	-1.14	**8.51**	**14.51**	**64.25**
66	20 µM, some 10µM	**25.55**	**62.62**	**73.96**	**25.65**	-1.14	**8.12**	**-2.42**	**-2.42**
67	20 µM, some 10µM	**15.00**	**11.20**	1.67	-0.66	-1.14	**8.51**	**11.81**	**2.52**
68	-	-1.77	**-2.17**	-1.08	-0.34	-0.86	-0.52	**2.79**	**7.32**
69	-	**-2.36**	-0.13	**-2.78**	**-2.20**	-0.56	0.31	**5.25**	**3.31**
70	-	**-2.20**	-1.99	-0.44	-1.42	-0.70	-1.04	**-2.42**	-1.30
71	20 µM, some 10µM	**18.83**	-1.10	**16.17**	**7.56**	**7.13**	**19.90**	**-2.42**	**11.00**
72	-	-1.49	-0.25	-1.94	-0.81	-0.94	-0.74	-1.22	-1.08
73	-	**9.42**	**3.54**	**-2.78**	0.60	**8.51**	-1.14	**-2.42**	1.77
74	-	-0.54	0.76	**2.60**	**3.48**	**4.96**	-0.48	**2.05**	1.66
75	20 µM only	-1.95	0.23	0.80	0.18	**15.96**	-1.95	**22.67**	**71.93**
76	-	-1.56	-0.80	-0.01	1.23	-0.23	-0.05	-0.14	-0.88
77	-	-0.92	-0.10	**24.84**	-0.82	0.67	-0.61	0.02	1.34

Table S2d - Summary of the results from the high-content assays for helix mimetics **1-77**. U2OS cells were treated with 2 different concentrations of the helix mimetics (10 and 20 µM) and cells with altered F-actin quantified.

		Cells with altered F-actin							
		Z-score							
		10 µM				20 µM			
**Compound Number**	**Summary**	**1**	**2**	**3**	**4**	**1**	**2**	**3**	**4**
**1**	**20 µM only**	-0.16	0.71	**-3.59**	-0.35	**-7.53**	**-35.01**	**-5.19**	**-2.11**
**2**	**-**	**-11.68**	-0.06	**-3.63**	-0.01	**-23.35**	**-2.97**	**-5.00**	-0.50
**3**	**-**	**-6.74**	0.74	0.52	0.65	0.62	**-9.38**	0.68	-0.26
**4**	**-**	**-4.22**	0.75	0.73	0.39	**-6.43**	0.65	**-3.01**	-1.40
**5**	**-**	**-3.88**	0.61	**-3.83**	-0.07	**-2.03**	-1.02	0.04	-0.87
**6**	**-**	-0.17	0.63	0.17	-0.28	-0.73	-0.26	-1.23	-0.45
**7**	**-**	**-2.49**	0.72	-0.24	-0.61	**-6.22**	**-3.18**	**-2.67**	-1.03
**8**	**20 µM, some 10µM**	**-4.39**	0.50	**-10.43**	**-2.60**	**-41.69**	**-4.52**	**-7.65**	**-3.00**
**9**	**-**	0.56	0.72	-0.40	-0.62	-0.59	**-22.21**	**-4.09**	-1.38
**10**	**-**	**-8.89**	0.42	0.16	0.65	-1.04	-0.72	0.11	0.58
**11**	**-**	**-3.02**	0.74	0.73	0.55	0.65	0.38	0.39	-0.03
**12**	**-**	0.00	0.73	0.73	0.65	0.09	0.68	-1.30	-0.38
**13**	**-**	**-2.60**	0.74	0.66	0.73	**-10.75**	0.51	-1.46	-0.82
**14**	**-**	0.25	0.69	-0.54	0.33	-1.38	0.35	-0.33	-0.64
**15**	**-**	-1.97	0.68	**-2.28**	-0.15	**-12.76**	-1.32	**-4.10**	**-2.14**
**16**	**20 µM, some 10µM**	-0.96	0.60	**-4.91**	**-3.19**	**-8.09**	**-11.51**	**-4.58**	**-2.66**
**17**	**-**	0.54	0.55	-1.42	-0.33	-0.70	**-7.56**	**-3.33**	**-2.25**
**18**	**-**	**-3.34**	0.72	-0.38	0.73	0.24	-0.11	0.01	0.74
**19**	**-**	-0.06	0.74	0.73	0.66	0.60	0.51	0.92	0.74
**20**	**-**	-0.61	0.73	0.52	0.55	**-8.79**	0.68	**-4.19**	**-2.24**
**21**	**-**	0.32	0.73	0.73	0.58	-0.36	**-2.87**	0.66	-0.01
**22**	**-**	0.61	0.75	0.59	0.66	0.14	0.29	-0.01	-0.55
**23**	**-**	0.55	0.70	0.03	0.73	0.68	0.06	**-6.97**	-1.36
**24**	**20 µM only**	0.59	0.71	-1.34	**-3.21**	**-5.67**	**-4.38**	**-4.21**	**-2.43**
**25**	**20 µM only**	0.26	0.64	**-2.56**	-1.37	**-7.07**	**-4.51**	**-3.68**	**-3.37**
**26**	**-**	-0.89	0.73	-0.14	0.73	0.61	**-2.06**	0.43	0.96
**27**	**-**	0.52	0.75	0.73	0.73	0.59	0.68	0.50	0.68
**28**	**-**	0.75	0.74	**-4.01**	0.66	**-13.34**	0.65	-0.22	0.59

		Cells with altered F-actin							
		Z-score							
		10 µM				20 µM			
Compound Number	Summary	1	2	3	4	1	2	3	4
29	-	**-3.85**	0.75	-0.53	0.73	**-10.79**	0.26	1.25	-1.68
30	-	0.34	0.75	-0.48	0.73	**-5.27**	**-6.84**	**-3.46**	-0.37
31	-	0.72	0.75	0.35	0.31	-1.28	**-4.95**	**-2.61**	-0.51
32	-	0.69	0.66	**-5.51**	-1.83	**-4.71**	-1.12	**-4.52**	**-2.28**
33	20 µM only	0.62	0.70	**-2.91**	0.48	**-2.21**	**-3.82**	**-2.10**	**-2.22**
34	-	-0.58	0.74	0.58	0.64	0.65	-0.37	-0.10	0.98
35	-	0.75	0.74	0.65	0.73	0.68	0.68	0.18	0.78
36	-	0.74	0.72	0.65	**-2.03**	**-12.00**	0.65	**-5.46**	0.15
37	-	-1.27	0.74	0.57	0.65	**-3.44**	0.38	**-3.83**	-0.59
38	20 µM only	**-4.76**	0.75	**-7.62**	0.55	**-28.15**	**-7.72**	**-4.08**	-1.00
39	-	-0.47	0.60	**-3.19**	-0.39	**-6.46**	**-16.88**	-0.48	-0.79
40	-	-1.56	0.54	**-3.88**	**-4.54**	**-35.01**	**-25.54**	**-2.59**	**-6.25**
41	10 µM only	**-19.32**	**-17.27**	**-30.68**	**-22.02**	0.42	0.42	**2.27**	**2.27**
42	-	0.77	0.99	0.65	0.65	-0.58	0.10	-1.52	-1.29
43	-	0.66	0.46	0.65	0.65	**-2.05**	-0.24	**-3.10**	-1.80
44	-	-0.30	-0.43	0.65	0.48	-0.02	0.09	-0.32	-0.35
45	-	0.08	-0.83	0.46	0.65	-1.32	-0.18	-1.16	-1.53
46	-	-0.39	-1.33	0.27	0.65	-1.74	-0.90	**-4.87**	**-4.21**
47	-	**-3.40**	**-3.01**	-0.28	-0.73	-0.68	-0.21	-0.33	-1.88
48	20 µM, some 10µM	**-21.76**	**-19.09**	**-37.11**	**-26.25**	-1.48	**-9.44**	**-15.39**	**-16.65**
49	Hit in all replicates	**-7.07**	**-12.24**	**-5.93**	**-8.86**	**-7.24**	**-7.05**	**-16.46**	**-17.10**
50	-	0.03	0.18	0.05	0.65	0.15	0.22	0.60	-1.02
51	-	0.48	-0.64	0.65	0.65	-1.11	-1.97	**-2.48**	**-7.26**
52	-	-0.94	**-2.20**	0.19	0.46	-0.40	-0.50	**-2.73**	**-4.35**
53	-	**-3.68**	**-4.13**	0.28	-0.10	**-2.60**	-0.75	-0.96	**-4.19**
54	10 µM only	**-22.71**	**-22.95**	**-83.29**	**-81.02**	0.42	0.42	**2.27**	**-12.18**
55	Hit in all replicates	**-9.05**	**-11.95**	**-13.89**	**-9.45**	**-10.98**	**-7.32**	**-15.87**	**-16.35**
56	-	1.11	1.04	0.65	0.65	0.42	0.39	1.67	-0.36
57	-	0.78	0.54	0.65	0.65	0.42	0.33	1.34	-1.10
58	-	**-14.55**	**-12.05**	-1.74	**-3.37**	0.42	-0.61	**2.27**	**-6.24**
59	20 µM, some 10µM	**-16.23**	**-11.95**	**-9.36**	**-9.40**	0.42	**-6.00**	-0.57	**-9.32**

		Cells with altered F-actin							
		Z-score							
		10 µM				20 µM			
**Compound Number**	**Summary**	**1**	**2**	**3**	**4**	**1**	**2**	**3**	**4**
**60**	**Hit in all replicates**	**-15.72**	**-20.87**	**-27.29**	**-29.87**	**-10.87**	**-10.79**	**-11.41**	**-15.30**
**61**	-	**-10.73**	**-18.42**	0.65	**-61.39**	0.42	-0.80	**2.27**	**-15.26**
**62**	-	1.15	1.05	0.50	0.65	0.41	0.27	1.11	-0.30
**63**	-	**-7.61**	**-10.26**	0.65	-0.27	-0.36	0.42	**2.27**	**-5.18**
**64**	-	**-10.83**	**-8.78**	0.65	**-2.84**	-1.14	0.42	**2.27**	-0.79
**65**	-	**-4.27**	**-10.62**	0.65	0.65	0.42	-1.01	**2.27**	**-2.70**
**66**	-	**-4.92**	**-8.09**	0.65	0.65	0.42	0.42	**2.27**	**2.27**
**67**	**10 µM only**	**-19.30**	**-15.11**	**-9.98**	**-18.11**	0.42	-0.29	-1.70	**-4.08**
**68**	**Hit in all replicates**	**-13.61**	**-16.15**	**-10.94**	**-17.02**	**-6.20**	**-6.02**	**-12.55**	**-12.57**
**69**	**Hit in all replicates**	**-11.32**	**-15.05**	**-4.81**	**-13.48**	**-9.91**	**-9.78**	**-14.80**	**-13.68**
**70**	-	0.90	0.81	0.65	0.65	0.34	0.22	0.01	-0.63
**71**	-	**-12.18**	**-6.71**	**-5.81**	-1.11	0.42	0.42	**2.27**	**-8.45**
**72**	-	0.25	0.47	0.51	0.65	0.30	0.32	0.59	-0.56
**73**	-	**-10.21**	**-10.74**	-1.83	-0.11	0.42	0.42	0.28	**-5.44**
**74**	**Hit in all replicates**	**-13.67**	**-12.25**	**-11.87**	**-14.29**	**-30.46**	**-28.92**	**-29.76**	**-31.15**
**75**	**Hit in all replicates**	**-21.14**	**-6.10**	**-8.49**	**-4.25**	**-31.25**	**-31.25**	**-31.25**	**-22.75**
**76**	-	0.64	0.58	**-3.08**	-1.25	0.87	-0.07	-0.43	0.54
**77**	-	0.98	0.90	-1.60	-0.24	0.35	0.26	0.10	0.82

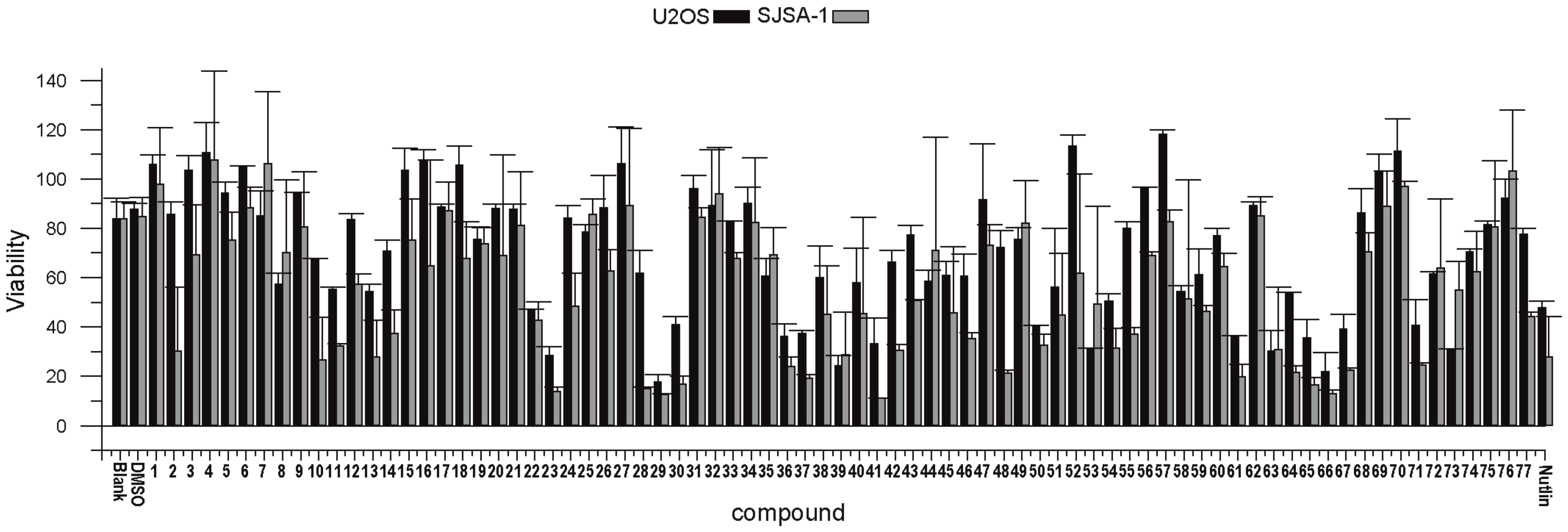

Figure S3– Cell toxicity assay data for the full compound library. Compounds are added to either U2OS or SJSA-1 cells as 50 µM stocks in serum-free media. Cells are incubated for 18 hours.

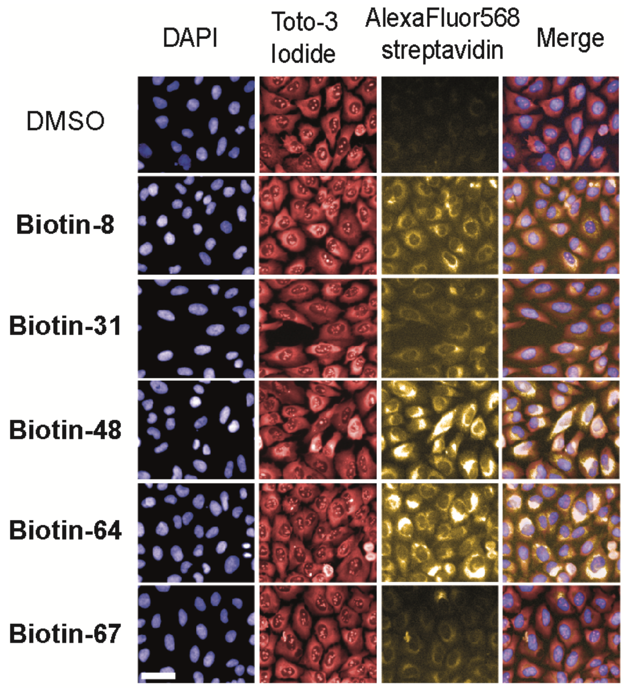

Figure S4 – cell uptake studies using biotin labelled mimetics. Cells were fixed then permeabilized with 0.1% Triton X-100 (VWR) rinsed in PBS, then incubated with AlexaFluor 568.

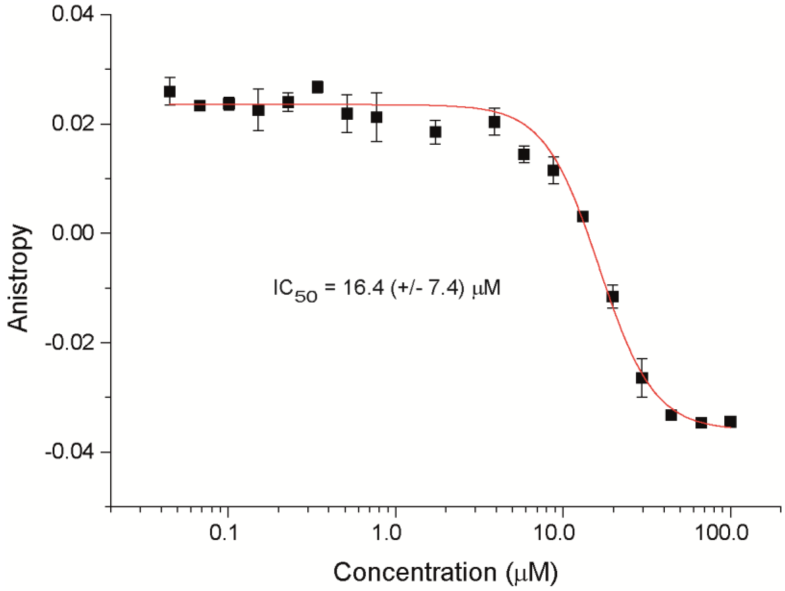

Figure S5 - Dose response curve for the inhibition of the p53/*h*DM2 interaction by mimetic **23** measured with fluorescence anisotropy. *h*DM2 and FITC-p53 were added to a dilution series of the mimetics to give final concentrations of 154.2 and 54.5 nM, respectively.

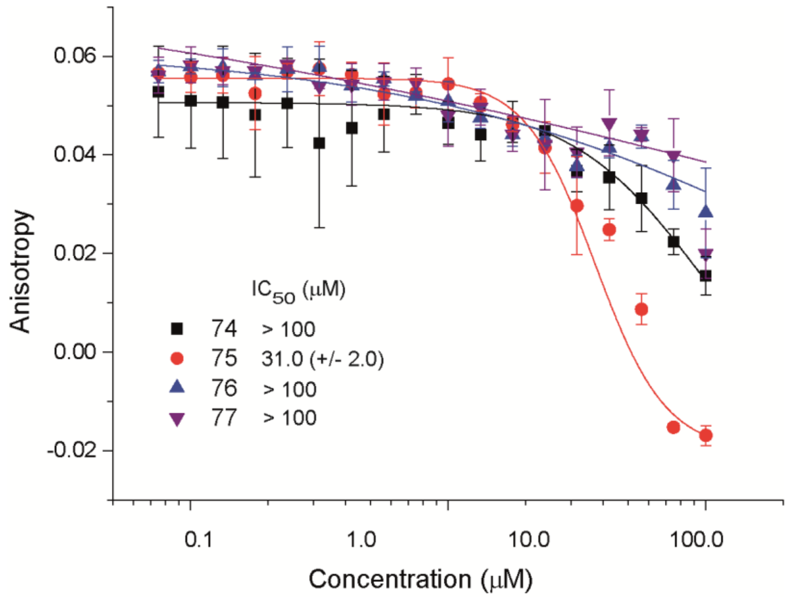

Figure S6 Dose response curve for the inhibition of the p53/*h*DM2 interaction by mimetics **74-77**measured with fluorescence anisotropy. *h*DM2 and FITC-p53 were added to a dilution series of the mimetics to give final concentrations of 154.2 and 54.5 nM, respectively.

The dimers showed interesting results in the competition assay. Three of the compounds show no inhibition up to 100 µM but one – compound **75** – disrupted the interaction with an IC_{50} of 31 µM; this is not too surprising given that 75 has a naphthyl group as the C-terminal monomer but, as the isoleucine side chain might also participate in binding it is possible that the aromatic naphthyl group effectively mimics the tryptophan on p53 resulting in the observed activity. Supporting this hypothesis, dimer 77 also contains a naphthyl but was synthesized using glycine loaded Wang resin so does not possess enough side chains to mimic all of the 'hot-spot' residues on p53

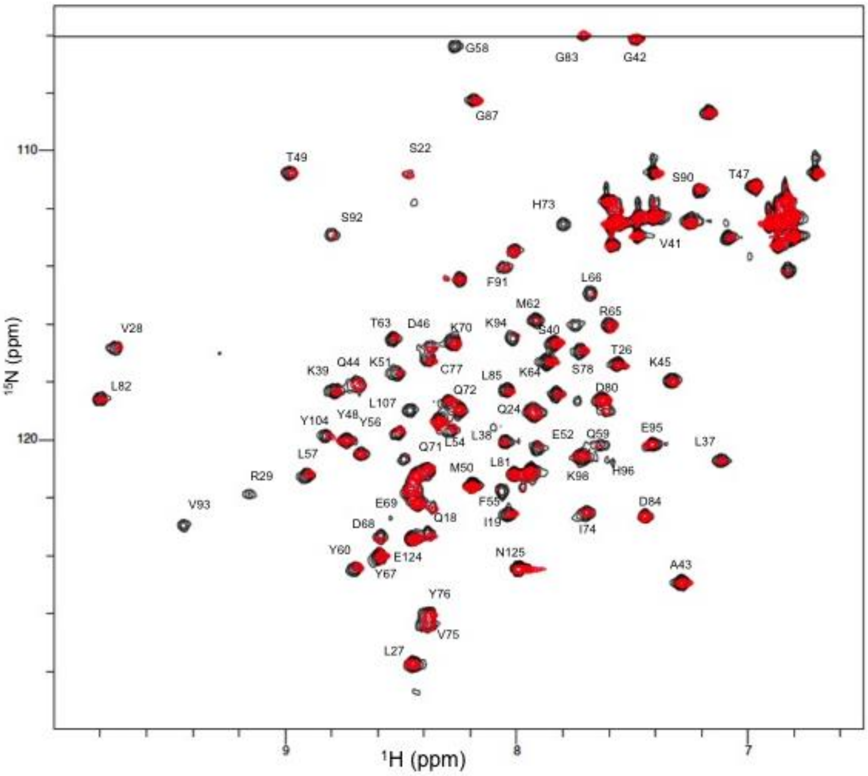

Figure S7 – ^{1}H-^{15}N HSQC of *h*DM2 in the absence and presence of Compound **64**. Black is from the protein alone, with red showing the crosspeaks upon inclusion of Compound **64**.

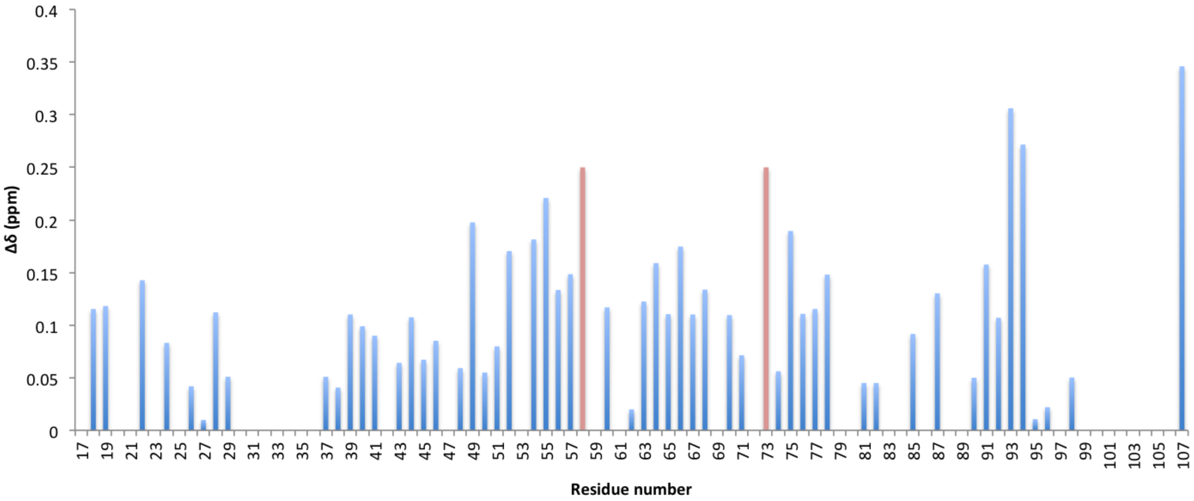

Figure S8 - Plot of chemical shifts perturbations of *h*DM2 in the presence of Compound **64**. The difference in chemical shift is shown above the residue number in blue. The crosspeaks that disappear in the presence of Compound **64** are shown in red with an arbitrary value of 0.25ppm.

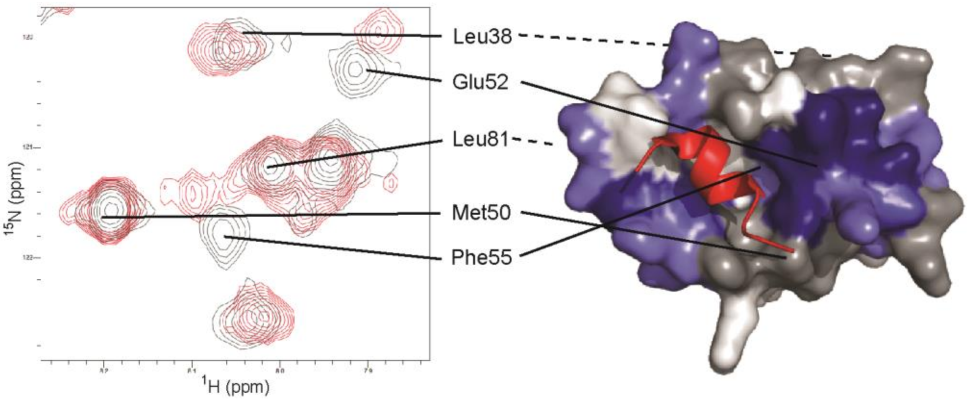

Figure S9 - ^{1}H-^{15}N HSQC of ^{15}N *h*DM2 recorded in the absence (in black) and presence (in red) of Compound **67** (cross-peaks that move or change in volume are mapped onto the surface of hDM2 and shown in blue).

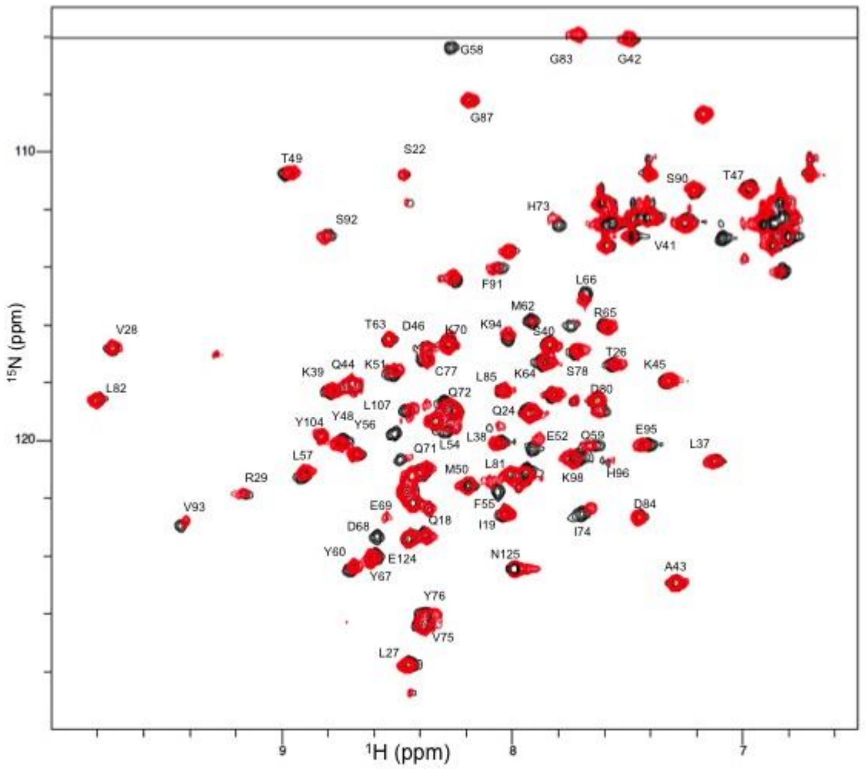

Figure S10 - ^{1}H-^{15}N HSQC of *h*DM2 in the absence and presence of Compound **67**. Black is from the protein alone, with red showing the crosspeaks upon inclusion of Compound **67**.

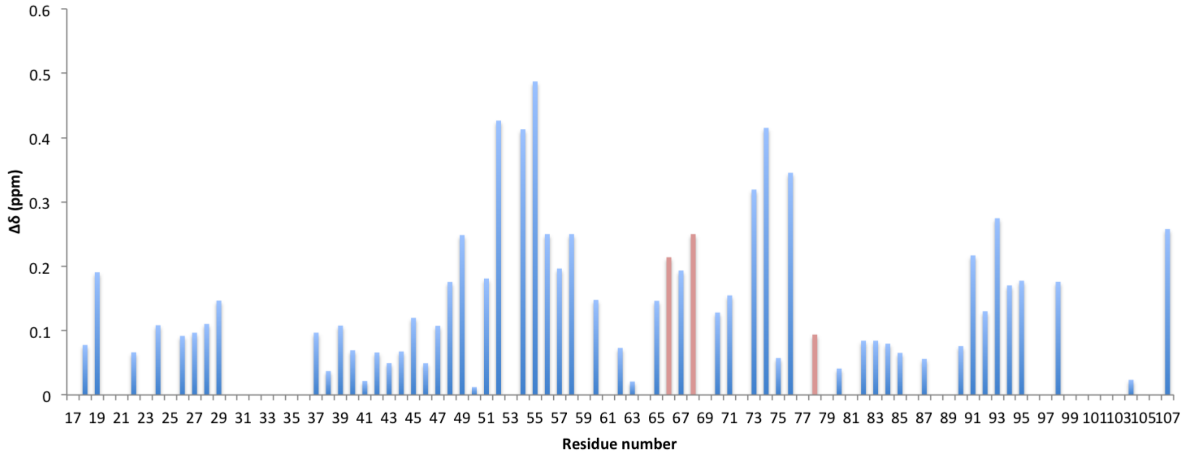

Figure S11 - Plot of chemical shifts perturbations of *h*DM2 in the presence of Compound **67**. The difference in chemical shift is shown above the residue number in blue. The crosspeaks that disappear in the presence of Compound **67** are shown in red with an arbitrary value of 0.25ppm.

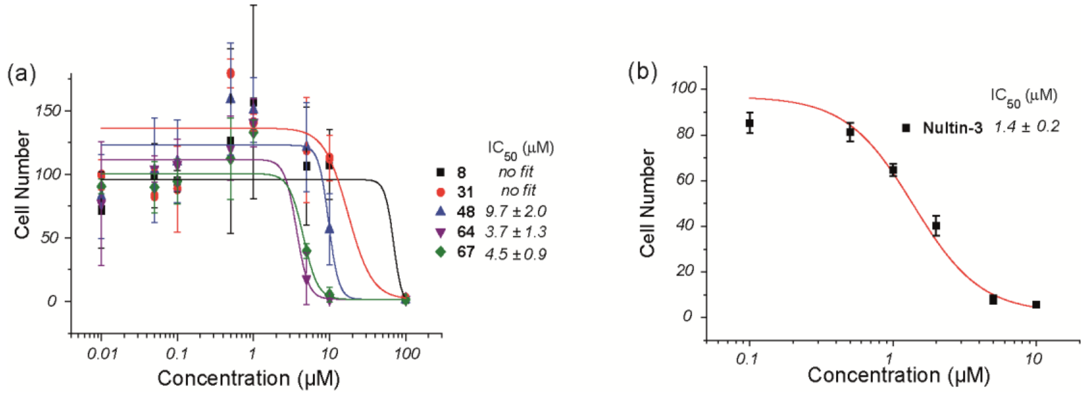

Figure S12 - Dose response curves for HCS (a) performed for selected helix mimetics in SJSA-1 cells and (b) performed for Nutlin-3 in U20S cells.

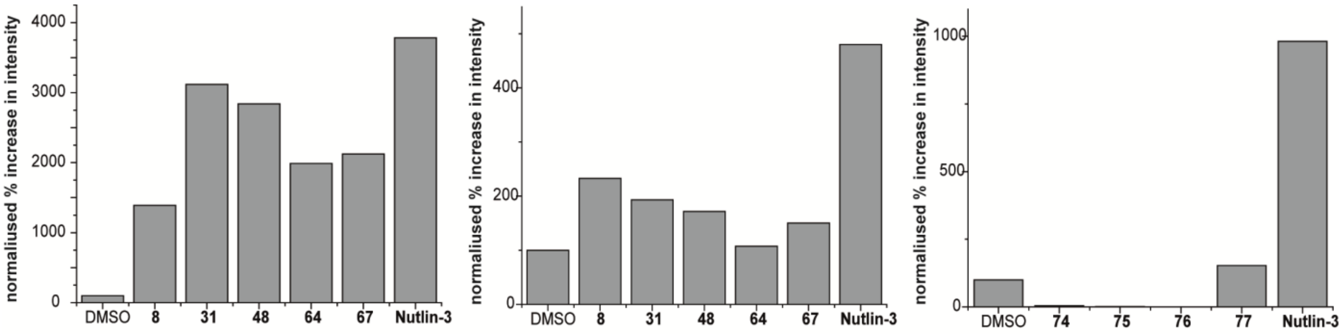

Figure S13– Densitometry analyses for p21 induction with trimers (left U2OS and right SJSA-1) and dimers (U2OS). For each gel, Image J was used to measure the intensity of the band – each band was normalized to the GAPDH control and then the entire set normalized to the DMSO control

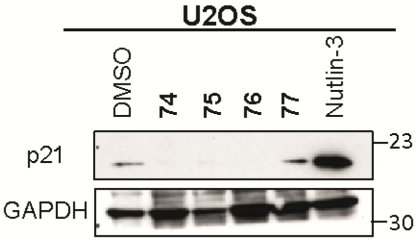

Figure S14 – U2OS cell line was incubated with mimetics (50 µM) or Nutlin-3 (10 µM) for 4 hours and lysates analysed by western blotting for p21 and GAPDH

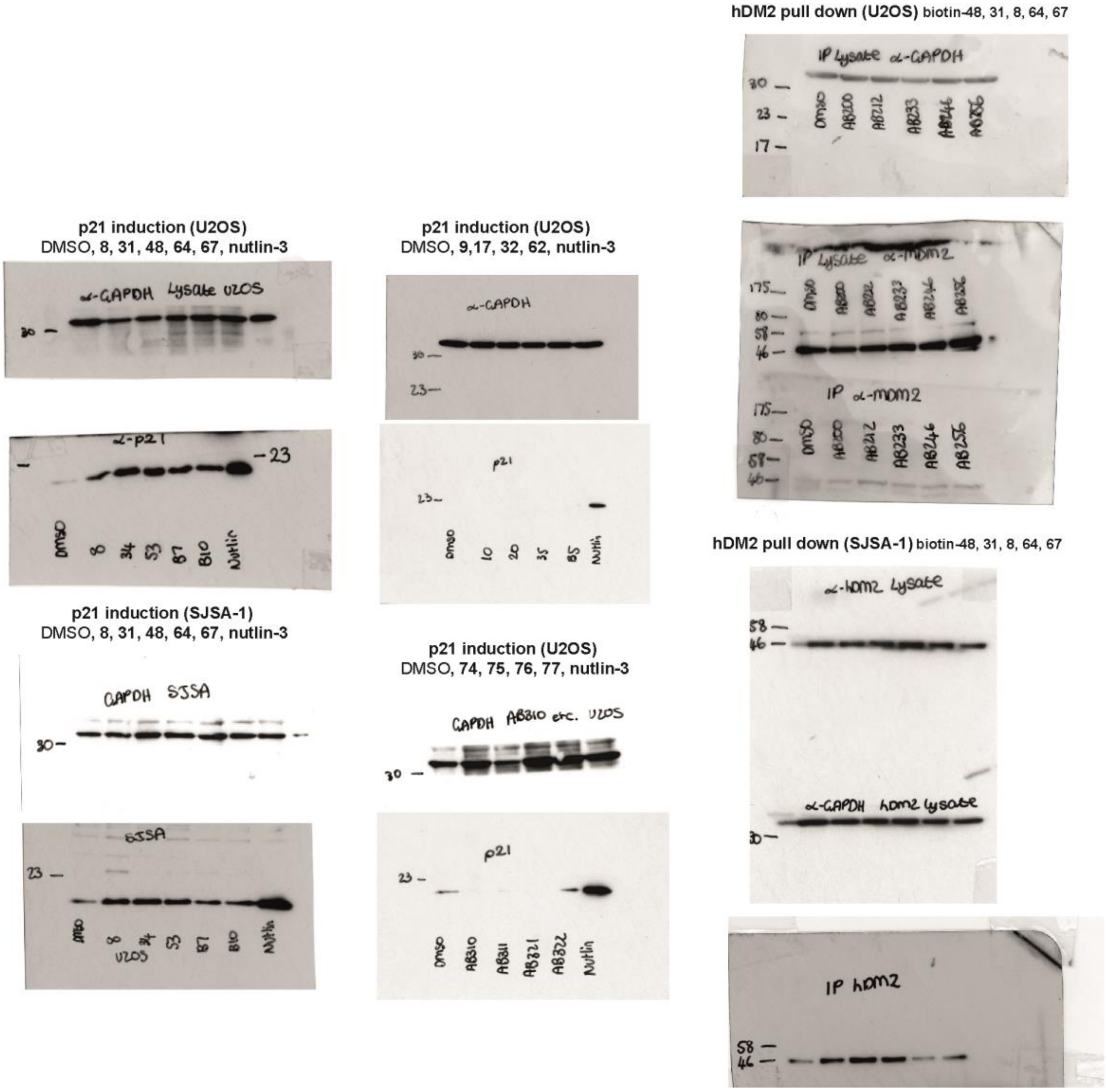

Figure S15 – Uncropped gel images from Western blotting against p21 and pull down of *h*DM2.

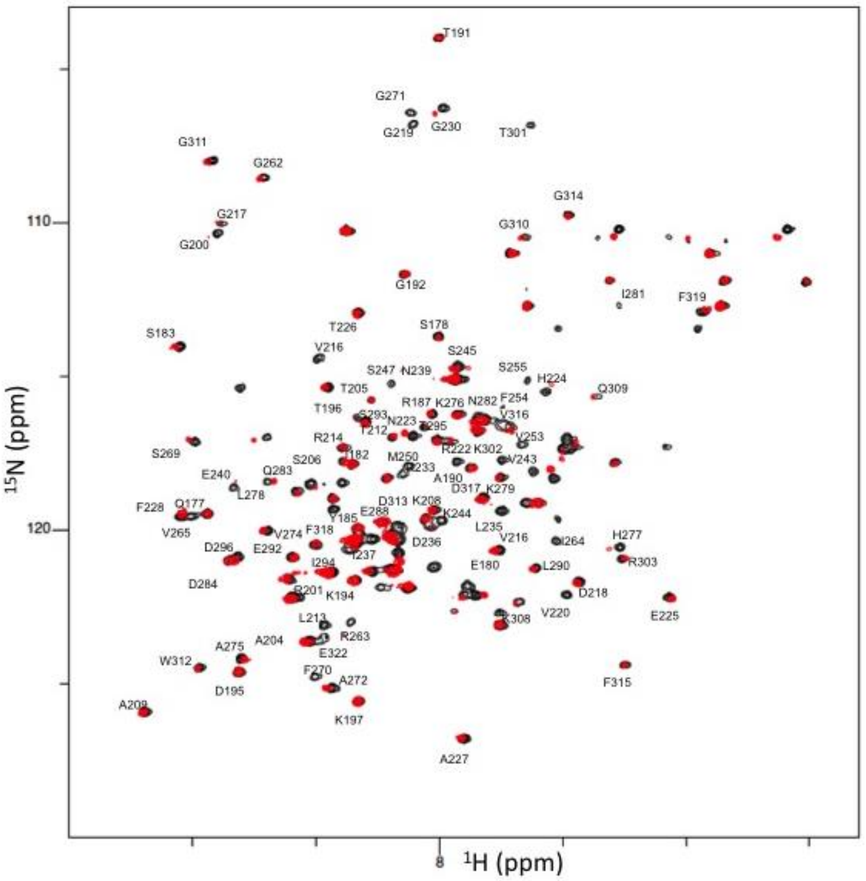

Figure S16 - ^{1}H-^{15}N HSQC of Mcl-1 in the absence and presence of Compound **64**. Black is from the protein alone, with red showing the crosspeaks upon inclusion of Compound **64**.

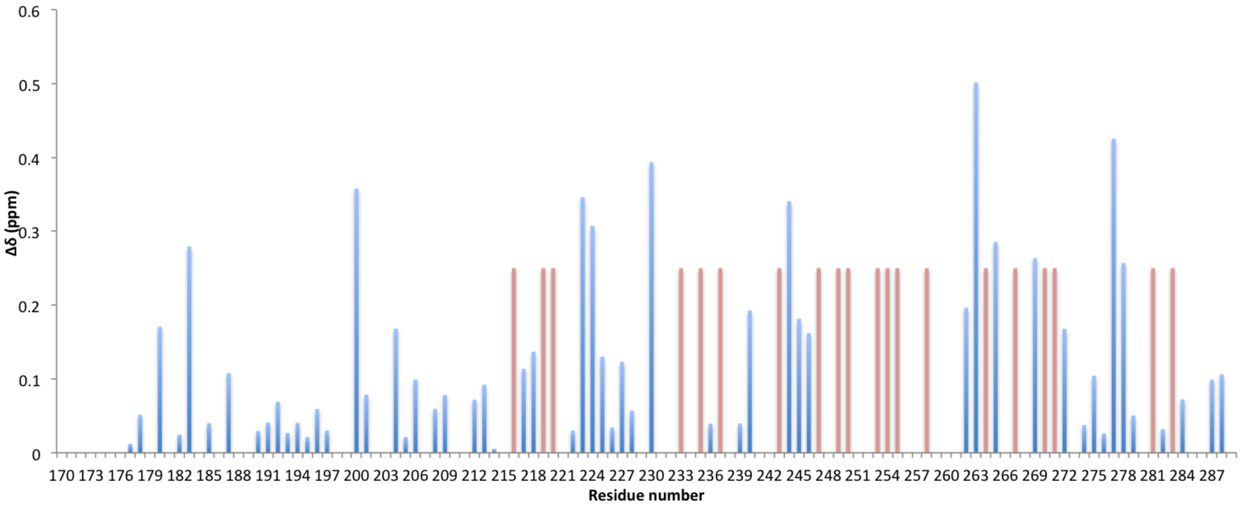

Figure S17 - Plot of chemical shifts perturbations of Mcl-1 in the presence of Compound **64**. The difference in chemical shift is shown above the residue number in blue. The crosspeaks that disappear in the presence of Compound **64** are shown in red with an arbitrary value of 0.25ppm.

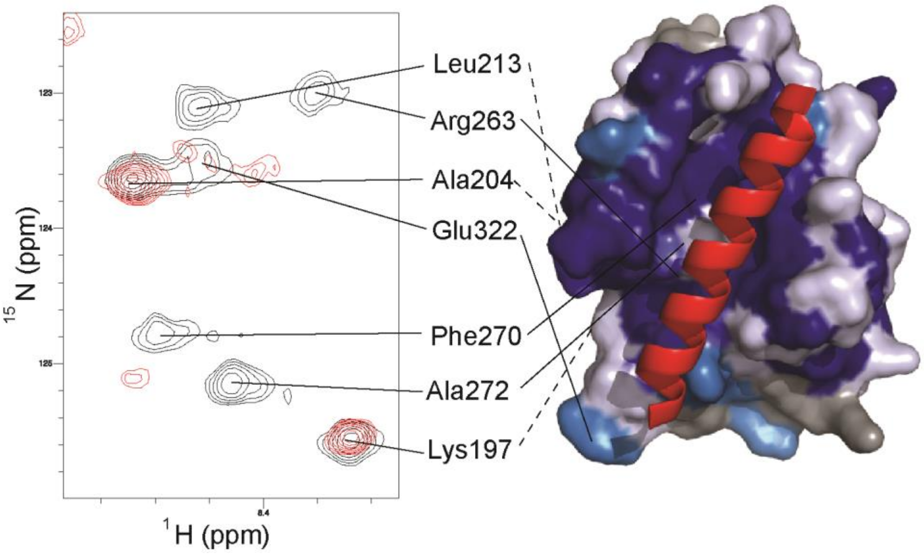

Figure S18 - ^{1}H-^{15}N HSQC of ^{15}N Mcl-1 recorded in the absence (in black) and presence (in red) of **67.** Crosspeaks that move or change in volume are mapped onto the surface of Mcl-1 and shown in blue.

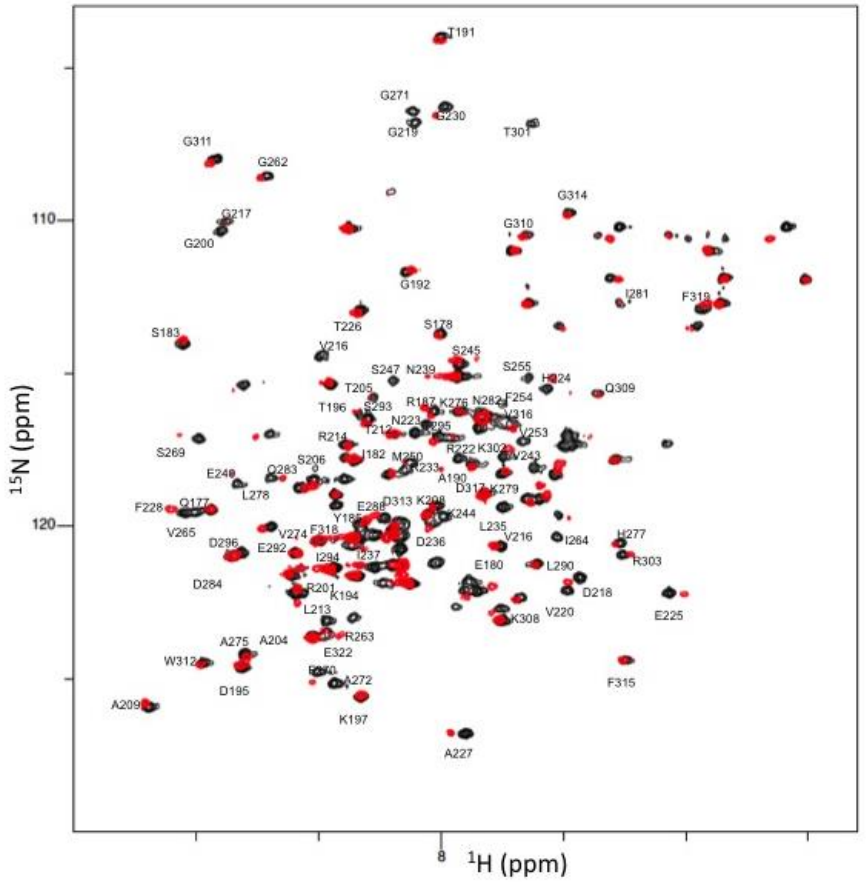

Figure S19- ^{1}H-^{15}N HSQC of Mcl-1 in the absence and presence of Compound **67**. Black is from the protein alone, with red showing the crosspeaks upon inclusion of Compound **67**.

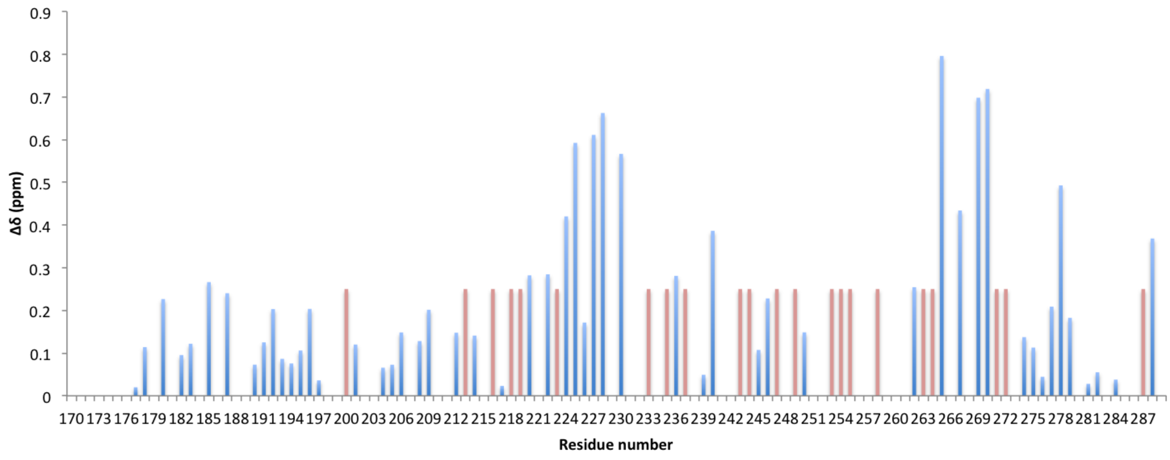

Figure S20 - Plot of chemical shifts perturbations of Mcl-1 in the presence of Compound **67**. The difference in chemical shift is shown above the residue number in blue. The crosspeaks that disappear in the presence of Compound **67** are shown in red with an arbitrary value of 0.25ppm.

Biotin pull-down (Mcl-1)
Left to right: DMSO, Biotin-48, Biotin-31, Biotin-8, Biotin-64 and Biotin-67
U2OS cells

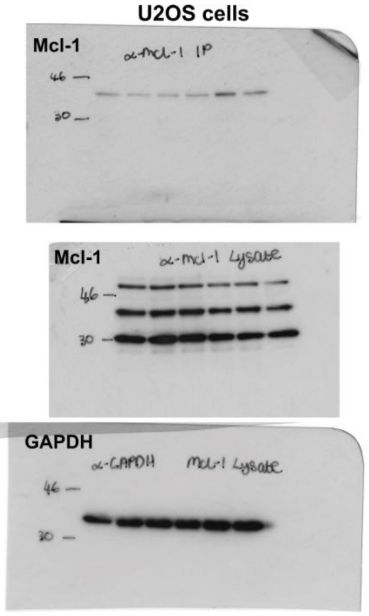

Biotin pull-down (Mcl-1)
Left to right: DMSO, Biotin-48, Biotin-31, Biotin-8, Biotin-64 and Biotin-67
Saos-2 cells

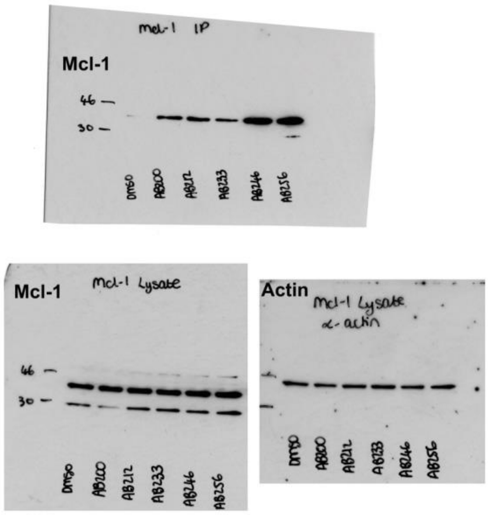

Biotin pull-down (Bcl-x$_L$)
Left to right: DMSO, Biotin-48, Biotin-31, Biotin-8, Biotin-64 and Biotin-67
U2OS cells

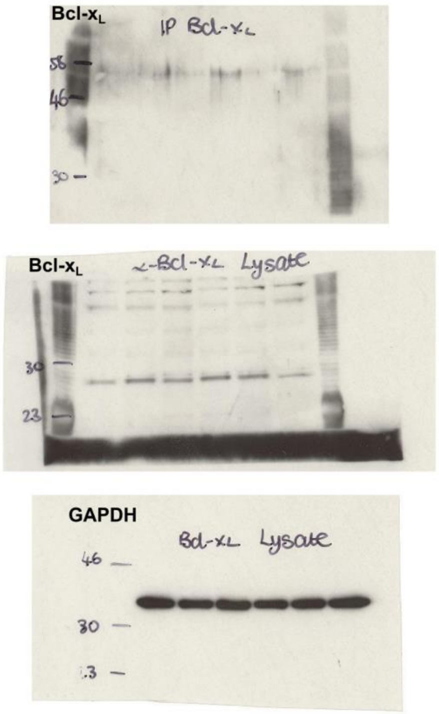

Biotin pull-down (Bcl-x$_L$)
Left to right: DMSO, Biotin-48, Biotin-31, Biotin-8, Biotin-64 and Biotin-67
Saos-2 cells

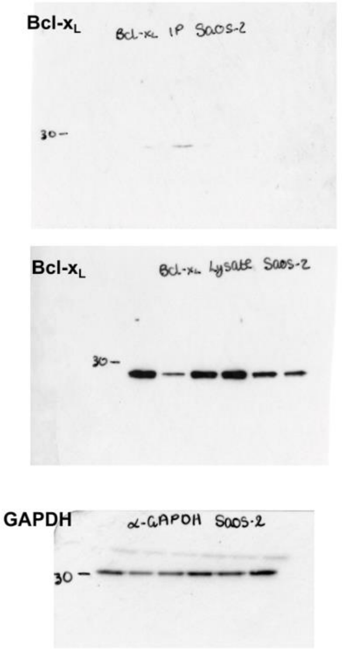

Figure S21 – Uncropped gel images from pull down with Mcl-1 and Bcl-x$_L$.

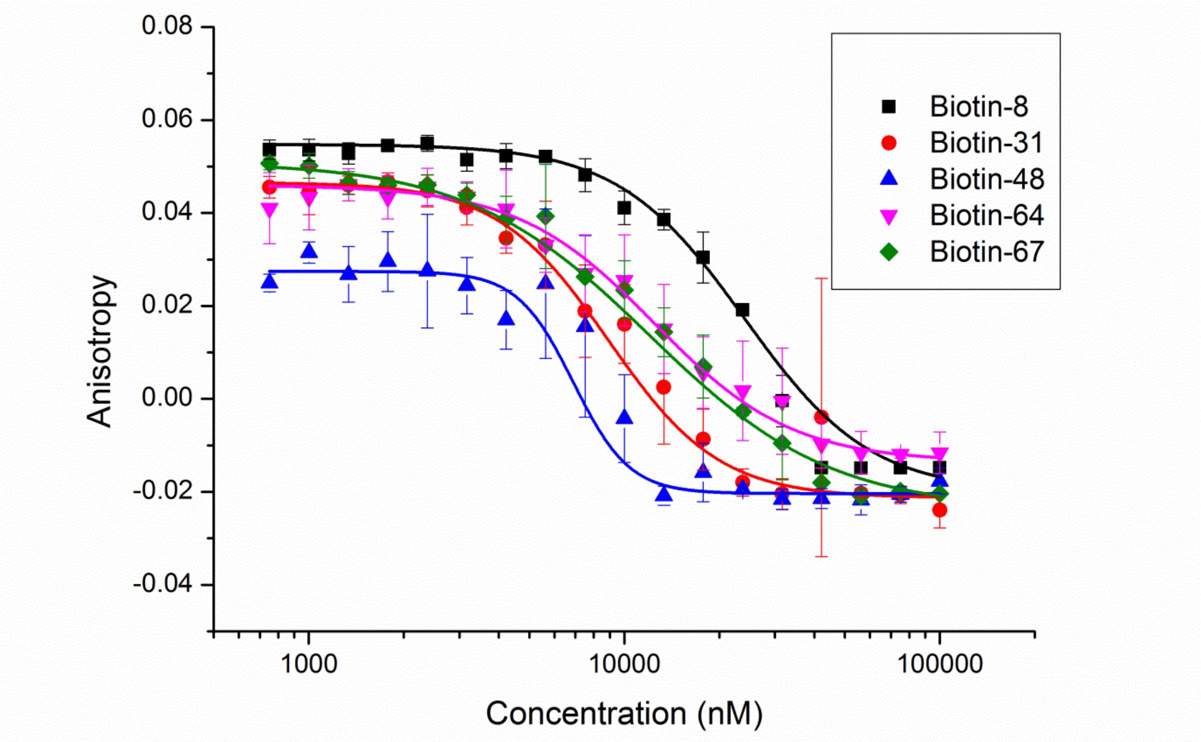

Compound Number	IC_{50}, µM
**Biotin-8**	23.2 ± 0.7
**Biotin-31**	8.9 ± 0.5
**Biotin-48**	7 ± 1
**Biotin-64**	12.5 ± 0.7
**Biotin-67**	12.1 ± 0.4

Figure S22 - Dose response curves of the inhibition of the p53/*h*DM2 interaction by the biotinylated library of helix mimetics measured with fluorescence anisotropy. *h*DM2 and FITC-p53 were added to a dilution series of the mimetics to give final concentrations of 154.2 and 54.5 nM, respectively.

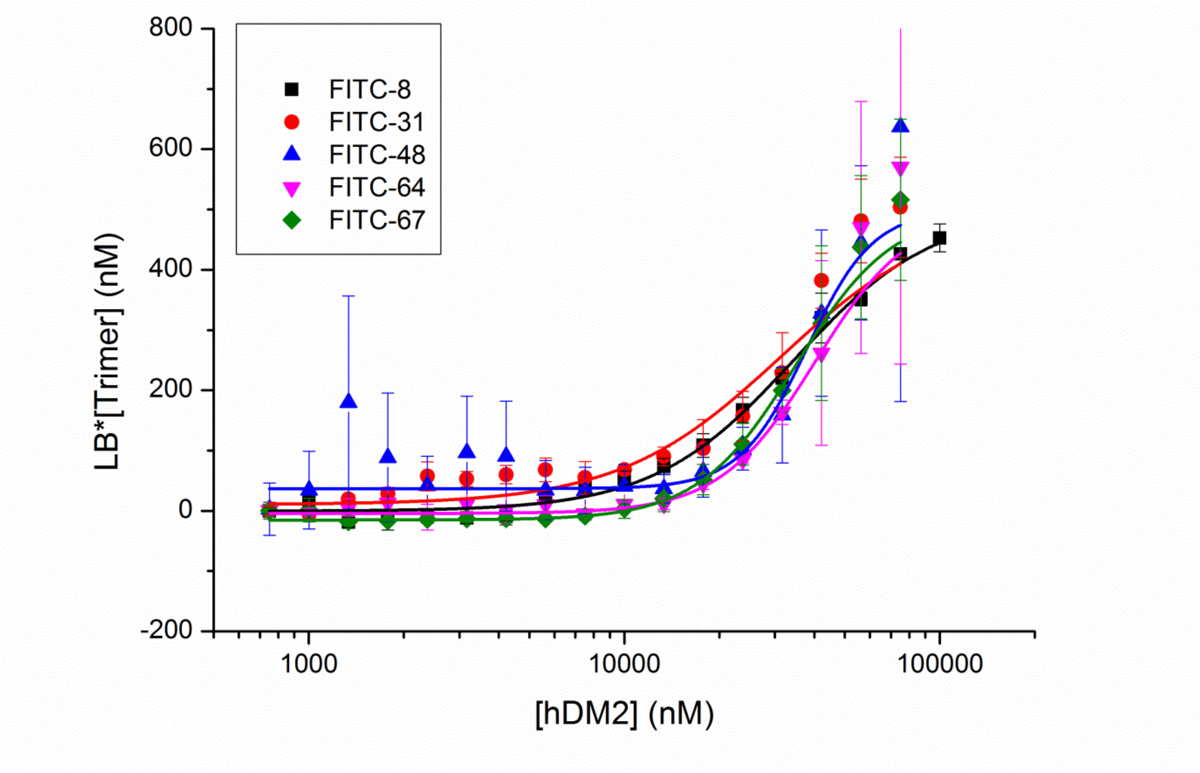

Compound Number	IC_{50}, μM
**FITC-8**	34.0 ± 1.0
**FITC-31**	31.0 ± 5.0
**FITC-48**	38.0 ± 3.0
**FITC-64**	40.0 ± 3.0
**FITC-67**	35.0 ± 2.0

Figure S23 – Dose response curves for the direct binding of the FITC-labelled trimers to *h*DM2 measured using fluorescence anisotropy. FITC trimers were fixed at a concentration of 500 nM.

Table S2a - Summary of the results from the high-content assays for conjugated helix mimetics. U2OS cells were treated with 2 different concentrations of the helix mimetics (10 and 20 µM) and cell number assessed

		Cell Number							
		Z-score							
		10 µM				20 µM			
**Compound Number**	**Summary**	**1**	**2**	**3**	**4**	**1**	**2**	**3**	**4**
**Biotin-8**	-	0.50	1.75	-0.18	-0.59	1.89	1.48	-0.34	0.37
**Biotin-31**	-	0.60	1.38	0.33	-0.81	1.46	1.50	-0.04	0.06
**Biotin-48**	-	0.38	1.18	1.21	-0.15	1.49	**2.82**	-0.42	0.01
**Biotin-64**	-	-0.04	1.29	0.29	-0.36	0.86	0.84	0.00	-0.40
**Biotin-67**	-	-0.66	0.60	-0.06	0.37	1.37	0.12	-0.73	-0.74
**FITC-8**	-	-0.57	-0.56	-0.35	0.10	-0.47	**-2.16**	-1.59	**-2.25**
**FITC-31**	**20 µM, some 10µM**	-1.15	**-2.26**	-1.09	0.62	**-2.58**	**-3.40**	**-3.49**	**-3.96**
**FITC-48**	-	-0.30	-1.05	-0.04	0.48	-1.14	-1.50	-1.11	-1.01
**FITC-64**	**20 µM only**	-0.21	-0.31	-0.24	0.58	**-2.61**	**-2.88**	**-3.05**	**-2.99**
**FITC-67**	**20 µM only**	-0.69	-0.83	-0.78	-0.14	**-2.39**	**-2.58**	**-3.39**	**-3.37**

Table S2b - Summary of the results from the high-content assays for conjugated helix mimetics. U2OS cells were treated with 2 different concentrations of the helix mimetics (10 and 20 µM) and active for Caspase-3 quantified

		Caspase-3 positive cells							
		Z-score							
		10 µM				20 µM			
**Compound Number**	**Summary**	**1**	**2**	**3**	**4**	**1**	**2**	**3**	**4**
**Biotin-8**	**-**	-1.08	1.22	-0.55	-0.48	0.71	-1.00	-1.03	-1.22
**Biotin-31**	**-**	-1.09	0.62	-0.78	-1.00	-0.39	-0.64	-0.58	-0.90
**Biotin-48**	**-**	-0.64	**9.13**	-0.60	-1.21	-1.25	-0.45	-0.68	-0.90
**Biotin-64**	**-**	-1.37	0.65	-0.62	-0.69	-0.29	0.26	-1.21	-1.03
**Biotin-67**	**-**	-0.65	0.04	-0.73	0.24	0.75	-1.37	-0.82	-0.64
**FITC-8**	**-**	-0.54	-0.32	0.86	0.64	**24.98**	1.28	**3.68**	**2.73**
**FITC-31**	**-**	0.14	1.13	1.83	**6.05**	**30.11**	**5.45**	**8.76**	0.66
**FITC-48**	**-**	0.47	0.96	1.64	**10.68**	**10.01**	0.29	0.08	-0.24
**FITC-64**	**-**	0.09	-0.22	-0.39	**2.97**	**5.91**	**3.18**	**4.22**	0.17
**FITC-67**	**-**	-0.45	-0.30	-0.63	-0.27	**5.14**	1.34	-0.01	**3.04**

Table S2c- Summary of the results from the high-content assays for conjugated helix mimetics. U2OS cells were treated with 2 different concentrations of the helix mimetics (10 and 20 µM) and autophagic cells quantified

		**Autophagic cells**							
		**Z-score**							
		**10 µM**				**20 µM**			
**Compound Number**	**Summary**	**Rep 1**	**Rep 2**	**Rep 3**	**Rep 4**	**Rep 1**	**Rep 2**	**Rep 3**	**Rep 4**
**Biotin-8**	**Hit in all replicates**	**3.72**	**2.43**	**7.64**	**6.27**	**11.75**	**13.14**	**15.18**	**7.34**
**Biotin-31**	**20 µM, some 10µM**	**6.98**	1.84	**10.58**	**4.16**	**16.23**	**15.16**	**22.61**	**9.12**
**Biotin-48**	**20 µM, some 10µM**	0.70	**15.00**	-0.22	1.71	**17.00**	**28.16**	**12.94**	**7.99**
**Biotin-64**	**20 µM, some 10µM**	1.45	**2.32**	**2.26**	**3.59**	**13.31**	**17.19**	**5.52**	**3.25**
**Biotin-67**	-	-1.95	-1.48	-0.70	-0.63	0.16	0.48	-0.51	0.53
**FITC-8**	**Not measured**	-	-	-	-	-	-	-	-
**FITC-31**	**Not measured**	-	-	-	-	-	-	-	-
**FITC-48**	**Not measured**	-	-	-	-	-	-	-	-
**FITC-64**	**Not measured**	-	-	-	-	-	-	-	-
**FITC-67**	**Not measured**	-	-	-	-	-	-	-	-

Table S2d - Summary of the results from the high-content assays for conjugated helix mimetics. U2OS cells were treated with 2 different concentrations of the helix mimetics (10 and 20 µM) and cells with altered F-actin quantified.

		Cells with altered F-actin							
		Z-score							
		10 µM				20 µM			
**Compound Number**	**Summary**	**1**	**2**	**3**	**4**	**1**	**2**	**3**	**4**
**Biotin-8**	**-**	0.40	-0.47	0.71	0.23	-0.07	-1.48	0.75	-0.19
**Biotin-31**	**-**	-0.48	-0.52	0.95	0.10	-0.08	-0.97	0.50	-0.87
**Biotin-48**	**-**	0.49	**-2.16**	0.75	-0.09	0.69	-0.94	1.07	-0.27
**Biotin-64**	**-**	0.62	0.01	0.76	-0.19	-1.25	-0.16	0.58	-0.05
**Biotin-67**	**-**	0.07	1.24	1.20	0.08	-0.48	0.32	0.57	-0.94
**FITC-8**	**Not measured**	**-**	**-**	**-**	**-**	**-**	**-**	**-**	**-**
**FITC-31**	**Not measured**	**-**	**-**	**-**	**-**	**-**	**-**	**-**	**-**
**FITC-48**	**Not measured**	**-**	**-**	**-**	**-**	**-**	**-**	**-**	**-**
**FITC-64**	**Not measured**	**-**	**-**	**-**	**-**	**-**	**-**	**-**	**-**
**FITC-67**	**Not measured**	**-**	**-**	**-**	**-**	**-**	**-**	**-**	**-**

Synthetic Procedures and Characterisation

Monomers

Fmoc-protected monomers **a**, **c**, **k**, **m**, **o**, and **s**;[6] **e**, **f**, **g**, **h**, **l**, **n**, **q**, **r** and **t**;[1] **j** and **u**[4] were synthesised as described previously.

4-((((9H-Fluoren-9-yl)methoxy)carbonyl)(4-((trifluoromethyl)benzyl)amino))benzoic acid b

4-Aminobenzoic acid (3.00 g, 21.9 mmol), 4-trifluoromethylbenzaldehyde (3.00 mL, 21.9 mmol) and picoline-borane complex (2.57 g, 24.1 mmol) were stirred at room temperature in methanol (20 mL) for 14 hours. A white precipitate formed. The reaction mixture was filtered, and the filtrate acidified with 1N hydrochloric acid (~15 mL) to induce further precipitation. The combined precipitates were dried under high vacuum to yield 4-((4-(trifluoromethyl)benzyl)amino)benzoic acid (3.91 g, 61%) as a colourless powder; R_F 0.40 (10% methanol in dichloromethane); m.p. 181-182 ^oC; δ_H (500 MHz, $CDCl_3$) 4.51 (s, 2H, Hα), 6.49 (d, 2H, *J* = 9.1 Hz, H3), 7.47 (d, 2H, *J* = 7.5 Hz, HAr2), 7.63 (d, 2H, *J* = 7.5 Hz, HAr3), 7.92 (d, 2H, *J* = 9.1 Hz, H2); δ_C (125 MHz, $CDCl_3$) 50.19, 115.46, 121.82, 129.32, 130.51, 131.55, 133.14 (q), 135.69, 148.53, 156.98, 173.62; ν_{max}/cm^{-1} (solid state) = 3414 (NH), ~3000 (COOH), 1690 (CO), 1120-1180 (CF_3); ESI-MS *m/z* 296 $[M+H]^+$; ESI-HRMS found *m/z* 294.0755 $[M-H]^-$ $C_{15}H_{11}F_3NO_2$ requires 294.0747.

To a refluxing solution of 4-((4-(trifluoromethyl)benzyl)amino)benzoic acid (3.91 g, 13.3 mmol) in tetrahydrofuran (60 mL) was added dropwise a solution of Fmoc-Cl (3.78 g, 14.7 mmol) in tetrahydrofuran (20 mL). The reaction mixture was stirred at reflux for 14 hours. The solvents were evaporated and the residue was crystallised (tetrahydrofuran - dichloromethane) to yield the target compound (6.50 g, 91%) as yellow crystals; R_F 0.37 (ethyl acetate - hexane 1-1); m.p. 144-145 ^oC; (Found: C, 69.3; H, 4.3; N, 2.6; $C_{30}H_{22}F_3NO_4$ requires: C, 69.6; H, 4.2; N, 2.7%); δ_H (500 MHz, $CDCl_3$) 4.11 (t, 1H, *J* = 5.1 Hz, FHβ), 4.64 (d, 2H, *J* = 5.1 Hz, FHα), 4.82 (s, 2H, Hα), 7.06 (d, 2H, *J* = 8.4 Hz, H3), 7.14 (d, 2H, *J* = 7.9 Hz, HAr2/3), 7.21 (t, 2H, *J* = 7.4 Hz, FH4), 7.30 (d, 2H, *J* = 7.4 Hz, FH5), 7.37 (t, 2H, *J* = 7.5 Hz, FH3), 7.50 (d, 2H, *J* = 7.9 Hz, HAr3/2), 7.69 (d, 2H, *J* = 7.5, FH2), 7.97 (d, 2H, *J* = 8.4, H2); δ_C (125 MHz, $CDCl_3$) 47.58, 53.76, 67.92, 120.40, 125.09, 126.00, 126.05, 126.58, 127.45, 128.02, 128.16, 129.95 (q), 130.35, 131.54, 141.54, 141.79, 143.83, 146.75, 155.42, 171.68; ν_{max}/cm^{-1} (solid state) = ~3000 (COOH), 1710 (CO), 1230-1120 (CF_3); ESI-MS *m/z* 540 $[M+Na]^+$; ESI-HRMS found 516.1435 *m/z* $[M-H]^-$ $C_{30}H_{21}F_3NO_4$ requires 516.1428.

4-((((9H-Fluoren-9-yl)methoxy)carbonyl)((2-methylnaphthalen-1-yl)methyl)amino)benzoic acid d

4-Aminobenzoic acid (0.81 g, 5.9 mmol), 2-methyl-1-naphthaldehyde (1.00 g, 5.9 mmol) and picoline-borane complex (0.72 g, 6.8 mmol) were stirred at room temperature in methanol (20 mL)

for 14 hours. A white precipitate formed. The reaction mixture was filtered, and the filtrate acidified with 1N hydrochloric acid (~10 mL) to induce further precipitation. The combined precipitates were dried under high vacuum to yield 4-(((2-methylnaphthalen-1-yl)methyl)amino)benzoic acid (1.60 g, 94%) as a colourless powder; R_F 0.65 (10% methanol in dichloromethane); m.p. 209-211 °C; δ_H (300 MHz, DMSO-d_6) 2.52 (s, 3H, CH_3), 4.61 (s, 2H, Hα), 6.55 (br s, 1H, NH), 6.78 (d, 2H, *J* = 8.9 Hz, H3), 7.43 (d, 1H, *J* = 8.4 Hz, HAr3), 7.46-7.56 (m, 2H, HAr6-HAr7), 7.73 (d, 2H, *J* = 8.9 Hz, H2), 7.85 (d, 1H, *J* = 8.4 Hz, HAr4), 7.92 (d, 1H, *J* = 7.8 Hz, HAr5/8), 7.97 (d, 1H, *J* = 8.4 Hz, HAr8/5); δ_C (75 MHz, DMSO-d_6) 19.92, 40.64, 111.27, 117.36, 124.04, 125.23, 126.86, 128.14, 128.62, 129.43 ,131.01, 131.45, 132.38, 135.37, 153.07, 167.94; ν_{max}/cm^{-1} (solid state) = 3373 (NH), ~3000 (COOH), 1738 (CO); ESI-MS *m/z* 314 $[M+Na]^+$; ESI-HRMS found *m/z* 292.1332 $[M+H]^+$ $C_{19}H_{18}NO_2$ requires 292.1327.

To a refluxing solution of 4-(((2-methylnaphthalen-1-yl)methyl)amino)benzoic acid (1.60 g, 5.5 mmol) in tetrahydrofuran (10 mL) was added dropwise a solution of Fmoc-Cl (1.50 g, 5.8 mmol) in tetrahydrofuran (10 mL). The reaction mixture was stirred at reflux for 15 hours. The solvents were evaporated and the residue crystallized from a mixture of tetrahydrofuran – hexane to yield the target compound as pale yellow crystals (2.43 g, 83%); R_F 0.53 (10% methanol in dichloromethane); m.p. 183-185 °C; δ_H (500 MHz, $CDCl_3$) 2.07 (s, 3H, CH_3), 4.03 (t, 1H, *J* = 6.5 Hz, FHβ), 4.49 (d, 2H, *J* = 6.5 Hz, FHα), 5.38 (s, 2H, Hα), 6.70 (d, 2H, *J* = 8.5 Hz, H3), 7.05 (d, 1H, *J* = 8.5 Hz, ArCH), 7.11-7.14 (m, 4H, ArCH), 7.27-7.31 (m, 2H, ArCH), 7.41 (t, 1H, *J* = 7.0 Hz, FH3/4), 7.48-7.51 (m, 1H, ArCH), 7.60-7.64 (m, 3H, ArCH), 7.75 (d, 1H, *J* = 8.0 Hz, ArCH), 7.80 (d, 2H, *J* = 8.5 Hz, H2), 8.15 (d, 1H, *J* = 8.5 Hz, ArCH); δ_C (125 MHz, $CDCl_3$) 19.97, 46.21, 47.06, 67.76, 119.87, 119.97, 123.78, 124.86, 125.02, 126.93, 127.63, 128.17, 128.55, 128.63, 128.85, 130.67, 132.44, 132.65, 135.92, 141.29, 143.60, 144.96, 155.22, 171.25; ν_{max}/cm^{-1} (solid state) = ~3000 (COOH), 1738 (CO); ESI-MS *m/z* 536 $[M+Na]^+$; ESI-HRMS found *m/z* 512.1879 $[M-H]^-$ $C_{34}H_{26}NO_4$ requires 512.1867.

4-((((9H-Fluoren-9-yl)methoxy)carbonyl)(3-fluorobenzylamino)benzoic acid i

4-Aminobenzoic acid (3.88 g, 28.3 mmol), 3-fluorobenzaldehyde (3.0 mL, 28.3 mmol) and picoline-borane complex (3.33 g, 31.1 mmol) were stirred at room temperature in methanol (90 mL) for 14 hours. A white precipitate formed. The reaction mixture was filtered, and the filtrate acidified (~pH 4) with 1N hydrochloric acid then left to crystallise to yield 4-(3-fluorobenzylamino)benzoic acid (4.10 g, 59%) as colourless crystals; R_F 0.37 (40% hexane in ethyl acetate); m.p. 170-172 °C; (Found: C, 68.20; H, 4.95; N, 5.75%; $C_{14}H_{12}FNO_2$ requires: C, 68.56; H, 4.93; N, 5.71%); δ_H (300 MHz, DMSO-d_6) 4.36 (d, 2H, *J* = 6.1 Hz, Hα), 6.59 (d, 2H, *J* = 8.9 Hz, H3), 7.03-7.20 (m, 4H, NH, HAr2, HAr3, HAr5), 7.34-7.41 (m, 1H, HAr4), 7.66 (d, 2H, *J* = 8.9 Hz, H2); δ_C (75 MHz, DMSO-d_6) 45.05, 111.15, 114.92, 115.20, 117.21, 128.97, 131.04, 135.51, 152.21, 167.41; ν_{max}/cm^{-1} (solid state) = 3427 (NH), ~3002 (COOH),

1736 (CO), 1365 (C-N), 1216 (C-O); ESI-HRMS found m/z 244.0788 [M-H]$^-$ $C_{14}H_{11}FNO_2$ requires 244.0779.

To a refluxing solution of 4-(3-fluorobenzylamino)benzoic acid (2.61 g, 10.6 mmol) in tetrahydrofuran (40 mL) was added dropwise a solution of Fmoc-chloride (2.89 g, 11.2 mmol) in tetrahydrofuran (10 mL). The reaction mixture was then stirred at reflux overnight. The solvents were evaporated, then the residue was purified by column chromatography (*Stationary phase:* silica, *Mobile phase:* dichloromethane–hexane 1-1 then neat dichloromethane then 3% methanol in dichloromethane) to afford the target material as a white solid (3.41 g, 69%); R_F 0.45 (7% methanol in dichloromethane); (Found: C, 73.75; H, 4.75; N, 2.95%; $C_{29}H_{22}FNO_4$ requires: C, 74.51; H, 4.74; N, 3.00%); δ_H (300 MHz, $CDCl_3$) 4.12 (t, 1H, J = 5.8 Hz, FHβ), 4.60 (d, 2H, J = 5.8 Hz, FHα), 4.80 (s, 2H, Hα), 6.84 (m, 2H, HAr4, HAr6), 6.93 (m, 1H, HAr5), 7.07 (d, 2H, J = 8.6 Hz, H3), 7.18-7.24 (m, 3H, HAr2, FH4), 7.29 (d, 2H, J = 7.2 Hz, FH5), 7.36 (t, 2H, J = 7.5 Hz, FH3), 7.70 (d, 2H, J = 7.5 Hz, FH2), 7.98 (d, 2H, J = 8.6 Hz, H2); δ_C (125 MHz, $CDCl_3$) 47.17, 53.29, 67.63, 114.45, 114.62, 119.97, 124.74, 126.38, 127.04, 127.74, 130.14, 130.21, 131.07, 139.70, 141.38, 143.50, 146.42, 166.07, 161.95, 163.91, 171.07; ν_{max}/cm^{-1} (solid state) = ~3016 (COOH), 1739 (CO), 1365 (C-N), 1216 (C-O); ESI-HRMS found *m/z* 466.1476 [M-H]$^-$ $C_{29}H_{22}FNO_4$ requires 466.1460.

4-((((9H-Fluoren-9-yl)methoxy)carbonyl)(cyclohexylmethyl)amino)benzoic acid p

4-Aminobenzoic acid (1.50 g, 10.9 mmol), cyclohexanaldehyde (1.3 mL, 10.9 mmol) and picoline-borane complex (1.35 g, 12.0 mmol) were stirred at room temperature in methanol (60 mL) for 14 hours. A white precipitate formed. The reaction mixture was filtered, and the filtrate acidified with 1N hydrochloric acid (~ pH 4) to induce further precipitation. The combined precipitates were dried under high vacuum to yield 4-(cyclohexylmethylamino)benzoic acid (2.09 g, 82%) as a white powder; R_F 0.48 (40% hexane in ethyl acetate); (Found: C, 71.90; H, 8.25; N, 6.00%; $C_{14}H_{19}NO_2$ requires: C, 72.07; H, 8.21; N, 6.00%); δ_H (500 MHz, MeOD) 1.03 (dd, 2H, J_1 = 11.7 Hz, J_2 = 2.6 Hz, Hγ), 1.21-1.36 (m, 3H, Hδ, Hε), 1.63 (m, 1H, Hβ), 1.72 (d, 1H, J = 11.1 Hz, Hε’), 1.78 (d, 2H, J = 12.9 Hz, Hδ’), 1.87 (d, 2H, J = 11.7 Hz, Hγ’), 3.01 (d, 2H, J = 6.8 Hz, Hα), 6.59 (d, 2H, J = 8.9 Hz, H3), 7.79 (d, 2H, J = 8.9 Hz, H2); δ_C (75 MHz, MeOD) 27.15, 27.71, 32.31, 38.70, 50.70, 111.98, 117.55, 132.77, 154.83, 170.87; ν_{max}/cm^{-1} (solid state) = 3417 (NH), ~3015 (COOH), 1738 (CO), 1365 (C-N), 1217 (C-O); ESI-MS *m/z* 232 [M-H]$^-$; ESI-HRMS found *m/z* 234.1484 [M+H]$^+$ $C_{14}H_{20}NO_2$ requires 234.1489.

To a refluxing solution of 4-(cyclohexylmethylamino)benzoic acid (2.09 g, 8.9 mmol) in tetrahydrofuran (30 mL) was added dropwise a solution of Fmoc-chloride (2.65 g, 9.9 mmol) in tetrahydrofuran (5 mL). The reaction mixture was then stirred at reflux overnight. The solvents were evaporated, then the residue was purified by column chromatography (*Stationary phase :* silica;

Mobile phase : ethyl acetate–hexane 2-3 to neat ethyl acetate) then crystallised from dichloromethane–hexane to yield the pure target material (2.33 g, 57%) as colourless platelets; R_F 0.25 (ethyl acetate–hexane 2-3); m.p. 150-152 ^{o}C; (Found: C, 75.50; H, 6.30; N, 3.10%; $C_{29}H_{29}NO_4$ requires: C, 76.46; H, 6.42; N, 3.07%); δ_H (500 MHz, $CDCl_3$) 0.72-0.84 (m, 2H, Hγ), 1.04-1.12 (m, 3H, Hδ, Hε), 1.35 (m, 1H, Hβ), 1.52 (d, 2H, *J* = 12.3 Hz, Hδ'), 1.51-1.66 (m, 3H, Hγ', Hε'), 3.46 (d, 2H, *J* = 7.0 Hz, Hα), 4.12 (t, 1H, *J* = 5.6 Hz, FHβ), 4.56 (d, 2H, *J* = 5.6 Hz, FHα), 7.15 (d, 2H, *J* = 8.1 Hz, H3), 7.24 (t, 2H, *J* = 7.4 Hz, FH4), 7.34 (d, 2H, *J* = 7.4 Hz, FH5), 7.38 (t, 2H, *J* = 7.4 Hz, FH3), 7.72 (d, 2H, *J* = 7.5 Hz, FH2), 8.03 (d, 2H, *J* = 8.1 Hz, H2); δ_C (75 MHz, $CDCl_3$) 25.67, 26.33, 30.47, 36.50, 47.24, 55.89, 67.04, 119.92, 124.79, 126.84, 126.92, 126.99, 127.65, 131.01, 141.40, 143.71, 147.08, 155.21, 171.23; ν_{max}/cm^{-1} (solid state) = 3426 (NH), ~3015 (COOH), 1737 (CO), 1365 (C-N), 1216 (C-O); ESI-HRMS found *m/z* 478.2007 $[M+Na]^+$ $C_{29}H_{29}NaNO_4$ requires 478.1989.

4-((((9*H*-fluoren-9-yl)methoxy)carbonyl)(naphthalene-2-ylmethyl)amino)-3-(prop-2-ynyloxy)benzoic acid v

4-Amino-3-(prop-2-ynyloxy)benzoic acid (1 g, 5.20 mmol), 1-naphthaldehyde (817 mg, 5.20 mmol) and picoline-borane complex (730 mg, 6.8 mmol) were stirred at room temperature in methanol (50 mL) for 14 hours. A white precipitate formed. The reaction mixture was filtered, and the filtrate acidified with 1N hydrochloric acid (~ pH 4) to induce further precipitation. The combined precipitates were dried under high vacuum to yield 4-(naphthalene-1-ylmethylamino)-3-(prop-2-ynyloxy)benzoic acid (1.16 g, 67%) as a white powder; R_F 0.60 (10% methanol in dichloromethane); (Found: C, 76.05; H, 5.15; N, 4.15%; $C_{14}H_{19}NO_2$ requires: C, 76.12; H, 5.17; N, 4.23%); δ_H (500 MHz, $CDCl_3$) 2.56 (s, 1H, $CH_2C{\equiv}C\textit{H}$), 4.60 (s, 2H, $C\textit{H}_2$naph), 4.80 (app d, 2H, *J* = 2 Hz, $C\textit{H}_2C{\equiv}CH$), 6.61 (d, 1H, *J* = 8 Hz, ArC*H*), 7.46-7.48 (m, 3H, C*H* naph), 7.60 (s, 1H, ArC*H*), 7.68 (d, 1H, *J* = 8 Hz, ArC*H*), 7.80-7.85 (m, 4H, C*H* naph); δ_C (125 MHz, $CDCl_3$) 47.51, 56.54, 76.06, 78.13, 108.95, 112.34, 116.67, 125.44, 125.92, 126.31, 126.48, 127.73, 127.79, 128.60; ν_{max}/cm^{-1} (solid state) = 3433, 3282 (NH, OH), 2130 (C≡C), 1662 (CO); ESI-MS *m/z* 333.2 $[M+H]^+$; ESI-HRMS found *m/z* 332.1273 $[M+H]^+$ $C_{21}H_{18}NO_3$ requires 332.1281.

To a refluxing solution of 4-(naphthalene-2-ylmethylamino)-3-(prop-2-ynyloxy)benzoic acid (1.16 g, 3.5 mmol) in chloroform (50 ml) was added dropwise a solution of Fmoc-chloride (1.54 g, 5.96 mmol) in chloroform (50 ml) and the reaction mixture was stirred at reflux overnight. The solvents were evaporated, then the residue was purified by column chromatography (*Stationary phase :* silica; *Mobile phase* : chloroform–methanol 95-5) to yield the pure target material (1.03 g, 53%) as an off-white solid; R_F 0.40 (chloroform–methanol 95-5); δ_H (500 MHz, $CDCl_3$) 2.34 (s, 1H, $CH_2C{\equiv}C\textit{H}$), 4.06 (s, 1H, C*H*Fmoc), 4.47-4.68 (m, 6H, $C\textit{H}_2C{\equiv}CH$, $C\textit{H}_2$naph, $C\textit{H}_2$ Fmoc), 6.91 (s, 1H, ArC*H*), 7.15 (s,

4H, ArC*H*), 7.31 (s, 2H, ArC*H*), 7.44-7.50 (m, 3H, ArC*H*), 7.61-7.62 (m, 4H, ArC*H*), 7.74-7.84 (m, 5H,ArC*H*); δ_C (75 MHz, $CDCl_3$) 46.96, 56.04, 56.32, 67.67, 76.20, 77.51, 119.75, 124.89, 125.79, 125.95, 126.81, 127.48, 127.55, 127.66, 127.79, 128.07, 130.01, 132.75, 133.12, 134.66, 141.15, 143.65, 153.02, 171.30; v_{max}/cm^{-1} (solid state) = 3290 (NH), ~3063 (COOH), 2893 (CH), 2124 (C≡C) 1693 (CO), 1338 (C-N), 1196 (C-O); ESI-HRMS found *m/z* 554.197581 $[M+H]^+$ $C_{36}H_{28}NO_5$ requires 554.196199.

4-(((9*H*-fluoren-9-yl)methoxy)carbonyl)(4-chlorobenzyl)amino)-3-(prop-2-ynyloxy)benzoic acid w

4-Amino-3-(prop-2-ynyloxy)benzoic acid (961 mg, 5.03 mmol), 4-chlorobenzaldehyde (707 mg, 5.03 mmol) and picoline-borane complex (700 mg, 6.54 mmol) were stirred at room temperature in methanol (50 mL) for 14 hours. A white precipitate formed. The reaction mixture was filtered, and the filtrate acidified with 1N hydrochloric acid (~ pH 4) to induce further precipitation. The combined precipitates were dried under high vacuum to yield 4-(4-chlorobenzylamino)-3-(prop-2-ynyloxy)benzoic acid (1.0 g, 63%) as a white powder; R_F 0.59 (10% methanol in dichloromethane); (Found: C, 64.60; H, 4.45; N, 4.30%; $C_{14}H_{19}NO_2$ requires: C, 64.67; H, 4.47; N, 4.44%); δ_H (500 MHz, DMSO-d_6) 3.63 (app. t, 1H, *J* = 2.5, $CH_2C{\equiv}CH$), 4.40 (s, 2H, C*H₂*naph), 4.88 (app d, 2H, *J* = 2.5 Hz, $CH_2C{\equiv}CH$), 6.41 (d, 1H, *J* = 8.5 Hz, C*H*CNH), 7.33-7.39 (m, 5H, C*H* naph), 7.44 (d, 1H, *J* = 1.5 Hz, C*H*COOH); δ_C (75 MHz, DMSO-d_6) 44.75, 55.92, 78.47, 79.18, 108.40, 112.17, 116.90, 125.64, 128.17, 128.63, 131.07, 138.62, 142.04, 143.28, 167.28; v_{max}/cm^{-1} (solid state) = 3429, 3290 (NH, OH), 2866 (C-H), 2284 (C≡C), 1666 (CO); ESI-MS *m/z* 316.3 $[M+H]^+$; ESI-HRMS found *m/z* 316.073756 $[M+H]^+$ $C_{17}H_{15}ClNO_3$ requires 316.073497.

^1H NMR (500 MHz, $CDCl_3$) δ: 7.73 (s, ArC*H*, 1H); 7.66 (d, ArC*H*, ^3J = 7.5, 3H); 7.37-7.34 (m, ArC*H*, 2H); 7.21-7.15 (m, ArC*H*, 8H); 6.89 (d, C*H*CNH, ^3J = 4.5, 1H); 4.68-4.31 (m, $CH_2C{\equiv}CH$, CH_2Ar, CH_2 Fmoc, 6H); 4.07 (s, C*H* Fmoc, 1H); 2.44 (s, $CH_2C{\equiv}CH$, 1H).

To a refluxing solution of 4-(4-chlorobenzylamino)-3-(prop-2-ynyloxy)benzoic acid (979 mg, 3.11 mmol) in chloroform (30 ml) was added dropwise a solution of Fmoc-chloride (1.37 g, 5.28 mmol) in chloroform (20 ml) and the reaction mixture was stirred at reflux overnight. The solvents were evaporated, then the residue taken up in chloroform and precipitated with hexane to yield the pure target material (1.22 g, 81%) as an off-white solid; R_F 0.36 (chloroform–methanol 95-5); δ_H (500 MHz, $CDCl_3$) 2.44 (s, 1H, $CH_2C{\equiv}CH$), 4.07 (s, 1H, C*H*Fmoc), 4.31-4.68 (m, 6H, $CH_2C{\equiv}CH$, CH_2naph, CH_2 Fmoc), 6.89 (d, 1H, *J* = 4.5 Hz, C*H*CNH), 7.15-7.21 (m, 8H, ArC*H*), 7.34-7.37 (m, 2H, ArC*H*), 7.66 (d, 3H, *J* = 7.5, ArC*H*), 7.73 (s, 1H, ArC*H*); δ_C (125 MHz, $CDCl_3$) 46.95, 52.44, 56.01, 67.54, 76.33, 119.80, 123.57, 124.85, 126.84, 127.33, 127.53, 128.45, 129.36, 130.03, 133.29, 135.58, 141.19, 143.61, 152.93,

170.92; ν_{max}/cm^{-1} (solid state) = 3292 (NH), ~3068 (COOH), 2893 (CH), 2124 (C≡C) 1695 (CO), 1358 (C-N), 1195 (C-O); ESI-HRMS found *m/z* 538.142162 $[M+H]^+$ $C_{32}H_{25}ClNO_5$ requires 538.141577.

Trimers

Trimers **25**, **26** and **72**[6] and **6**, **10**, **17**, **19**, **38**, **44**, **56** and **57**[1] were synthesised as described previously.

***N*-(*N*-(*N*-(Isobutyl-4-aminobenzoyl)-*N*-(4-trifluoromethyl)benzyl-4-aminobenzoyl)-*N*-isobutyl-4-aminobenzoyl)-glycine 1**

Crude: 70% pure; isolated crude yield: 44 mg then purified by Flash chromatography (6 mg), > 90% pure by NMR; δ_H (300 MHz, MeOD) 0.94 (d, 6H, *J* = 6.7 Hz, 1-Hγ), 0.98 (d, 6H, *J* = 6.6 Hz, 3-Hγ), 1.78-1.92 (m, 2H, 1-Hβ, 3-Hβ), 2.89 (d, 2H, *J* = 6.8 Hz, 1-Hα), 3.82 (d, 2H, *J* = 6.8 Hz, 3-Hα), 4.02 (s, 2H, 2-Hα), 5.13 (s, 2H, 4-Hα), 6.34 (d, 2H, *J* = 8.7 Hz, 2-H3), 6.91 (d, 2H, *J* = 8.4 Hz, 3-H2), 7.02 (d, 2H, *J* = 8.9 Hz, 2-H2), 7.17 (d, 2H, *J* = 8.7 Hz, 3-H3), 7.21 (d, 2H, *J* = 8.9 Hz, 1-H3), 7.41 (d, 2H, *J* = 8.0 Hz, 2-HAr3), 7.57 (d, 2H, *J* = 8.9 Hz, 2-HAr2), 7.79 (d, 2H, *J* = 8.7 Hz, 1-H2); ESI-MS found *m/z* 701.2 $[M-H]^-$; ESI-HRMS found *m/z* 701.2926 $[M-H]^-$ $C_{39}H_{40}F_3N_4O_5$ requires 701.2956.

***N*-(*N*-(*N*-(Benzyl-4-aminobenzoyl)-*N*-(2-methyl)naphth-1-yl-4-aminobenzoyl)-*N*-isobutyl-4-aminobenzoyl)-glycine 2**

> 95% pure by NMR, i solated crude yield: 70 mg; δ_H (300 MHz, MeOD) 0.90 (d, 6H, *J* = 6.9 Hz, 3-Hγ), 1.79 (m, 1H, 3-Hβ), 3.76 (d, 2H, *J* = 6.9 Hz, 3-Hα), 4.14 (s, 2H, 2-Hα), 4.32 (s, 2H, 1-Hα), 5.53 (s, 2H, 4-Hα), 6.40-6.45 (m, 4H, ArCH), 6.90 (d, 2H, *J* = 8.2 Hz, 3-H2), 6.99 (d, 2H, *J* = 8.2 Hz, 2-H2), 7.05 (d, 2H, *J* = 8.5 Hz, 3-H3), 7.15 (d, 1H, *J* = 8.7 Hz, 1-H3), 7.25 (m, 1H, ArCH), 7.29-7.31 (m, 4H, ArCH), 7.40-7.51 (m, 2H, ArCH), 7.69 (d, 1H, *J* = 8.9 Hz, ArCH), 7.72 (d, 2H, *J* = 8.3 Hz, ArCH), 7.82 (d, 1H, *J* = 8.0 Hz, 1-H2), 8.12 (d, 1H, *J* = 8.5 Hz, ArCH); ESI-HRMS found *m/z* 731.3222 $[M-H]^-$ $C_{46}H_{43}N_4O_5$ requires 731.3239.

***N*-(*N*-(*N*-(Phenethyl-4-aminobenzoyl)-*N*-4-chlorobenzyl-4-aminobenzoyl)-*N*-isobutyl-4-aminobenzoyl)-glycine 3**

>90% pure by NMR, isolated crude yield: 58 mg, purified by precipitation: 12 mg; δ_H (300 MHz, MeOD) 0.94 (d, 6H, *J* = 6.7 Hz, 3-Hγ), 1.84 (m, 1H, 3-Hβ), 2.91 (t, 2H, *J* = 7.6 Hz, 1-Hβ), 3.45 (t, 2H, *J* = 7.6 Hz, 1-Hα), 3.82 (d, 2H, *J* = 7.4 Hz, 3-Hα), 4.12 (s, 2H, 2-Hα), 5.04 (s, 2H, 4-Hα), 6.53 (d, 2H, *J* = 8.7 Hz, 2-H3), 6.88 (d, 2H, *J* = 8.6 Hz, 3-H2), 7.10 (d, 2H, *J* = 8.8 Hz, 2-H2), 7.15 (d, 2H, *J* = 8.6 Hz, 3-H3),

7.16-7.21 (m, 4H, ArCH), 7.22-7.31 (m, 7H, ArCH), 7.74 (d, 2H, *J* = 8.7 Hz, ArCH); ESI-MS found *m/z* 717.3 $[M+H]^+$.

N-(N-(N-(Isobutyl-4-aminobenzoyl)-N-3-fluorobenzyl-4-aminobenzoyl)-N-isobutyl-4-aminobenzoyl)-glycine 4

> 80% pure by NMR, isolated crude yield: 71 mg; δ_H (300 MHz, MeOD) 0.95 (d, 6H, *J* = 6.8 Hz, 3-Hγ), 1.02 (d, 6H, *J* = 6.4 Hz, 1-Hγ), 1.86 (m, 1H, 3-Hβ), 1.95 (m, 1H, 1-Hβ), 2.99 (t, 2H, *J* = 7.2 Hz, 3-Hα), 3.83 (t, 2H, *J* = 7.2 Hz, 1-Hα), 4.13 (s, 2H, 2-Hα), 5.08 (s, 2H, 4-Hα), 6.60 (d, 2H, *J* = 8.3 Hz, 2-H3), 6.90 (d, 2H, *J* = 8.3 Hz, 3-H2), 6.96-6.99 (m, 2H, ArCH), 7.02 (d, 1H, *J* = 8.9 Hz, ArCH), 7.13-7.16 (m, 4H, 2-H2, 3-H3), 7.18 (d, 2H, *J* = 8.7 Hz, 1-H3), 7.26 (m, 1H, ArCH), 7.75 (d, 2H, *J* = 8.3 Hz, 1-H2); ESI-HRMS found *m/z* 651.2960 $[M-H]^-$ $C_{38}H_{40}FN_4O_5$ requires 651.2988.

N-(N-(N-(Benzyl-4-aminobenzoyl)-N-4-fluorobenzyl-4-aminobenzoyl)-N-isobutyl-4-aminobenzoyl)-glycine 5

> 80% pure by NMR, isolated crude yield: 62 mg; δ_H (300 MHz, MeOD) 0.90 (d, 6H, *J* = 6.6 Hz, 3-Hγ), 1.80 (m, 1H, 3-Hβ), 3.76 (d, 2H, *J* = 7.2 Hz, 3-Hα), 4.03 (s, 2H, 2-Hα), 4.33 (s, 2H, 1-Hα), 4.98 (s, 2H, 4-Hα), 6.49 (d, 2H, *J* = 8.7 Hz, 2-H3), 6.79 (d, 2H, *J* = 8.3 Hz, 3-H2), 6.90 (d, 2H, *J* = 8.5 Hz, 2-H2), 6.92 (d, 2H, *J* = 8.7 Hz, ArCH), 7.02 (d, 2H, *J* = 8.5 Hz, ArCH), 7.07 (d, 2H, *J* = 8.3 Hz, ArCH), 7.09 (d, 2H, *J* = 8.3 Hz, ArCH), 7.13 (d, 2H, *J* = 8.2 Hz, ArCH), 7.15 (d, 2H, *J* = 8.3 Hz, ArCH), 7.22 (m, 1H, 1-HAr4), 7.26-7.30 (m, 4H, 1-HAr2,3), 7.67 (d, 2H, *J* = 8.3 Hz, 1-H2); ESI-HRMS found *m/z* 685.2804 $[M-H]^-$ $C_{41}H_{38}FN_4O_5$ requires 685.2832.

N-(N-(N-(Benzyl-4-aminobenzoyl)-N-3-fluorobenzyl-4-aminobenzoyl)-N-isobutyl-4-aminobenzoyl)-glycine 7

> 95% pure by NMR, isolated crude yield: 68 mg; δ_H (300 MHz, MeOD) 0.95 (d, 6H, *J* = 6.7 Hz, 3-Hγ), 1.84 (m, 1H, 3-Hβ), 3.82 (d, 2H, *J* = 7.4 Hz, 3-Hα), 4.07 (s, 2H, 2-Hα), 4.36 (s, 2H, 1-Hα), 5.06 (s, 2H, 4-Hα), 6.47 (d, 2H, *J* = 8.8 Hz, 2-H3), 6.88 (d, 2H, *J* = 8.5 Hz, 3-H2), 6.92-7.00 (m, 3H, 2-HAr4,5,6), 7.05 (d, 2H, *J* = 8.6 Hz, 2-H2), 7.11-7.15 (m, 4H, 3-H3, 1-H3), 7.21-7.27 (m, 2H, 1-HAr2), 7.29-7.36 (m, 3H, 1-HAr3,4), 7.34 (s, 1H, 2-HAr2), 7.75 (d, 2H, *J* = 8.5 Hz, 1-H2); ESI-HRMS found *m/z* 685.2849 $[M-H]^-$ $C_{41}H_{38}FN_4O_5$ requires 685.2832.

N-(N-(N-(Benzyl-4-aminobenzoyl)-N-naphth-1-yl-4-aminobenzoyl)-N-isobutyl-4-aminobenzoyl)-glycine 8

> 90% pure by NMR, isolated crude yield: 71 mg; Crude: 85% pure; δ_H (300 MHz, MeOD) 0.89 (d, 6H, *J* = 6.6 Hz, 3-Hγ), 1.77 (m, 1H, 3-Hβ), 3.75 (d, 2H, *J* = 7.6 Hz, 3-Hα), 4.10 (m, 2H, 1-Hα), 4.40 (s, 2H, 2-Hα), 5.55 (s, 2H, 4-Hα), 6.66 (d, 2H, *J* = 8.8 Hz, 2-H3), 6.67 (d, 2H, *J* = 8.5 Hz, 3-H2), 6.97 (d, 2H, *J* = 8.5 Hz, ArCH), 7.05 (d, 2H, *J* = 8.6 Hz, ArCH), 7.11 (d, 2H, *J* = 8.6 Hz, ArCH), 7.16 (d, 1H, *J* = 7.0 Hz, ArCH), 7.25-7.31 (m, 2H, ArCH), 7.33-7.35 (m, 4H, ArCH), 7.45-7.55 (m, 2H, ArCH), 7.67 (d, 2H, *J* = 8.5 Hz, 1-H2), 7.75 (d, 1H, *J* = 8.2 Hz, ArCH), 7.85 (dd, 1H, J_1 = 7.2 Hz and J_2 = 1.9 Hz, ArCH), 8.17 (d, 1H, *J* = 7.7 Hz, ArCH); ESI-HRMS found *m/z* 717.3080 $[M-H]^-$ $C_{45}H_{41}N_4O_5$ requires 717.3082.

N-(N-(N-(Benzyl-4-aminobenzoyl)-N-3-aminopropyl-4-aminobenzoyl)-N-isobutyl-4-aminobenzoyl)-glycine 9

Purified by column chromatography, > 85% pure by NMR, isolated yield: 6%; δ_H (300 MHz, MeOD) 0.92-0.95 (m, 6H, 3-Hγ), 1.88 (m, 1H, 3-Hβ), 3.10 (m, 2H, 2-Hβ), 3.27(m, 2H, *J* = 6.0 Hz, 2-Hα), 3.80-3.84 (m, 2H, 1-Hα), 4.09 (m, 4H, 3-Hα, 2-Hγ), 4.88 (s, 2H, 4-Hα, under solvent peak), 6.35 (d, 2H, *J* = 9.1 Hz, 2-H3), 7.04 (d, 2H, *J* = 8.7 Hz, 3-H2), 7.03 (d, 2H, *J* = 8.7 Hz, 2-H2), 7.20-7.26 (m, 4H, 3-H3, 1-H3), 7.78 (d, 2H, *J* = 8.7 Hz, 1-H2); ESI-MS *m/z* 602.4 $[M+H]^+$; ESI-HRMS found *m/z* 636.3194 $[M+H]^+$ $C_{37}H_{42}N_5O_5$ requires 636.3180.

N-(N-(N-(Benzyl-4-aminobenzoyl)-N-cyclopropylmethyl-4-aminobenzoyl)-N-isobutyl-4-aminobenzoyl)-glycine 10

> 90% pure by NMR, isolated crude yield: 60 mg; δ_H (300 MHz, MeOD) 0.01 (m, 2H, 2-Hγ/γ'), 0.35 (m, 2H, 2-Hγ'/γ), 0.92-1.00 (m, 1H, 2-Hβ), 0.96 (d, 6H, *J* = 6.7 Hz, 3-Hγ), 1.86 (m, 1H, 3-Hβ), 3.70 (d, 2H, *J* = 7.1 Hz, 3-Hα), 3.84 (d, 2H, *J* = 7.4 Hz, 2-Hα), 4.05 (s, 2H, 1-Hα), 4.37 (s, 2H, 4-Hα), 6.52 (d, 2H, *J* = 8.7 Hz, 2-H3), 6.99 (d, 2H, *J* = 8.3 Hz, 3-H2), 7.02 (d, 2H, *J* = 8.5 Hz, 2-H2), 7.17 (m, 4H, 3-H3, 1-H3), 7.27-7.34 (m, 5H, HAr2-4), 7.72 (d, 2H, *J* = 8.5 Hz, 1-H2); ESI-HRMS found *m/z* 631.2910 $[M-H]^-$ $C_{38}H_{39}N_4O_5$ requires 631.2926.

N-(N-(N-(Benzyl-4-aminobenzoyl)-N-4-fluorobenzyl-4-aminobenzoyl)-N-propyl-4-aminobenzoyl)-glycine 11

> 85% pure by NMR, isolated crude yield: 64 mg; δ_H (500 MHz, MeOD) 1.95 (t, 3H, *J* = 7.6 Hz, 3-Hγ), 1.63 (m, 2H, 3-Hβ), 3.91 (t, 2H, *J* = 7.6 Hz, 3-Hα), 4.10 (s, 2H, 1-Hα), 4.41 (s, 2H, 2-Hα), 5.05 (s, 2H, 4-Hα), 6.67 (d, 2H, *J* = 7.8 Hz, 2-H3), 6.86 (d, 2H, *J* = 8.5 Hz, 3-H2), 6.98 (t, 2H, *J* = 8.8 Hz, 1-HAr4), 7.12-

7.15 (m, 6H, ArCH), 7.21 (dd, 2H, J_1 = 8.8 Hz and J_2 = 5.4 Hz, ArCH), 7.31 (m, 1H, ArCH), 7.34-7.38 (m, 4H, ArCH), 7.73 (d, 2H, *J* = 8.5 Hz, 1-H2); ESI-HRMS found *m/z* 671.2653 $[M-H]^-$ $C_{40}H_{36}FN_4O_5$ requires 671.2675.

N-(N-(N-(3-Fluorobenzyl-4-aminobenzoyl)-N-(3-trifluoromethyl)benzyl-4-aminobenzoyl)-N-isobutyl-4-aminobenzoyl)-glycine 12

> 85% pure by NMR, isolated crude yield: 71 mg; δ_H (300 MHz, MeOD) 0.95 (d, 6H, *J* = 6.6 Hz, 3-Hγ), 1.83 (m, 1H, 3-Hβ), 3.82 (d, 2H, *J* = 7.4 Hz, 3-Hα), 4.14 (s, 2H, 1-Hα), 4.37 (s, 2H, 2-Hα), 5.17 (s, 2H, 4-Hα), 6.37 (d, 2H, *J* = 8.8 Hz, 2-H3), 6.90 (d, 2H, *J* = 8.2 Hz, 3-H2), 6.91 (d, 1H, *J* = 8.2 Hz, 1-HAr6), 7.02 (d, 2H, *J* = 8.8 Hz, 2-H2), 7.15 (d, 2H, *J* = 8.5 Hz, 3-H3), 7.16 (d, 2H, *J* = 8.4 Hz, 1-H3), 7.23 (d, 1H, *J* = 8.5 Hz, ArCH), 7.29 (m, 1H, ArCH), 7.37 (m, 1H, HAr), 7.44 (t, 1H, *J* = 8.0 Hz, 2-HAr5), 7.53 (d, 1H, *J* = 8.5 Hz, 2-HAr4), 7.60 (s, 1H, 2-HAr2), 7.72 (d, 1H, *J* = 8.5 Hz, ArCH), 7.73 (d, 2H, *J* = 8.8 Hz, 1-H2); ESI-HRMS found *m/z* 755.2832 $[M+H]^+$ $C_{42}H_{39}F_4N_4O_5$ requires 755.2851.

N-(N-(N-(4-Chlorobenzyl-4-aminobenzoyl)-N-(3-trifluoromethyl)benzyl-4-aminobenzoyl)-N-isobutyl-4-aminobenzoyl)-glycine 13

> 85% pure by NMR, isolated crude yield: 67 mg; δ_H (300 MHz, MeOD) 0.95 (d, 6H, *J* = 6.9 Hz, 3-Hγ), 1.84 (m, 1H, 3-Hβ), 3.83 (d, 2H, *J* = 7.1 Hz, 3-Hα), 4.14 (s, 2H, 1-Hα), 4.34 (s, 2H, 2-Hα), 5.18 (s, 2H, 4-Hα), 6.39 (d, 2H, *J* = 8.8 Hz, 2-H3), 6.90 (d, 2H, *J* = 8.0 Hz, 3-H2), 7.02 (d, 2H, *J* = 8.8 Hz, 2-H2), 7.13 (d, 2H, *J* = 8.5 Hz, 3-H3), 7.16 (d, 2H, *J* = 8.5 Hz, 1-H3), 7.28-7.32 (m, 4H, 1-HAr2,3,5,6), 7.37 (d, 1H, *J* = 8.2 Hz, 2-HAr6), 7.44 (t, 1H, *J* = 7.9 Hz, 2-HAr5), 7.53 (d, 1H, *J* = 8.2 Hz, 2-HAr4), 7.60 (s, 1H, 2-HAr2), 7.70 (d, 2H, *J* = 8.8 Hz, 1-H2); ESI-MS found *m/z* 772.2 $[M+H]^+$. ESI-HRMS found *m/z* 771.2567 $[M+H]^+$ $C_{42}H_{39}ClF_3N_4O_5$ requires 771.2555.

N-(N-(N-(4-Fluorobenzyl-4-aminobenzoyl)-N-(3-trifluoromethyl)benzyl-4-aminobenzoyl)-N-isobutyl-4-aminobenzoyl)-glycine 14

> 85% pure by NMR, isolated crude yield: 74 mg; δ_H (300 MHz, MeOD) 0.94 (d, 6H, *J* = 6.6 Hz, 3-Hγ), 1.84 (m, 1H, 3-Hβ), 3.82 (d, 2H, *J* = 7.3 Hz, 3-Hα), 4.14 (s, 2H, 1-Hα), 4.33 (s, 2H, 2-Hα), 5.17 (s, 2H, 4-Hα), 6.41(d, 2H, *J* = 8.8 Hz, 2-H3), 6.87-6.92 (m, 2H, ArCH), 6.98-7.07 (m, 2H, ArCH), 7.13-7.17 (m, 4H, ArCH), 7.28 (d, 2H, *J* = 8.7 Hz, ArCH), 7.36 (dd, 1H, J_1 = 8.8 Hz and J_2 = 5.8 Hz, ArCH), 7.45 (d, 2H, *J* = 8.2 Hz, ArCH), 7.52-7.57 (m, 2H, ArCH), 7.60 (s, 1H, 2-HAr2), 7.70-7.74 (m, 2H, ArCH); ESI-HRMS found *m/z* 753.2734 $[M-H]^-$ $C_{42}H_{37}F_4N_4O_5$ requires 753.2706.

N-(N-(N-(Benzyl-4-aminobenzoyl)-N-5-bromoindol-3-yl-4-aminobenzoyl)-N-isobutyl-4-aminobenzoyl)-glycine 15

> 85% pure by NMR, isolated crude yield: 73 mg; δ_H (300 MHz, MeOD) 0.93 (d, 6H, *J* = 6.7 Hz, 3-Hγ), 1.86 (m, 1H, 3-Hβ), 3.81 (d, 2H, *J* = 7.4 Hz, 3-Hα), 4.07 (s, 2H, 1-Hα), 4.33 (s, 2H, 2-Hα), 5.19 (s, 2H, 4-Hα), 6.48 (d, 2H, *J* = 8.6 Hz, 2-H3), 6.72 (d, 2H, *J* = 8.4 Hz, 3-H2), 7.00-7.07 (m, 3H, ArCH), 7.10-7.18 (m, 4H, ArCH), 7.23 (d, 2H, *J* = 8.9 Hz, ArCH), 7.28-7.34 (m, 4H, ArCH), 7.71 (d, 2H, *J* = 8.5 Hz, 1-H2), 7.78 (d, 2H, *J* = 8.8 Hz, ArCH); ESI-HRMS found *m/z* 784.2137 $[M-H]^-$ $C_{43}H_{39}BrN_5O_5$ requires 784.2140.

N-(N-(N-(Benzyl-4-aminobenzoyl)-N-cyclohexylmethyl-4-aminobenzoyl)-N-isobutyl-4-aminobenzoyl)-glycine 16

> 80% pure by NMR, isolated crude yield: 69 mg; δ_H (300 MHz, MeOD) 0.91 (d, 6H, *J* = 6.8 Hz, 3-Hγ), 0.91-0.95 (m, 2H, 2-Hε), 1.06-1.11 (m, 4H, 2-Hδ,δ'), 1.56-1.66 (m, 5H, 2-Hγ,γ',β), 1.82 (m, 1H, 3-Hβ), 3.68 (d, 2H, *J* = 7.2 Hz, 3-Hα), 3.79 (d, 2H, *J* = 7.6 Hz, 2-Hα), 4.01 (s, 2H, 1-Hα), 4.34 (s, 2H, 4-Hα), 6.50 (d, 2H, *J* = 8.5 Hz, 2-H3), 6.91 (d, 2H, *J* = 8.3 Hz, 3-H2), 6.85 (d, 2H, *J* = 8.5 Hz, 2-H2), 7.13 (d, 2H, *J* = 8.3 Hz, 3-H3), 7.14 (d, 2H, *J* = 8.3 Hz, 1-H3), 7.22 (m, 1H, 1-HAr4), 7.26-7.31 (m, 4H, 1-HAr2,3), 7.68 (d, 2H, *J* = 8.5 Hz, 1-H2); ESI-HRMS found *m/z* 673.3419 $[M-H]^-$ $C_{41}H_{45}N_4O_5$ requires 673.3395.

N-(N-(N-(3-Fluorobenzyl-4-aminobenzoyl)-N-phenethyl-4-aminobenzoyl)-N-propyl-4-aminobenzoyl)-glycine 18

> 75% pure by NMR, isolated crude yield: 57 mg; δ_H (300 MHz, MeOD) 0.94 (t, 3H, *J* = 7.4 Hz, 3-Hγ), 1.62 (m, 2H, 3-Hβ), 2.86 (t, 2H, *J* = 7.4 Hz, 3-Hα), 3.90 (t, 2H, *J* = 7.5 Hz, 2-Hβ), 3.99 (t, 2H, *J* = 7.5 Hz, 2-Hα), 4.04 (s, 2H, 1-Hα), 4.34 (s, 2H, 4-Hα), 6.43 (d, 2H, *J* = 8.7 Hz, 2-H3), 6.75 (d, 2H, *J* = 8.5 Hz, 3-H2), 6.93 (d, 2H, *J* = 8.7 Hz, 2-H2), 7.12-7.16 (m, 6H, ArCH), 7.22-7.26 (m, 4H, ArCH), 7.29-7.33 (m, 4H, ArCH), 7.74 (d, 2H, *J* = 8.7 Hz, 1-H2); ESI-HRMS found *m/z* 667.2893 $[M-H]^-$ $C_{41}H_{39}N_4O_5$ requires 667.2926.

N-(N-(N-(Benzyl-4-aminobenzoyl)-N-(3-trifluoromethyl)benzyl-4-aminobenzoyl)-N-cyclohexylmethyl-4-aminobenzoyl)-glycine 20

> 90% pure by NMR, isolated crude yield: 72 mg; δ_H (300 MHz, MeOD) 1.04 (m, 2H, 3-Hε), 1.13-1.23 (m, 4H, 3-Hδ,δ'), 1.55 (m, 1H, 3-Hβ), 1.65-1.74 (m, 4H, 3-Hγ,γ'), 3.83 (d, 2H, *J* = 7.0 Hz, 3-Hα), 4.07 (s, 2H, 1-Hα), 4.37 (s, 2H, 2-Hα), 5.14 (s, 2H, 4-Hα), 6.51 (d, 2H, *J* = 8.8 Hz, 2-H3), 6.88 (d, 2H, *J* = 8.4 Hz, 3-H2), 7.07 (d, 2H, *J* = 8.8 Hz, 2-H2), 7.12-7.16 (m, 4H, ArCH), 7.30-7.36 (m, 4H, ArCH), 7.41-7.46 (m, 2H, ArCH), 7.53 (d, 1H, *J* = 8.6 Hz, 2-HAr4), 7.59 (s, 1H, 2-HAr2), 7.72 (d, 2H, *J* = 8.5 Hz, 1-H2), 7.99 (s, 1H, NH); ESI-HRMS found *m/z* 775.3127 $[M-H]^-$ $C_{45}H_{42}F_3N_4O_5$ requires 775.3113.

***N*-(*N*-(*N*-(Cyclopropylmethyl-4-aminobenzoyl)-*N*-phenethyl-4-aminobenzoyl)-*N*-3-fluorobenzyl-4-aminobenzoyl)-glycine 21**

> 95% pure by NMR, isolated crude yield: 68 mg; δ_H (300 MHz, $CDCl_3$) 0.39 (m, 2H, 1-Hγ), 0.71 (m, 2H, 1-Hγ'), 1.12 (m, 1H, 1-Hβ), 2.94 (t, 2H, *J* = 7.4 Hz, 2-Hβ), 3.20 (d, 2H, *J* = 7.4 Hz, 1-Hα), 4.11-4.16 (m, 4H, 2-Hα and 3-Hα), 5.13 (s, 2H, 4-Hα), 6.66 (d, 2H, *J* = 8.4 Hz, 2-H3), 6.92 (d, 2H, *J* = 8.5 Hz, 3-H2), 6.96-7.02 (m, 4H, ArCH), 7.15-7.18 (m, 4H, ArCH), 7.22-7.25 (m, 4H, ArCH), 7.27-7.29 (m, 2H, ArCH), 7.32 (d, 2H, *J* = 8.5 Hz, 1-H3), 7.56 (d, 2H, *J* = 8.5 Hz, 1-H2), 8.82 (t, 1H, *J* = 5.8 Hz, Ar-NH); ESI-HRMS found *m/z* 697.2855 $[M-H]^-$ $C_{42}H_{38}FN_4O_5$ requires 697.2832.

***N*-(*N*-(*N*-(3-Fluorobenzyl-4-aminobenzoyl)-*N*-phenethyl-4-aminobenzoyl)-*N*-cyclopropylmethyl-4-aminobenzoyl)-glycine 22**

> 90% pure by NMR, isolated crude yield: 69 mg; δ_H (300 MHz, $CDCl_3$) 0.19 (m, 2H, 3-Hγ), 0.48 (m, 2H, 3-Hγ'), 1.06 (m, 1H, 3-Hβ), 2.92 (t, 2H, *J* = 7.5 Hz, 2-Hβ), 3.84 (d, 2H, *J* = 7.0 Hz, 3-Hα), 4.00 (m, 2H, 1-Hα), 4.11 (t, 2H, *J* = 7.5 Hz, 2-Hα), 4.42 (s, 2H, 4-Hα), 6.64 (d, 2H, *J* = 8.5 Hz, 2-H3), 7.06 (d, 2H, *J* = 8.4 Hz, 3-H2), 7.11-7.19 (m, 10H, ArCH), 7.24-7.28 (m, 4H, ArCH), 7.31-7.35 (m, 1H, ArCH), 7.62 (d, 2H, *J* = 8.4 Hz, 1-H2), 8.78 (t, 1H, *J* = 4.8 Hz, Ar-NH); ESI-HRMS found *m/z* 697.2859 $[M-H]^-$ $C_{42}H_{38}FN_4O_5$ requires 697.2832.

***N*-(*N*-(*N*-(Phenethyl-4-aminobenzoyl)-*N*-(4-trifluoromethyl)benzyl-4-aminobenzoyl)-*N*-isobutyl-4-aminobenzoyl)-glycine 23**

> 85% pure by NMR, isolated crude yield: 67 mg; δ_H (300 MHz, MeOD) 0.94 (d, 6H, *J* = 6.7 Hz, 3-Hγ), 1.84 (m, 1H, 3-Hβ), 2.95 (t, 2H, *J* = 7.5 Hz, 1-Hβ), 3.45 (t, 2H, *J* = 7.5 Hz, 1-Hα), 3.82 (d, 2H, *J* = 7.5 Hz, 3-Hα), 4.12 (s, 2H, 2-Hα), 5.15 (s, 2H, 4-Hα), 6.69 (d, 2H, *J* = 8.6 Hz, 2-H3), 6.92 (d, 2H, *J* = 8.5 Hz, 3-H2), 7.14-7.19 (m, 6H, ArCH), 7.23-7.32 (m, 5H, ArCH), 7.42 (d, 2H, *J* = 8.0 Hz, ArCH), 7.58 (d, 2H, *J* = 8.6 Hz, ArCH), 7.73 (d, 2H, *J* = 8.6 Hz, 1-H2); ESI-HRMS found *m/z* 749.2967 $[M-H]^-$ $C_{43}H_{40}F_3N_4O_5$ requires 749.2956.

***N*-(*N*-(*N*-(4-Fluorobenzyl-4-aminobenzoyl)-*N*-cyclohexylmethyl-4-aminobenzoyl)-*N*-propyl-4-aminobenzoyl)-glycine 24**

> 85% pure by NMR, isolated crude yield: 60 mg; δ_H (300 MHz, MeOD) 0.96 (t, 3H, *J* = 7.2 Hz, 3-Hγ), 0.96-1.04 (m, 2H, 2-Hε), 1.10-1.19 (m, 2H, 2-Hδ), 1.47 (m, 2H, 2-Hδ'), 1.60-1.73 (m, 7H, 3Hβ, 2-Hγ,γ',β), 3.73 (d, 2H, *J* = 7.1 Hz, 2-Hα), 3.93 (t, 2H, *J* = 7.6 Hz, 3-Hα), 4.06 (s, 2H, 1-Hα), 4.33 (s, 2H, 4-Hα), 6.40 (d, 2H, *J* = 8.8 Hz, 2-H3), 6.93 (d, 2H, *J* = 8.5 Hz, 3-H2), 6.96 (d, 2H, *J* = 8.3 Hz, 1-HAr2), 7.02 (d, 2H, *J* = 8.8 Hz, 2-H2), 7.18 (d, 2H, *J* = 8.2 Hz, 1-HAr3), 7.21 (d, 2H, *J* = 8.5 Hz, 3-H3), 7.35 (d, 2H, *J* =

8.5 Hz, 1-H3), 7.76 (d, 2H, *J* = 8.5 Hz, 1-H2); ESI-HRMS found *m/z* 677.3129 $[M-H]^-$ $C_{40}H_{42}FN_4O_5$ requires 677.3145.

N-(N-(N-(Benzyl-4-aminobenzoyl)-N-naphth-2-yl-4-aminobenzoyl)-N-cyclopropylmethyl-4-aminobenzoyl)-glycine 27

> 80% pure by NMR, isolated crude yield: 66 mg; δ_H (300 MHz, $CDCl_3$) 0.15 (m, 2H, 3-Hγ), 0.43 (m, 2H, 3-Hγ'), 0.86 (m, 1H, 3-Hβ), 3.78 (d, 2H, *J* = 7.0 Hz, 3-Hα), 3.96 (d, 2H, *J* = 4.9 Hz, 1-Hα), 4.40 (s, 2H, 2-Hα), 5.22 (s, 2H, 4-Hα), 6.75 (d, 2H, *J* = 8.4 Hz, 2-H3), 6.99 (d, 2H, *J* = 8.2 Hz, 3-H2), 7.06-7.11 (m, 4H, ArCH), 7.24 (s, 1H, 2-HAr2), 7.34-7.37 (m, 5H, ArCH), 7.44-7.47 (m, 3H, ArCH), 7.59-7.65 (m, 3H, ArCH), 7.77 (d, 2H, *J* = 8.8 Hz, 1-H2), 7.74-7.81 (m, 2H, ArCH); ESI-HRMS found *m/z* 715.2929 $[M-H]^-$ $C_{45}H_{39}N_4O_5$ requires 715.2926.

N-(N-(N-(4-Chlorobenzyl-4-aminobenzoyl)-N-naphth-2-yl-4-aminobenzoyl)-N-cyclopropylmethyl-4-aminobenzoyl)-glycine 28

> 90% pure by NMR, isolated crude yield: 71 mg; δ_H (500 MHz, $CDCl_3$) 0.42 (m, 2H, 3-Hγ), 0.72 (m, 2H, 3-Hγ'), 0.86 (m, 1H, 3-Hβ), 3.19 (d, 2H, *J* = 7.4 Hz, 3-Hα), 4.09 (d, 2H, *J* = 5.2 Hz, 1-Hα), 5.07 (s, 2H, 2-Hα), 5.27 (s, 2H, 4-Hα), 6.81 (d, 2H, *J* = 8.2 Hz, 2-H3), 6.88 (d, 2H, *J* = 8.2 Hz, 3-H2), 7.13-7.19 (m, 4H, ArCH), 7.25 (d, 2H, *J* = 8.4 Hz, ArCH), 7.33-7.39 (m, 5H, ArCH), 7.45-7.48 (m, 2H, ArCH), 7.59 (d, 2H, *J* = 8.2 Hz, ArCH), 7.66 (s, 1H, 2-HAr2), 7.78 (d, 2H, *J* = 8.4 Hz, 1-H2), 7.78-7.82 (m, 1H, ArCH), 7.95 (t, 1H, *J* = 5.2 Hz, Ar-NH); ESI-HRMS found *m/z* 749.2560 $[M-H]^-$ $C_{45}H_{38}ClN_4O_5$ requires 749.2536.

N-(N-(N-((3-Trifluoromethyl)benzyl-4-aminobenzoyl)-N-(3-trifluoromethyl)benzyl-4-aminobenzoyl)-N-benzyl-4-aminobenzoyl)-glycine 29

> 90% pure by NMR, isolated crude yield: 83 mg; δ_H (300 MHz, MeOD) 4.05 (s, 2H, 3-Hα), 4.43 (s, 2H, 1-Hα), 5.14 (s, 2H, 2-Hα), 5.16 (s, 2H, 4-Hα), 6.36 (d, 2H, *J* = 8.8 Hz, 2-H3), 6.89 (d, 2H, *J* = 8.5 Hz, 3-H2), 7.00 (d, 2H, *J* = 8.5 Hz, 3-H3), 7.01 (d, 2H, *J* = 8.6 Hz, 2-H2), 7.21 (d, 2H, *J* = 8.5 Hz, 1-H3), 7.24-7.28 (m, 6H, ArCH), 7.37 (d, 1H, *J* = 7.8 Hz, ArCH), 7.42 (d, 1H, *J* = 7.7 Hz, ArCH), 7.46-7.51 (m, 2H, ArCH), 7.57-7.59 (m, 3H, ArCH), 7.63 (d, 2H, *J* = 8.6 Hz, 1-H2); ESI-HRMS found *m/z* 837.2558 $[M-H]^-$ $C_{46}H_{35}F_6N_4O_5$ requires 837.2517.

N-(N-(N-(4-Chlorobenzyl-4-aminobenzoyl)-N-naphth-2-yl-4-aminobenzoyl)-N-cyclopropylmethyl-4-aminobenzoyl)-glycine 30

> 95% pure by NMR, isolated crude yield: 73 mg; δ_H (300 MHz, MeOD) 0.14 (m, 2H, 3-Hγ), 0.45 (m, 2H, 3-Hγ'), 1.06 (m, 1H, 3-Hβ), 3.81 (d, 2H, *J* = 7.1 Hz, 3-Hα), 4.09 (s, 2H, 1-Hα), 4.36 (s, 2H, 2-Hα),

5.14 (s, 2H, 4-Hα), 6.45 (d, 2H, *J* = 8.8 Hz, 2-H3), 6.88 (d, 2H, *J* = 8.4 Hz, 3-H2), 7.05 (d, 2H, *J* = 8.8 Hz, 2-H2), 7.17 (d, 2H, *J* = 8.4 Hz, 3-H3), 7.19 (d, 2H, *J* = 8.5 Hz, 1-H3), 7.30-7.33 (m, 4H, 1-HAr2,3), 7.37 (d, 1H, *J* = 7.6 Hz, 2-HAr6), 7.43 (t, 1H, *J* = 7.6 Hz, 2-HAr5), 7.53 (d, 1H, *J* = 7.5 Hz, 2-HAr4), 7.59 (s, 1H, 2-HAr2), 7.73 (d, 2H, *J* = 8.5 Hz, 1-H2); ESI-HRMS found *m/z* 767.2263 $[M-H]^-$ $C_{42}H_{35}ClF_3N_4O_5$ requires 767.2254.

***N*-(*N*-(*N*-(4-Fluorobenzyl-4-aminobenzoyl)-*N*-naphth-2-yl-4-aminobenzoyl)-*N*-propyl-4-aminobenzoyl)-glycine 31**

> 90% pure by NMR, isolated crude yield: 69 mg; δ_H (300 MHz, MeOD) 0.91 (t, 3H, *J* = 7.4 Hz, 3-Hγ), 1.59 (m, 2H, 3-Hβ), 3.87 (t, 2H, *J* = 7.5 Hz, 3-Hα), 4.09 (s, 2H, 1-Hα), 4.33 (s, 2H, 2-Hα), 5.23 (s, 2H, 4-Hα), 6.41 (d, 2H, *J* = 8.8 Hz, 2-H3), 6.88 (d, 2H, *J* = 8.5 Hz, 3-H2), 6.89 (d, 2H, *J* = 8.5 Hz, ArCH), 7.11 (d, 2H, *J* = 8.5 Hz, ArCH), 7.12 (d, 2H, *J* = 8.8 Hz, ArCH), 7.28 (d, 1H, *J* = 8.8 Hz, ArCH), 7.33-7.38 (m, 2H, ArCH), 7.42-7.47 (m, 4H, ArCH), 7.63 (d, 2H, *J* = 8.2 Hz, 1-H3), 7.70 (d, 1H, *J* = 8.5 Hz, ArCH), 7.72 (d, 1H, *J* = 8.5 Hz, ArCH), 7.79 (d, 2H, *J* = 8.2 Hz, 1-H2); ESI-HRMS found *m/z* 721.2843 $[M-H]^-$ $C_{44}H_{38}FN_4O_5$ requires 721.2832.

***N*-(*N*-(*N*-(Naphth-2-yl-4-aminobenzoyl)-*N*-isobutyl-4-aminobenzoyl)-*N*-benzyl-4-aminobenzoyl)-glycine 32**

~ 70% pure by NMR, isolated crude yield: 61 mg; δ_H (500 MHz, DMSO-d_6) 0.77 (d, 6H, *J* = 5 Hz, 2-Hγ), 1.66 (m, 1H, 2-Hβ), 3.60 (d, 2H, *J* = 5 Hz, 2-Hα), 3.88 (s, 2H, 1-Hα), 4.41 (s, 2H, 3-Hα), 5.12 (s, 2H, 4-Hα), 6.38 (d, 2H, *J* = 8.8 Hz, 2-H3), 6.86-6.96 (m, 4H, ArCH), 7.07 (d, 2H, *J* = 5 Hz, ArCH), 7.22 (d, 2H, *J* = 5 Hz, ArCH), 7.27-7.32 (m, 4H, ArCH), 7.45-7.49 (m, 2H, ArCH), 7.66 (d, 2H, *J* = 10 Hz, ArCH), 7.80-7.87 (m, 3H, ArCH), 8.87 (m, 1H, NH); ESI-MS found *m/z* 717.2 $[M-H]^-$.

***N*-(*N*-(*N*-(4-Fluorobenzyl-4-aminobenzoyl)-*N*-(4-fluorobenzyl)-4-aminobenzoyl)-*N*-isobutyl-4-aminobenzoyl)-glycine 33**

~70% pure by NMR, no identifiable impurity on LC-MS; isolated crude yield: 62 mg; δ_H (300 MHz, MeOD) 0.95 (d, 6H, *J* = 6.6 Hz, 3-Hγ), 1.85 (m, 1H, 3-Hβ), 3.82 (d, 2H, *J* = 7.4 Hz, 3-Hα), 4.09 (s, 2H, 2-Hα), 4.36 (s, 2H, 1-Hα), 5.03 (s, 2H, 4-Hα), 6.37 (d, 2H, *J* = 9.0 Hz, 2-H3), 6.84 (d, 2H, *J* = 8.5 Hz, 3-H2), 6.95 (d, 2H, *J* = 8.8 Hz, ArCH), 7.01 (d, 2H, *J* = 8.8 Hz, ArCH), 7.01-7.06 (m, 2H, ArCH), 7.13 (d, 2H, *J* = 8.5 Hz, ArCH), 7.14-7.20 (m, 6H, ArCH), 7.75 (d, 2H, *J* = 8.5 Hz, 1-H2); ESI-MS found *m/z* 703.1 $[M-H]^-$; ESI-HRMS found *m/z* 705.2893 $[M+H]^+$ $C_{41}H_{39}F_2N_4O_5$ requires 705.2883.

N-(N-(N-(4-Fluorobenzyl-4-aminobenzoyl)-N-(3-fluorobenzyl)-4-aminobenzoyl)-N-isobutyl-4-aminobenzoyl)-glycine 34

> 85% pure by NMR, no identifiable impurity on LC-MS; isolated crude yield: 63 mg; δ_H (300 MHz, MeOD) 0.95 (d, 6H, *J* = 6.6 Hz, 3-Hγ), 1.84 (m, 1H, 3-Hβ), 3.83 (d, 2H, *J* = 7.4 Hz, 3-Hα), 4.08 (s, 2H, 2-Hα), 4.32 (s, 2H, 1-Hα), 5.06 (s, 2H, 4-Hα), 6.38 (d, 2H, *J* = 8.8 Hz, 2-H3), 6.87 (d, 2H, *J* = 8.5 Hz, 3-H2), 6.92-6.98 (m, 3H, ArCH), 7.01-7.03 (m, 4H, ArCH), 7.14 (d, 2H, *J* = 8.2 Hz, ArCH), 7.16 (d, 2H, *J* = 8.2 Hz, ArCH), 7.23 (m, 1H, ArCH), 7.33-7.37 (m, 2H, ArCH), 7.73 (d, 2H, *J* = 8.8 Hz, 1-H2); ESI-MS found *m/z* 703.1 $[M-H]^-$; ESI-HRMS found *m/z* 727.2716 $[M+Na]^+$ $C_{41}H_{38}F_2N_4NaO_5$ requires 727.2702.

N-(N-(N-(Benzyl-4-aminobenzoyl)-N-(3-trifluoromethyl)benzyl-4-aminobenzoyl)-N-2-methylbutyl-4-aminobenzoyl)-glycine 35

> 85% pure by NMR, isolated crude yield: 71 mg; δ_H (300 MHz, MeOD) 0.88 (t, 3H, *J* = 7.4 Hz, 3-Hδ), 0.93 (d, 3H, *J* = 6.6 Hz, 3-Hε), 1.18 (m, 1H, 3-Hβ), 1.46 (m, 1H, 3-Hγ), 1.60 (m, 1H, 3-Hγ'), 3.87 (d, 2H, *J* = 7.4 Hz, 3-Hα), 4.07 (s, 2H, 1-Hα), 4.36 (s, 2H, 2-Hα), 5.14 (s, 2H, 4-Hα), 6.47 (d, 2H, *J* = 8.6 Hz, 2-H3), 6.89 (d, 2H, *J* = 8.5 Hz, 3-H2), 7.06 (d, 2H, *J* = 8.6 Hz, 2-H2), 7.14 (d, 2H, *J* = 8.5 Hz, 3-H3), 7.15 (d, 2H, *J* = 8.5 Hz, 1-H3), 7.25 (m, 1H, 1-HAr4), 7.29-7.35 (m, 4H, 1-HAr2,3), 7.37 (d, 1H, *J* = 8.2 Hz, 2-HAr6), 7.44 (t, 1H, *J* = 7.5 Hz, 2-HAr5), 7.53 (d, 1H, *J* = 7.4 Hz, 2-HAr4), 7.60 (s, 1H, 2-HAr2), 7.72 (d, 2H, *J* = 8.5 Hz, 1-H2); ESI-MS found *m/z* 749.2 $[M-H]^-$; ESI-HRMS found *m/z* 773.2936 $[M+Na]^+$ $C_{43}H_{41}F_3NaN_4O_5$ requires 773.2921.

N-(N-(N-(Benzyl-4-aminobenzoyl)-N-(4-trifluoromethyl)benzyl-4-aminobenzoyl)-N-2-methylbutyl-4-aminobenzoyl)-glycine 36

> 95% pure by NMR, isolated crude yield: 68 mg; δ_H (300 MHz, MeOD) 0.88 (t, 3H, *J* = 7.3 Hz, 3-Hδ), 0.93 (d, 3H, *J* = 6.9 Hz, 3-Hε), 1.19 (m, 1H, 3-Hβ), 1.45 (m, 1H, 3-Hγ), 1.61 (m, 1H, 3-Hγ'), 3.87 (d, 2H, *J* = 7.4 Hz, 3-Hα), 4.07 (s, 2H, 1-Hα), 4.37 (s, 2H, 2-Hα), 5.13 (s, 2H, 4-Hα), 6.47 (d, 2H, *J* = 8.8 Hz, 2-H3), 6.91 (d, 2H, *J* = 8.4 Hz, 3-H2), 7.05 (d, 2H, *J* = 8.8 Hz, 2-H2), 7.14 (d, 2H, *J* = 8.4 Hz, 3-H3), 7.16 (d, 2H, *J* = 8.5 Hz, 1-H3), 7.25 (m, 1H, 1-HAr4), 7.29-7.36 (m, 4H, 1-HAr2,3), 7.41 (d, 2H, *J* = 8.2 Hz, 2-HAr2), 7.57 (d, 2H, *J* = 8.2 Hz, 2-HAr4), 7.73 (d, 2H, *J* = 8.5 Hz, 1-H2); ESI-HRMS found *m/z* 751.3081 $[M+H]^+$ $C_{43}H_{42}F_3N_4O_5$ requires 751.3102.

N-(N-(N-(4-Fluorobenzyl-4-aminobenzoyl)-N-(3-trifluoromethyl)benzyl-4-aminobenzoyl)-N-2-methylbutyl-4-aminobenzoyl)-glycine 37

> 95% pure by NMR, isolated crude yield: 73 mg; δ_H (300 MHz, MeOD) 0.88 (t, 3H, *J* = 7.4 Hz, 3-Hδ), 0.93 (d, 3H, *J* = 6.9 Hz, 3-Hε), 1.18 (m, 1H, 3-Hβ), 1.46 (m, 1H, 3-Hγ), 1.59 (m, 1H, 3-Hγ'), 3.87 (d, 2H, *J*

= 7.4 Hz, 3-Hα), 4.08 (s, 2H, 1-Hα), 4.35 (s, 2H, 2-Hα), 5.14 (s, 2H, 4-Hα), 6.45 (d, 2H, *J* = 8.6 Hz, 2-H3), 6.88 (d, 2H, *J* = 8.2 Hz, 3-H2), 7.03 (d, 2H, *J* = 8.6 Hz, 2-H2), 7.06 (d, 2H, *J* = 8.2 Hz, 3-H3), 7.15 (d, 2H, *J* = 8.5 Hz, ArCH), 7.16 (d, 2H, *J* = 8.5 Hz, ArCH), 7.36 (d, 1H, *J* = 8.2 Hz, 2-HAr6), 7.37 (d, 2H, *J* = 8.2 Hz, 1-HAr3), 7.44 (t, 1H, *J* = 7.7 Hz, 2-HAr5), 7.53 (d, 1H, *J* = 7.7 Hz, 2-HAr4), 7.60 (s, 1H, 2-HAr2), 7.72 (d, 2H, *J* = 8.5 Hz, 1-H2); ESI-MS found *m/z* 767.3 $[M-H]^-$; ESI-HRMS found *m/z* 767.2872 $[M-H]^-$ $C_{43}H_{39}F_4N_4O_5$ requires 767.2862.

***N*-(*N*-(*N*-(Phenethyl-4-aminobenzoyl)-*N*-(3-trifluoromethyl)benzyl-4-aminobenzoyl)-*N*-2-methylbutyl-4-aminobenzoyl)-glycine 39**

> 95% pure by NMR, isolated crude yield: 67 mg; δ_H (300 MHz, MeOD) 0.87 (t, 3H, *J* = 7.4 Hz, 3-Hδ), 0.92 (d, 3H, *J* = 6.6 Hz, 3-Hε), 1.18 (m, 1H, 3-Hβ), 1.45 (m, 1H, 3-Hγ), 1.60 (m, 1H, 3-Hγ'), 2.92 (t, 2H, *J* = 7.45 Hz, 1-Hβ), 3.41 (t, 2H, *J* = 7.45 Hz, 1-Hα), 3.86 (d, 2H, *J* = 7.2 Hz, 3-Hα), 4.11 (s, 2H, 2-Hα), 5.15 (s, 2H, 4-Hα), 6.55 (d, 2H, *J* = 8.5 Hz, 2-H3), 6.89 (d, 2H, *J* = 8.2 Hz, 3-H2), 7.12 (d, 2H, *J* = 8.5 Hz, 2-H2), 7.15 (d, 2H, *J* = 8.2 Hz, 3-H3), 7.17 (d, 2H, *J* = 8.5 Hz, 1-H3), 7.23 (m, 1H, 1-HAr4), 7.27-7.30 (m, 4H, 1-HAr2-3), 7.38 (d, 1H, *J* = 8.0 Hz, 2-HAr6), 7.45 (t, 1H, *J* = 7.7 Hz, 2-HAr5), 7.55 (d, 1H, *J* = 7.7 Hz, 2-HAr4), 7.61 (s, 1H, 2-HAr2), 7.73 (d, 2H, *J* = 8.5 Hz, 1-H2); ESI-MS found *m/z* 766.0 $[M+H]^+$; ESI-HRMS found *m/z* 763.3126 $[M-H]^-$ $C_{44}H_{42}F_3N_4O_5$ requires 763.3113.

***N*-(*N*-(*N*-(4-Chlorobenzyl-4-aminobenzoyl)-*N*-(4-trifluoromethyl)benzyl-4-aminobenzoyl)-*N*-(2-methyl)butyl-4-aminobenzoyl)-glycine 40**

> 95% pure by NMR, isolated crude yield: 76 mg; δ_H (300 MHz, MeOD) 0.88 (t, 3H, *J* = 7.4 Hz, 3-Hδ), 0.93 (d, 3H, *J* = 6.7 Hz, 3-Hε), 1.19 (m, 1H, 3-Hβ), 1.46 (m, 1H, 3-Hγ), 1.60 (m, 1H, 3-Hγ'), 3.87 (d, 2H, *J* = 7.4 Hz, 3-Hα), 4.08 (s, 2H, 1-Hα), 4.35 (s, 2H, 2-Hα), 5.13 (s, 2H, 4-Hα), 6.41 (d, 2H, *J* = 8.7 Hz, 2-H3), 6.90 (d, 2H, *J* = 8.2 Hz, 3-H2), 7.03 (d, 2H, *J* = 8.7 Hz, 2-H2), 7.15 (d, 2H, *J* = 8.2 Hz, 3-H3), 7.17 (d, 2H, *J* = 8.5 Hz, 1-H3), 7.29-7.33 (m, 4H, 1-HAr2,3), 7.40 (d, 2H, *J* = 8.0 Hz, 2-HAr2), 7.57 (d, 2H, *J* = 8.2 Hz, 2-HAr3), 7.70 (d, 2H, *J* = 8.5 Hz, 1-H2); ESI-HRMS found *m/z* 783.2554 $[M-H]^-$ $C_{43}H_{39}ClF_3N_4O_5$ requires 783.2567.

***N*-(*N*-(*N*-(Phenethyl-4-aminobenzoyl)-*N*-(4-trifluoromethyl)benzyl-4-aminobenzoyl)-*N*-(2-methyl)butyl-4-aminobenzoyl)-glycine 41**

> 95% pure by NMR, isolated crude yield: 70 mg; δ_H (300 MHz, MeOD) 0.87 (t, 3H, *J* = 7.4 Hz, 3-Hδ), 0.92 (d, 3H, *J* = 6.6 Hz, 3-Hε), 1.18 (m, 1H, 3-Hβ), 1.45 (m, 1H, 3-Hγ), 1.59 (m, 1H, 3-Hγ'), 2.92 (t, 2H, *J* = 7.5 Hz, 1-Hβ), 3.39 (t, 2H, *J* = 7.5 Hz, 1-Hα), 3.86 (d, 2H, *J* = 7.4 Hz, 3-Hα), 4.11 (s, 2H, 2-Hα), 5.14 (s,

2H, 4-Hα), 6.52 (d, 2H, *J* = 8.8 Hz, 2-H3), 6.92 (d, 2H, *J* = 8.5 Hz, 3-H2), 7.11 (d, 2H, *J* = 8.5 Hz, 2-H2), 7.16 (d, 2H, *J* = 8.5 Hz, 3-H3), 7.18 (d, 2H, *J* = 8.5 Hz, 1-H3), 7.22 (m, 1H, 1-HAr4), 7.25-7.30 (m, 4H, 1-HAr2,3), 7.42 (d, 2H, *J* = 8.1 Hz, 2-HAr2), 7.58 (d, 2H, *J* = 8.1 Hz, 2-HAr3), 7.75 (d, 2H, *J* = 8.2 Hz, 1-H2); δ_C (75 MHz, MeOD) 11.52, 17.35, 28.01, 34.61, 36.31, 42.39, 45.75, 54.17, 56.27, 112.07, 122.48, 126.39, 127.29, 128.31, 129.10, 129.50, 129.86, 129.90, 130.24, 130.73, 132.33, 135.69, 140.92, 143.25, 146.42, 147.33, 152.15, 169.34, 171.98, 172.47, 173.17; ν_{max}/cm^{-1} (solid state) = ~3016 (COOH), 1738 (CO), 1365 (C-N), 1217 (C-O); ESI-HRMS found *m/z* 763.3108 [M-H]$^-$ $C_{44}H_{42}F_3N_4O_5$ requires 763.3113.

_N_-(_N_-(_N_-(4-Chlorobenzyl-4-aminobenzoyl)-_N_-(3-fluorobenzyl-4-aminobenzoyl)-_N_-2-methylbutyl-4-aminobenzoyl)-glycine 42

> 90% pure by NMR, isolated crude yield: 66 mg; δ_H (300 MHz, MeOD) 0.91 (t, 3H, *J* = 7.8 Hz, 3-Hδ), 0.93 (d, 3H, *J* = 6.9 Hz, 3-Hε), 1.19 (m, 1H, 3-Hβ), 1.45 (m, 1H, 3-Hγ), 1.61 (m, 1H, 3-Hγ'), 3.87 (d, 2H, *J* = 7.4 Hz, 3-Hα), 4.08 (s, 2H, 1-Hα), 4.33 (s, 2H, 2-Hα), 5.06 (s, 2H, 4-Hα), 6.38 (d, 2H, *J* = 8.5 Hz, 2-H3), 6.87 (d, 2H, *J* = 8.2 Hz, 3-H2), 6.93 (d, 2H, *J* = 7.6 Hz, ArCH), 6.97 (d, 2H, *J* = 8.2 Hz, ArCH), 7.02 (d, 2H, *J* = 8.5 Hz, ArCH), 7.14 (d, 2H, *J* = 8.2 Hz, ArCH), 7.15 (d, 2H, *J* = 8.2 Hz, ArCH), 7.25 (t, 1H, *J* = 7.8 Hz, 2-HAr5), 7.29 (d, 1H, *J* = 7.8 Hz, 2-HAr6), 7.31 (s, 1H, 2-HAr2), 7.41 (d, 1H, *J* = 7.7 Hz, 2-HAr4), 7.72 (d, 2H, *J* = 8.5 Hz, 1-H2); ESI-MS found *m/z* 733.4 [M-H]$^-$; ESI-HRMS found *m/z* 733.2605 [M-H]$^-$ $C_{42}H_{39}ClFN_4O_5$ requires 733.2598.

_N_-(_N_-(_N_-(4-Chlorobenzyl-4-aminobenzoyl)-_N_-4-chlorobenzyl-4-aminobenzoyl)-_N_-(2-methyl)butyl-4-aminobenzoyl)-glycine 43

> 75% pure by NMR, , isolated crude yield: 71 mg; δ_H (300 MHz, MeOD) 0.87-0.94 (m, 6H, 3-Hδ,ε), 1.19 (m, 1H, 3-Hβ), 1.44 (m, 1H, 3-Hγ), 1.60 (m, 1H, 3-Hγ'), 3.85 (d, 2H, *J* = 7.5 Hz, 3-Hα), 4.14 (s, 2H, 1-Hα), 4.33 (s, 2H, 2-Hα), 5.06 (s, 2H, 4-Hα), 6.37 (d, 2H, *J* = 8.8 Hz, 2-H3), 6.88 (d, 2H, *J* = 8.0 Hz, 3-H2), 7.12-7.18 (m, 6H, ArCH), 7.21-7.28 (m, 6H, ArCH), 7.29 (d, 2H, *J* = 8.8 Hz, ArCH), 7.72 (d, 2H, *J* = 8.2 Hz, 1-H2); ESI-MS found *m/z* 749.7 [M-H]$^-$; ESI-HRMS found *m/z* 749.2283 [M-H]$^-$ $C_{42}H_{39}Cl_2N_4O_5$ requires 749.2303.

_N_-(_N_-(_N_-(4-Fluorobenzyl-4-aminobenzoyl)-_N_-(4-trifluoromethyl)benzyl-4-aminobenzoyl)-_N_-isobutyl-4-aminobenzoyl)-glycine 45

> 90% pure by NMR, isolated crude yield: 71 mg; δ_H (300 MHz, MeOD) 0.94 (d, 6H, *J* = 6.6 Hz, 3-Hγ), 1.84 (m, 1H, 3-Hβ), 3.82 (d, 2H, *J* = 7.3 Hz, 3-Hα), 4.08 (s, 2H, 1-Hα), 4.36 (s, 2H, 2-Hα), 5.13 (s, 2H, 4-Hα), 6.48 (d, 2H, *J* = 8.8 Hz, 2-H3), 6.90 (d, 2H, *J* = 8.4 Hz, 3-H2), 7.02-7.08 (m, 4H, ArCH), 7.13-7.18

(m, 4H, ArCH), 7.34-7.37 (m, 2H, ArCH), 7.40 (d, 2H, *J* = 7.7 Hz, ArCH), 7.56 (d, 2H, *J* = 8.2 Hz, ArCH), 7.73 (d, 2H, *J* = 8.5 Hz, 1-H2); ESI-HRMS found *m/z* 753.2729 $[M-H]^-$ $C_{42}H_{37}F_4N_4O_5$ requires 753.2706.

***N*-(*N*-(*N*-(4-Fluorobenzyl-4-aminobenzoyl)-*N*-(4-trifluoromethyl)benzyl-4-aminobenzoyl)-*N*-cyclopropylmethyl-4-aminobenzoyl)-glycine 46**

> 90% pure by NMR, isolated crude yield: 65 mg; δ_H (300 MHz, MeOD) 0.14 (m, 2H, 3-Hγ), 0.45 (m, 2H, 3-Hγ'), 1.06 (m, 1H, 3-Hβ), 3.82 (d, 2H, *J* = 7.0 Hz, 3-Hα), 4.08 (s, 2H, 1-Hα), 4.35 (s, 2H, 2-Hα), 5.13 (s, 2H, 4-Hα), 6.45 (d, 2H, *J* = 8.8 Hz, 2-H3), 6.89 (d, 2H, *J* = 8.4 Hz, 3-H2), 7.02-7.10 (m, 4H, ArCH), 7.16-7.21 (m, 4H, 2-H2 and 3-H3), 7.34 (m, 2H, ArCH), 7.40 (d, 2H, *J* = 7.7 Hz, ArCH), 7.56 (d, 2H, *J* = 8.0 Hz, 2-HAr3), 7.75 (d, 2H, *J* = 8.5 Hz, 1-H2); ESI-HRMS found *m/z* 751.2535 $[M-H]^-$ $C_{42}H_{37}F_4N_4O_5$ requires 751.2549.

***N*-(*N*-(*N*-(Benzyl-4-aminobenzoyl)-*N*-(3-trifluoromethyl)benzyl-4-aminobenzoyl)-*N*-propyl-4-aminobenzoyl)-glycine 47**

> 90% pure by NMR, isolated crude yield: 69 mg; δ_H (300 MHz, MeOD) 0.94 (t, 3H, *J* = 7.4 Hz, 3-Hγ), 1.61 (m, 2H, 3-Hβ), 3.89 (t, 2H, *J* = 7.5 Hz, 3-Hα), 4.07 (s, 2H, 1-Hα), 4.37 (s, 2H, 2-Hα), 5.14 (s, 2H, 4-Hα), 6.51 (d, 2H, *J* = 8.8 Hz, 2-H3), 6.87 (d, 2H, *J* = 8.4 Hz, 3-H2), 7.06 (d, 2H, *J* = 8.8 Hz, 2-H2), 7.12-7.17 (m, 6H, ArCH), 7.32-7.35 (m, 4H, ArCH), 7.43 (t, 1H, *J* = 7.8 Hz, 2-HAr4), 7.53 (d, 1H, *J* = 8.1 Hz, ArCH), 7.59 (s, 1H, 2-HAr2), 7.73 (d, 2H, *J* = 8.5 Hz, 1-H2); ESI-HRMS found *m/z* 721.2651 $[M-H]^-$ $C_{41}H_{36}F_3N_4O_5$ requires 721.2643.

***N*-(*N*-(*N*-(Cyclohexylmethyl-4-aminobenzoyl)-*N*-naphth-2-yl-4-aminobenzoyl)-*N*-phenethyl-4-aminobenzoyl)-glycine 48**

> 85% pure by NMR, isolated crude yield: 75 mg; δ_H (300 MHz, $CDCl_3$) 1.04 (m, 2H, 1-Hε), 1.23-1.36 (m, 4H, 1-Hδ,δ'), 1.65-1.89 (m, 5H, 1-Hγ,γ',β), 2.93 (t, 2H, *J* = 8.2 Hz, 3-Hβ), 2.99 (d, 2H, *J* = 6.9 Hz, 1-Hα), 4.10 (t, 2H, *J* = 8.2 Hz, 3-Hα), 4.13 (s, 2H, 2-Hα), 5.25 (s, 2H, 4-Hα), 6.56 (d, 2H, *J* = 8.2 Hz, 2-H3), 6.91 (d, 2H, *J* = 8.3 Hz, 3-H2), 6.93 (d, 2H, *J* = 8.2 Hz, 2-H2), 7.07 (d, 2H, *J* = 8.3 Hz, 3-H3), 7.13 (d, 2H, *J* = 8.7 Hz, 1-H3), 7.16-7.20 (m, 2H, 3-HAr2), 7.21-7.28 (m, 3H, 3-HAr3,4), 7.36 (d, 1H, *J* = 8.2 Hz, ArCH), 7.44-7.48 (m, 2H, ArCH), 7.64 (s, 1H, 2-HAr1), 7.66 (d, 2H, *J* = 8.7 Hz, 1-H2), 7.73-7.83 (m, 3H, ArCH); ESI-MS found *m/z* 773.4 $[M+H]^+$; ESI-HRMS found *m/z* 771.3543 $[M-H]^-$ $C_{49}H_{47}N_4O_5$ requires 771.3552.

_N_-(_N_-(_N_-(Benzyl-4-aminobenzoyl)-_N_-(3-trifluoromethyl)benzyl-4-aminobenzoyl)-_N_-cyclopropylmethyl-4-aminobenzoyl)-glycine 49

> 75% pure by NMR, isolated crude yield: 70 mg; δ_H (500 MHz, $CDCl_3$) 0.17 (m, 2H, 3-Hγ), 0.46 (m, 2H, 3-Hγ'), 1.05 (m, 1H, 3-Hβ), 3.81 (d, 2H, *J* = 7.1 Hz, 3-Hα), 3.99 (d, 2H, *J* = 4.2 Hz, 1-Hα), 4. (s, 2H, 2-Hα), 5.12 (s, 2H, 4-Hα), 6.74 (d, 2H, *J* = 8.2 Hz, 2-H3), 7.03 (d, 2H, *J* = 8.0 Hz, 3-H2), 7.12 (d, 2H, *J* = 8.0 Hz, ArCH), 7.14 (d, 2H, *J* = 8.0 Hz, ArCH), 7.25 (d, 2H, *J* = 8.5 Hz, ArCH), 7.34-7.37 (m, 5H, 1-HAr2-4), 7.39 (d, 2H, *J* = 8.0 Hz, 1-H3), 7.52 (m, 2H, ArCH), 7.60 (d, 2H, *J* = 8.0 Hz, 1-H2), 7.75 (m, 1H, Ar-NH); ESI-HRMS found *m/z* 733.2672 $[M-H]^-$ $C_{42}H_{36}F_3N_4O_5$ requires 733.2643.

_N_-(_N_-(_N_-(Phenethyl-4-aminobenzoyl)-_N_-3-fluorobenzyl-4-aminobenzoyl)-_N_-2-isobutyl-4-aminobenzoyl)-glycine 50

> 80% pure by NMR, isolated crude yield: 49 mg; δ_H (300 MHz, MeOD) 0.93 (d, 6H, *J* = 6.6 Hz, 3-Hγ), 1.78 (m, 1H, 3-Hβ), 2.83 (t, 2H, *J* = 7.7 Hz, 1-Hβ), 3.83 (d, 2H, *J* = 7.7 Hz, 3-Hα), 4.06 (t, 2H, *J* = 7.7 Hz, 1-Hα), 4.18 (s, 2H, 2-Hα), 5.07 (s, 2H, 4-Hα), 6.39 (d, 2H, *J* = 8.8 Hz, 2-H3), 6.88 (d, 2H, *J* = 8.2 Hz, 3-H2), 7.12 (d, 2H, *J* = 8.5 Hz, ArCH), 7.15 (d, 2H, *J* = 8.2 Hz, ArCH), 7.19 (d, 2H, *J* = 8.5 Hz, ArCH), 7.23-7.30 (m, 7H, ArCH), 7.71 (d, 2H, *J* = 8.2 Hz, 1-H2), 7.75-7.80 (m, 2H, ArCH); ESI-HRMS found *m/z* 699.2986 $[M-H]^-$ $C_{42}H_{40}FN_4O_5$ requires 699.2988.

_N_-(_N_-(_N_-(4-Chlorobenzyl-4-aminobenzoyl)-_N_-3-fluorobenzyl-4-aminobenzoyl)-_N_-isobutyl-4-aminobenzoyl)-glycine 51

> 85% pure by NMR, isolated crude yield: 61 mg; δ_H (300 MHz, MeOD) 0.95 (d, 6H, *J* = 5.9 Hz, 3-Hγ), 1.84 (m, 1H, 3-Hβ), 3.83 (d, 2H, *J* = 6.9 Hz, 3-Hα), 4.08 (s, 2H, 1-Hα), 4.33 (s, 2H, 2-Hα), 5.06 (s, 2H, 4-Hα), 6.36 (d, 2H, *J* = 8.7 Hz, 2-H3), 6.87 (d, 2H, *J* = 8.7 Hz, 3-H2), 6.95 (d, 2H, *J* = 8.2 Hz, ArCH), 7.01 (d, 2H, *J* = 7.7 Hz, ArCH), 7.11-7.17 (m, 5H, ArCH), 7.23 (t, 1H, *J* = 7.8 Hz, 2-HAr5), 7.28-7.31 (m, 4H, 1-HAr2,3), 7.72 (d, 2H, *J* = 8.2 Hz, 1-H2); ESI-HRMS found *m/z* 719.2463 $[M-H]^-$ $C_{41}H_{37}ClFN_4O_5$ requires 719.2442.

_N_-(_N_-(_N_-(4-Chlorobenzyl-4-aminobenzoyl)-_N_-4-fluorobenzyl-4-aminobenzoyl)-_N_-isobutyl-4-aminobenzoyl)-glycine 52

> 85% pure by NMR, isolated crude yield: 60 mg; δ_H (300 MHz, MeOD) 0.95 (d, 6H, *J* = 6.1 Hz, 3-Hγ), 1.85 (m, 1H, 3-Hβ), 3.83 (d, 2H, *J* = 7.1 Hz, 3-Hα), 4.08 (s, 2H, 1-Hα), 4.32 (s, 2H, 2-Hα), 5.03 (s, 2H, 4-Hα), 6.35 (d, 2H, *J* = 8.2 Hz, 2-H3), 6.83-6.88 (m, 4H, ArCH), 6.97 (d, 2H, *J* = 7.1 Hz, ArCH), 6.99 (d, 2H, *J* = 7.7 Hz, ArCH), 7.12-7.21 (m, 6H, ArCH), 7.30 (d, 2H, *J* = 7.0 Hz, ArCH), 7.72 (d, 2H, *J* = 7.1 Hz, 1-H2); ESI-HRMS found *m/z* 719.2432 $[M-H]^-$ $C_{41}H_{37}ClFN_4O_5$ requires 719.2442.

***N*-(*N*-(*N*-(Phenethyl-4-aminobenzoyl)-*N*-4-fluorobenzyl-4-aminobenzoyl)-*N*-isobutyl-4-aminobenzoyl)-glycine 53**

> 90% pure by NMR, isolated crude yield: 61 mg; δ_H (300 MHz, MeOD) 0.94 (d, 6H, *J* = 6.6 Hz, 3-Hγ), 1.84 (m, 1H, 3-Hβ), 2.91 (t, 2H, *J* = 7.4 Hz, 1-Hβ), 3.39 (t, 2H, *J* = 7.4 Hz, 1-Hα), 3.82 (d, 2H, *J* = 7.4 Hz, 3-Hα), 4.11 (s, 2H, 1-Hα), 5.04 (s, 2H, 4-Hα), 6.51 (d, 2H, *J* = 8.8 Hz, 2-H3), 6.86 (d, 2H, *J* = 8.5 Hz, 3-H2), 6.98 (t, 2H, *J* = 8.8 Hz, ArCH), 7.08 (d, 2H, *J* = 8.8 Hz, 2-H2), 7.14 (d, 2H, *J* = 8.5 Hz, 3-H3), 7.16-7.23 (m, 5H, ArCH), 7.24-7.27 (m, 2H, ArCH), 7.29 (d, 2H, *J* = 8.1 Hz, 1-H3), 7.73 (d, 2H, *J* = 8.5 Hz, 1-H2); ESI-HRMS found *m/z* 699.2981 $[M-H]^-$ $C_{42}H_{40}FN_4O_5$ requires 699.2988.

***N*-(*N*-(*N*-(Phenethyl-4-aminobenzoyl)-*N*-(2-methyl)naphth-1-yl-4-aminobenzoyl)-*N*-isobutyl-4-aminobenzoyl)-glycine 54**

> 95% pure by NMR, isolated crude yield: 58 mg; δ_H (300 MHz, MeOD) 0.90 (d, 6H, *J* = 6.6 Hz, 3-Hγ), 1.79 (m, 1H, 3-Hβ), 2.89 (t, 2H, *J* = 7.4 Hz, 1-Hβ), 3.36 (t, 2H, *J* = 7.4 Hz, 1-Hα), 3.75 (d, 2H, *J* = 7.4 Hz, 3-Hα), 4.15 (s, 2H, 1-Hα), 4.90 (s, 3H, 2-Me), 5.54 (s, 2H, 4-Hα), 6.43 (d, 2H, *J* = 8.2 Hz, 2-H3), 6.53 (d, 2H, *J* = 8.5 Hz, ArCH), 6.90 (d, 2H, *J* = 8.2 Hz, ArCH), 7.02 (d, 2H, *J* = 8.8 Hz, ArCH), 7.06 (d, 2H, *J* = 8.8 Hz, ArCH), 7.14 (d, 1H, *J* = 8.5 Hz, ArCH), 7.19-7.29 (m, 5H, ArCH), 7.48 (m, 1H, ArCH), 7.69 (d, 2H, *J* = 8.2 Hz, ArCH), 7.73 (d, 2H, *J* = 8.5 Hz, ArCH), 7.82 (d, 1H, *J* = 8.2 Hz, ArCH), 8.13 (d, 1H, *J* = 8.5 Hz, ArCH); ESI-HRMS found *m/z* 745.3383 $[M-H]^-$ $C_{47}H_{45}N_4O_5$ requires 745.3395.

***N*-(*N*-(*N*-(4-Chlorobenzyl-4-aminobenzoyl)-*N*-naphth-2-yl-4-aminobenzoyl)-*N*-isobutyl-4-aminobenzoyl)-glycine 55**

> 80% pure by NMR, isolated crude yield: 71 mg; δ_H (500 MHz, MeOD) 0.94 (d, 6H, *J* = 6.4 Hz, 3-Hγ), 1.83 (m, 1H, 1-Hβ), 3.81 (d, 2H, *J* = 7.3 Hz, 3-Hα), 4.10 (s, 2H, 1-Hα), 4.35 (s, 2H, 2-Hα), 5.24 (s, 2H, 4-Hα), 6.41 (d, 2H, *J* = 8.7 Hz, 2-H3), 6.90 (d, 2H, *J* = 8.2 Hz, 3-H2), 7.06 (d, 2H, *J* = 8.7 Hz, 2-H2), 7.11 (d, 2H, *J* = 8.2 Hz, ArCH), 7.14 (d, 2H, *J* = 7.8 Hz, ArCH), 7.30-7.36 (m, 4H, ArCH), 7.39 (m, 1H, ArCH), 7.45 (d, 2H, *J* = 7.8 Hz, ArCH), 7.64 (s, 1H, 2-HAr2), 7.70 (d, 2H, *J* = 8.2 Hz, ArCH), 7.77 (d, 2H, *J* = 8.7 Hz, ArCH), 7.81 (d, 1H, *J* = 8.2 Hz, ArCH); ESI-HRMS found *m/z* 751.2664 $[M-H]^-$ $C_{45}H_{40}ClN_4O_5$ requires 751.2693.

***N*-(*N*-(*N*-(Propyl-4-aminobenzoyl)-*N*-phenethyl-4-aminobenzoyl)-*N*-cyclohexylmethyl-4-aminobenzoyl)-valine 58**

> 85% pure by NMR, isolated crude yield: 70 mg; δ_H (300 MHz, $CDCl_3$) 0.97-1.17 (m, 5H, 3-Hδ,δ′,ε,), 1.02 (d, 6H, *J* = 7.4 Hz, 4-Hγ), 1.11 (t, 3H, *J* = 7.2 Hz, 1-Hγ), 1.61-1.77 (m, 7H, 1-Hβ, 3-Hγ,γ′,β), 1.86 (m, 1H, 4-Hβ), 2.95 (m, 2H, 2-Hβ), 3.29 (t, 2H, *J* = 8.2 Hz, 1-Hα), 3.70 (dd, 1H, J_1 = 13.8 Hz, J_2 = 6.6 Hz, 3-

Hα), 4.00 (m, 2H, 2-Hα), 4.26 (m, 1H, 3-Hα’), 4.34 (d, 1H, *J* = 6.6 Hz, 4-Hα), 6.81 (d, 2H, *J* = 7.2 Hz, 2-H3), 7.03 (d, 2H, *J* = 8.4 Hz, 3-H2), 7.19 (d, 2H, *J* = 8.4 Hz, 3-H3), 7.25 (d, 2H, *J* = 7.2 Hz, 2-H2), 7.28-7.32 (m, 5H, 2-HAr2-4), 7.37 (d, 2H, *J* = 8.5 Hz, 1-H3), 7.59 (d, 2H, *J* = 8.5 Hz, 1-H2); ESI-MS found *m/z* 715.3 [M-H]$^-$; ESI-HRMS found *m/z* 715.3853 [M-H]$^-$ $C_{44}H_{51}N_4O_5$ requires 715.3865.

N-(N-(N-(Propyl-4-aminobenzoyl)-N-cyclohexylmethyl-4-aminobenzoyl)-N-propyl-4-aminobenzoyl)-isoleucine 59

> 90% pure by NMR, isolated crude yield: 61 mg; δ_H (300 MHz, $CDCl_3$) 0.93-1.05 (m, 15H, 1-Hγ, 2-Hδ,δ’,ε, 3-Hγ, 4-Hγ), 1.08 (d, 3H, *J* = 6.9 Hz, 4-Hε), 1.11-1.16 (m, 2H, 4-Hγ), 1.30 (m, 1H, 3-Hβ), 1.60-1.70 (m, 7H, 1-Hβ, 2-Hβ,γ,γ’), 1.83 (m, 1H, 2-Hβ), 2.07 (m, 1H, 4-Hβ), 3.29 (t, 2H, *J* = 7.9 Hz, 3-Hα), 3.59 (dd, 1H, J_1 = 13.4 Hz, J_2 = 6.6 Hz, 2-Hα), 3.63 (m, 1H, 2-Hα’), 4.03 (m, 2H, 1-Hα), 4.47 (d, 1H, *J* = 6.6 Hz, 4-Hα), 6.96 (d, 2H, *J* = 7.2 Hz, 2-H3), 7.06 (d, 2H, *J* = 8.2 Hz, 3-H2), 7.07 (d, 2H, *J* = 7.2 Hz, 2-H2), 7.27 (d, 2H, *J* = 8.5 Hz, 3-H3), 7.32 (d, 2H, *J* = 8.3 Hz, 1-H3), 7.59 (d, 2H, *J* = 8.3 Hz, 1-H2); ESI-MS found *m/z* 667.3 [M-H]$^-$; ESI-HRMS found *m/z* 667.3867 [M-H]$^-$ $C_{40}H_{51}N_4O_5$ requires 667.3865.

N-(N-(N-(3-Fluorobenzyl-4-aminobenzoyl)-N-phenethyl-4-aminobenzoyl)-N-propyl-4-aminobenzoyl)-leucine 60

> 90% pure by NMR, isolated crude yield: 70 mg; δ_H (300 MHz, $CDCl_3$) 0.93 (t, 3H, *J* = 7.4 Hz, 3-Hγ), 0.94 (d, 6H, *J* = 7.4 Hz, 4-Hδ), 1.55-1.72 (m, 5H, 3-Hβ, 4-Hβ,γ), 2.91 (t, 2H, *J* = 7.2 Hz, 2-Hβ), 3.82 (m, 1H, 3-Hα), 3.98 (t, 2H, *J* = 7.4 Hz, 2-Hα), 4.20 (m, 1H, 3-Hα’), 4.34 (s, 2H, 1-Hα), 4.44 (m, 1H, 4-Hα), 6.63 (d, 2H, *J* = 7.9 Hz, 2-H3), 6.98 (d, 2H, *J* = 8.2 Hz, 3-H2), 7.10 (d, 2H, *J* = 7.9 Hz, 2-H2), 7.11 (d, 2H, *J* = 8.2 Hz, 3-H3), 7.13-7.19 (m, 7H, ArCH), 7.23 (d, 1H, *J* = 6.9 Hz, 1-HAr4), 7.27 (d, 2H, *J* = 8.1 Hz, 1-H3), 7.30 (m, 1H, 1-HAr5), 7.59 (d, 2H, *J* = 8.1 Hz, 1-H2); ESI-MS found *m/z* 741.2 [M-H]$^-$; ESI-HRMS found *m/z* 741.3456 [M-H]$^-$ $C_{45}H_{46}FN_4O_5$ requires 741.3458.

N-(N-(N-(2-Methylbutyl-4-aminobenzoyl)-N-(2-methyl)butyl-4-aminobenzoyl)-N-2-methylbutyl-4-aminobenzoyl)-isoleucine 61

> 95% pure by NMR, isolated crude yield: 63 mg; δ_H (300 MHz, MeOD) 0.71-0.81 (m, 9H, 1-Hδ, 2-Hδ, 4-Hδ), 0.83-0.89 (m, 9H, 1-Hε, 3-Hγ), 0.91-0.95 (m, 6H, 2-Hε, 4-Hε), 1.03-1.15 (m, 3H, 1-Hγ, 1-Hβ), 1.22-1.33 (m, 2H, 2-Hγ), 1.37-1.45 (m, 4H, 4-Hγ, 2-Hβ, 3-Hβ), 1.56-1.69 (m, 1H, 4-Hβ), 2.89 (dd, 1H, J_1 = 12.6 Hz and J_2 = 7.4 Hz, 1-Hα), 3.02 (dd, 1H, J_1 = 12.6 Hz and J_2 = 7.4 Hz, 1-Hα’), 3.68 (d, 2H, *J* = 7.1 Hz, 3-Hα), 3.79 (d, 2H, *J* = 7.4 Hz, 2-Hα), 4.45 (d, 1H, *J* = 6.4 Hz, 4-Hα), 6.55 (d, 2H, *J* = 8.0 Hz, 2-H3), 6.91 (d, 2H, *J* = 8.5 Hz, 3-H2), 6.97 (d, 2H, *J* = 8.5 Hz, 2-H2), 7.10 (d, 2H, *J* = 8.5 Hz, 3-H3), 7.11 (d, 2H, *J*

= 8.5 Hz, 1-H3), 7.63 (d, 2H, *J* = 8.5 Hz, 1-H2); ESI-HRMS found *m/z* 697.4356 [M-H]$^-$ $C_{42}H_{57}N_4O_5$ requires 697.4334.

Valine-*N*-(*N*-(*N*-(isobutyl-4-aminobenzoyl)-*N*-(isobutyl-4-aminobenzoyl)-*N*-isobutyl-4-aminobenzoyl)-glycine 62

> 90% pure by NMR, isolated crude yield: 62 mg; δ_H (300 MHz, MeOD) 0.80 (d, 6H, *J* = 7.1 Hz, 2-Hγ), 0.87 (d, 6H, *J* = 7.1 Hz, 3-Hγ), 0.92 (d, 6H, *J* = 6.6 Hz, 4-Hγ), 0.96 (d, 6H, *J* = 6.6 Hz, 1-Hγ), 1.66-1.80 (m, 2H, 2-Hβ, 3-Hβ), 1.84-1.98 (m, 2H, 1-Hβ, 4-Hβ), 3.71-3.80 (m, 4H, 3-Hα, 4-Hα), 3.83-3.86 (m, 2H, 2-Hα), 4.07-4.11 (m, 1H, 1-Hα), 4.09 (s, 2H, 5-Hα), 7.04 (d, 2H, *J* = 8.5 Hz, 2-H3), 7.18 (d, 2H, *J* = 7.9 Hz, 3-H2), 7.21 (d, 2H, *J* = 7.9 Hz, 3-H3), 7.25 (d, 2H, *J* = 8.5 Hz, 2-H2), 7.34 (d, 2H, *J* = 8.3 Hz, 1-H3), 7.77 (d, 2H, *J* = 8.3 Hz, 1-H2); ESI-MS found *m/z* 700.5 [M+H]$^+$; ESI-HRMS found *m/z* 698.3916 [M-H]$^-$ $C_{40}H_{52}N_5O_6$ requires 698.3923.

***N*-(*N*-(*N*-(Isobutyl-4-aminobenzoyl)-*N*-(2-methylbutyl-4-aminobenzoyl)-*N*-2-methylbutyl-4-aminobenzoyl)-isoleucine 63**

> 95% pure by NMR, isolated crude yield: 63 mg; δ_H (300 MHz, MeOD) 0.83 (t, 3H, *J* = 7.4 Hz, 3-Hδ), 0.87 (d, 3H, *J* = 6.6 Hz, 3-Hε), 0.89 (t, 3H, *J* = 7.4 Hz, 2-Hδ), 0.94 (d, 3H, *J* = 6.9 Hz, 2-Hε), 0.97 (d, 3H, *J* = 7.7 Hz, 4-Hε), 1.02 (d, 6H, *J* = 6.6 Hz, 1-Hγ), 1.03 (t, 3H, *J* = 6.6 Hz, 4-Hδ), 1.09-1.26 (m, 2H, 3-Hγ), 1.29-1.41 (m, 2H, 2-Hγ), 1.43-1.54 (m, 2H, 4-Hγ), 1.56-1.68 (m, 2H, 3-Hβ, 1-Hβ), 1.95 (m, 1H, 2-Hβ), 2.04 (m, 1H, 4-Hβ), 3.00 (d, 2H, *J* = 6.9 Hz, 1-Hα), 3.78 (d, 2H, *J* = 7.1 Hz, 2-Hα), 3.89 (d, 2H, *J* = 7.4 Hz, 3-Hα), 4.55 (d, 1H, *J* = 6.3 Hz, 4-Hα), 6.60 (d, 2H, *J* = 8.7 Hz, 2-H3), 7.01 (d, 2H, *J* = 8.2 Hz, 3-H2), 7.05 (d, 2H, *J* = 8.7 Hz, 2-H2), 7.20 (d, 2H, *J* = 8.2 Hz, 3-H3), 7.21 (d, 2H, *J* = 8.5 Hz, 1-H3), 7.74 (d, 2H, *J* = 8.5 Hz, 1-H2); ESI-MS found *m/z* 683.5 [M-H]$^-$; ESI-HRMS found *m/z* 683.4182 [M-H]$^-$ $C_{41}H_{55}N_4O_5$ requires 683.4178.

***N*-(*N*-(*N*-(Isobutyl-4-aminobenzoyl)-*N*-naphth-2-yl-4-aminobenzoyl)-*N*-2-methylbutyl-4-aminobenzoyl)-isoleucine 64**

> 95% pure by NMR, isolated crude yield: 70 mg; δ_H (300 MHz, MeOD) 0.85 (t, 3H, *J* = 7.4 Hz, 3-Hδ), 0.89 (d, 3H, *J* = 6.9 Hz, 3-Hε), 0.98 (t, 3H, *J* = 7.4 Hz, 4-Hδ), 1.02 (d, 6H, *J* = 7.2 Hz, 1-Hγ), 1.04 (d, 3H, *J* = 7.4 Hz, 4-Hε), 1.15 (m, 1H, 3-Hγ), 1.30-1.47 (m, 2H, 3-Hγ', 4-Hγ), 1.54-1.68 (m, 2H, 4-Hγ', 1-Hβ), 1.94 (m, 1H, 3-Hβ), 2.03 (m, 1H, 4-Hβ), 2.99 (d, 2H, *J* = 6.9 Hz, 1-Hα), 3.84 (d, 2H, *J* = 7.1 Hz, 3-Hα), 4.58 (d, 1H, *J* = 6.3 Hz, 4-Hα), 5.24 (s, 2H, 2-Hα), 6.59 (d, 2H, *J* = 8.7 Hz, 2-H3), 6.90 (d, 2H, *J* = 8.4 Hz, 3-H2), 7.11 (d, 2H, *J* = 8.7 Hz, 2-H2), 7.14 (d, 2H, *J* = 8.4 Hz, 3-H3), 7.15 (d, 2H, *J* = 8.5 Hz, 1-H3), 7.36 (m, 1H, ArCH), 7.43-7.46 (m, 2H, ArCH), 7.64 (s, 1H, 3-HAr2), 7.70 (d, 2H, *J* = 8.5 Hz, 1-H2), 7.74-7.78

(m, 2H, ArCH), 7.81 (m, 1H, 2- ArCH); ESI-MS found *m/z* 755.5 $[M+H]^+$; ESI-HRMS found *m/z* 753.4007 $[M-H]^-$ $C_{47}H_{53}N_4O_5$ requires 753.4021.

_N_-(_N_-(_N_-(Isobutyl-4-aminobenzoyl)-_N_-2-methylbutyl-4-aminobenzoyl)-_N_-naphth-2-yl-4-aminobenzoyl)-valine 65

> 95% pure by NMR, isolated crude yield: 73 mg; δ_H (300 MHz, MeOD) 0.83 (t, 3H, *J* = 7.4 Hz, 2-Hδ), 0.86 (d, 3H, *J* = 6.6 Hz, 2-Hε), 0.99 (d, 6H, *J* = 6.7 Hz, 1-Hγ), 1.01 (d, 6H, *J* = 6.8 Hz, 4-Hγ), 1.14 (m, 1H, 2-Hγ), 1.37 (m, 1H, 2-Hγ'), 1.53 (m, 1H, 1-Hβ), 1.91 (m, 1H, 2-Hβ), 2.23 (m, 1H, 4-Hβ), 2.96 (d, 2H, *J* = 6.9 Hz, 1-Hα), 3.76 (d, 2H, *J* = 7.2 Hz, 2-Hα), 4.43 (d, 1H, *J* = 6.3 Hz, 4-Hα), 5.36 (s, 2H, 3-Hα), 6.56 (d, 2H, *J* = 8.5 Hz, 2-H3), 7.01 (d, 2H, *J* = 8.5 Hz, 3-H2), 7.04 (d, 2H, *J* = 8.5 Hz, 2-H2), 7.09 (d, 2H, *J* = 8.5 Hz, 3-H3), 7.32 (d, 2H, *J* = 8.5 Hz, 1-H3), 7.43-7.51 (m, 3H, ArCH), 7.63 (d, 2H, *J* = 8.5 Hz, 1-H2), 7.69 (s, 1H, ArCH), 7.76 (m, 1H, ArCH), 7.81-7.83 (m, 2H, ArCH); ESI-MS found *m/z* 740.1 $[M-H]^-$; ESI-HRMS found *m/z* 739.3878 $[M-H]^-$ $C_{46}H_{51}N_4O_5$ requires 739.3865.

_N_-(_N_-(_N_-(Isobutyl-4-aminobenzoyl)-_N_-2-methylbutyl-4-aminobenzoyl)-_N_-4-chlorobenzyl-4-aminobenzoyl)-valine 66

> 95% pure by NMR, isolated crude yield: 69 mg; δ_H (300 MHz, MeOD) 0.83 (t, 3H, *J* = 7.2 Hz, 2-Hδ), 0.86 (d, 3H, *J* = 7.2 Hz, 2-Hε), 1.01 (d, 6H, *J* = 6.9 Hz, 1-Hγ), 1.04 (d, 6H, *J* = 7.4 Hz, 4-Hγ), 1.13 (m, 1H, 2-Hγ), 1.39 (m, 1H, 2-Hγ'), 1.52 (m, 1H, 1-Hβ), 1.94 (m, 1H, 2-Hβ), 2.28 (m, 1H, 4-Hβ), 2.98 (d, 2H, *J* = 6.9 Hz, 1-Hα), 3.78 (d, 2H, *J* = 6.9 Hz, 2-Hα), 4.46 (d, 1H, *J* = 6.3 Hz, 4-Hα), 5.17 (s, 2H, 3-Hα), 6.59 (d, 2H, *J* = 8.5 Hz, 2-H3), 7.01 (d, 2H, *J* = 8.2 Hz, 3-H2), 7.03 (d, 2H, *J* = 8.5 Hz, 2-H2), 7.05 (d, 2H, *J* = 8.2 Hz, 3-H3), 7.28 (d, 2H, *J* = 8.2 Hz, 1-H3), 7.28-7.30 (m, 4H, 3-HAr2,3), 7.67 (d, 2H, *J* = 8.5 Hz, 1-H2); ESI-MS found *m/z* 724.8 $[M+H]^+$; ESI-HRMS found *m/z* 723.3346 $[M-H]^-$ $C_{42}H_{48}ClN_4O_5$ requires 723.3319.

_N_-(_N_-(_N_-(Isobutyl-4-aminobenzoyl)-_N_-4-chlorobenzyl-4-aminobenzoyl)-_N_-isobutyl-4-aminobenzoyl)-isoleucine 67

> 95% pure by NMR, isolated crude yield: 61 mg; δ_H (300 MHz, MeOD) 0.92 (d, 6H, *J* = 6.6 Hz, 1-Hγ), 0.98 (t, 3H, *J* = 7.8 Hz, 4-Hδ), 1.01 (d, 6H, *J* = 6.9 Hz, 3-Hγ), 1.04 (d, 3H, *J* = 6.9 Hz, 4-Hε), 1.35 (m, 1H, 1-Hβ), 1.63 (m, 1H, 3-Hβ), 1.85 (m, 1H, 4-Hγ), 1.94 (m, 1H, 4-Hγ'), 2.05 (m, 1H, 4-Hβ), 2.98 (d, 2H, *J* = 6.9 Hz, 1-Hα), 3.82 (d, 2H, *J* = 7.4 Hz, 3-Hα), 4.57 (d, 1H, *J* = 6.1 Hz, 4-Hα), 5.03 (s, 2H, 2-Hα), 6.55 (d, 2H, *J* = 8.5 Hz, 2-H3), 6.88 (d, 2H, *J* = 8.2 Hz, 3-H2), 7.11 (d, 2H, *J* = 8.5 Hz, 2-H2), 7.15 (d, 2H, *J* = 8.2 Hz, 3-H3), 7.17-7.20 (m, 4H, 2-HAr2,3), 7.26 (d, 2H, *J* = 8.5 Hz, 1-H3), 7.74 (d, 2H, *J* = 8.5 Hz, 1-H2);

ESI-MS found *m/z* 723.8 $[M-H]^-$; ESI-HRMS found *m/z* 723.3324 $[M-H]^-$ $C_{42}H_{48}ClN_4O_5$ requires 723.3319.

_N_-(_N_-(_N_-(Carboxymethyl-4-aminobenzoyl)-_N_-2-methylbutyl-4-aminobenzoyl)-_N_-isobutyl-4-aminobenzoyl)-valine 68

> 80% pure by NMR, isolated crude yield: 56 mg; δ_H (300 MHz, MeOD) 0.82 (t, 3H, *J* = 7.4 Hz, 2-Hδ), 0.85 (d, 3H, *J* = 5.9 Hz, 2-Hε), 0.96 (d, 6H, *J* = 6.6 Hz, 3-Hγ), 1.05 (d, 6H, *J* = 6.6 Hz, 4-Hγ), 1.03-1.08 (m, 2H, 2-Hγ), 1.51 (m, 1H, 2-Hβ), 1.87 (m, 1H, 3-Hβ), 2.30 (m, 1H, 4-Hβ), 3.76 (m, 2H, 2-Hα), 3.85 (d, 2H, *J* = 7.4 Hz, 3-Hα), 3.91 (s, 2H, 1-Hα), 4.52 (m, 1H, 4-Hα), 6.37 (d, 2H, *J* = 8.5 Hz, 2-H3), 6.98 (d, 4H, *J* = 8.2 Hz, 3-H2, 2-H2), 7.20 (d, 4H, *J* = 8.0 Hz, 3-H3, 1-H3), 7.77 (d, 2H, *J* = 8.5 Hz, 1-H2); ESI-HRMS found *m/z* 657.3280 $[M-H]^-$ $C_{37}H_{45}N_4O_7$ requires 657.3294.

_N_-(_N_-(_N_-(Carboxymethyl-4-aminobenzoyl)-_N_-2-methylbutyl-4-aminobenzoyl)-_N_-2-methylbutyl-4-aminobenzoyl)-valine 69

> 80% pure by NMR, isolated crude yield: 52 mg; δ_H (300 MHz, MeOD) 0.86 (t, 3H, *J* = 7.1 Hz, 2-Hδ), 0.88 (t, 3H, *J* = 7.1 Hz, 3-Hδ), 0.86-0.90 (m, 2H, 3-Hγ), 0.94 (d, 6H, *J* = 6.9 Hz, 3-Hε, 2-Hε), 1.05 (d, 6H, *J* = 6.3 Hz, 4-Hγ), 1.03-1.08 (m, 2H, 2-Hγ), 1.49 (m, 1H, 3-Hβ), 1.61 (m, 1H, 2-Hβ), 2.29 (m, 1H, 4-Hβ), 3.77 (m, 2H, 2-Hα), 3.82 (s, 2H, 1-Hα), 3.90 (d, 2H, *J* = 7.2 Hz, 3-Hα), 4.52 (m, 1H, 4-Hα), 6.35 (d, 2H, *J* = 8.5 Hz, 2-H3), 6.98 (d, 2H, *J* = 8.3 Hz, 3-H2), 7.04 (d, 2H, *J* = 8.5 Hz, 2-H2), 7.20 (d, 2H, *J* = 8.3 Hz, 3-H3), 7.21 (d, 2H, *J* = 8.3 Hz, 1-H3), 7.78 (d, 2H, *J* = 8.3 Hz, 1-H2); ESI-HRMS found *m/z* 671.3423 $[M-H]^-$ $C_{38}H_{47}N_4O_7$ requires 671.3450.

_N_-(_N_-(_N_-(Isobutyl-4-aminobenzoyl)-_N_-(2-methyl)butyl-4-aminobenzoyl)-_N_-isobutyl-4-aminobenzoyl)-aspartic acid 70

> 90% pure by NMR, isolated crude yield: 52 mg; δ_H (300 MHz, MeOD) 0.84 (t, 3H, *J* = 7.4 Hz, 2-Hδ), 0.88 (d, 3H, *J* = 6.6 Hz, 2-Hε), 0.96 (d, 6H, *J* = 6.6 Hz, 1-Hγ), 1.04 (d, 6H, *J* = 6.6 Hz, 3-Hγ), 1.14 (m, 2H, 2-Hγ), 1.40 (m, 1H, 2-Hβ), 1.87 (m, 1H, 3-Hβ), 1.97 (m, 1H, 1-Hβ), 2.90 (m, 2H, 4-Hβ), 3.02 (d, 2H, *J* = 6.9 Hz, 2-Hα), 3.77 (d, 2H, *J* = 7.4 Hz, 3-Hα), 3.84 (d, 2H, *J* = 7.4 Hz, 1-Hα), 4.98 (m, 1H, 4-Hα), 6.65 (d, 2H, *J* = 8.5 Hz, 2-H3), 7.01 (d, 2H, *J* = 8.2 Hz, 3-H2), 7.07 (d, 2H, *J* = 8.5 Hz, 2-H2), 7.21 (d, 2H, *J* = 8.2 Hz, 3-H3), 7.22 (d, 2H, *J* = 8.5 Hz, 1-H3), 7.73 (d, 2H, *J* = 8.2 Hz, 1-H2); ESI-HRMS found *m/z* 671.3444 $[M-H]^-$ $C_{38}H_{47}N_4O_7$ requires 671.3450.

***N*-(*N*-(*N*-(Isobutyl-4-aminobenzoyl)-*N*-naphth-2-yl-4-aminobenzoyl)-*N*-isobutyl-4-aminobenzoyl)-isoleucine 71**

> 95% pure by NMR, isolated crude yield: 71 mg; δ_H (300 MHz, MeOD) 0.91 (d, 6H, *J* = 6.9 Hz, 1-Hγ), 0.98 (t, 3H, *J* = 7.4 Hz, 4-Hδ), 1.02 (d, 6H, *J* = 6.9 Hz, 3-Hγ), 1.03 (d, 3H, *J* = 6.9 Hz, 4-Hε), 1.35 (m, 1H, 1-Hβ), 1.62 (m, 1H, 3-Hβ), 1.82 (m, 1H, 4-Hγ), 1.95 (m, 1H, 4-Hγ'), 2.04 (m, 1H, 4-Hβ), 2.99 (d, 2H, *J* = 6.9 Hz, 1-Hα), 3.79 (d, 2H, *J* = 7.4 Hz, 3-Hα), 4.58 (d, 1H, *J* = 6.3 Hz, 4-Hα), 5.24 (s, 2H, 2-Hα), 6.58 (d, 2H, *J* = 8.8 Hz, 2-H3), 6.90 (d, 2H, *J* = 8.5 Hz, 3-H2), 7.11 (d, 2H, *J* = 8.8 Hz, 2-H2), 7.15 (d, 2H, *J* = 8.5 Hz, 3-H3), 7.16 (d, 2H, *J* = 8.5 Hz, 1-H3), 7.36 (m, 1H, ArCH), 7.43-7.46 (m, 2H, ArCH), 7.63 (s, 1H, ArCH), 7.71 (d, 2H, *J* = 8.5 Hz, 1-H2), 7.73-7.78 (m, 2H, ArCH), 7.81 (m, 1H, ArCH); ESI-HRMS found *m/z* 739.3831 $[M-H]^-$ $C_{46}H_{51}N_4O_5$ requires 739.3865.

***N*-(*N*-(*N*-(Isobutyl-4-aminobenzoyl)-*N*-isobutyl-4-aminobenzoyl)-*N*-naphth-2-yl-4-aminobenzoyl)-leucine 73**

> 90% pure by NMR, isolated crude yield: 70 mg; δ_H (300 MHz, MeOD) 0.89 (d, 6H, *J* = 6.6 Hz, 2-Hγ), 0.95 (d, 6H, *J* = 6.9 Hz, 4-Hδ), 0.97 (m, 1H, 4-Hγ), 0.99 (d, 6H, *J* = 6.6 Hz, 1-Hγ), 1.72 (m, 2H, 4-Hβ), 1.79 (m, 1H, 2-Hβ), 1.92 (m, 1H, 1-Hβ), 2.96 (d, 2H, *J* = 6.9 Hz, 1-Hα), 3.73 (d, 2H, *J* = 7.4 Hz, 2-Hα), 4.59 (d, 1H, *J* = 6.9 Hz, 4-Hα), 5.35 (s, 2H, 3-Hα), 6.56 (d, 2H, *J* = 8.5 Hz, 2-H3), 7.02 (d, 2H, *J* = 8.5 Hz, 3-H2), 7.03 (d, 2H, *J* = 8.5 Hz, 2-H2), 7.09 (d, 2H, *J* = 8.8 Hz, 3-H3), 7.31 (d, 2H, *J* = 8.8 Hz, 1-H3), 7.43-7.51 (m, 3H, ArCH), 7.62 (d, 2H, *J* = 8.8 Hz, 1-H2), 7.69 (s, 1H, ArCH), 7.76 (m, 1H, ArCH), 7.83 (d, 2H, *J* = 8.8 Hz, ArCH); ESI-MS found *m/z* 741.4 $[M+H]^+$; ESI-HRMS found *m/z* 739.3875 $[M-H]^-$ $C_{46}H_{51}N_4O_5$ requires 739.3865.

***N*-(*N*-(4-chlorobenzyl-4-aminobenzoyl)-*N*-isobutyl-4-aminobenzoyl)-isoleucine 74**

> 90% pure by NMR, isolated crude yield: 64 mg; δ_H (500 MHz, DMSO-d_6) 0.84-0.92 (m, 12H, 2-*Hγ*, 3-*Hδ*, 3-*Hε*), 1.24-1.26 (m, 1H, 3-*Hγ*), 1.48-1.50 (m, 1H, 3-*Hγ*), 1.73-1.80 (m, 1H, 2-*Hβ*), 1.91-1.93 (m, 1H, 3-*Hβ*), 3.71 (d, 2H, *J* = 7.5 Hz, 2-*Hα*), 4.20 (d, 2H, *J* = 6 Hz, 1-*Hα*), 4.28 (t, 1H, *J* = 8 Hz, 3-*Hα*), 6.34 (d, 2H, *J* = 8.5 Hz, 1-*H3*), 6.34 (t, 1H, *J* = 6 Hz, 1-N*H*), 7.00 (d, 2H, *J* = 8.5 Hz, 1-*H2*), 7.16 (d, 2H, *J* = 8.5 Hz, 2-*H3*), 7.30-7.35 (m, 5H, ArC*H*), 7.77 (d, 2H, *J* = 8.5 Hz, 2-*H2*), 8.37 (d, 1H, *J* = 8 Hz, 3-N*H*); ESI-HRMS found *m/z* 572.229311 $[M+Na]^+$ $C_{31}H_{36}ClNaN_3O_4$ requires 572.228655.

***N*-(*N*-(4-chlorobenzyl-4-aminobenzoyl)-*N*-isobutyl-4-aminobenzoyl)-isoleucine 75**

> 90% pure by NMR, isolated crude yield: 64 mg; δ_H (500 MHz, DMSO-d_6) 0.80-0.89 (m, 12H, 1-*Hγ*, 3-*Hδ*, 3-*Hε*), 1.21-1.24 (m, 1H, 3-*Hγ*), 1.42-1.47 (m, 1H, 3-*Hγ*), 1.74-1.77 (m, 1H, 1-*Hβ*), 1.85-1.87 (m, 1H, 3-*Hβ*), 2.78 (t, 2H, *J* = 6.6 Hz, 1-*Hα*), 4.22 (t, 1H, *J* = 8 Hz, 3-*Hα*), 5.28 (s, 2H, 2-*Hα*), 6.13 (t, 1H, *J* =

6 Hz, 1-N*H*), 6.37 (t, 1H, *J* = 8.5 Hz, 1-*H3*), 7.13-7.16 (m, 4H, 2-*H3*, 1-*H2*), 7.45-7.48 (m, 4H, ArC*H*), 7.68 (d, 2H, *J* = 8.6 Hz, 2-*H2*), 7.83-7.85 (m, 3H, ArC*H*), 8.30 (d, 1H, *J* = 8 HZ, 3-N*H*); ESI-HRMS found *m/z* 566.303511 $[M+H]^+$ $C_{35}H_{40}N_3O_4$ requires 566.301333.

_N_-(_N_-(4-chlorobenzyl-4-aminobenzoyl)-_N_-isobutyl-4-aminobenzoyl)-glycine 76

> 90% pure by NMR, isolated crude yield: 2.5 mg; δ_H (500 MHz, DMSO-d_6) 0.93-0.94 (m, 6H, 2-*Hγ*), 1.92-1.96 (m, 1H, 2-*Hβ*), 3.78-3.82 (m, 2H, 2-*Hα*), 4.06-4.17 (m, 2H, 3-*Hα*), 4.25 (s, 2H, 1-*Hα*), 6.34 (d, 2H, *J* = 8.5 Hz, 1-*H3*), 6.34 (d, 1H, *J* = 8.5 Hz, 1-N*H*), 6.79 (d, 1H, *J* = 8 Hz, 3-N*H*), 6.98-7.06 (m, 2H, 1-*H2*), 7.10-7.14 (m, 2H, 2-*H3*), 7.29-7.31 (m, 5H, ArC*H*), 7.68 (d, 2H, *J* = 8.5 Hz, 2-*H2*,); ESI-HRMS found *m/z* 494.185673 $[M+H]^+$ $C_{27}H_{29}ClN_3O_4$ requires 494.184111.

_N_-(_N_-(4-chlorobenzyl-4-aminobenzoyl)-_N_-isobutyl-4-aminobenzoyl)-glycine 77

> 90% pure by NMR, isolated crude yield: 5.8 mg; δ_H (500 MHz, DMSO-d_6); 0.94 (d, 6H, *J* = 6.5 Hz, 1-*Hγ*), 1.82-1.85 (m, 1H, 1-*Hβ*), 2.90 (d, 2H, *J* = 7 Hz, 1-*Hα*), 4.17 (d, 1H, *J* = 5.5 Hz, 3-*Hα*), 5.30 (s, 2H, 2-*Hα*), 6.42 (d, 2H, *J* = 8.5 Hz, 1-*H3*), 6.66 (s, 1H, 3-N*H*), 7.03 (d, 2H, *J* = 8.5 Hz, 2-*H3*), 7.24 (d, 2H, *J* = 8.5 Hz, 1-*H2*), 7.42-7.47 (m, 3H, ArC*H*), 7.58 (d, 2H, *J* = 8.5 Hz, 2-*H2*), 7.70 (s, 1H, ArC*H*), 7.75-7.79 (m, 3H, ArC*H*); ESI-HRMS found *m/z* 510.240688 $[M+H]^+$ $C_{31}H_{32}N_3O_4$ requires 510.238733.

Modified Trimers

1-Biotin-3-azidopropylamine

D-Biotin (1 g, 4.10 mmol) and 3-azidopropan-1-amine (1.11 g, 8.20 mmol) were dissolved in MeOH (50 ml) and DIPEA (2.85 ml, 16.39 mmol). HBTU (3.10 g, 8.20 mmol) was then added and the reaction mixture was stirred overnight at room temperature. The solvent was removed *in vacuo* and the residue taken up in EtOAc and filtered. The isolated precipitate was washed with EtOAc and DCM and recrystallised from MeOH affording the desired product as a white solid (248 mg, 0.81 mmol, 20 %). R_F 0.36 (10% methanol in dichloromethane); δ_H (500 MHz, MeOD) 1.42-1.46 (m, 2H, C*H₂*), 1.57-1.77 (m, 6H, C*H₂*), 2.21 (t, 2H, *J* = 7.25 Hz, C*H₂*CO), 2.71 (d, 1H, *J* = 12.5 Hz, C*H₂*S), 2.92 (dd, 1H, *J* = 12.5, 5 Hz, C*H₂*S), 3.18-3.22 (m, 1H, C*H*S), 3.25 (t, 2H, *J* = 6.5 Hz, CH_2N_3), 3.35 (t, 2H, *J* = 6.5 Hz, CH_2NH), 4.30-4.33 (m, 1H, CH_2C*H*NH), 4.49-4.52 (m, 1H, CHC*H*NH); δ_C (125 MHz, MeOD) 26.68, 29.26, 29.55, 31.74, 37.05, 37.51, 41.00, 48.20, 58.81, 61.38, 63.12, 165.79, 175.89; ν_{max}/cm^{-1} (solid state) = 3300 (NH), 2939 (CH), 2097(N_3), 1646 (CO); ESI-HRMS found *m/z* 349.1415 $[M+Na]^+$ $C_{13}H_{22}N_6NaO_2S$ requires 349.1417.

Biotin-*N*-(*N*-(*N*-(Benzyl-4-aminobenzoyl)-*N*-naphth-1-yl-4-aminobenzoyl)-*N*-isobutyl-4-aminobenzoyl)-glycine Biotin-8

> 90% pure by NMR, isolated crude yield: 48 mg; δ_H (500 MHz, DMSO-d$_6$) 0.82 (d, 6H, *J* = 5.5 Hz, 3-*Hγ*), 1.24-1.31 (m, 2H, 2-3°-*Hη*), 1.50-1.70 (m, 5H, 3-*Hβ*, 2-3°-*Hζ*, 2-3°-*Hϑ*), 1.95-1.97 (m, 2H, 2-3°-*Hγ*), 2.08 (s, 2H, 2-3°-*Hε*), 2.54-2.57 (m, 1H, 2-3°-*Hκ*), 2.73-2.81 (m, 1H, 2-3°-*Hκ*), 3.03-3.09 (m, 4H, 2-3°-*Hδ*, 2-3°-*Hι*), 3.65 (s, 2H, 3-*Hα*), 3.95 (d, 2H, *J* = 5 Hz, 4-*Hα*), 4.10-4.11 (m, 2H, 2-*Hα*), 4.19-4.29 (m, 4H, 1-*Hα*, 2-3°-*Hλ*, 2-3°-*Hα*), 4.38 (t, 2H, *J* = 6 Hz, 2-3°-*Hβ*), 4.86 (s, 1H, 1-N*H*), 6.28-6.38 (m, 4H, 2-*H5*, 2-*H6*, 1-*H3*), 6.88 (d, 2H, *J* = 7.5 Hz, 1-*H2*), 7.02-7.12 (m, 3H, ArC*H*), 7.21-7.33 (m, 6H, ArC*H*), 7.47 (s, 2H, ArC*H*), 7.72-7.75 (m, 3H, ArC*H*), 7.85-7.92 (m, 3H, ArC*H*), 8.13 (s, 1H, 2-3°-*T*), 8.85-8.86 (m, 1H, 4-N*H*); ESI-HRMS found *m/z* 1099.486619 $[M+H]^+$ $C_{61}H_{67}N_{10}O_8S$ requires 1099.485857.

Biotin-*N*-(*N*-(*N*-(4-fluorobenzyl-4-aminobenzoyl)-*N*-naphth-2-yl-4-aminobenzoyl)-*N*-isobutyl-4-aminobenzoyl)-glycine Biotin-31

> 90% pure by NMR, isolated crude yield: 33.5 mg; δ_H (500 MHz, DMSO-d$_6$) 0.83 (d, 6H, *J* = 6.5 Hz, 3-*Hγ*), 1.29-1.32 (m, 2H, 2-3°-*Hη*), 1.51-1.71 (m, 5H, 3-*Hβ*, 2-3°-*Hζ*, 2-3°-*Hϑ*), 1.93 (t, 2H, *J* = 7 Hz, 2-3°-*Hγ*), 2.08 (t, 2H, *J* = 7 Hz, 2-3°-*Hε*), 2.56 (d, 1H, *J* = 12.5, 2-3°-*Hκ'*), 2.78 (dd, 1H, *J* = 12.5, 5 Hz, 2-3°-*Hκ*), 3.03-3.07 (m, 3H, 2-3°-*Hδ*, 2-3°-*Hι*), 3.68 (d, 2H, *J* = 6 Hz, 3-*Hα*), 3.93 (d, 2H, *J* = 5.5 Hz, 4-*Hα*), 4.10-4.11 (m, 2H, 2-*Hα*), 4.23 (m, 3H, 2-3°-*Hλ'*, 1-*Hα*), 4.28 (t, 1H, *J* = 7 Hz, 2-3°-*Hλ*), 4.34 (t, 2H, *J* = 7 Hz, 2-3°-*Hβ*), 4.87 (s, 1H, 1-N*H*), 6.28 (d, 2H, *J* = 8.5 Hz, 1-*H3*), 6.56 (d, 1H, *J* = 8 Hz, 2-*H6*), 6.65 (d, 1H, *J* = 8 Hz, 2-*H5*), 6.91 (d, 2H, *J* = 8.5 Hz, 1-*H2*), 7.10 (t, 2H, *J* = 8 Hz, 3-*H2*), 7.17 (d, 2H, *J* = 8 Hz, ArC*H*), 7.34 (dd, 2H, *J* = 8, 6 Hz, 3-*H3*), 7.42-7.44 (m, 2H, ArC*H*), 7.62 (s, 1H, ArC*H*), 7.68-7.81 (m, 5H, ArC*H*, N*H* biotin), 7.89-7.91 (m, 2H, ArC*H*), 7.96 (s, 1H, 2-3°-*T*), 8.85 (s, 1H, 4-N*H*); ESI-HRMS found *m/z* 559.243469 $[2M+H]^{2+}$ $C_{61}H_{67}FN_{10}O_8S$ requires 559.241856.

Biotin-*N*-(*N*-(*N*-(Cyclohexylmethyl-4-aminobenzoyl)-*N*-naphth-2-yl-4-aminobenzoyl)-*N*-phenethyl-4-aminobenzoyl)-glycine Biotin-48

> 90% pure by NMR, isolated crude yield: 43.9 mg; δ_H (500 MHz, DMSO-d$_6$) 0.89-0.92 (m, 2H, 1-*Hε*), 1.14-1.31 (m, 6H, 1-*Hδ*, 1-*Hδ'*, 2-3°-*Hη*), 1.51-1.75 (m, 9H, 1-*Hγ*, 1-*Hγ'*, 1-*Hβ*, 2-3°-*Hζ*, 2-3°-*Hϑ*), 2.04 (t, 2H, *J* = 6.5 Hz, 2-3°-*Hγ*), 2.08 (t, 2H, *J* = 7 Hz, 2-3°-*Hε*), 2.55-2.89 (m, 4H, 3-*Hβ*, 1-*Hα*, 2-3°-*Hκ*), 3.03-3.07 (m, 3H, 2-3°-*Hδ*, 2-3°-*Hι*), 3.95-4.36 (m, 12H, 2-3°-*Hα*, 3-*Hα*, 2-*Hα*, 4-*Hα*, 2-3°-*Hβ*, 2-3°-*Hλ*), 4.90 (s, 1H, 1-N*H*), 6.23 (d, 2H, *J* = 8 Hz, 1-*H3*), 6.54 (d, 1H, *J* = 7 Hz, 2-*H5*), 6.67 (d, 1H, *J* = 7 Hz, 2-*H6*), 6.92 (d, 2H, *J* = 8 Hz, 1-*H3*), 7.10-7.26 (m, 8H, 3-*H3*, 3-*H2*, 3-*H*Ar, C*H* naph), 7.44 (s, 2H N*H* biotin), 7.63-7.81 (m, 6H, ArC*H*), 7.89-7.93 (m, 1H, 2-3°-*T*), 8.87 (s, 2H, 4-N*H*); ESI-HRMS found *m/z* 1153.533652 $[M+H]^+$ $C_{65}H_{73}N_{10}O_8S$ requires 1153.532807.

Biotin-*N*-(*N*-(*N*-(Isobutyl-4-aminobenzoyl)-*N*-naphth-2-yl-4-aminobenzoyl)-*N*-2-methylbutyl-4-aminobenzoyl)-isoleucine Biotin-64

> 90% pure by NMR, isolated crude yield: 26.3 mg; δ_H (500 MHz, DMSO-d_6) 0.76-0.92 (m, 18H, 1-*Hγ*, 3-*Hδ*, 3-*Hε*, 4-*Hδ*, 4-*Hε*), 1.20-1.34 (m, 6H, 2-3°-*Hη*, 3-*Hγ*, 4-*Hγ*), 1.47-1.64 (m, 5H, 3-*Hβ*, 2-3°-*Hζ*, 2-3°-*Hϑ*), 1.75-1.78 (m, 2H, 1-*Hβ*), 1.92-1.95 (m, 3H, 2-3°-*Hγ*, 4-*Hβ*), 2.08 (t, 2H, *J* = 7 Hz, 2-3°-*Hε*), 2.56 (d, 1H, *J* = 12.5 Hz, 2-3°-*Hκ'*), 2.77-2.81 (m, 3H, 1-*Hα*, 2-3°-*Hκ*), 3.06-3.09 (m, 3H, 2-3°-*Hδ*, 2-3°-*Hι*), 3.72 (d, 2H, 4-*Hα*), 4.09-4.12 (m, 1H, 2-3°-*Hλ'*), 4.28 (t, 1H, *J* = 7 Hz, 2-3°-*Hλ*), 4.33-4.38 (m, 8H, 2-3°-*Hα*, 2-3°-*Hβ*, 2-*Hα*, 3-*Hα*), 4.91 (s, 2H, 1-N*H*), 6.25 (d, 2H, *J* = 8 Hz, 1-*H3*), 6.57 (d, 1H, *J* = 7.5 Hz, 2-*H6*), 6.65 (d, 1H, *J* = 7.5 Hz, 2-*H5*), 6.92 (d, 2H, *J* = 8 Hz, 1-*H2*), 7.11-7.19 (m, 4H, ArC*H*), 7.42-7.45 (m, 3H, ArC*H*), 7.64-7.80 (m, 8H, ArC*H*, N*H* biotin, N*H*), 7.89-7.91 (m, 2H, 2-3°-*T*, ArC*H*), 8.46 (d, 1H, *J* = 8 Hz, 4-N*H*,); ESI-HRMS found *m/z* 1135.580014 $[M+H]^+$ $C_{63}H_{79}N_{10}O_8S$ requires 1135.579757.

Biotin-*N*-(*N*-(*N*-(Isobutyl-4-aminobenzoyl)-*N*-4-fluorobenzyl-4-aminobenzoyl)-*N*-isobutyl-4-aminobenzoyl)-isoleucine Biotin-67

> 90% pure by NMR, isolated crude yield: 25.4 mg; δ_H (500 MHz, DMSO-d_6) 0.84-0.92 (m, 18H, 1-*Hγ*, 3-*Hγ*, 4-*Hδ*, 4-*Hε*), 1.24 (s, 4H, 4-*Hγ*, 2-3°-*Hη*), 1.49-1.77 (m, 7H, 4-*Hβ*, 3-*Hβ*, 1-*Hβ*, 2-3°-*Hζ*, 2-3°-*Hϑ*), 1.95-1.96 (m, 2H, 2-3°-*Hγ*), 2.08 (s, 2H, 2-3°-*Hε*), 2.56 (d, 1H, *J* = 12, 2-3°-*Hκ'*), 2.78 (d, 2H, *J* = 5.5 Hz, 1-*Hα*, 2-3°-*Hκ*), 3.08 (s, 3H, 2-3°-*Hδ*, 2-3°-*Hι*), 3.69 (s, 2H, 2-*Hα*), 4.12 (s, 2H, 3-*Hα*), 4.28-4.39 (m, 4H, 4-*Hα*, 2-3°-*Hβ*, 2-3°-*Hλ*), 4.93 (s, 3H, 1-N*H*, 2-3°-*Hα*), 6.26 (d, 2H, *J* = 7.5 Hz, 1-*H3*), 6.59 (s, 2H, 2-*H5*, 2-*H6*), 6.89 (d, 2H, *J* = 7.5 Hz, 1-*H2*), 7.05 (d, 3H, *J* = 7.5 Hz, ArC*H*), 7.12 (s, 1H, 2-*H2*), 7.20 (d, 4H, *J* = 7 Hz, ArC*H*), 7.78-7.79 (m, 2H, ArC*H*), 7.80-8.04 (m, 2H, 2-3°-*T*, 2-3°-N*H*), 8.44-8.46 (m, 1H, 4-N*H*); ESI-HRMS found *m/z* 1105.509484 $[M+H]^+$ $C_{58}H_{74}ClN_{10}O_8S$ requires 1105.510332.

Fluorescein-*N*-(*N*-(*N*-(4-fluorobenzyl-4-aminobenzoyl)-*N*-naphth-2-yl-4-aminobenzoyl)-*N*-isobutyl-4-aminobenzoyl)-glycine FITC-31

> 90% pure by NMR, isolated crude yield: 59 mg; δ_H (500 MHz, DMSO-d_6) 0.82-0.85 (m, 6H, 3-*Hγ*), 1.63-1.70 (m, 2H, 3-*Hβ*), 2.16-2.17 (m, 2H, 2-3°-*Hγ*), 3.57-3.68 (m, 4H, 3-*Hα*, 2-3°-*Hδ*), 3.93 (d, 2H, *J* = 5.5 Hz, 4-*Hα*), 4.23 (s, 2H, 1- *Hα*), 4.44 (s, 2H, 2-3°-*Hβ*), 4.88 (s, 1H, 1-N*H*), 6.29 (d, 2H, *J* = 8 Hz, 1-*H3*), 6.55-6.68 (m, 8H, 2-*H6*, 2-*H5*, 2-3°-Ar*H*), 6.92 (d, 2H, *J* = 8 Hz, 1-*H2*), 7.08-7.10 (m, 3H, 2-3°-Ar*H*, 3-*H2*), 7.18 (d, 2H, *J* = 7.5 Hz, 3-*H3*), 7.32-7.34 (m, 2H, ArC*H*), 7.42 (d, 1H, *J* = 8.5 Hz, ArC*H*), 7.63-7.79 (m, 6H, ArC*H*), 7.93-7.96 (m, 2H, 2-3°-*T*, 2-3°-Ar*H*), 8.84-8.86 (s, 1H, 4-N*H*), 10.07-10.11 (br s, 2H, 2-3°-*NH*).

Fluorescein-*N*-(*N*-(*N*-(Cyclohexylmethyl-4-aminobenzoyl)-*N*-naphth-2-yl-4-aminobenzoyl)-*N*-phenethyl-4-aminobenzoyl)-glycine FITC-48

> 90% pure by NMR, isolated crude yield: 44.7 mg; δ_H (500 MHz, DMSO-d$_6$) 0.83-0.86 (m, 2H, 1-*Hε*), 1.12-1.22 (m, 4H, 1-*Hδ*, 1-*Hδ'*), 1.45-1.75 (m, 5H, 1-*Hγ*, 1-*Hγ'*, 1-*Hβ*), 2.13-2.16 (m, 2H, 2-3°-*Hγ*), 2.81 (s, 4H, 3-*Hβ*, 1-*Hα*), 3.52-3.57 (m, 2H, 2-3°-*Hδ*), 3.94-3.98 (m, 8H, 2-3°-*Hα*, 2-*Hα*, 3-*Hα*, 4-*Hα*), 4.40-4.44(m, 2H, 2-3°-*Hβ*), 4.88 (s, 1H, 1-N*H*), 6.23 (d, 2H, *J* = 8 Hz, 1-*H3*), 6.52-6.68 (m, 8H, 2-*H6*, 2-*H5*, 2-3°-Ar*H*), 6.91 (d, 2H, *J* = 8 Hz, 1-*H2*), 6.97-7.26 (m, 12H, 3-*H3*, 3-*H2*, 3-*H*Ar, C*H* naph, 2-3°-Ar*H*), 7.41-7.45 (m, 2H, ArC*H*), 7.63-7.80 (m, 7H, ArC*H*), 7.96 (s, 1H, 2-3°-Ar*H*), 8.23 (s, 1H, 2-3°-*T*, 2-3°-Ar*H*), 8.85 (d, 1H, *J* = 5.5 Hz, 4-N*H*), 10.02-10.05 (br s, 2H, 2-3°-*NH*).

Fluorescein-*N*-(*N*-(*N*-(Isobutyl-4-aminobenzoyl)-*N*-naphth-2-yl-4-aminobenzoyl)-*N*-2-methylbutyl-4-aminobenzoyl)-isoleucine FITC-64

> 90% pure by NMR, isolated crude yield: 51.6 mg; δ_H (500 MHz, DMSO-d$_6$) 0.76-0.91 (m, 18H, 1-*Hγ*, 3-*Hδ*, 3-*Hε*, 4-*Hδ*, 4-*Hε*), 1.23-1.37 (m, 4H, 3-*Hγ*, 4-*Hγ*), 1.43-1.59 (m, 1H, 1-*Hβ*), 1.75-1.95 (m, 3H, 2-3°-*Hγ*, 3-*Hβ*), 2.14-2.16 (m, 1H, 4-*Hβ*), 2.78 (d, 2H, *J* = 6.5 Hz, 3-*Hα*), 3.56-3.57 (m, 2H, 2-3°-*Hδ*), 3.71 (s, 4H, 1-*Hα*, 2-*Hα*), 4.36-4.45 (m, 4H, 2-3°-*Hβ*, 4-*Hα*), 4.90 (s, 1H, 1-N*H*), 6.25 (d, 2H, *J* = 8 Hz, 1-*H3*), 6.54-6.67 (m, 8H, 2-*H6*, 2-*H5*, 2-3°-ArC*H*), 6.92 (d, 2H, *J* = 8 Hz, 1-*H2*), 7.10-7.19 (m, 5H, ArC*H*), 7.41-7.42 (m, 2H, ArC*H*), 7.67-7.93 (m, 7H, ArC*H*), 8.23 (s, 1H, 2-3°-*T*), 8.46 (d, 1H, *J* = 8 Hz, 4-N*H*), 10.01-10.04 (br s, 2H, 2-3°-N*H*).

Fluorescein-*N*-(*N*-(*N*-(Isobutyl-4-aminobenzoyl)-*N*-4-fluorobenzyl-4-aminobenzoyl)-*N*-isobutyl-4-aminobenzoyl)-isoleucine FITC-67

> 90% pure by NMR, isolated crude yield: 51 mg; δ_H (500 MHz, DMSO-d$_6$) 0.83-0.91 (m, 18H, 1-*Hγ*, 3-*Hγ*, 4-*Hδ*, 4-*Hε*), 1.23-1.26 (m, 2H, 4-*Hγ*), 1.68-1.76 (m, 2H, 1-*Hβ*, 4-*Hβ*), 1.92 (s, 1H, 4-*Hβ*), 2.16-2.19 (m, 2H, 2-3°-*Hγ*), 2.77 (d, 2H, *J* = 6.5 Hz, 1-*Hα*), 3.66-3.68 (m, 6H, 2-*Hα*, 3-*Hα*, 2-3°-*Hδ*), 4.33 (t, 2H, *J* = 8 Hz, 4-*Hα*), 4.43-4.50 (m, 2H, 2-3°-*Hβ*), 4.92 (s, 3H, 1-N*H*, 2-3°-*Hα*), 6.23 (d, 2H, *J* = 8.5 Hz, 1-*H3*), 6.52-6.66 (m, 8H, 2-*H5*, 2-*H6*, ArC*H*), 6.88 (d, 2H, *J* = 8.5 Hz, 1-*H2*), 7.04-7.19 (m, 9H, ArC*H*), 7.74-7.78 (m, 3H, ArC*H*), 8.22 (s, 1H, 2-3°-*T*), 8.42-8.43 (m, 1H, 4-N*H*).

NMR Spectra

^{1}H NMR of Monomer b in DMSO-d$_6$

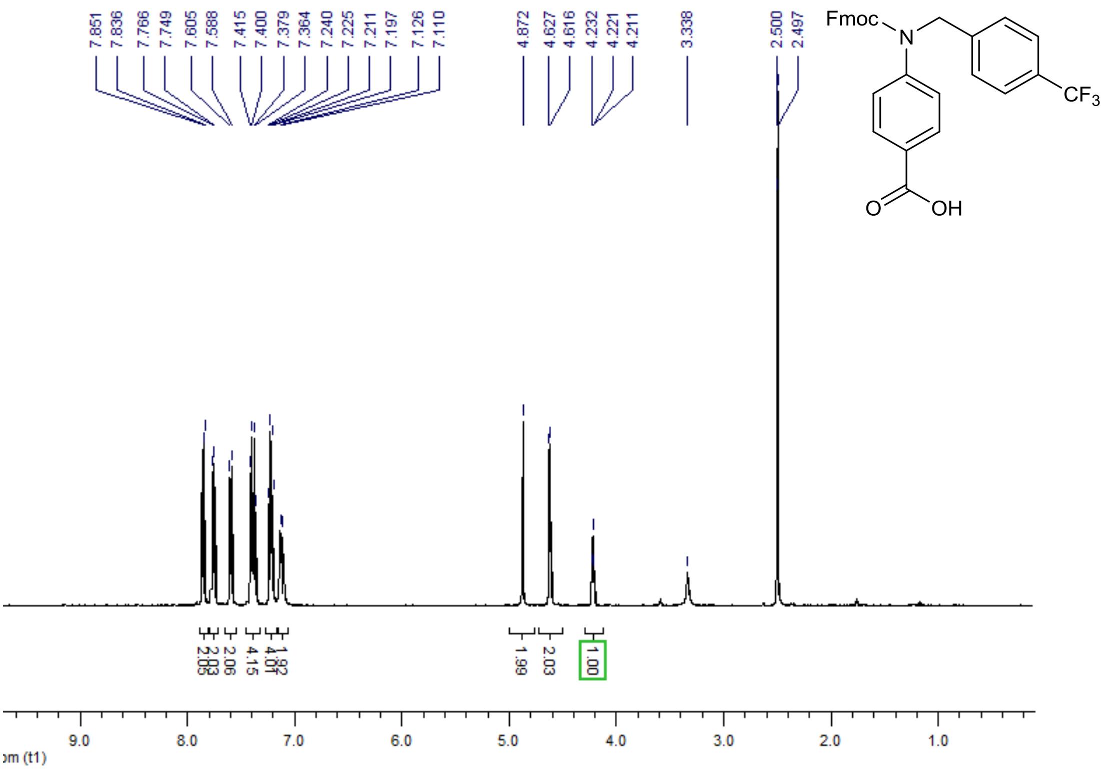

^{1}H NMR of Monomer d in DMSO-d$_6$

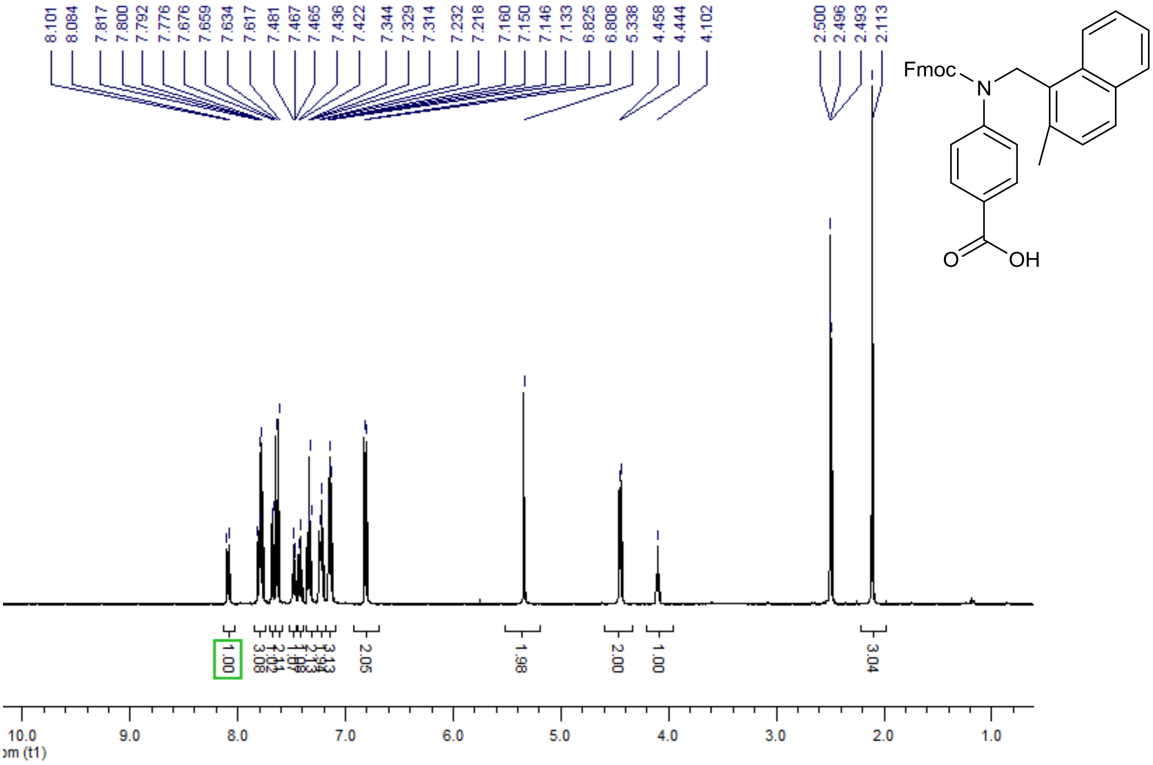

^{1}H NMR of Monomer i in DMSO-d_6

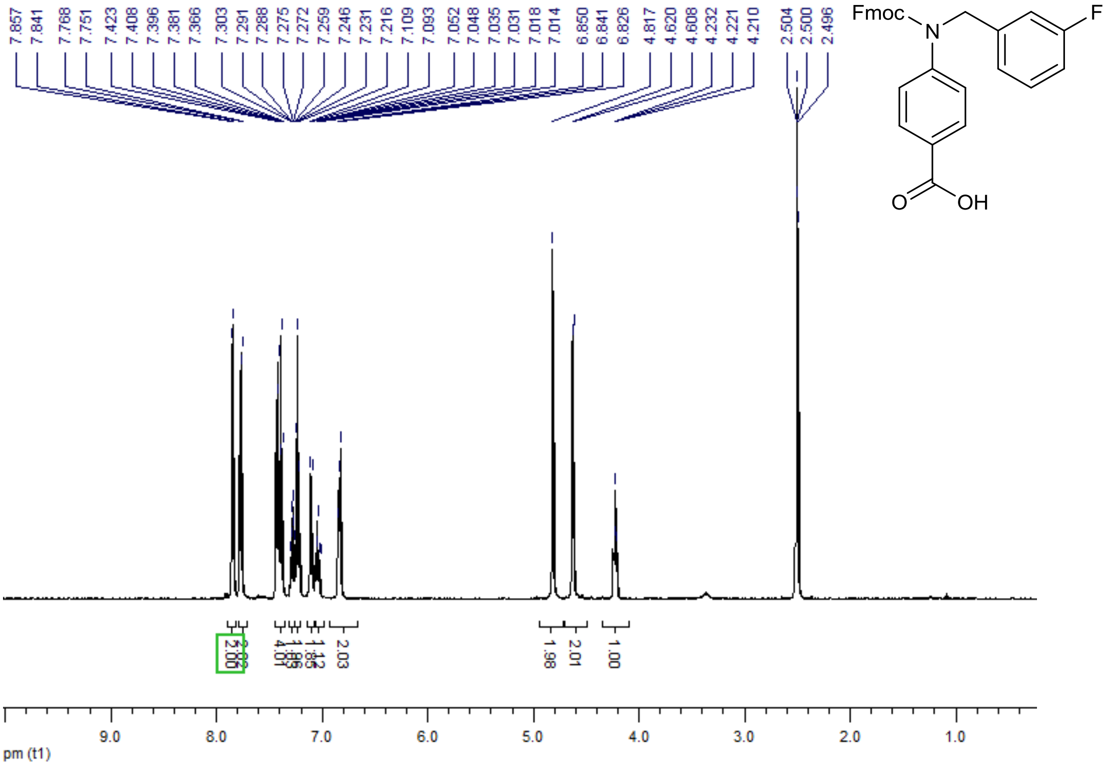

^{1}H NMR of Monomer p in $CDCl_3$

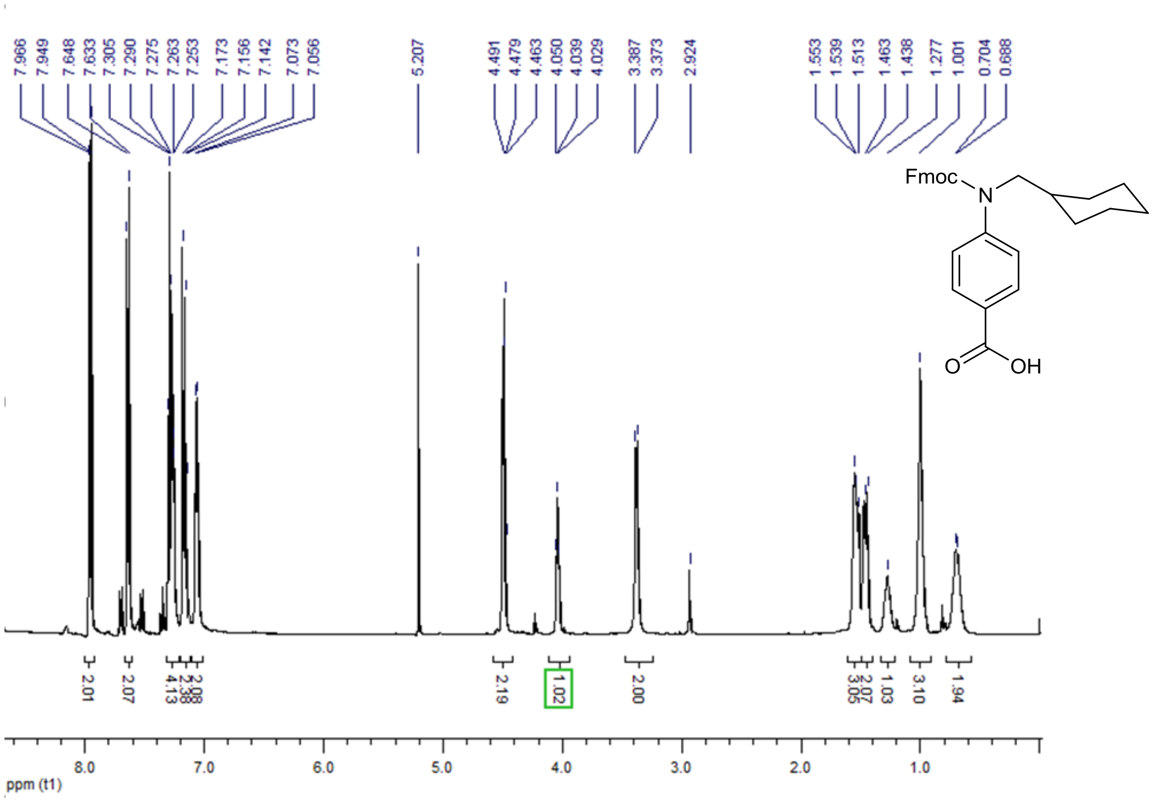

^{1}H NMR of Monomer v in CDCl$_3$

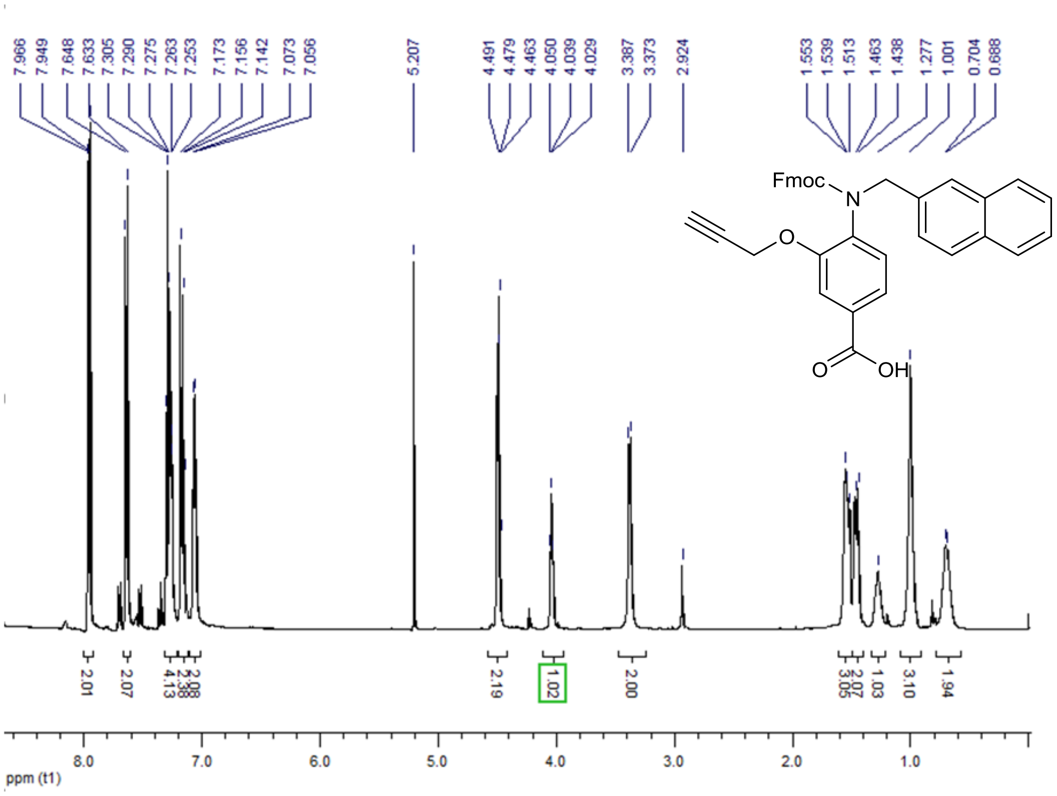

^{1}H NMR of Monomer w in CDCl$_3$

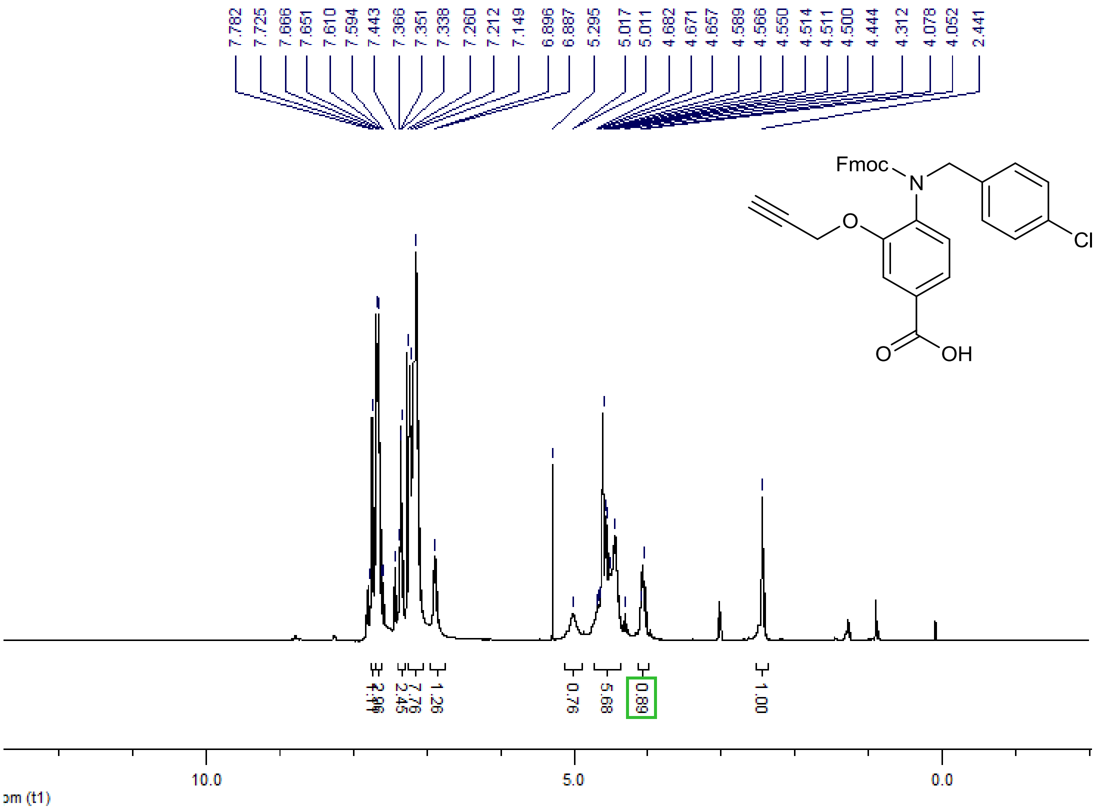

^{1}H NMR of Compound 1 in MeOD

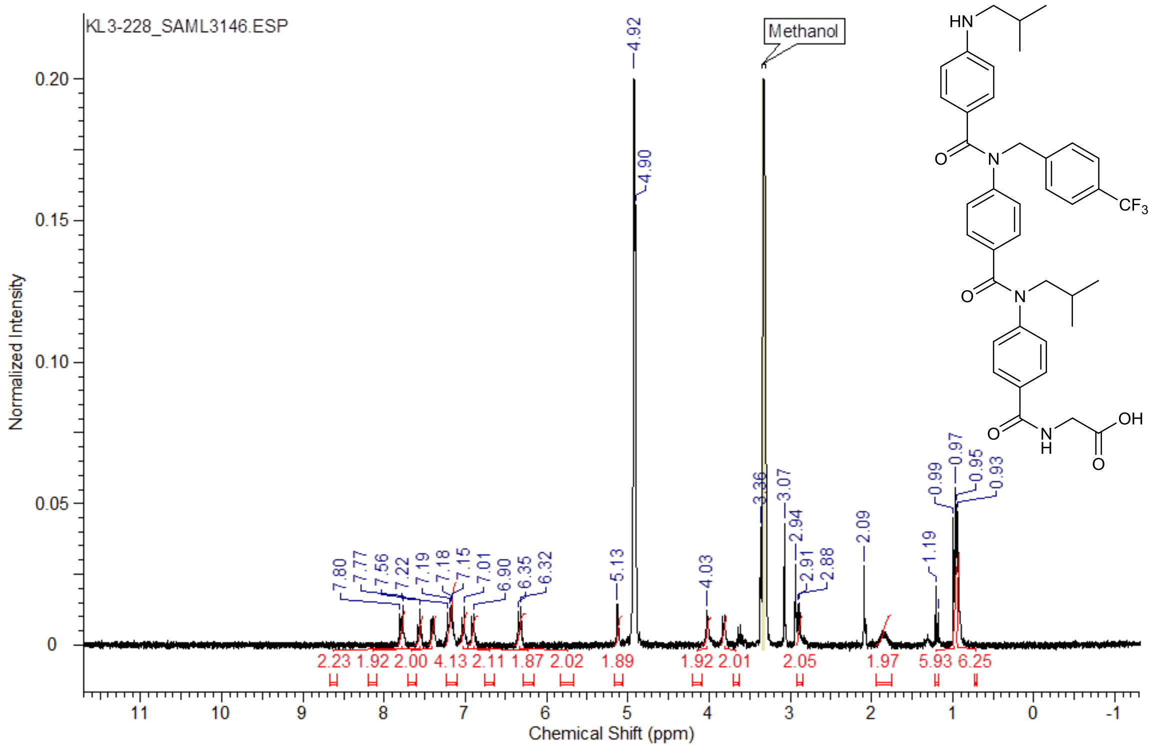

^{1}H NMR of Compound 2 in MeOD

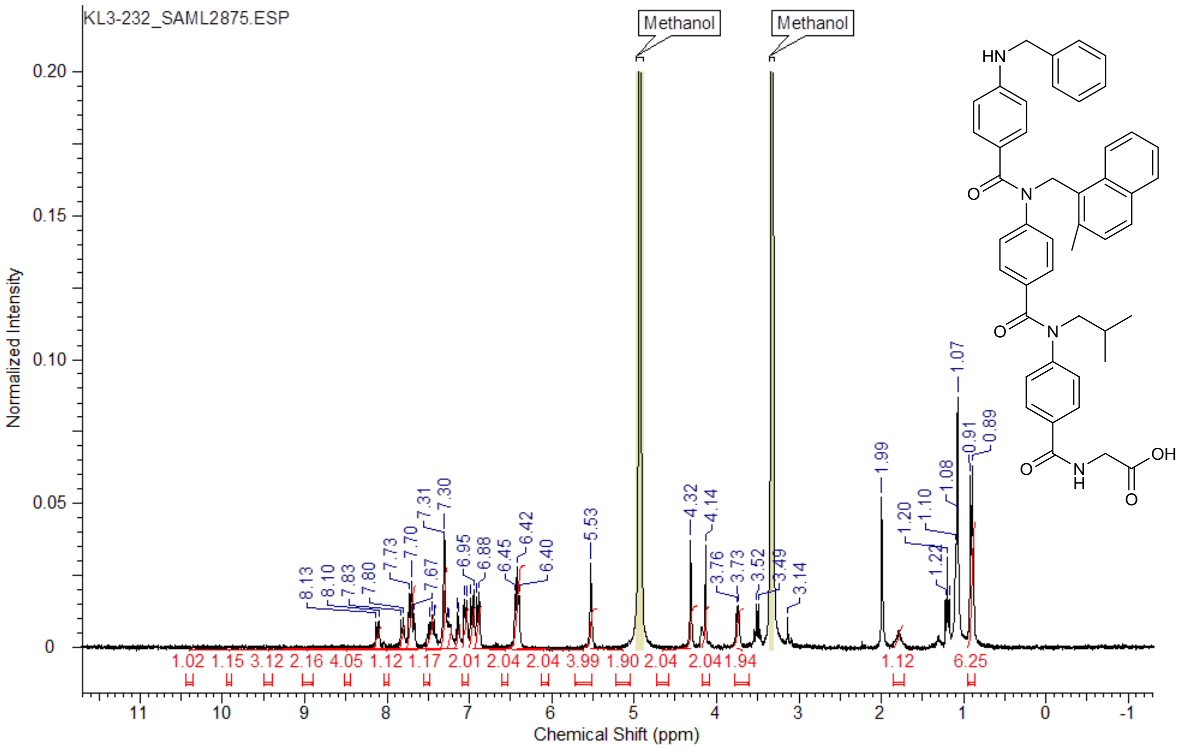

^{1}H NMR of Compound 3 in MeOD

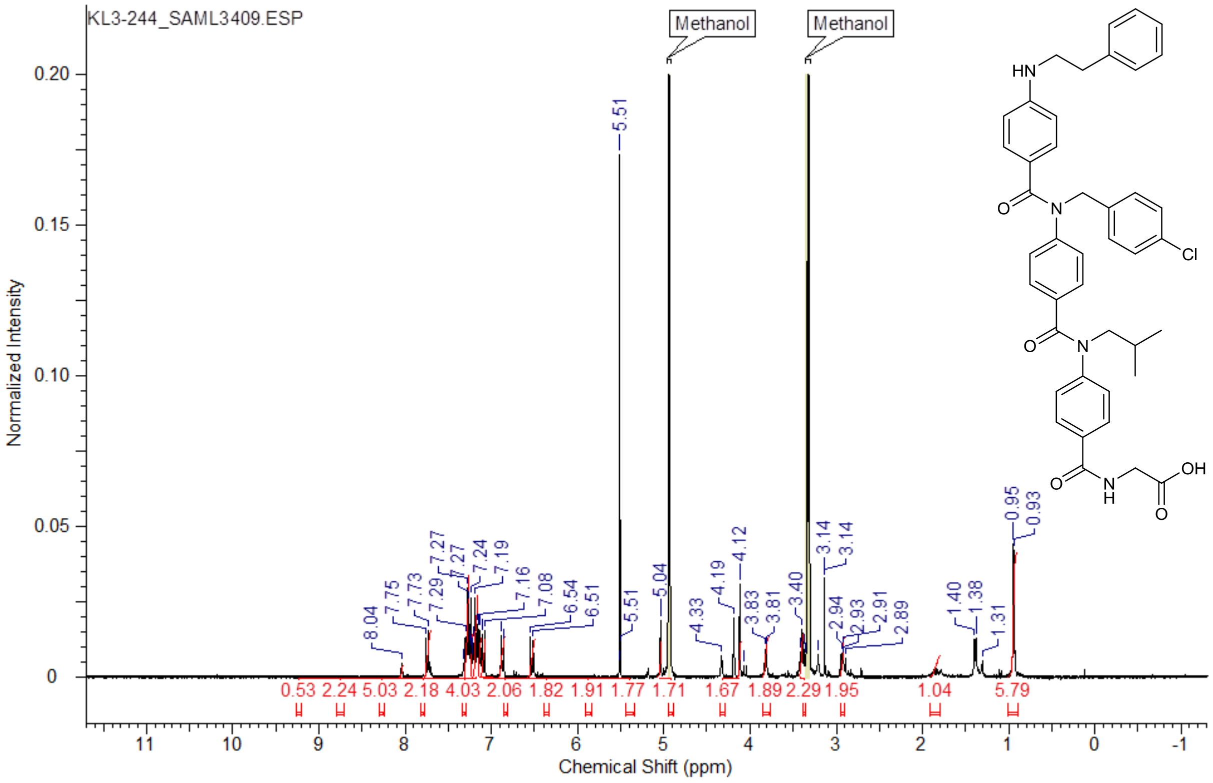

^{1}H NMR of Compound 4 in MeOD

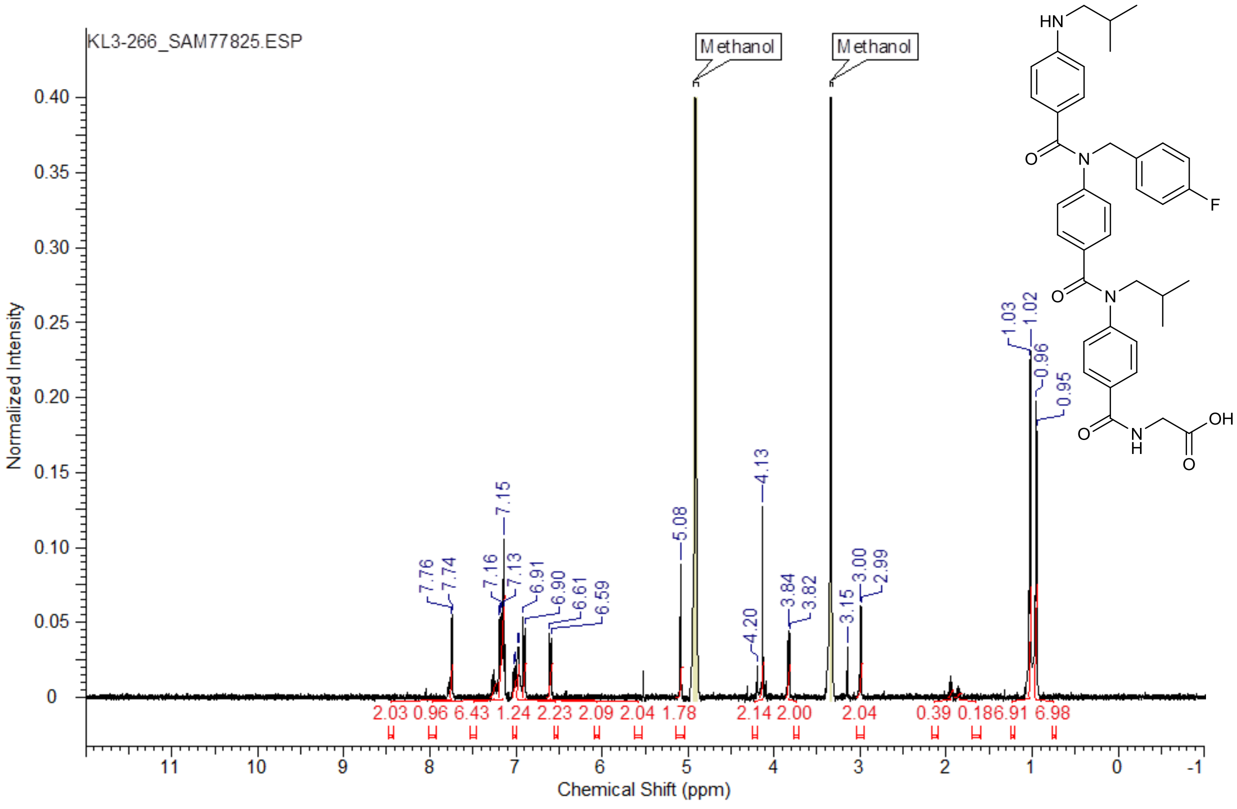

^{1}H NMR of Compound 5 in MeOD

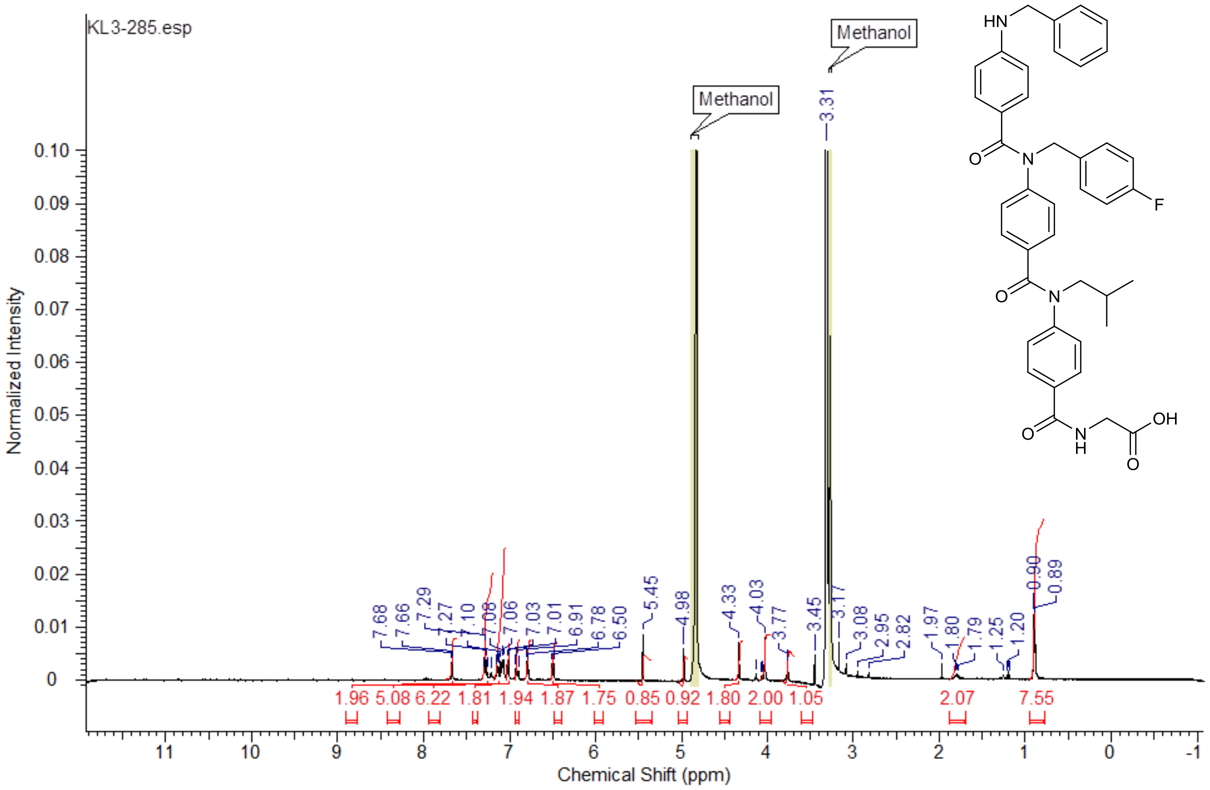

^{1}H NMR of Compound 7 in MeOD

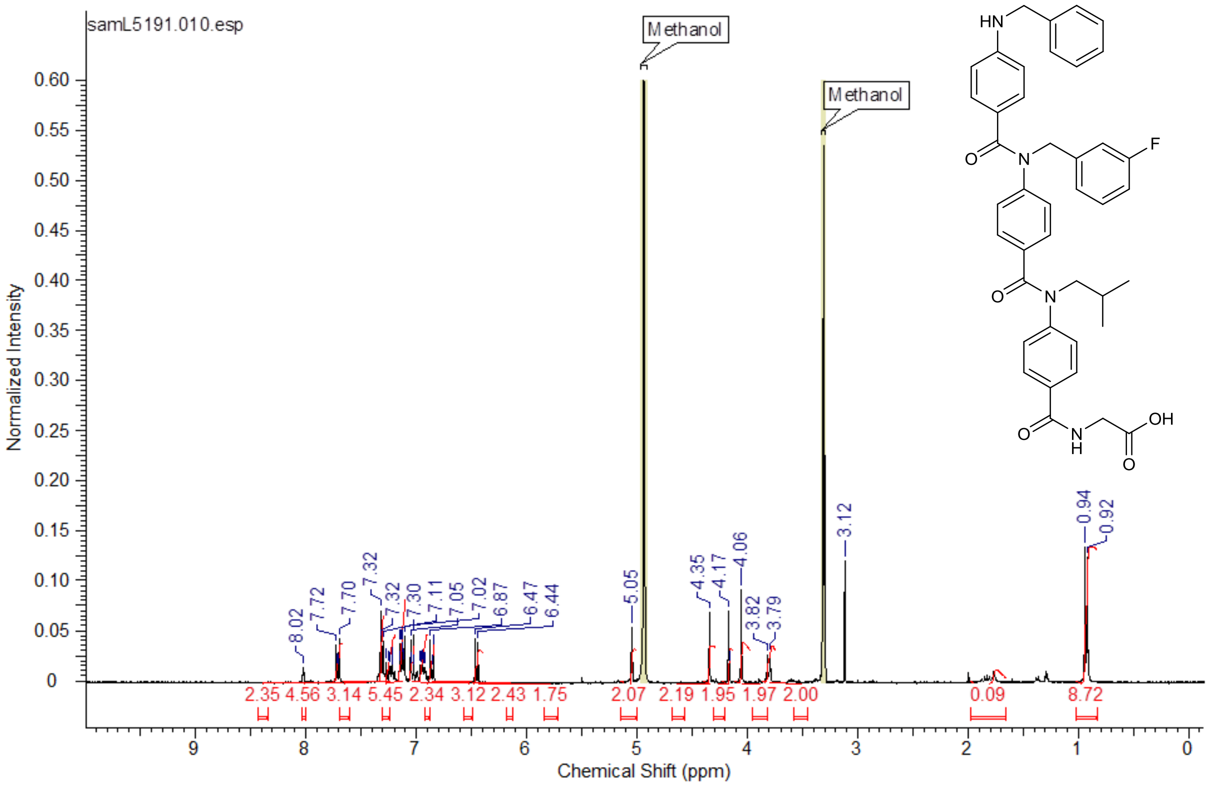

^{1}H NMR of Compound 8 in MeOD

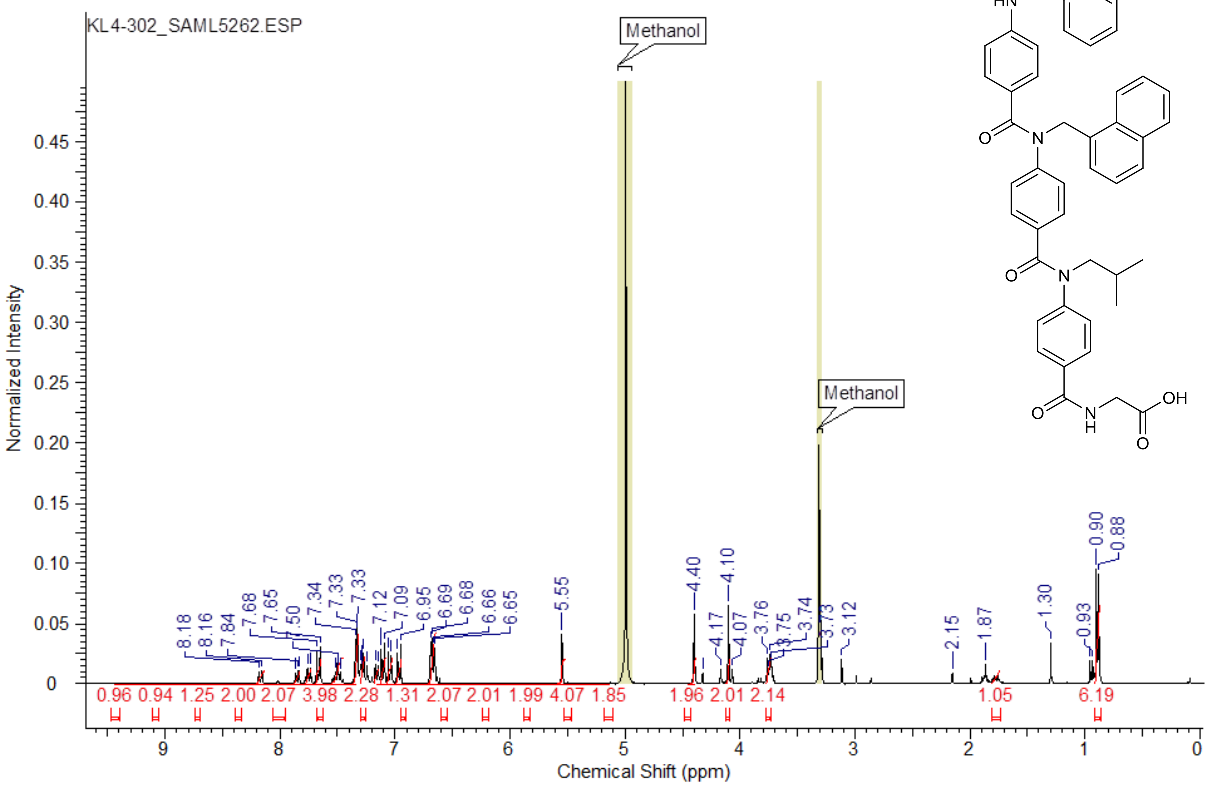

^{1}H NMR of Compound 9 in MeOD

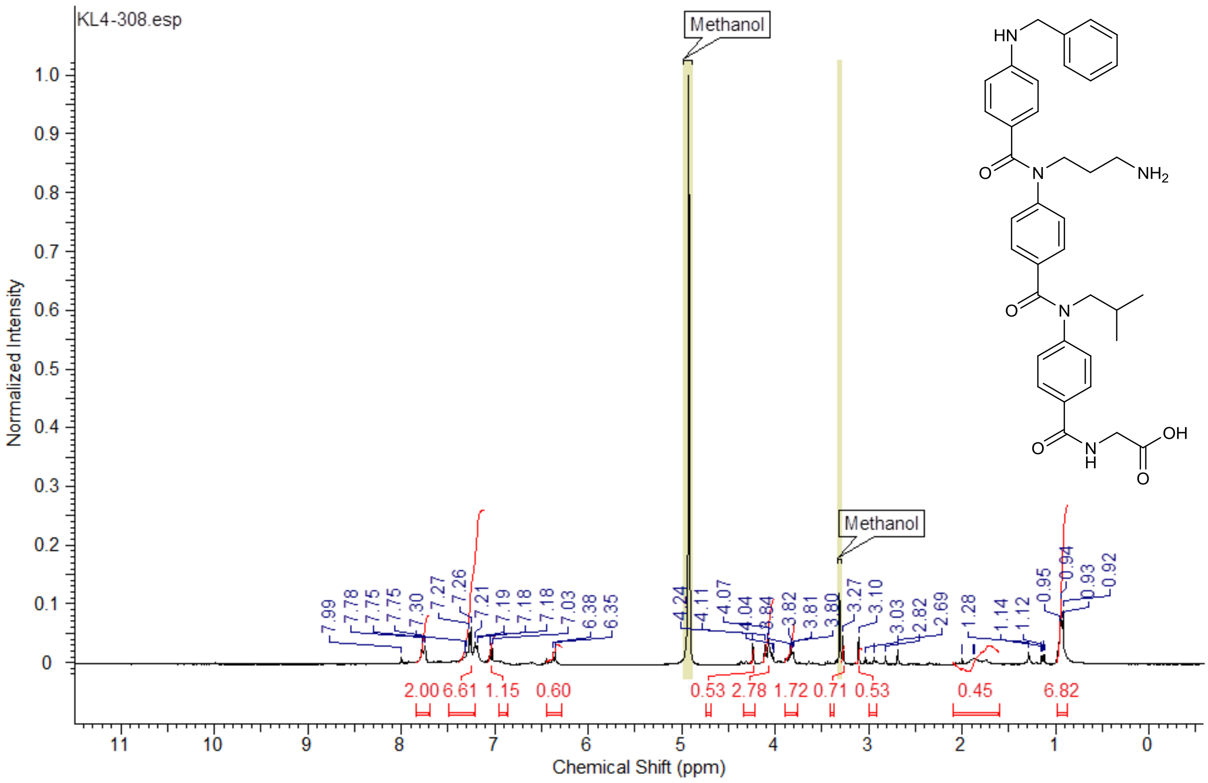

^{1}H NMR of Compound 10 in MeOD

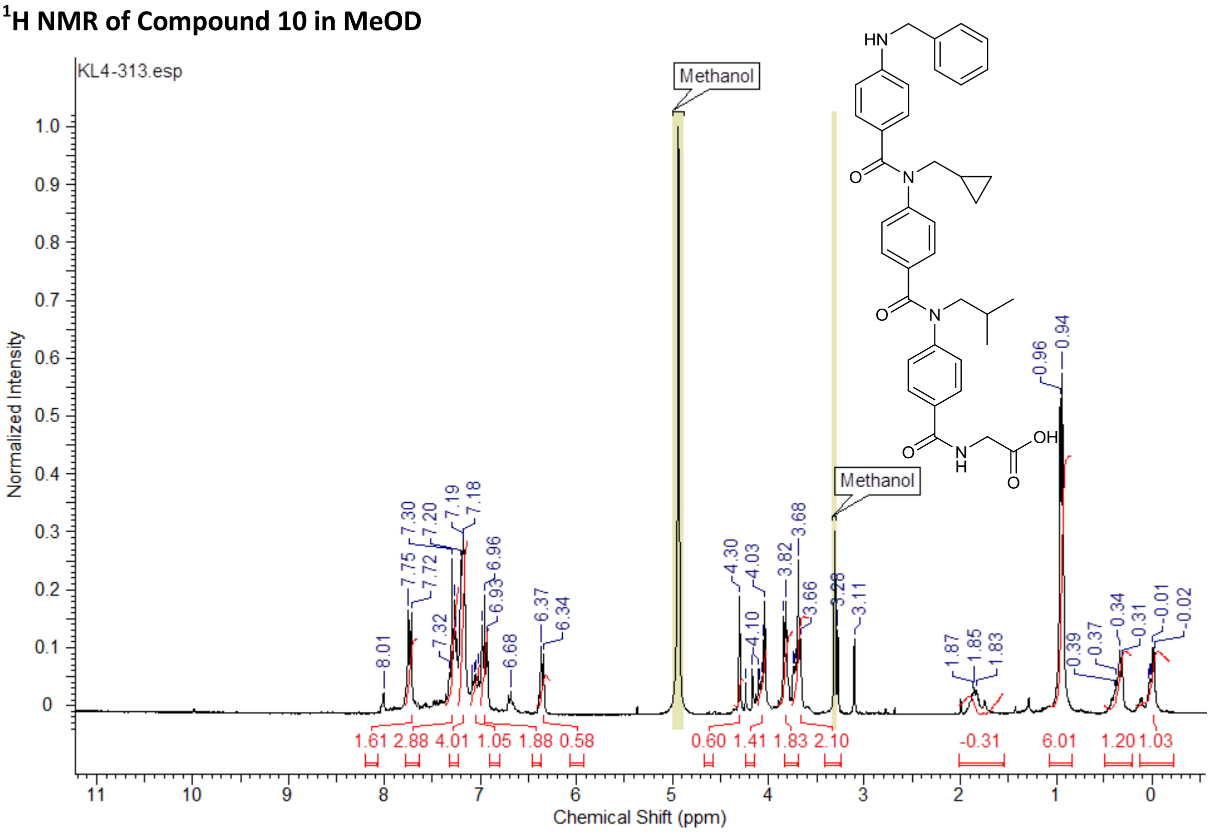

^{1}H NMR of Compound 11 in MeOD

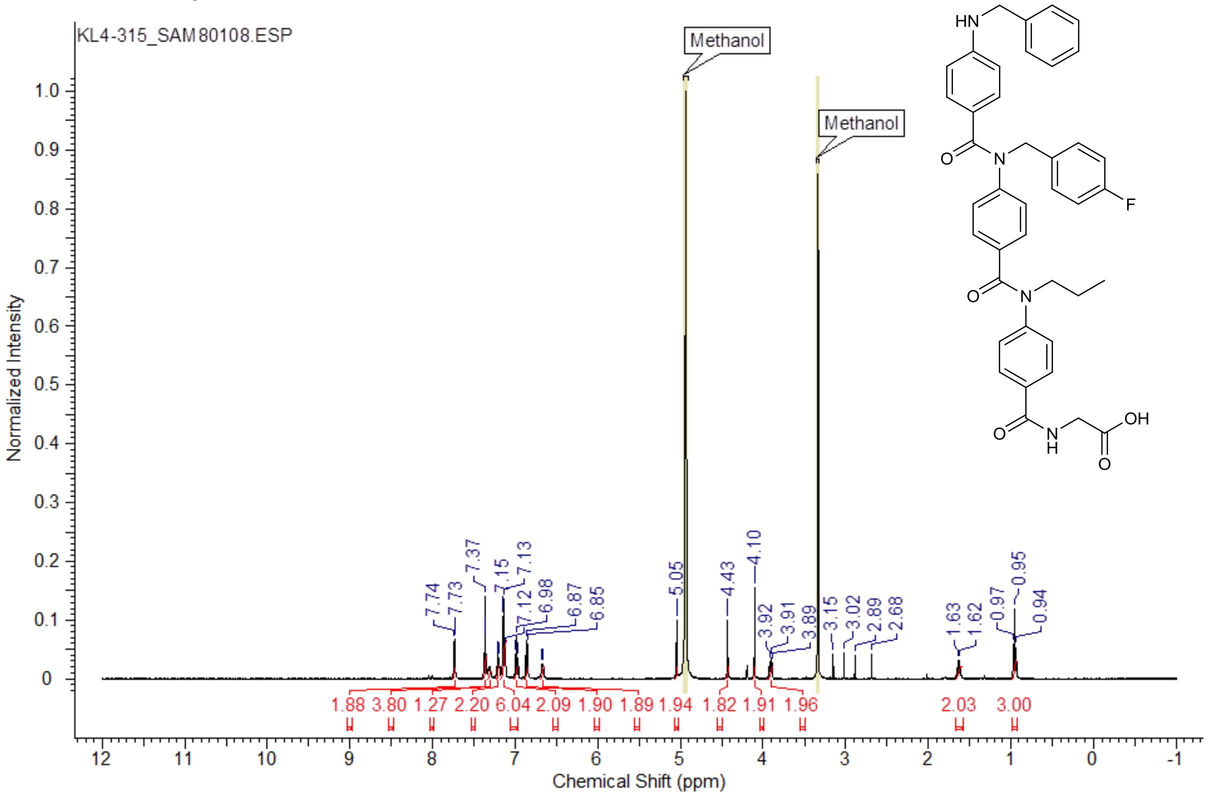

^{1}H NMR of Compound 12 in MeOD

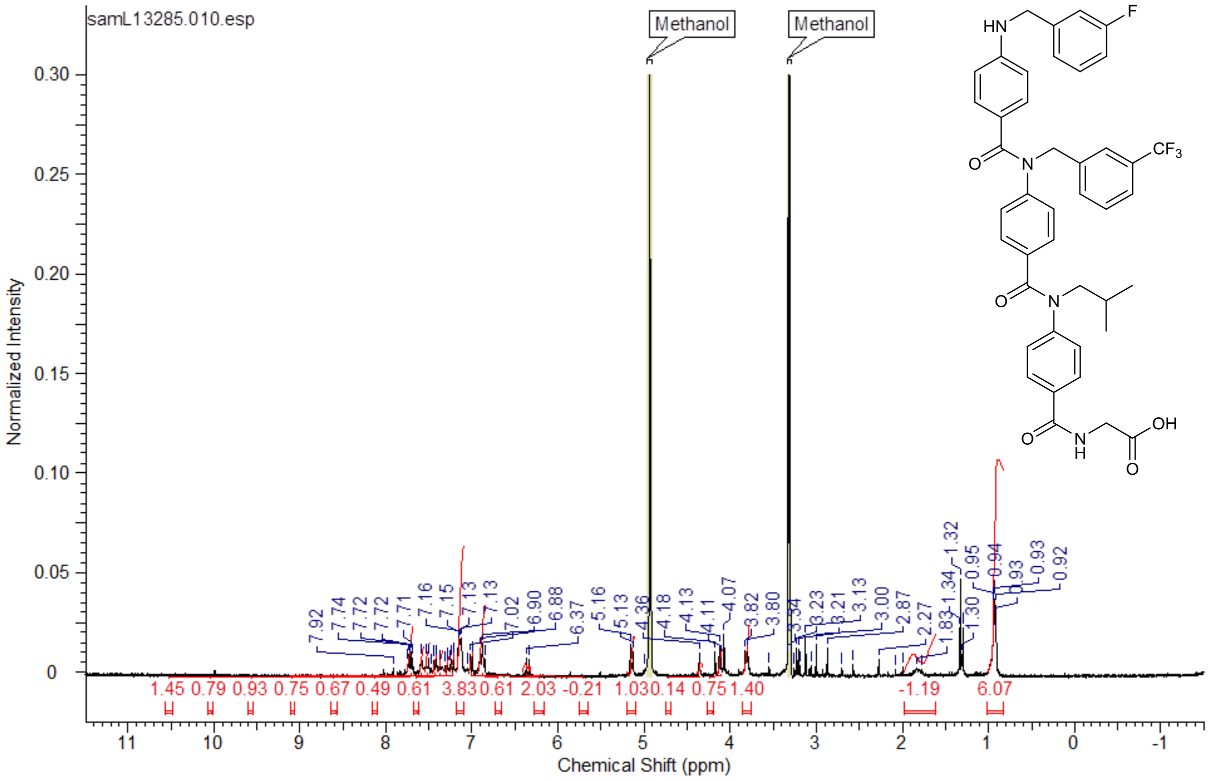

^{1}H NMR of Compound 13 in MeOD

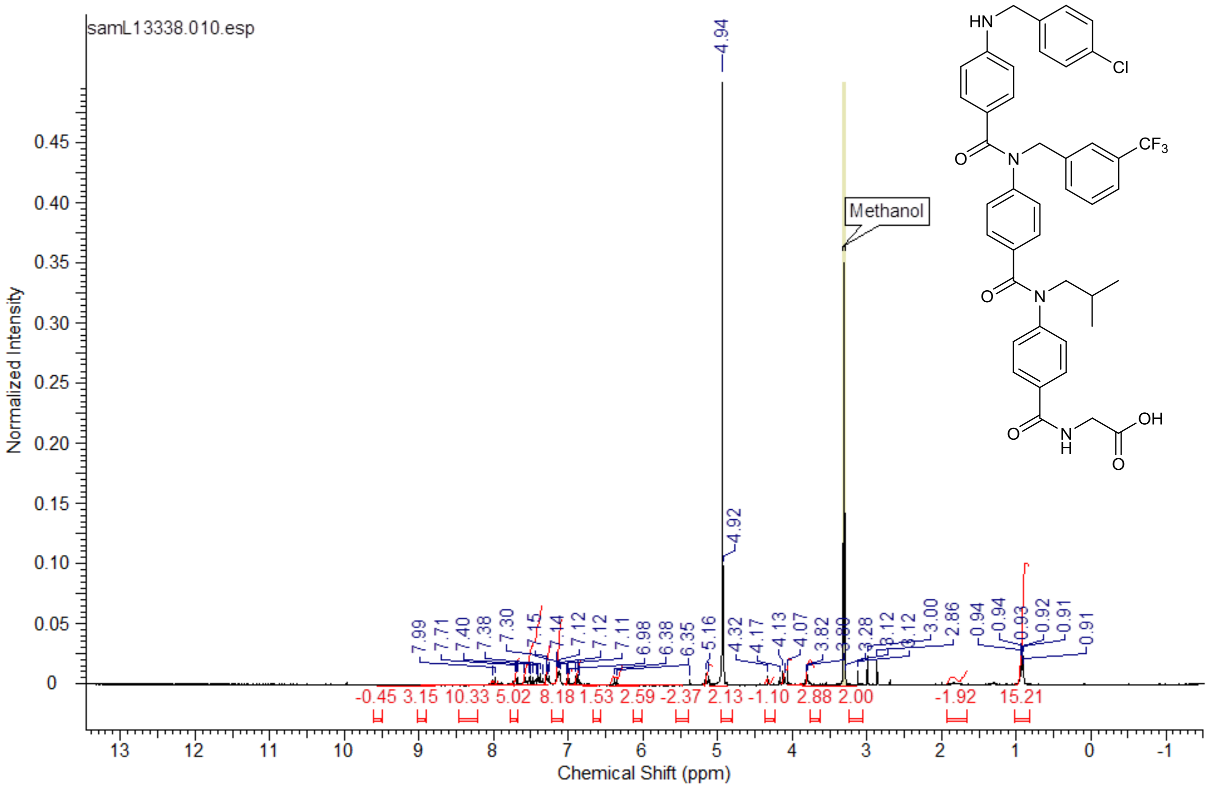

^{1}H NMR of Compound 14 in MeOD

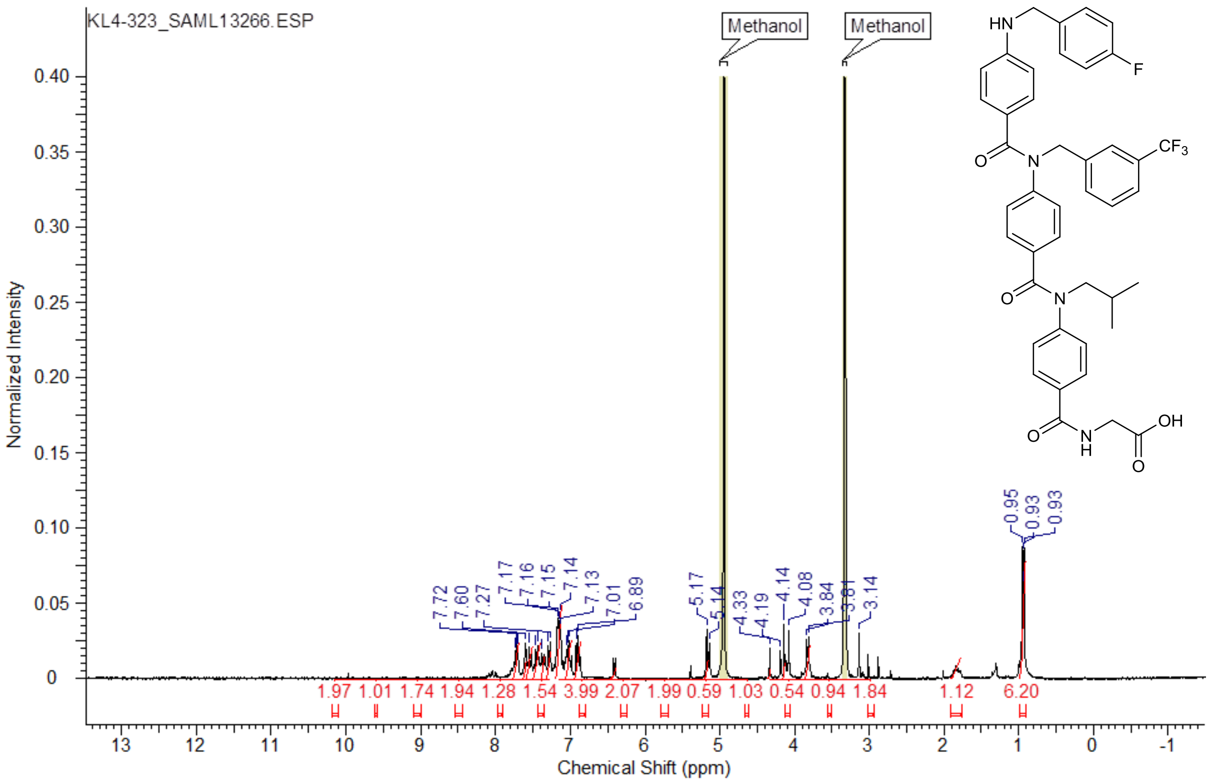

^{1}H NMR of Compound 15 in MeOD

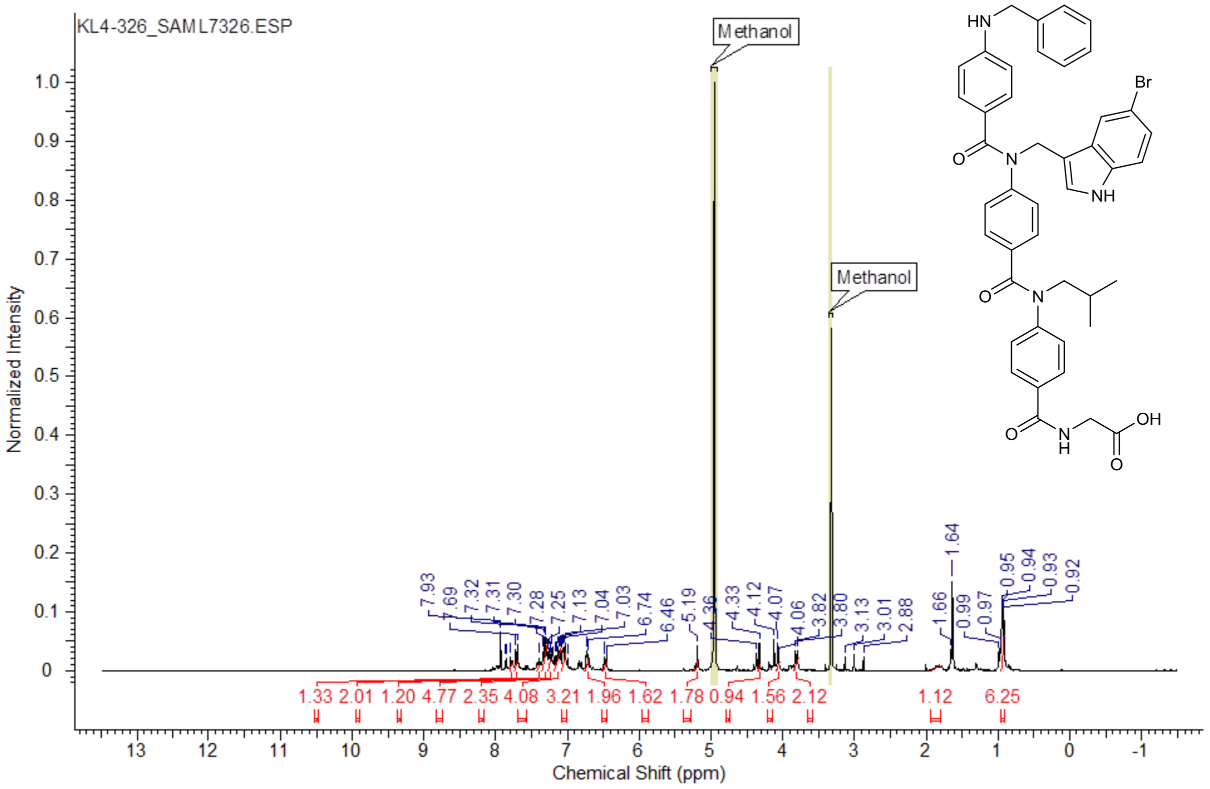

^{1}H NMR of Compound 16 in MeOD

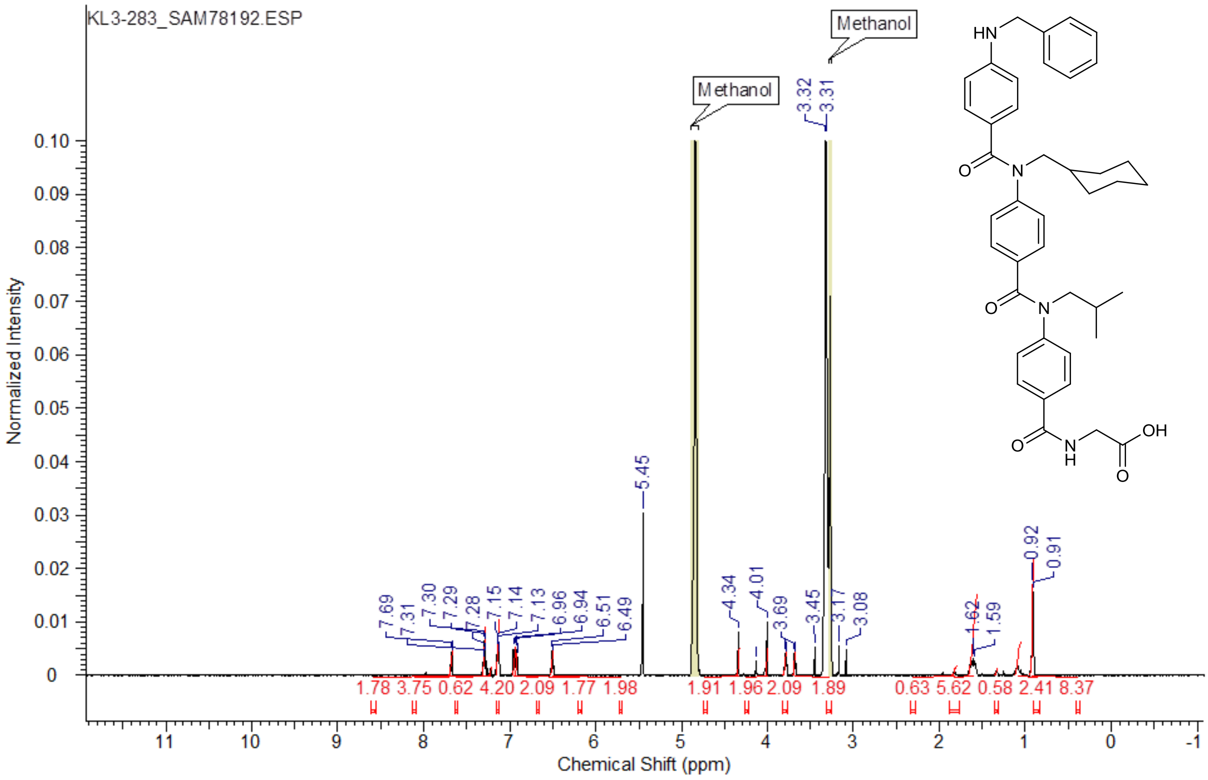

^{1}H NMR of Compound 18 in MeOD

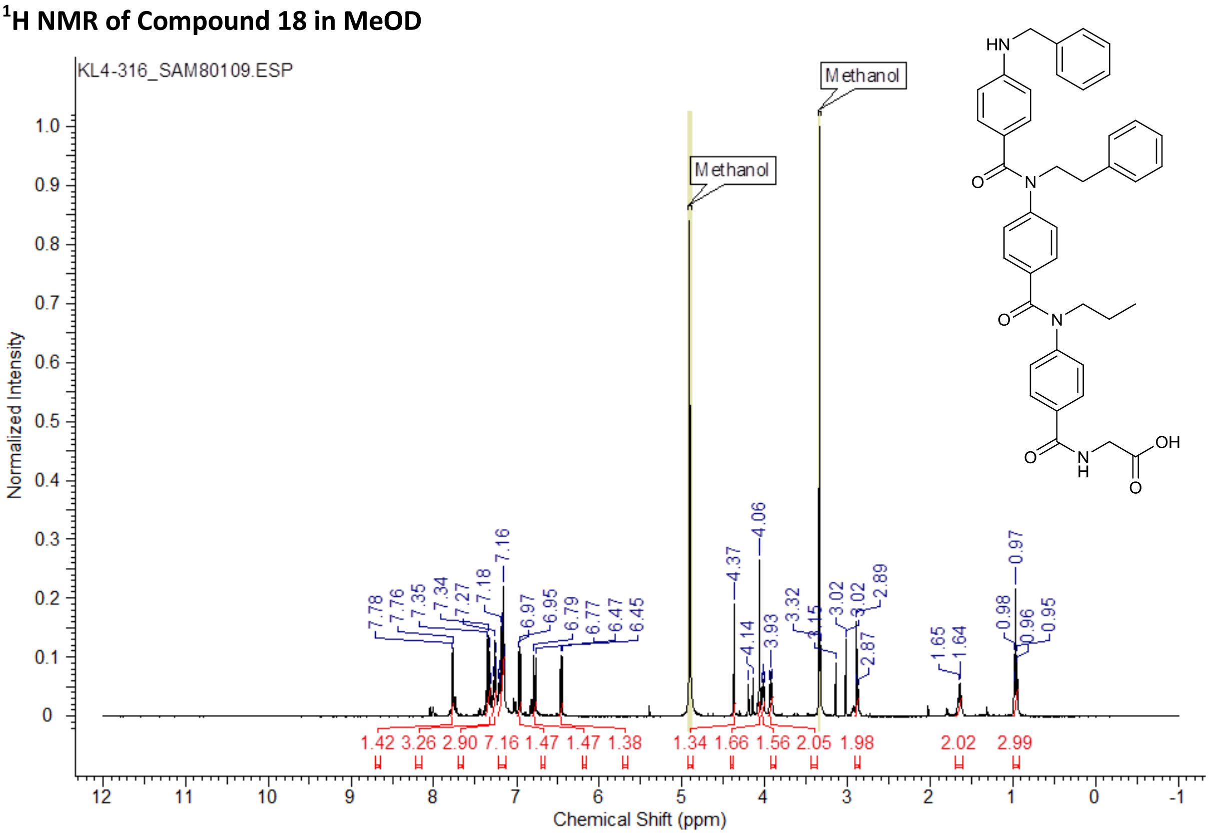

^{1}H NMR of Compound 20 in MeOD

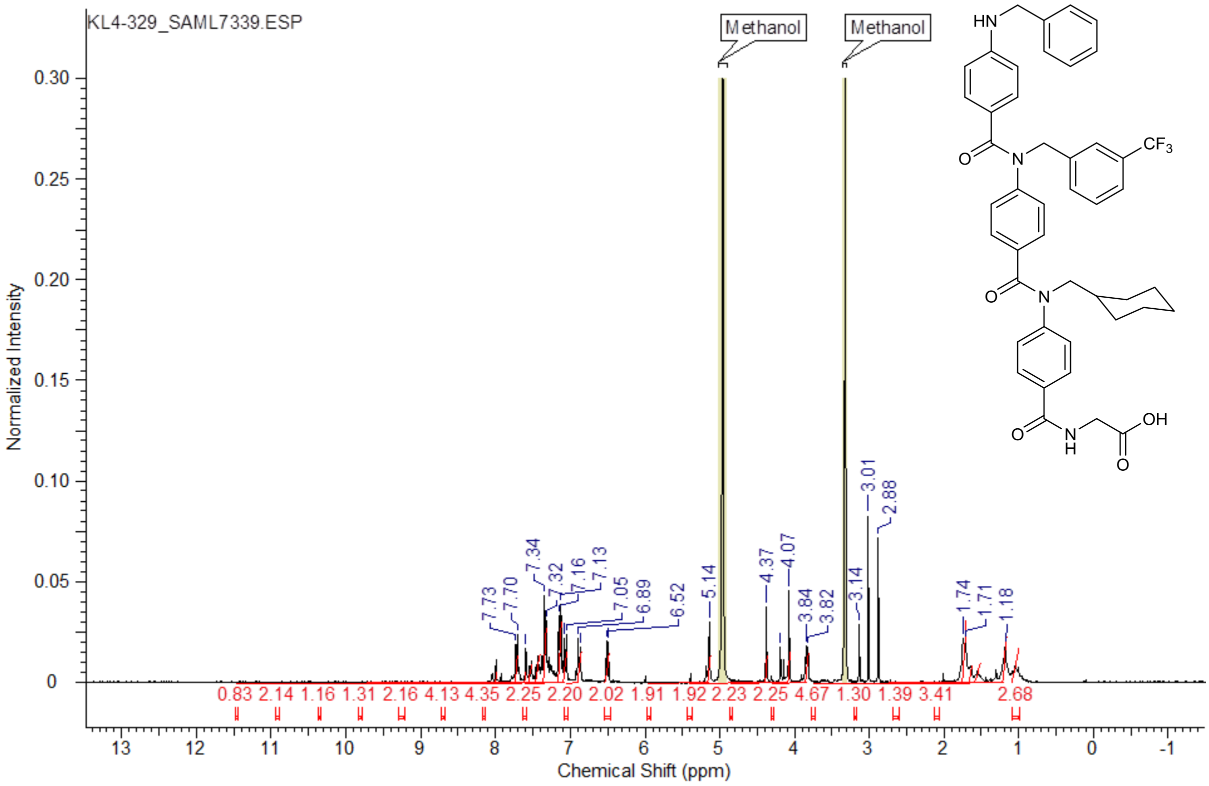

^{1}H NMR of Compound 21 in MeOD

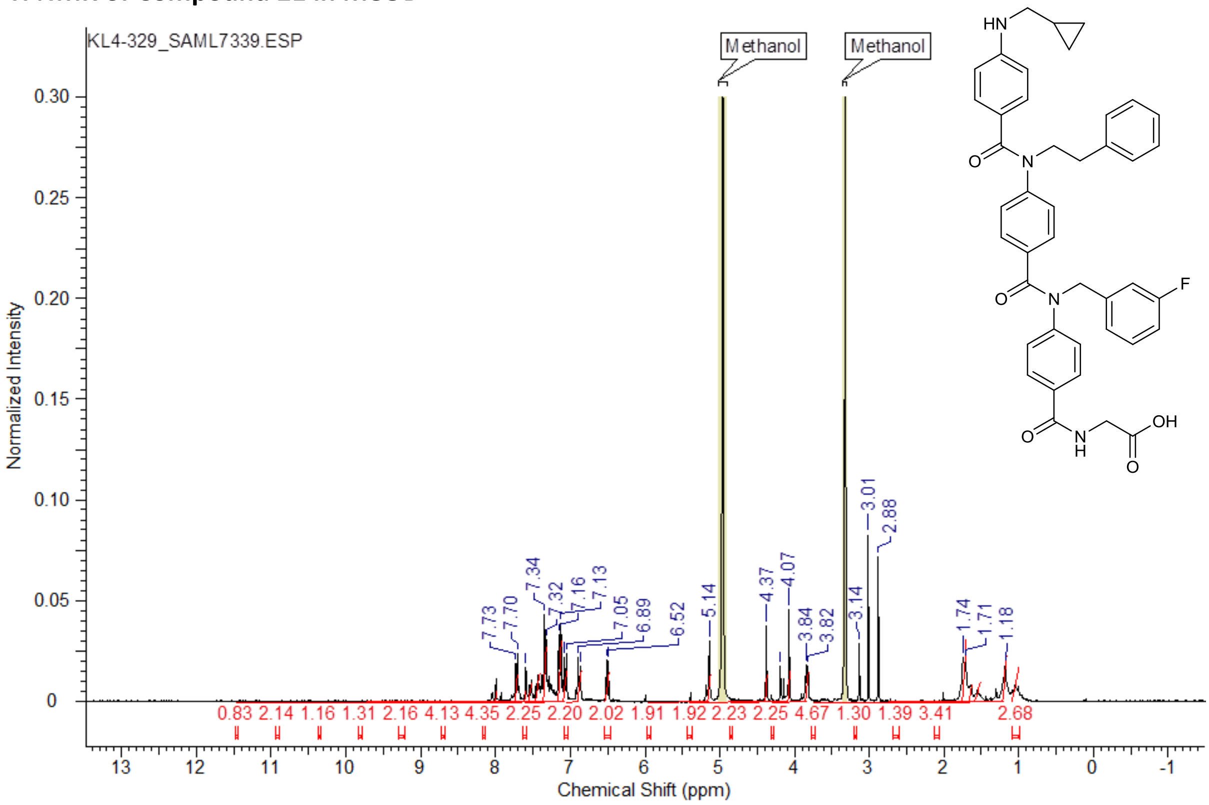

^{1}H NMR of Compound 22 in $CDCl_3$

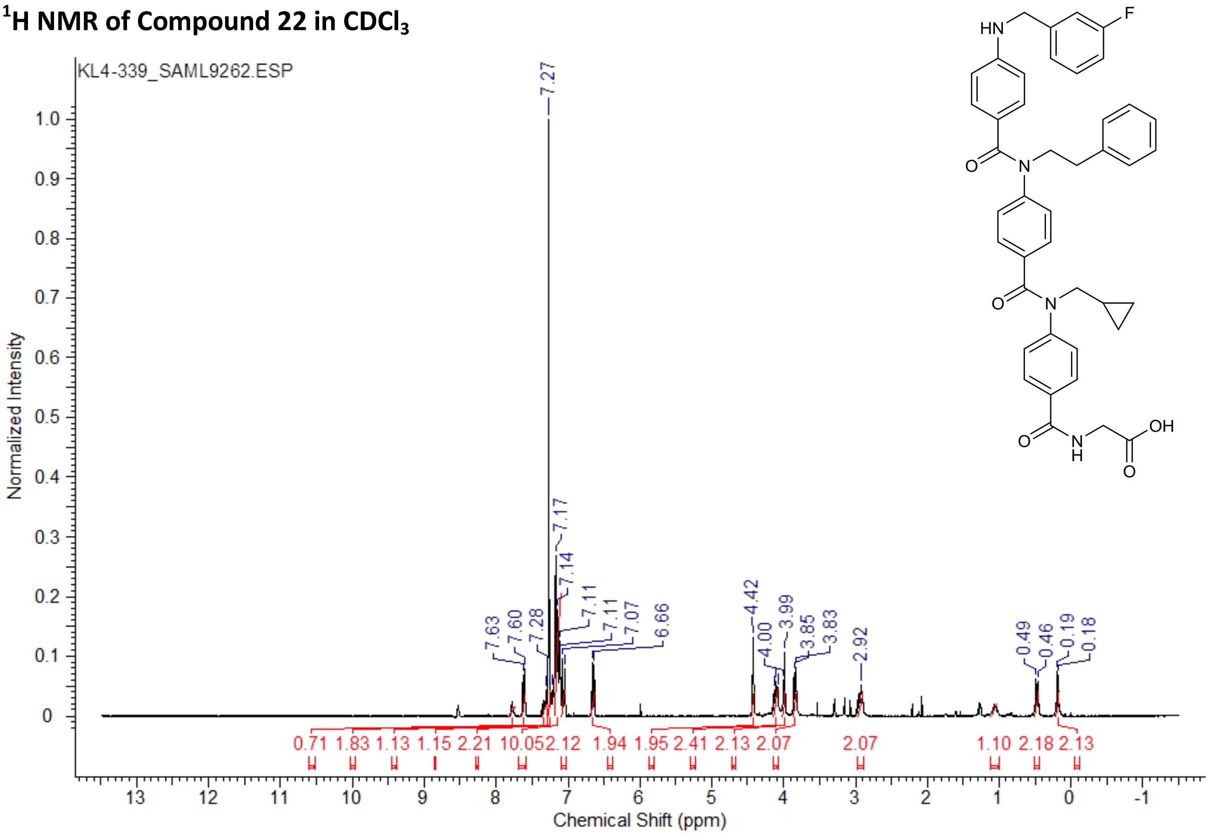

^{1}H NMR of Compound 23 in MeOD

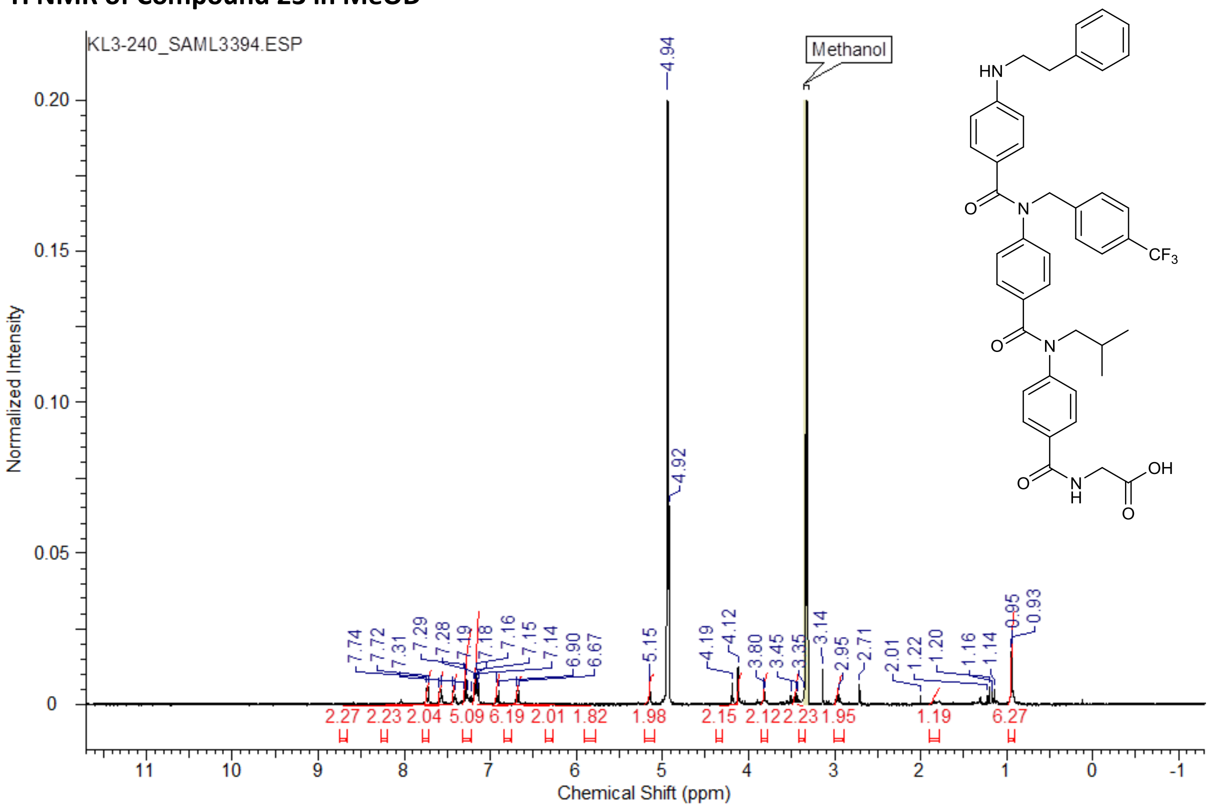

^{1}H NMR of Compound 24 in MeOD

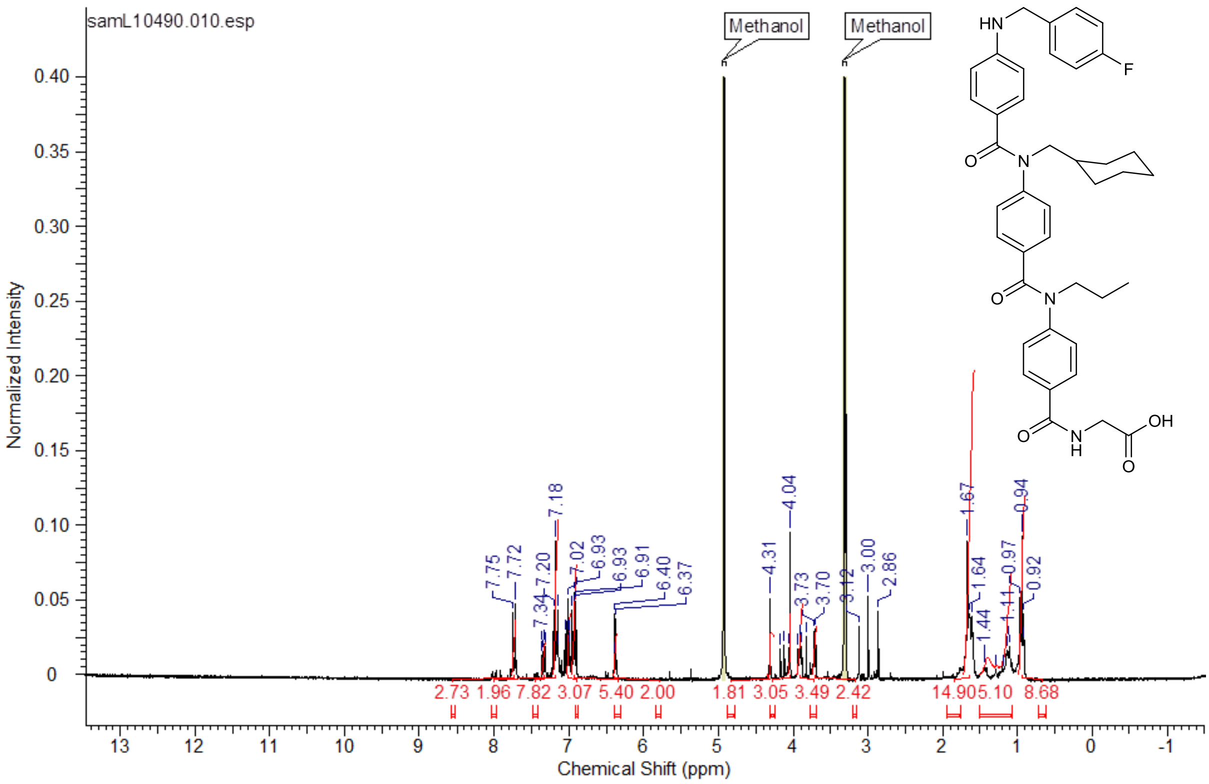

^{1}H NMR of Compound 27 in $CDCl_3$

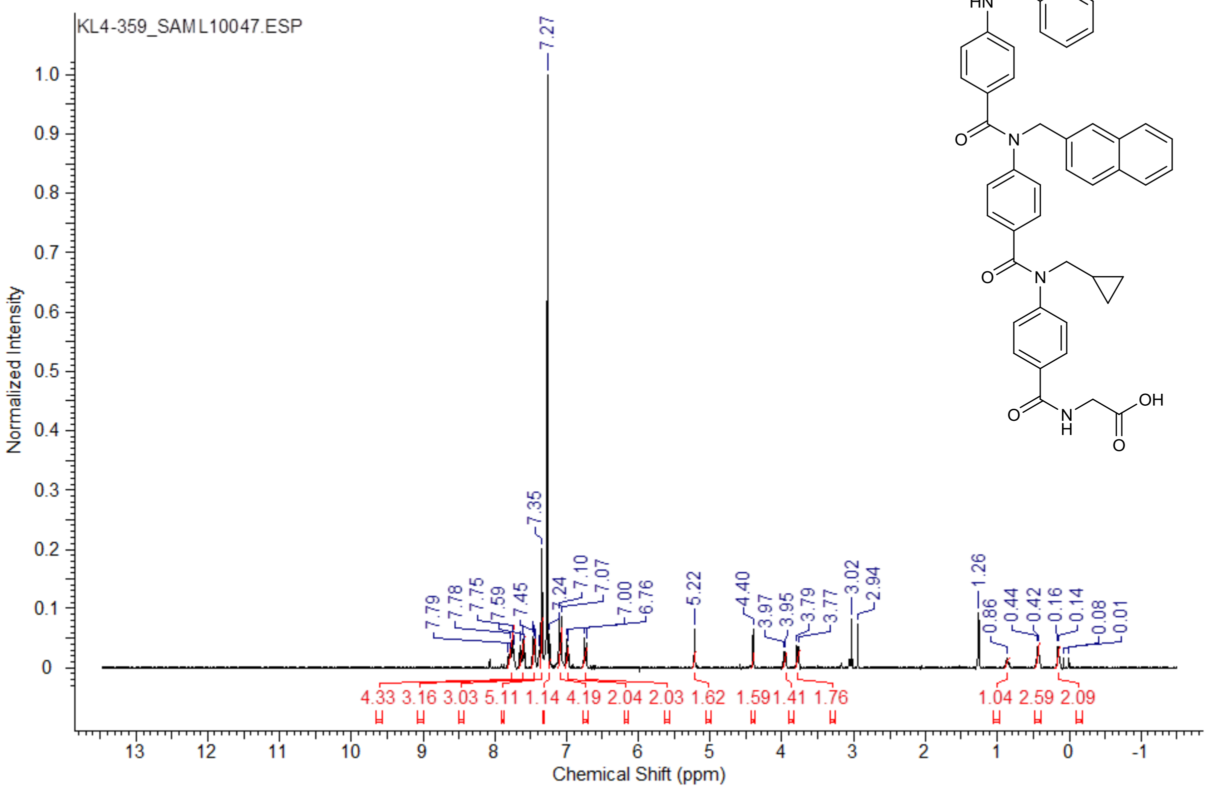

^{1}H NMR of Compound 28 in $CDCl_3$

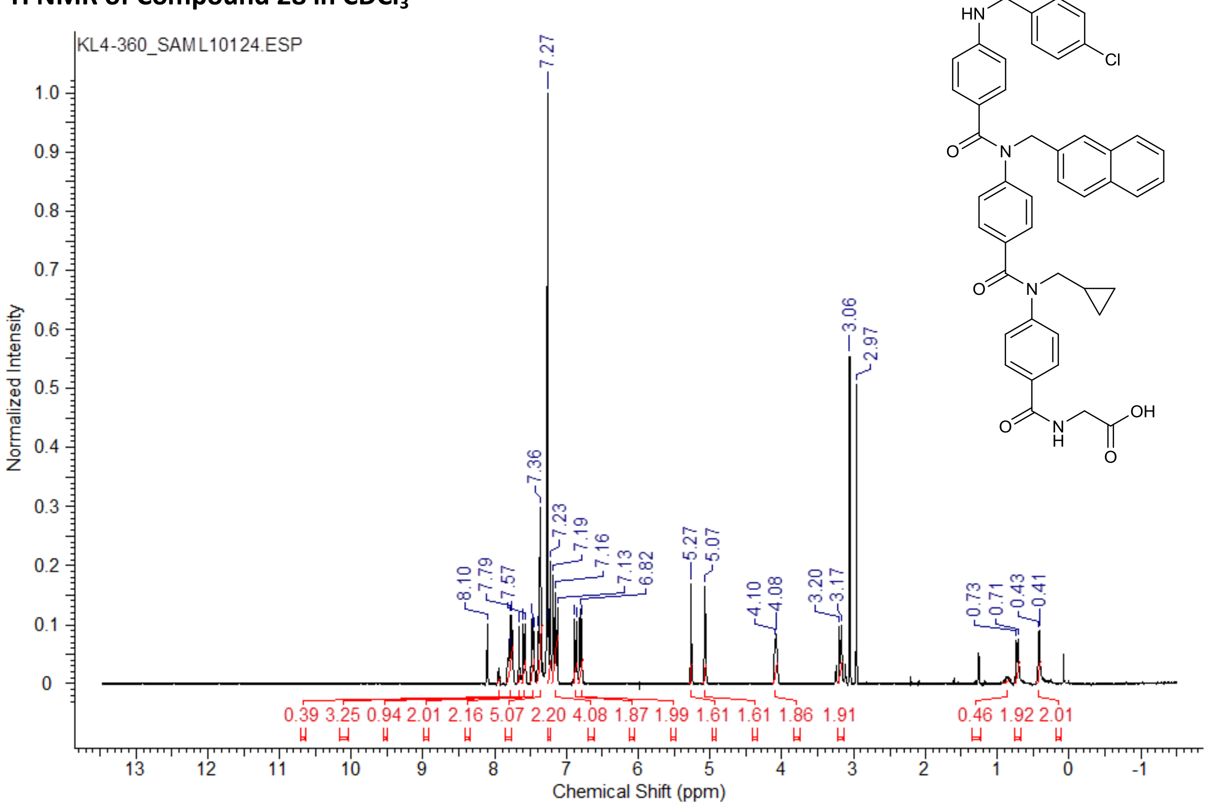

^{1}H NMR of Compound 29 in MeOD

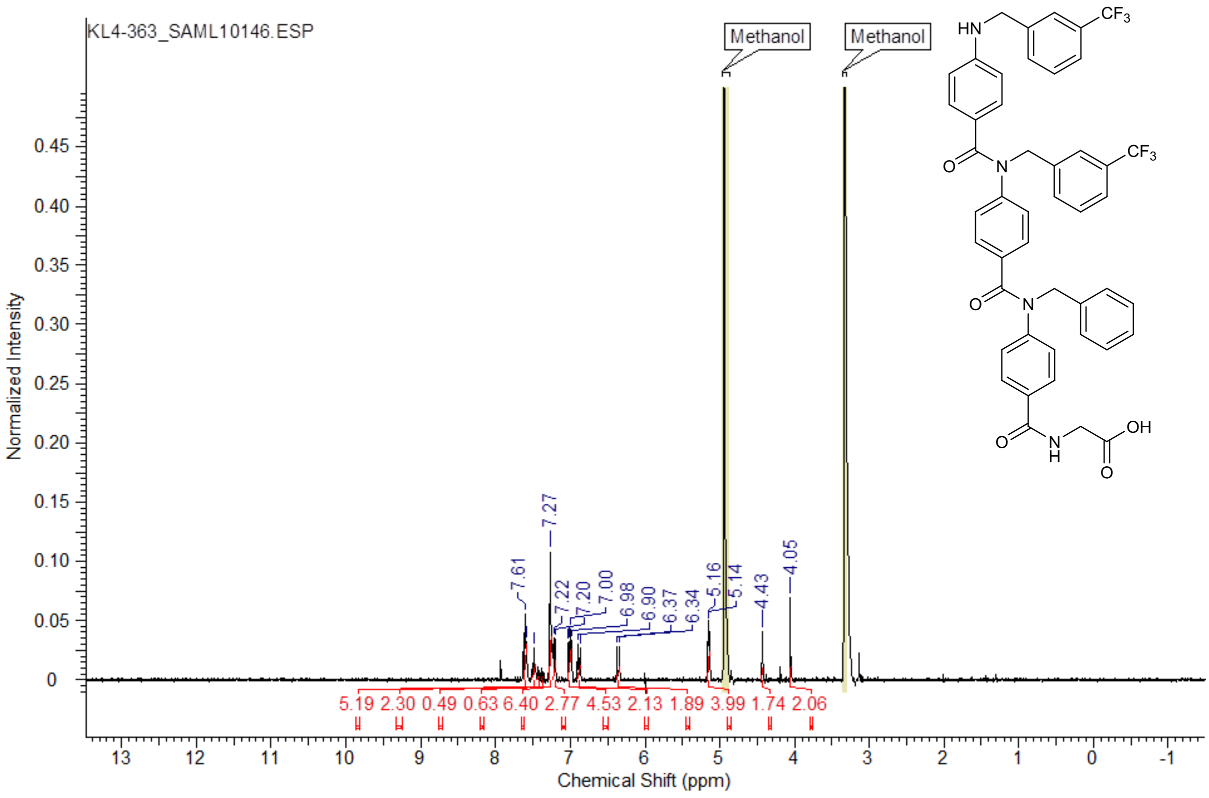

^{1}H NMR of Compound 30 in MeOD

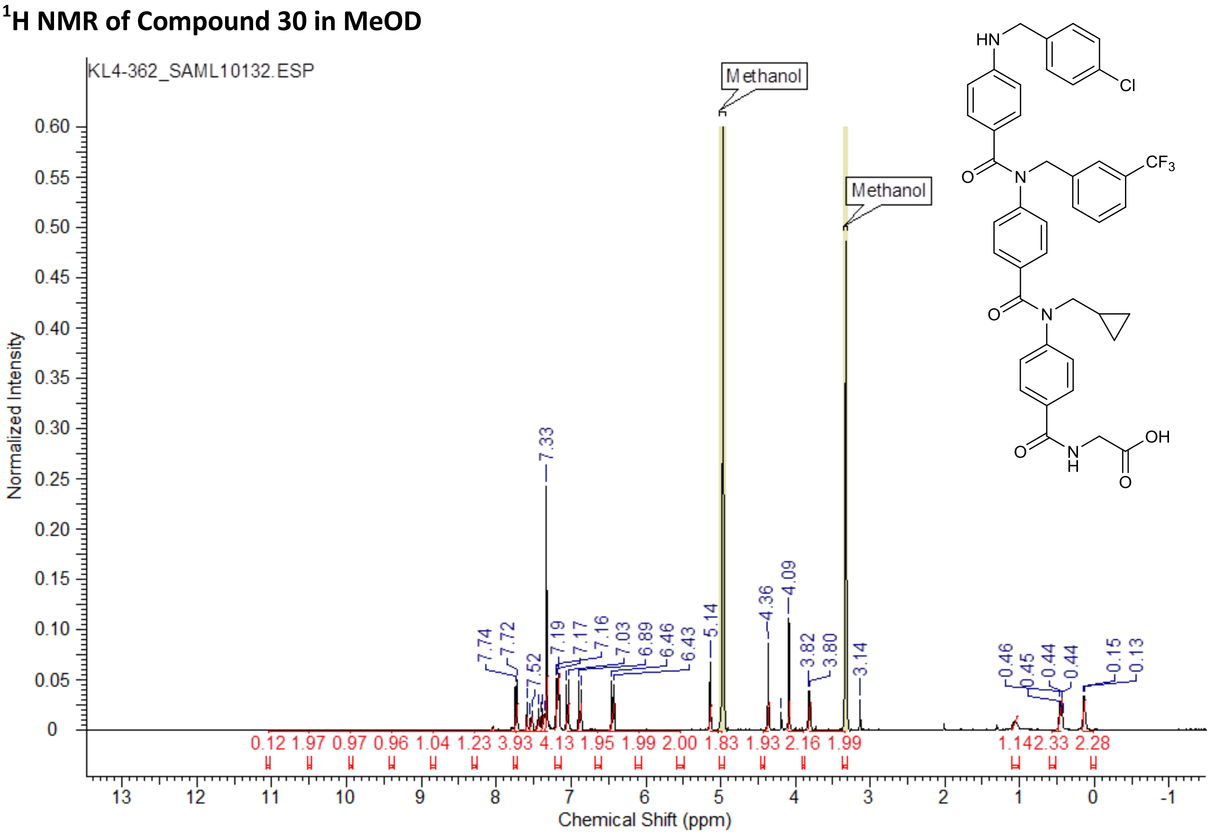

^{1}H NMR of Compound 31 in MeOD

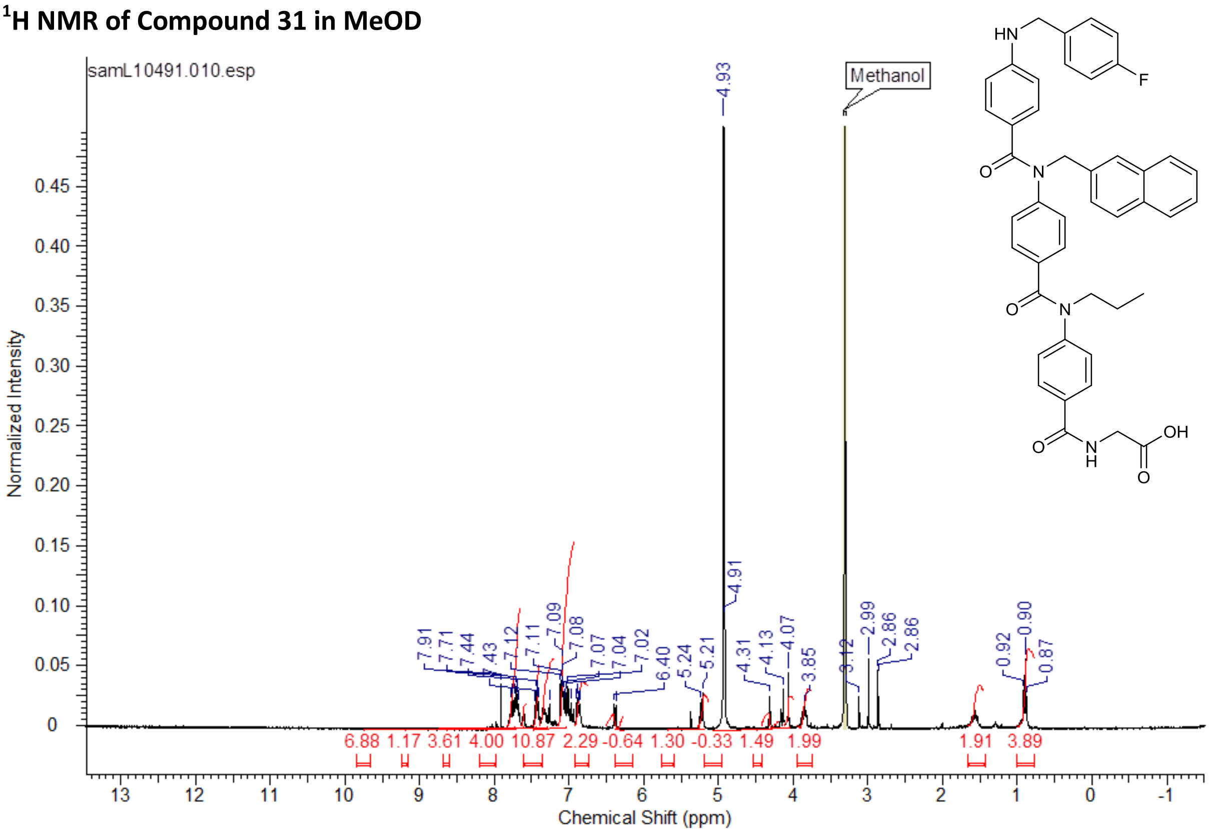

^{1}H NMR of Compound 32 in DMSO-d$_6$

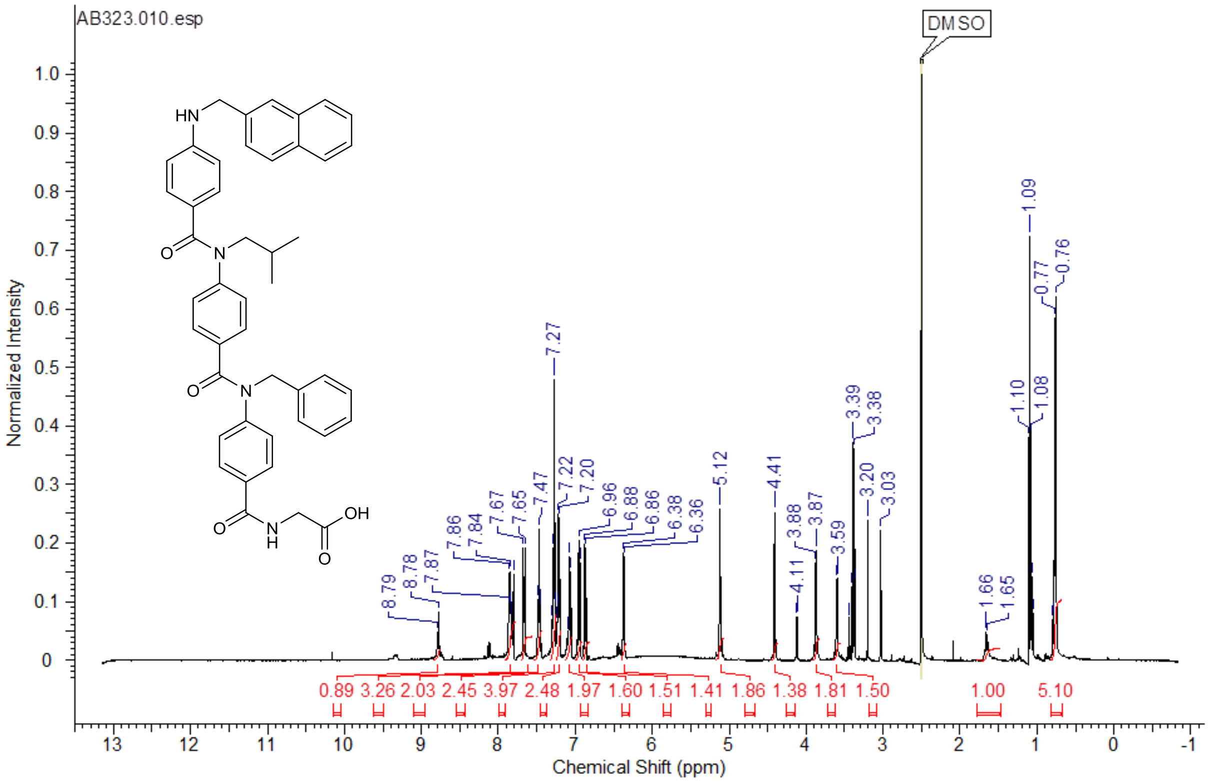

^{1}H NMR of Compound 33 in MeOD

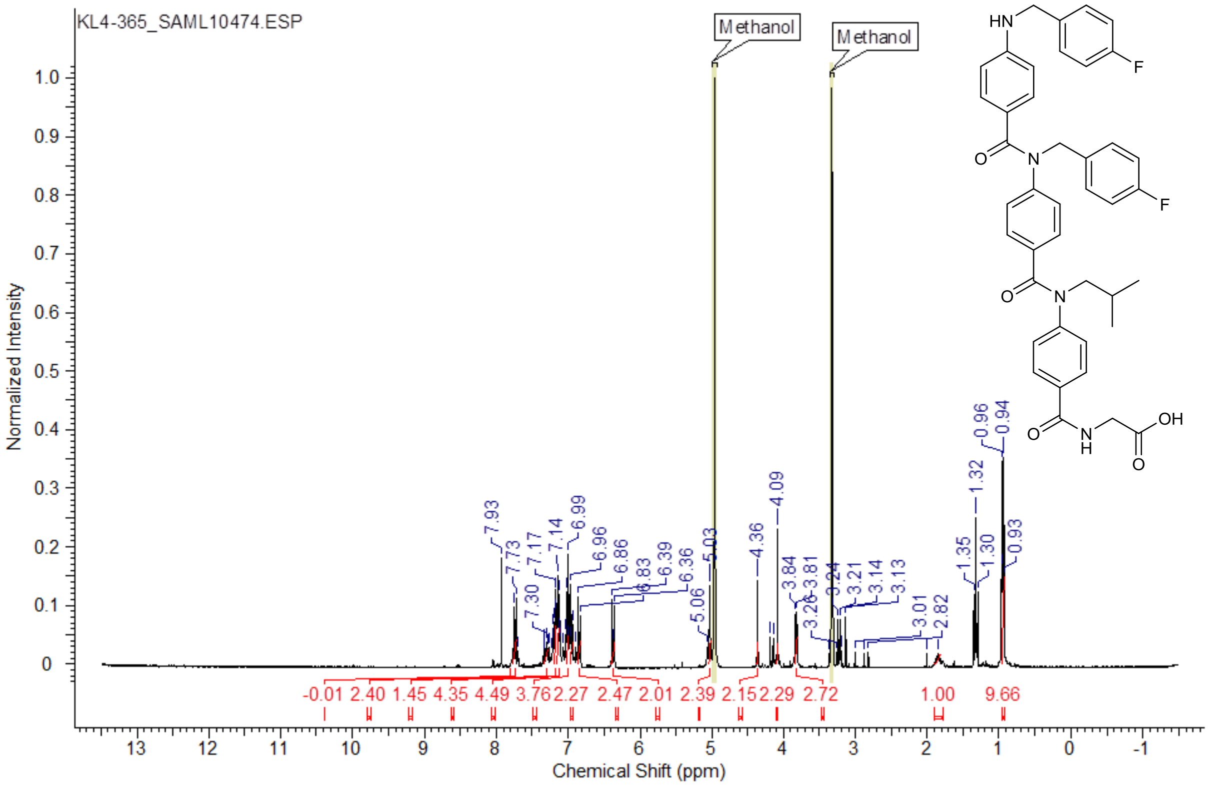

^{1}H NMR of Compound 34 in MeOD

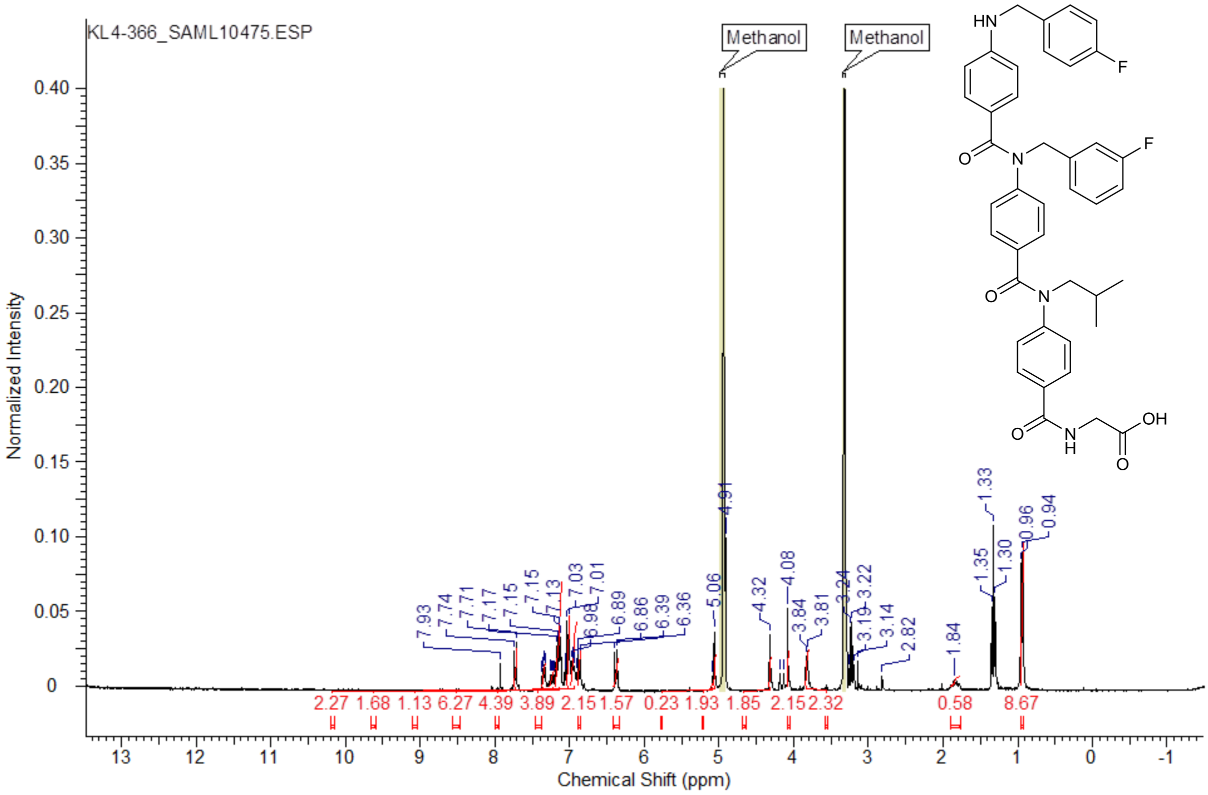

^{1}H NMR of Compound 35 in MeOD

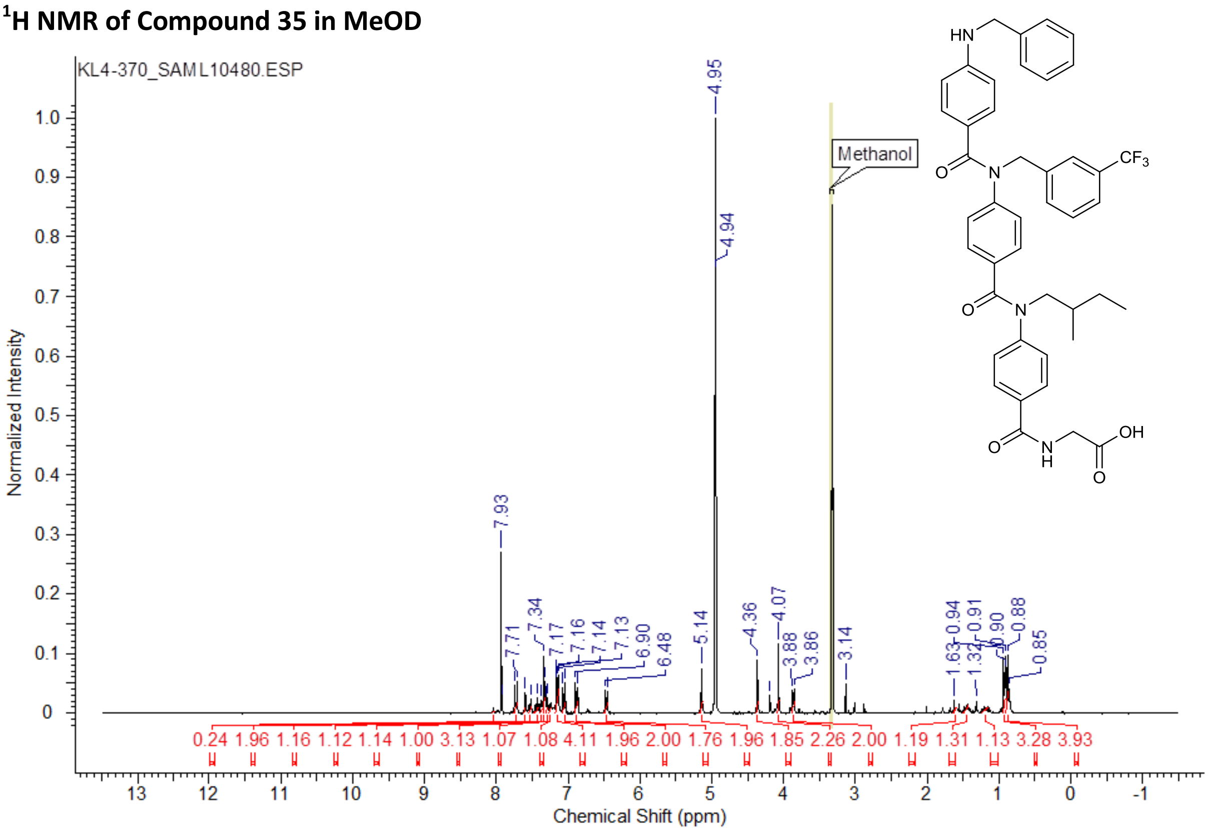

^{1}H NMR of Compound 36 in MeOD

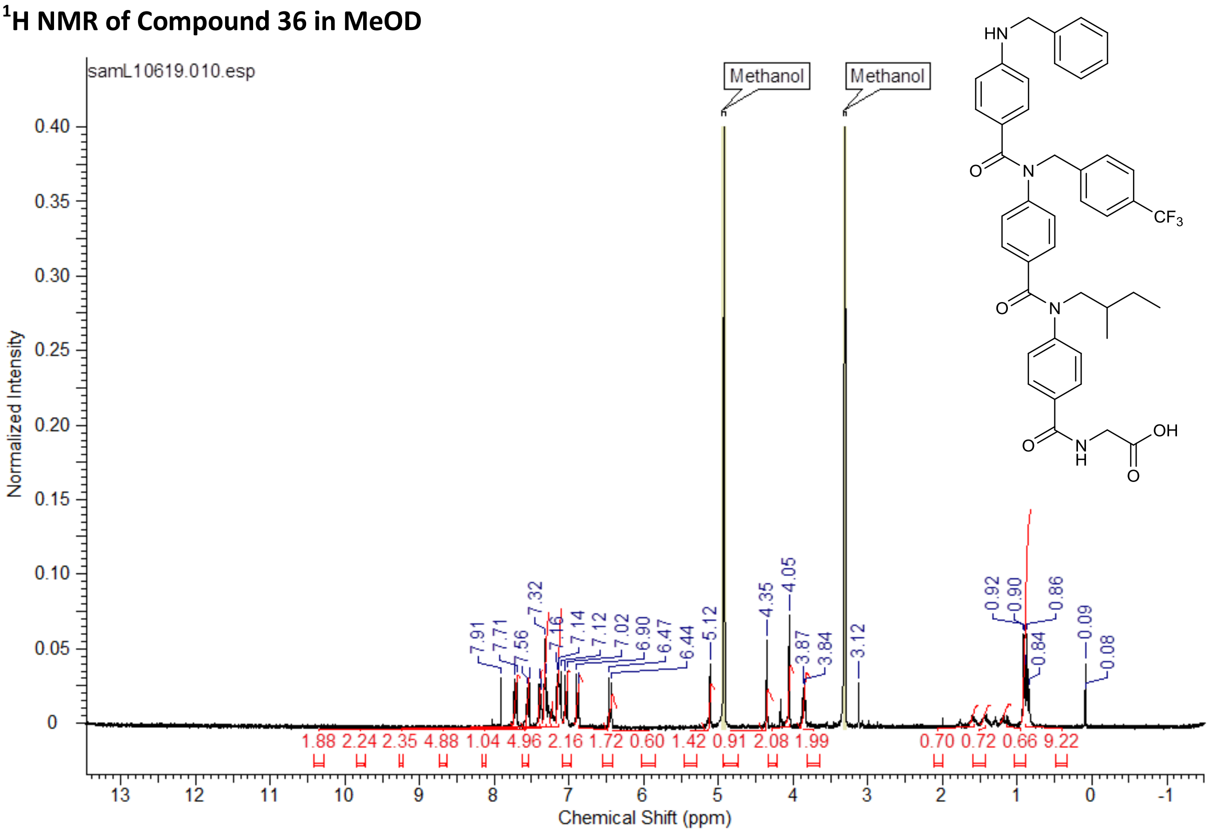

^{1}H NMR of Compound 37 in MeOD

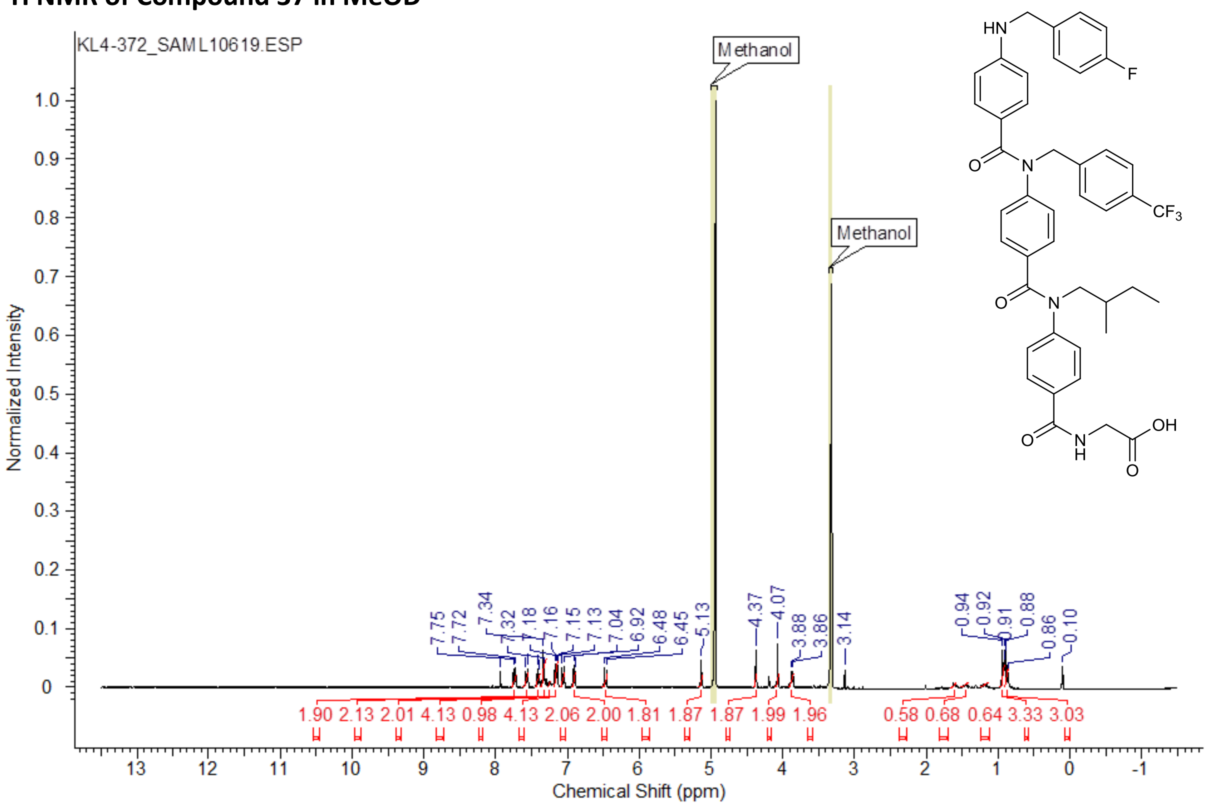

^{1}H NMR of Compound 39 in MeOD

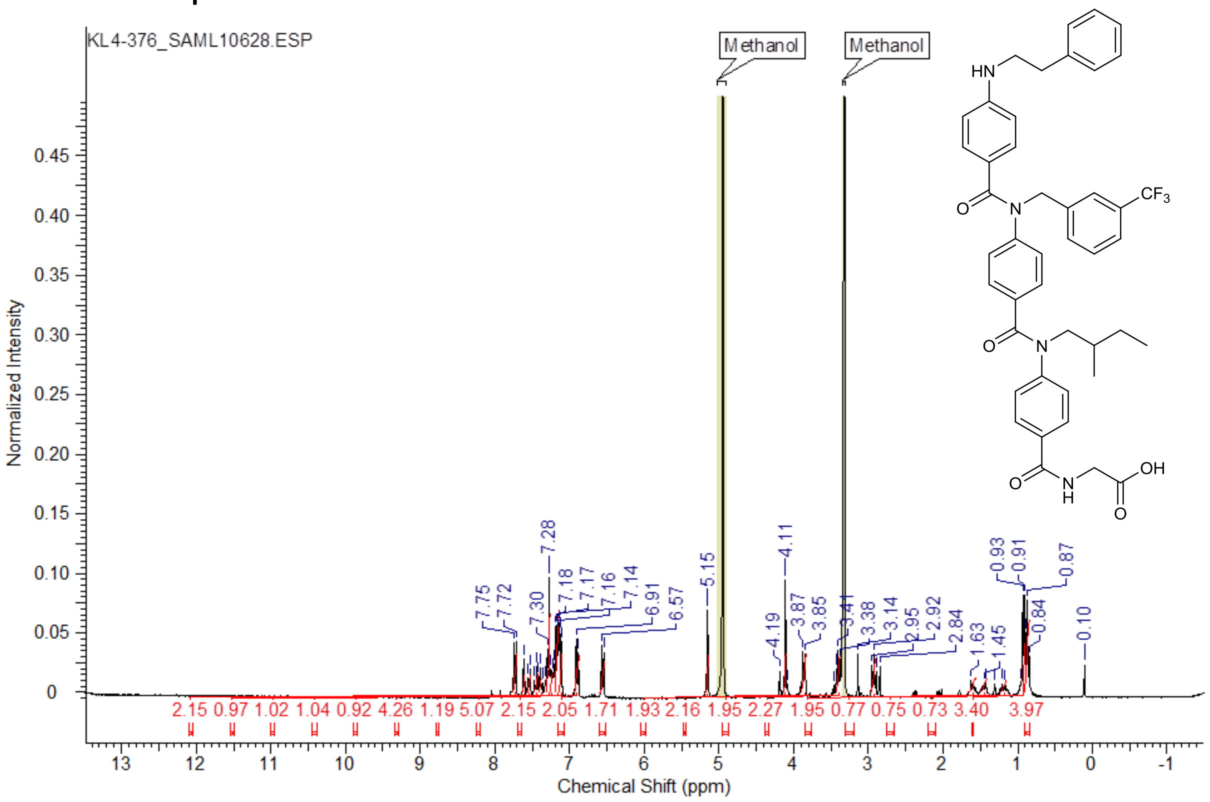

^{1}H NMR of Compound 40 in MeOD

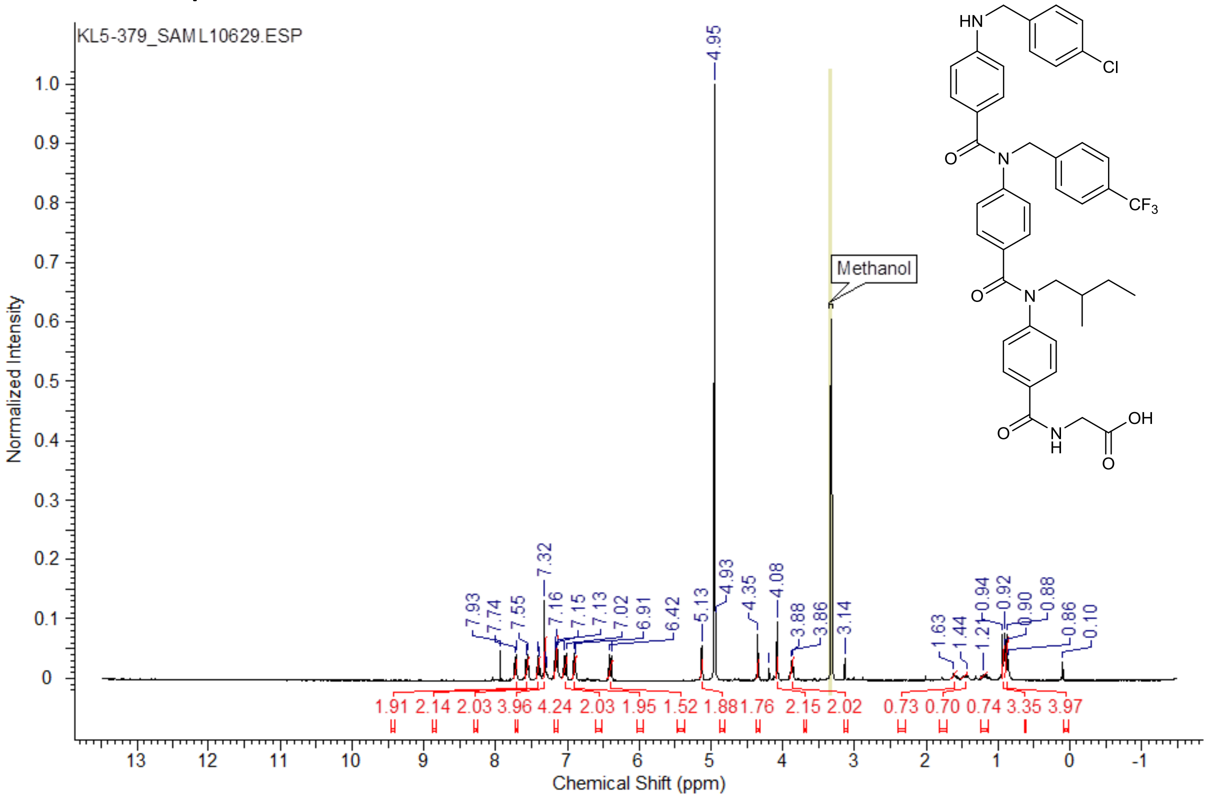

^{1}H NMR of Compound 41 in MeOD

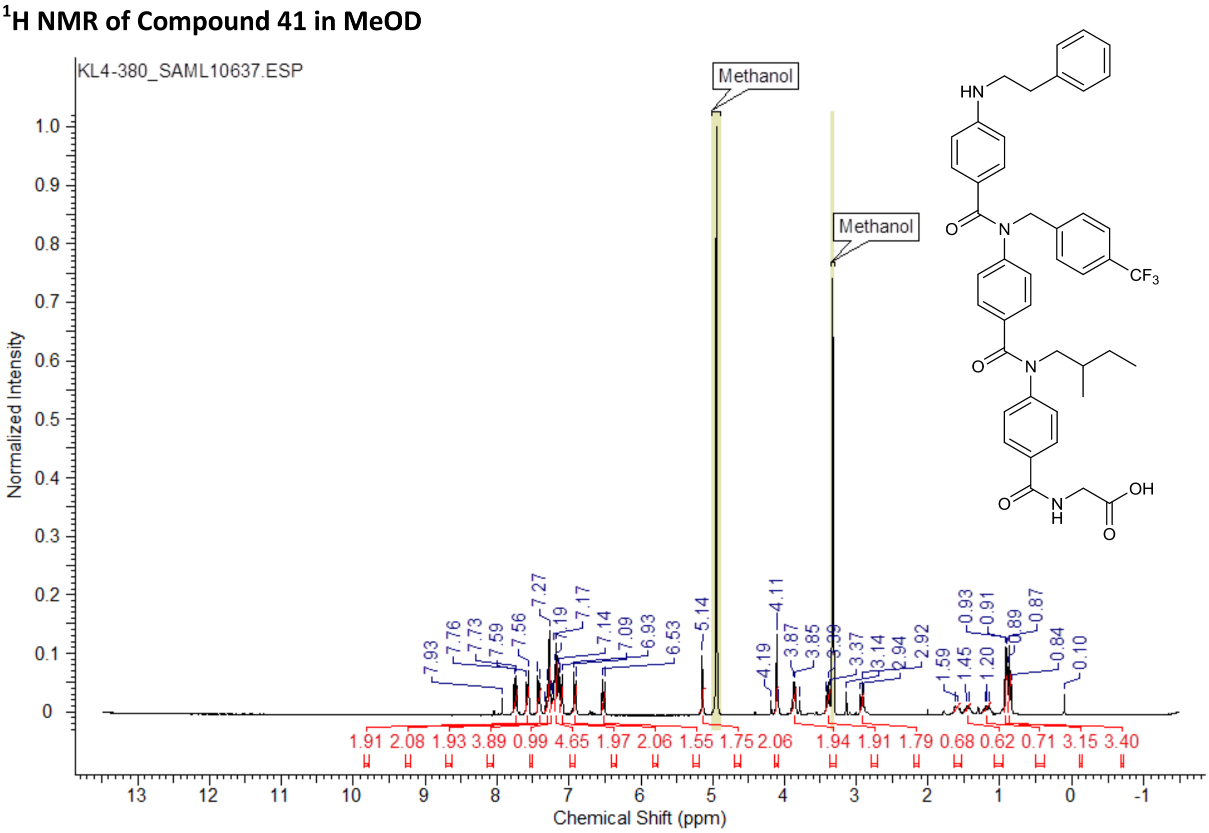

^{1}H NMR of Compound 42 in MeOD

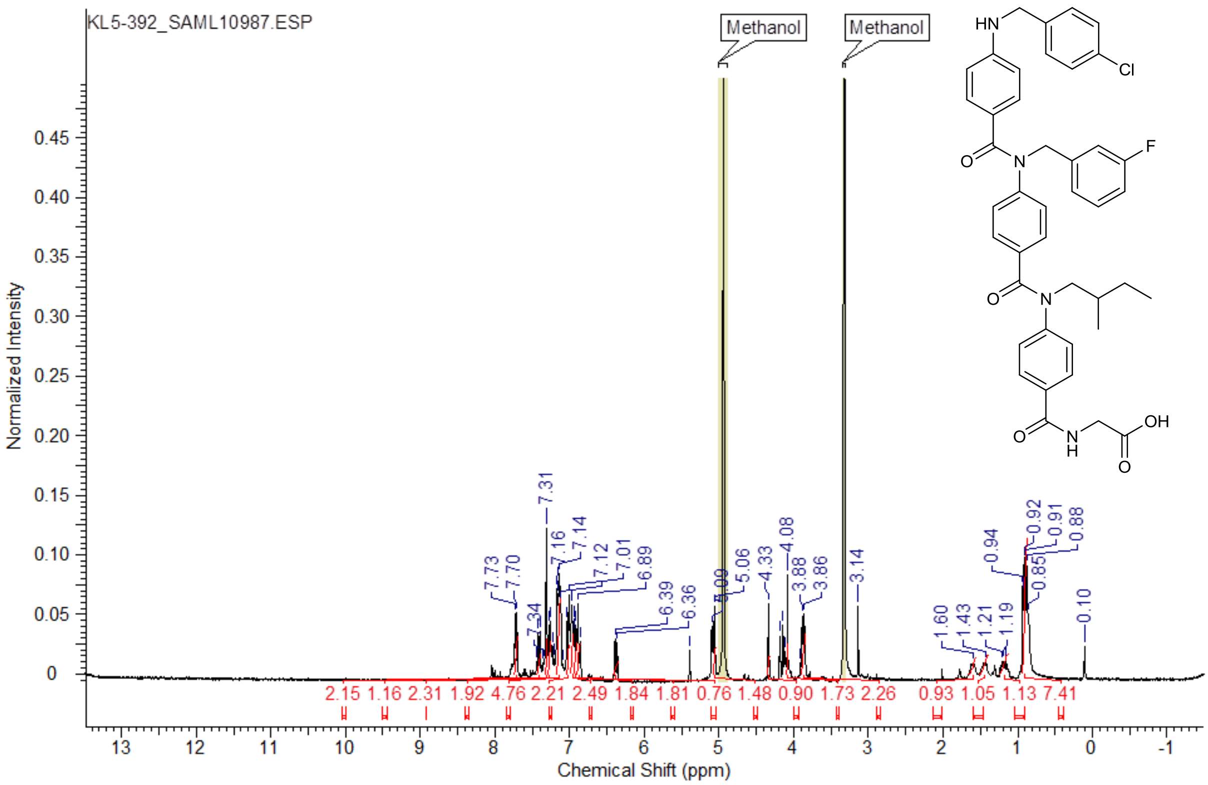

^{1}H NMR of Compound 43 in MeOD

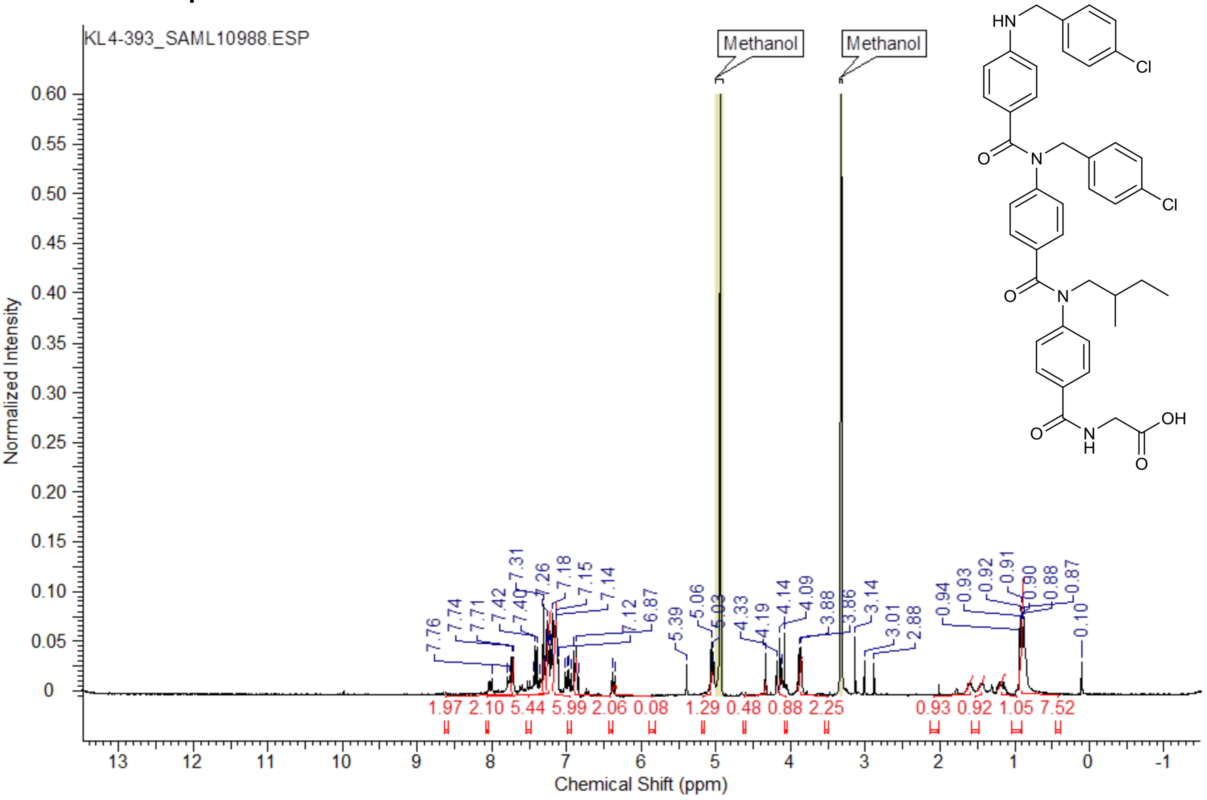

^{1}H NMR of Compound 45 in MeOD

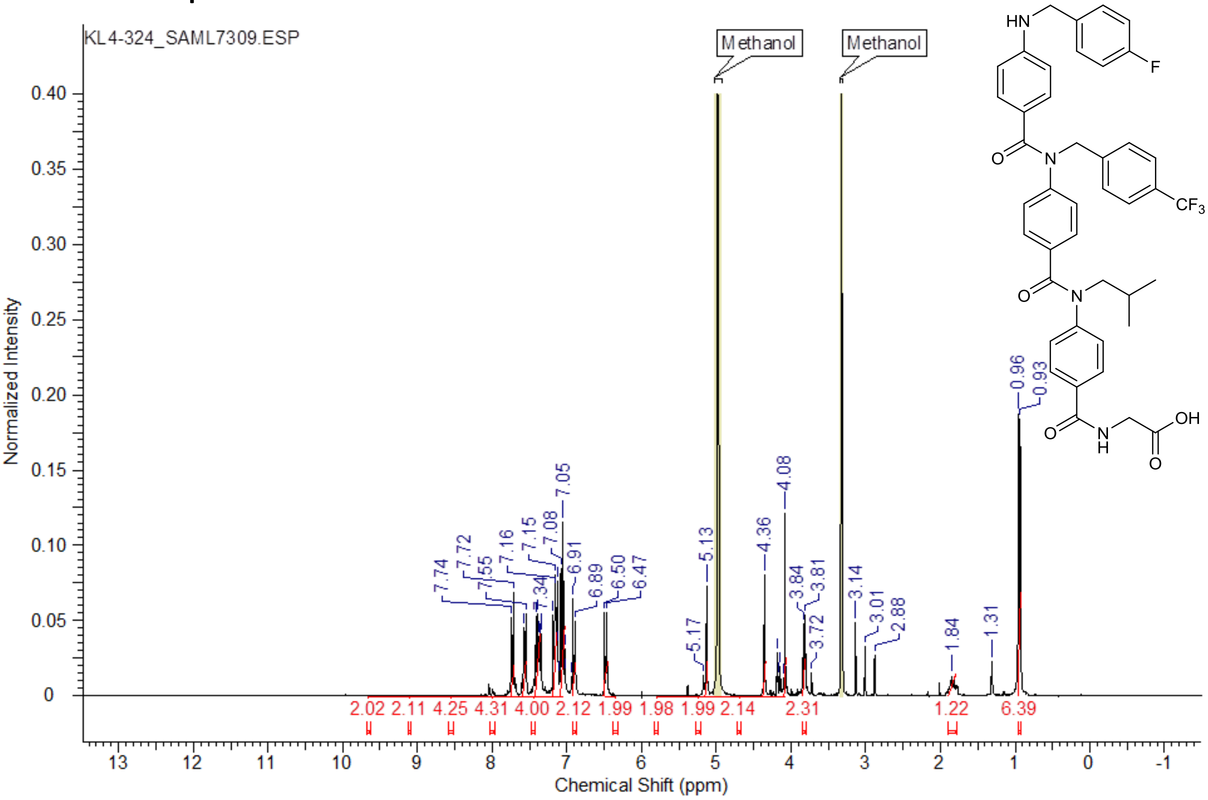

¹H NMR of Compound 46 in MeOD

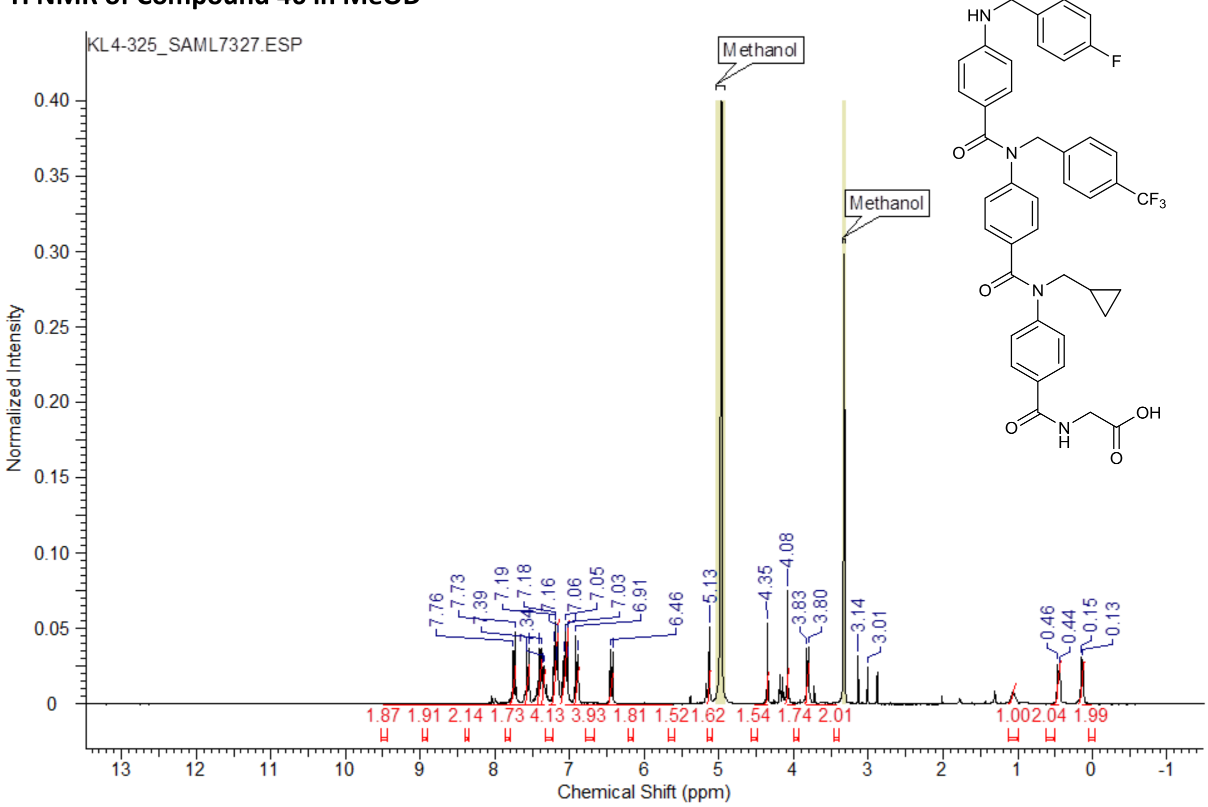

¹H NMR of Compound 47 in MeOD

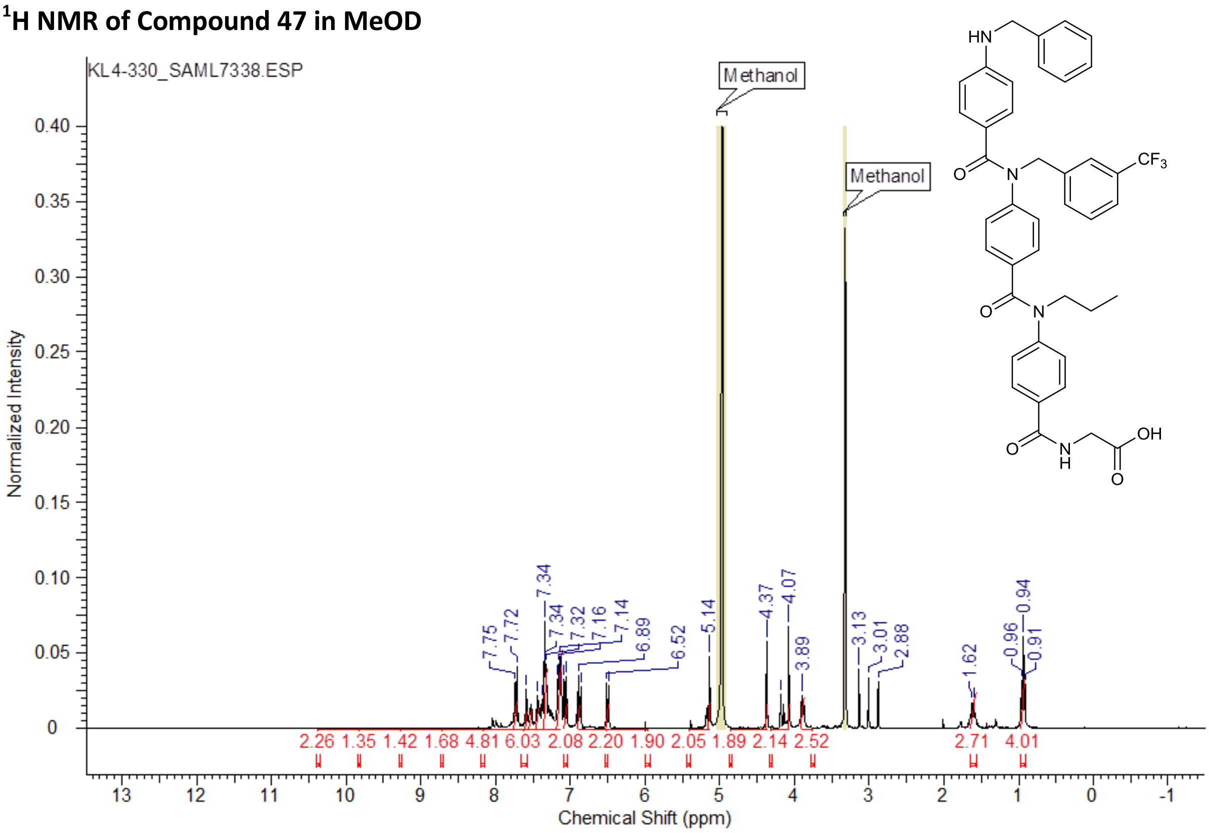

^{1}H NMR of Compound 48 in MeOD

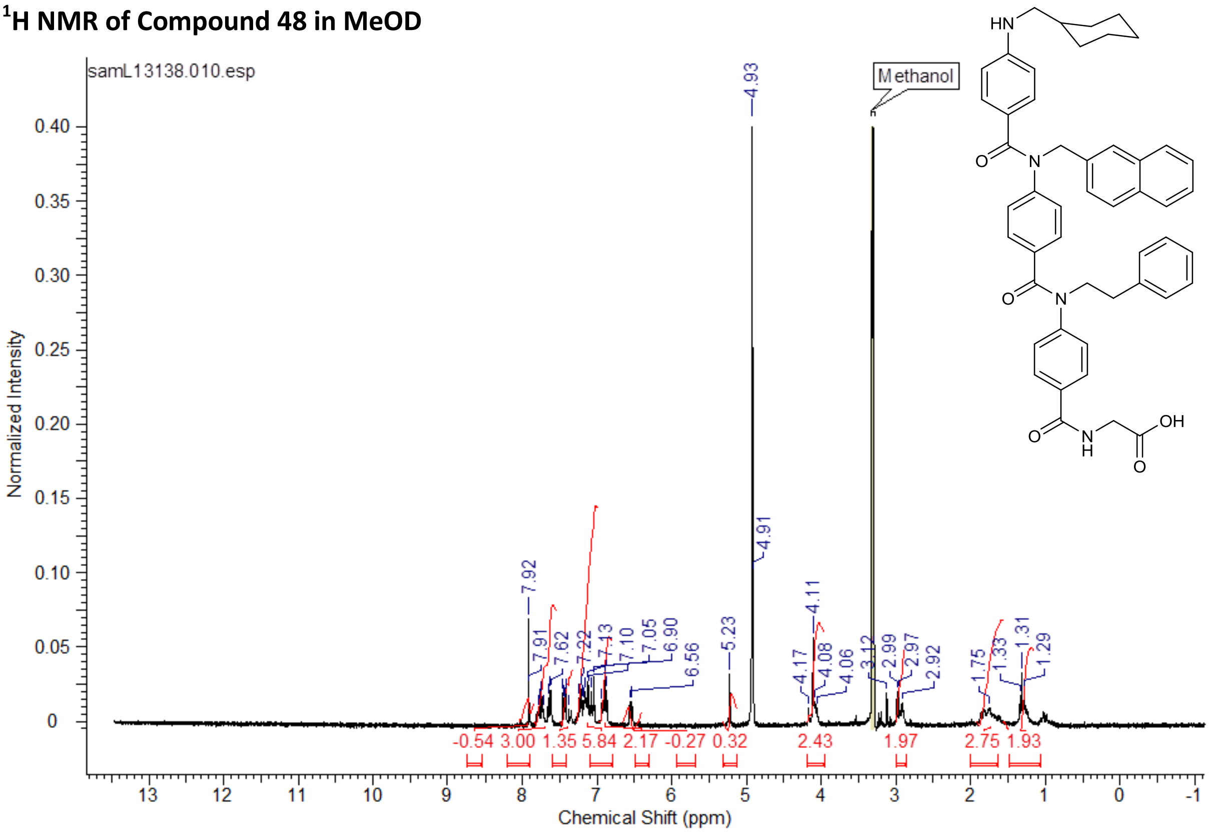

^{1}H NMR of Compound 49 in MeOD

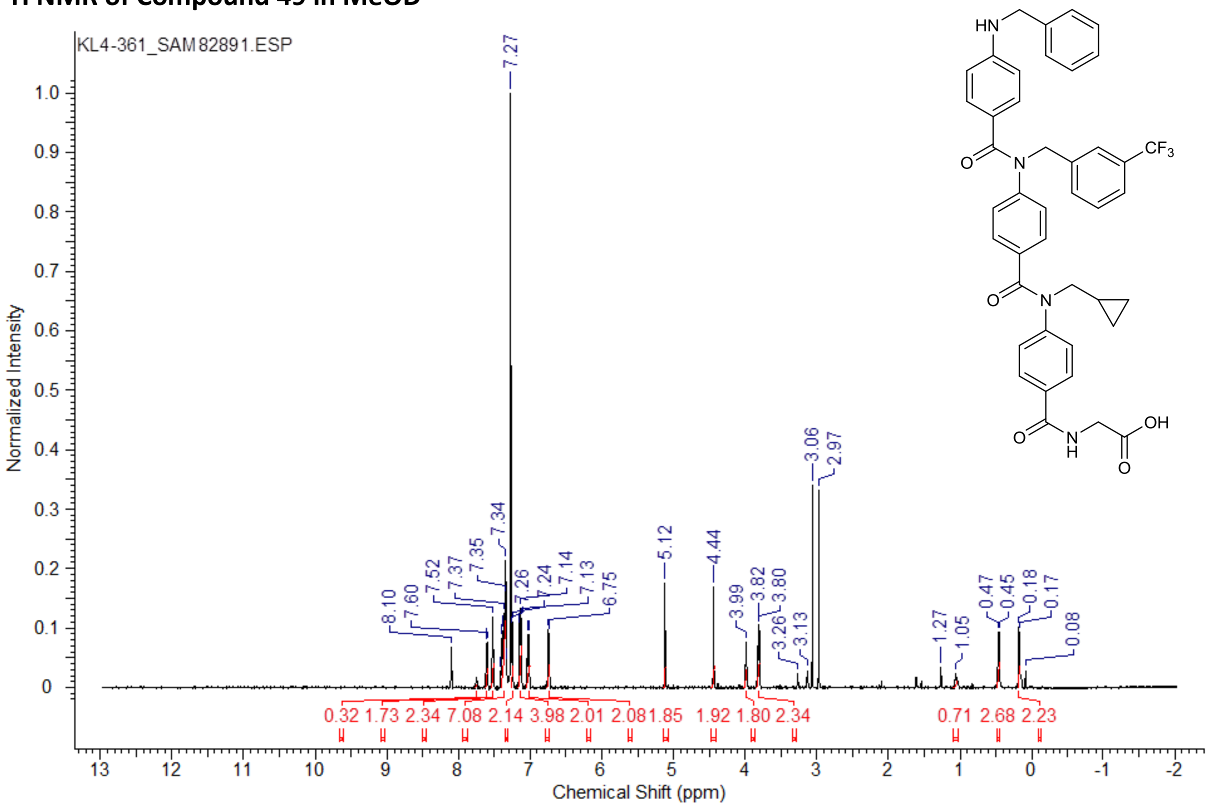

^{1}H NMR of Compound 50 in MeOD

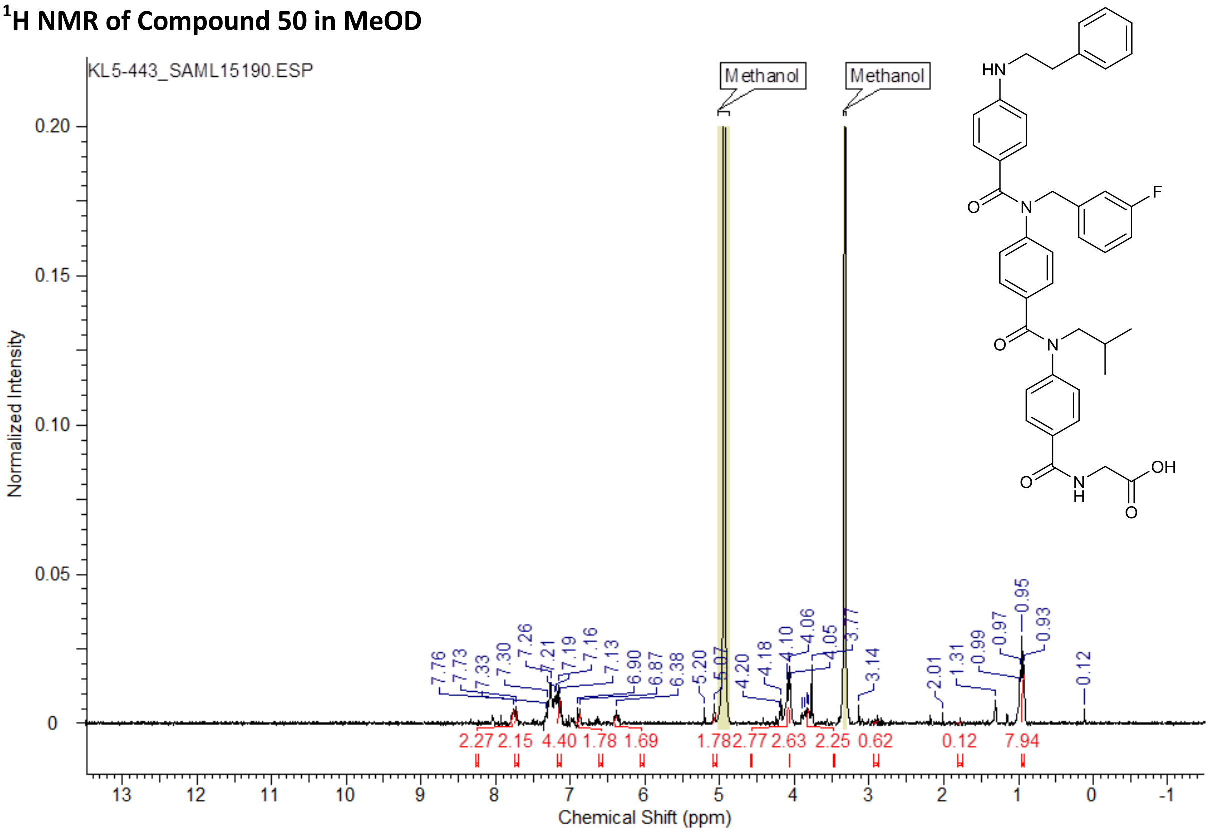

^{1}H NMR of Compound 51 in MeOD

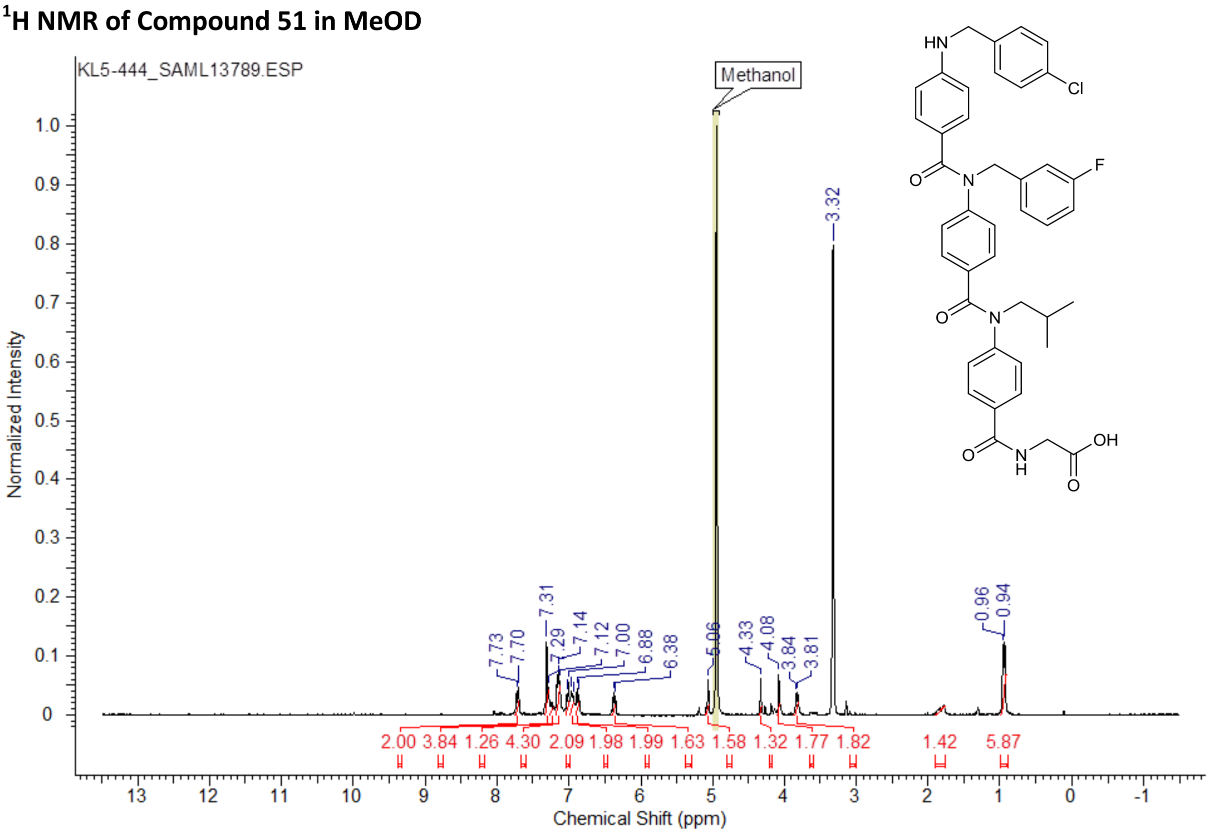

^{1}H NMR of Compound 52 in MeOD

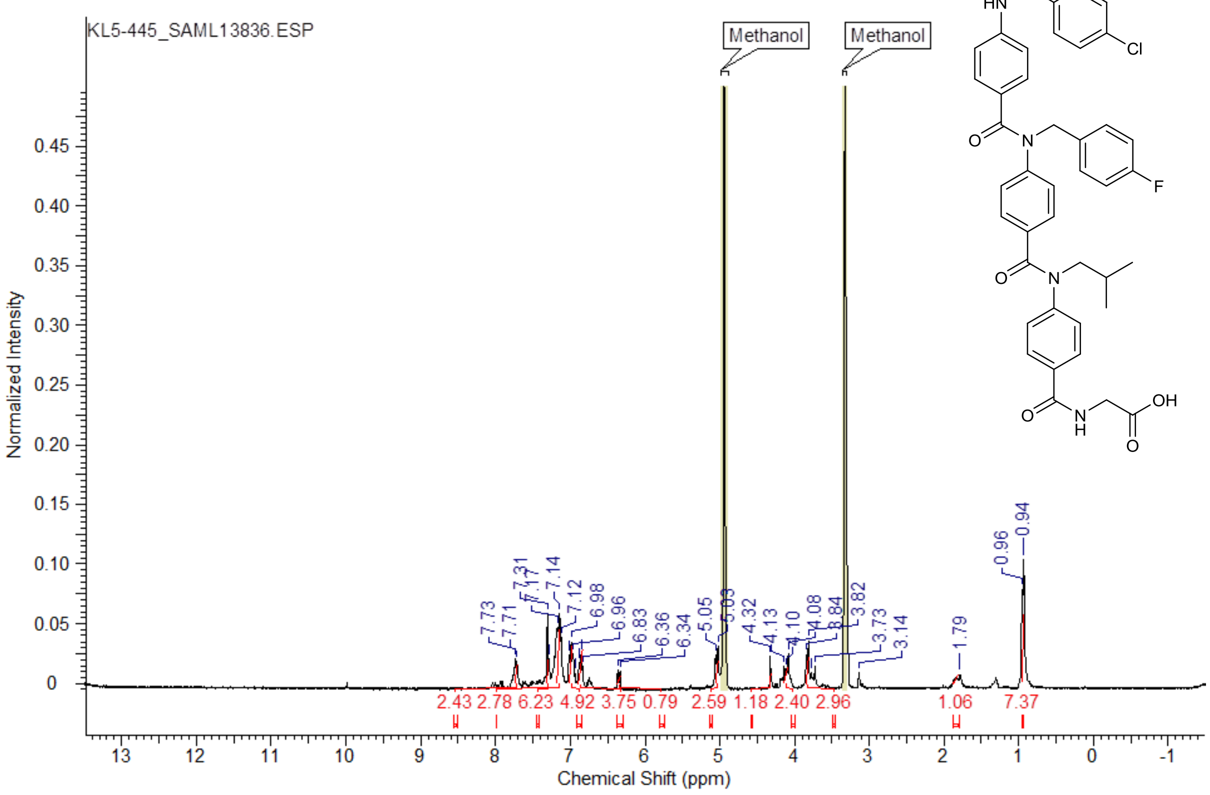

^{1}H NMR of Compound 53 in MeOD

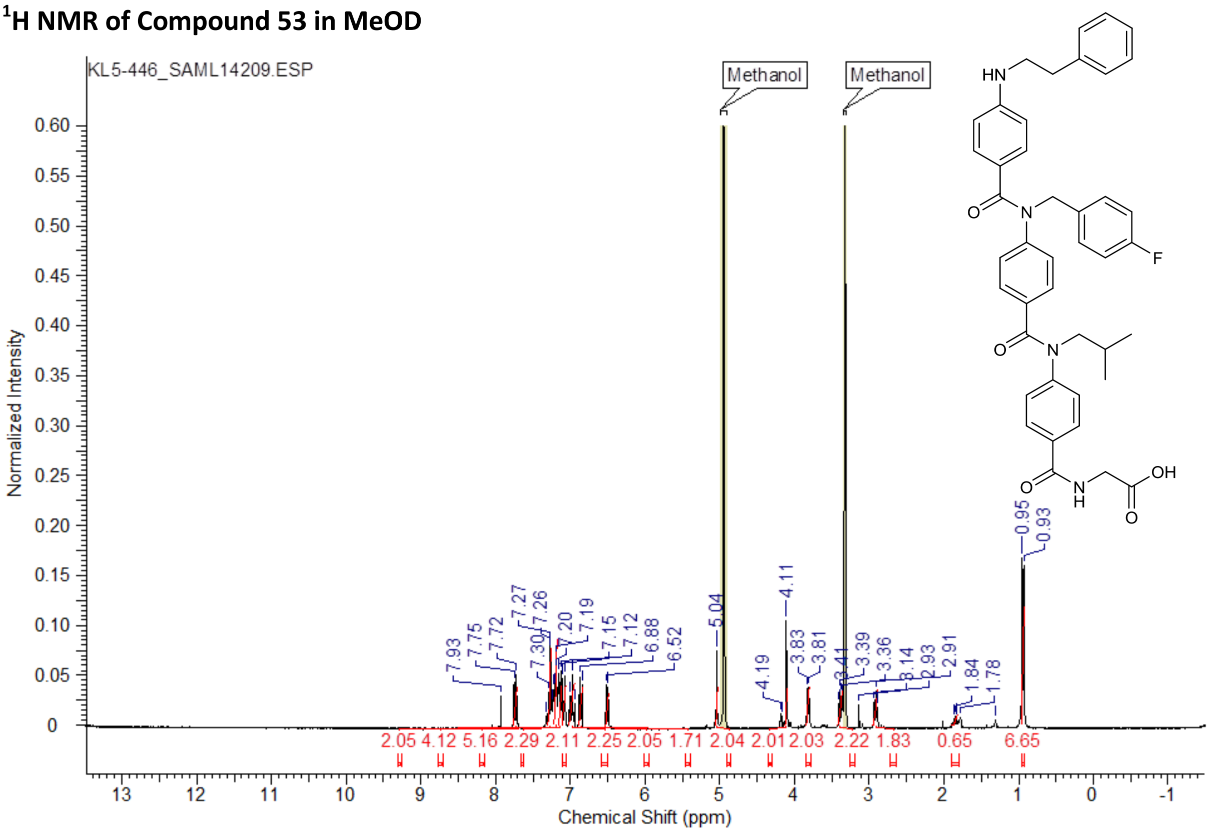

^{1}H NMR of Compound 54 in MeOD

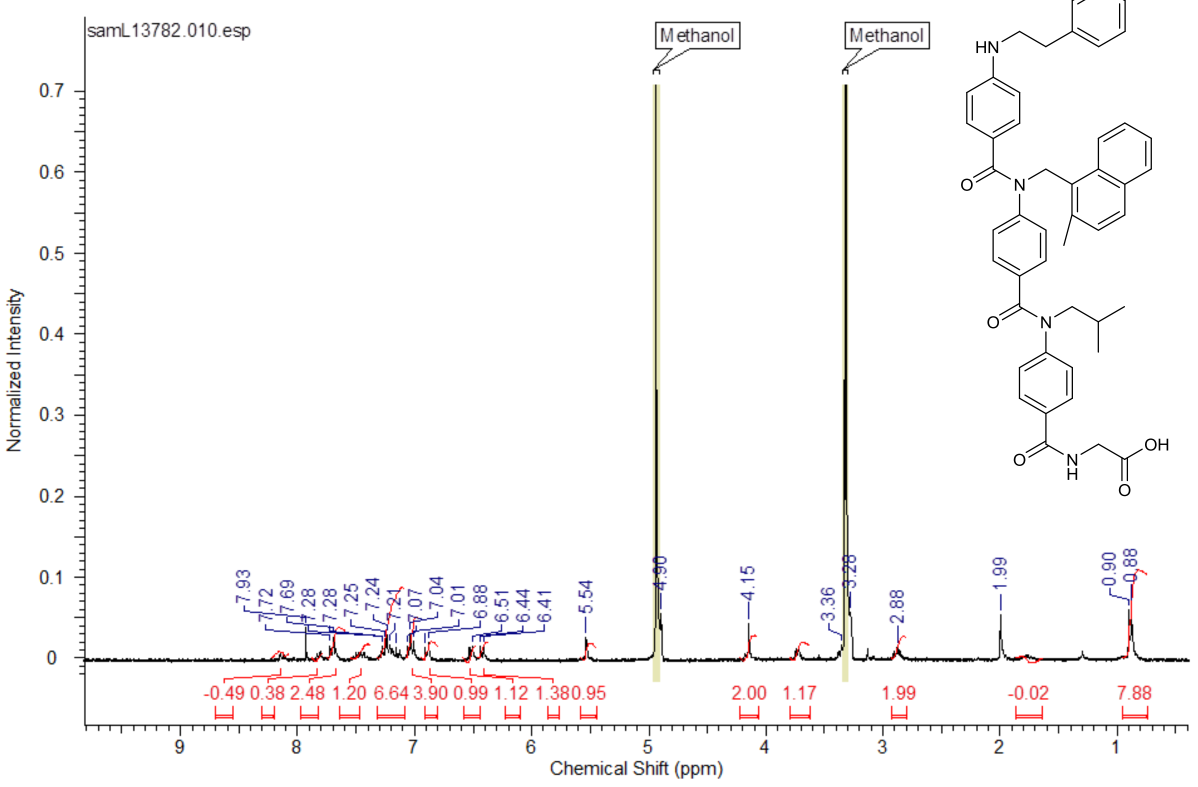

^{1}H NMR of Compound 55 in MeOD

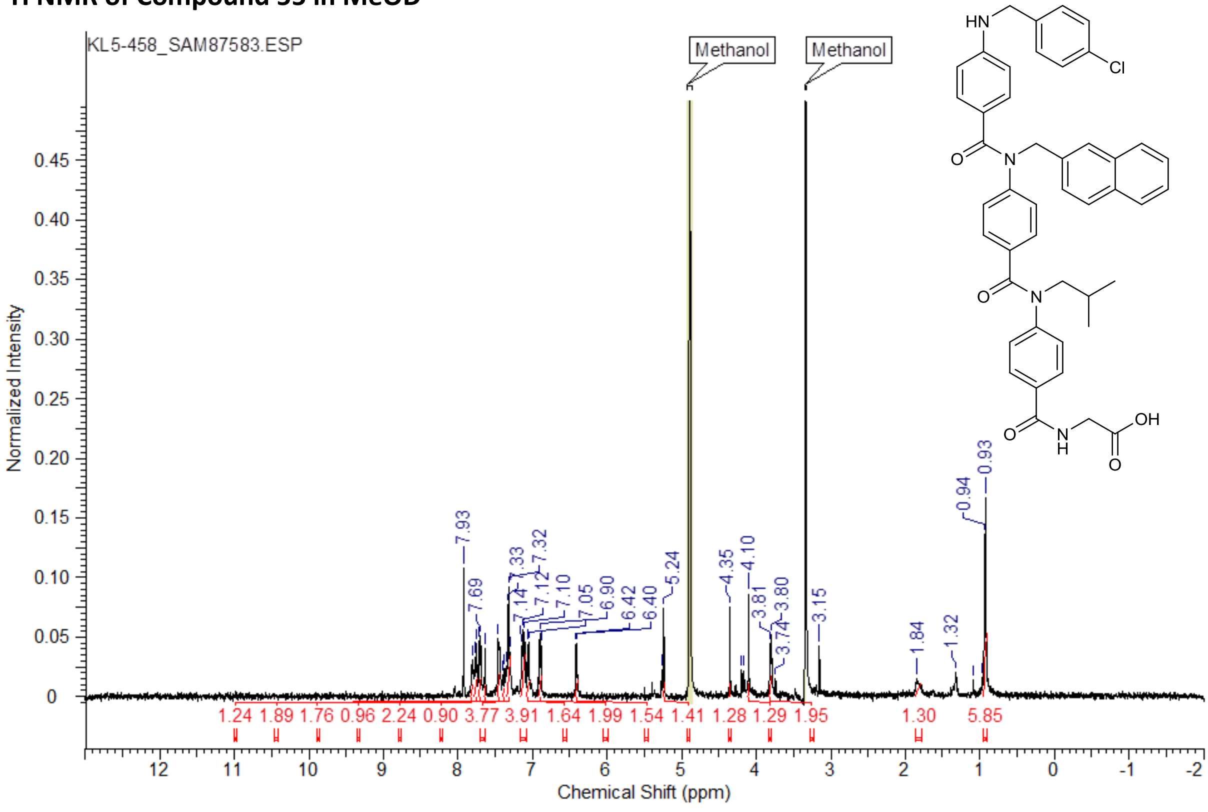

^{1}H NMR of Compound 58 in $CDCl_3$

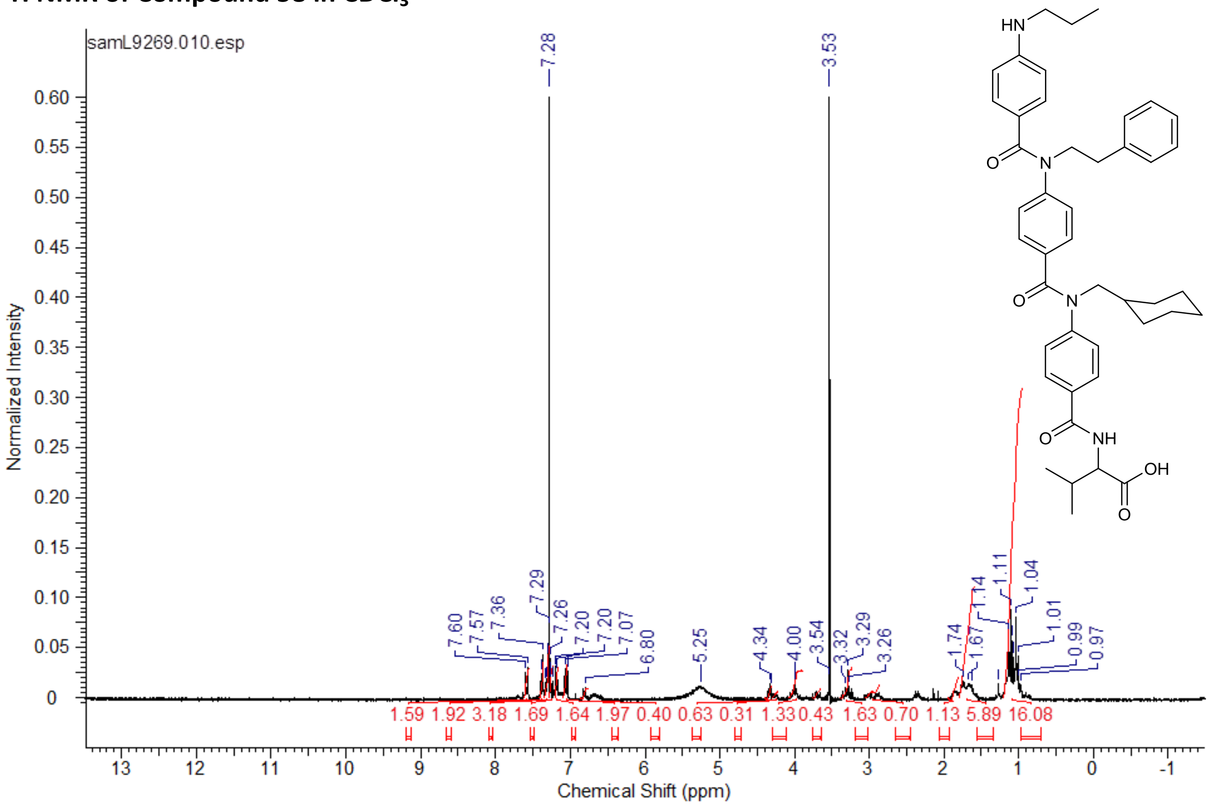

^{1}H NMR of Compound 59 in $CDCl_3$

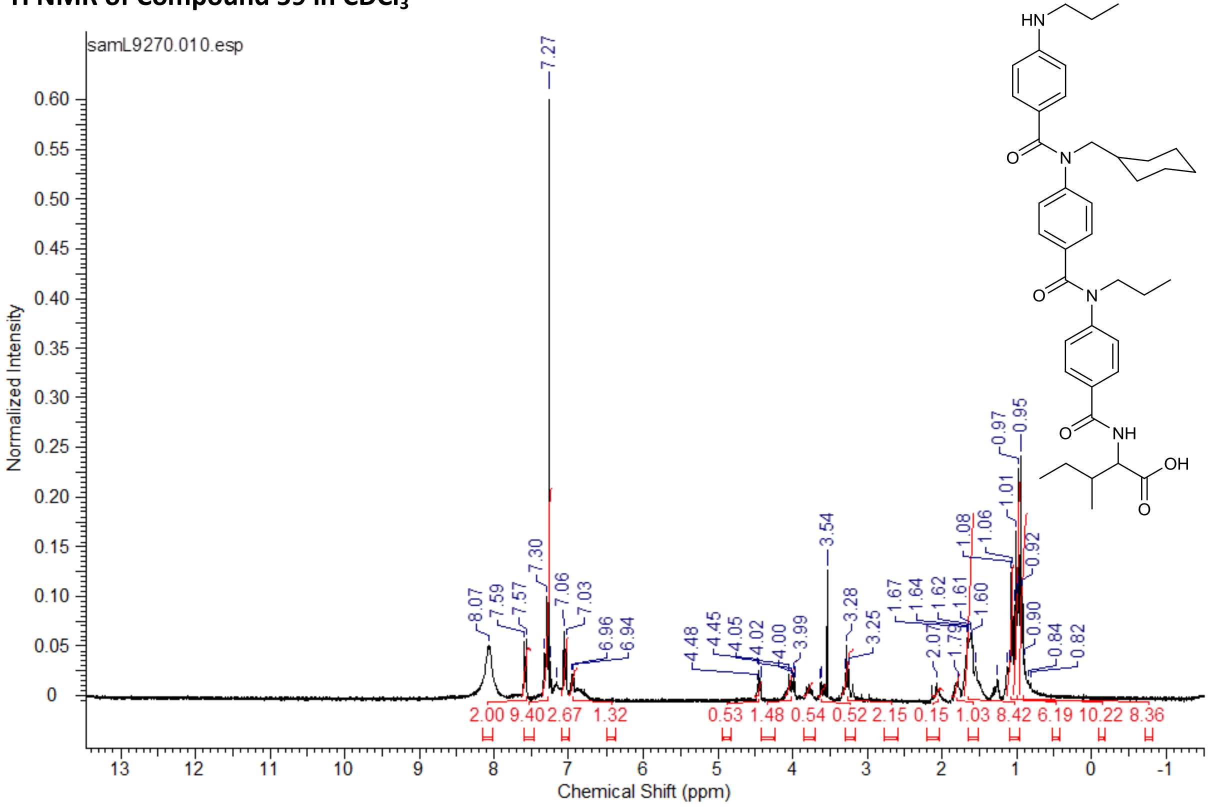

^{1}H NMR of Compound 60 in MeOD

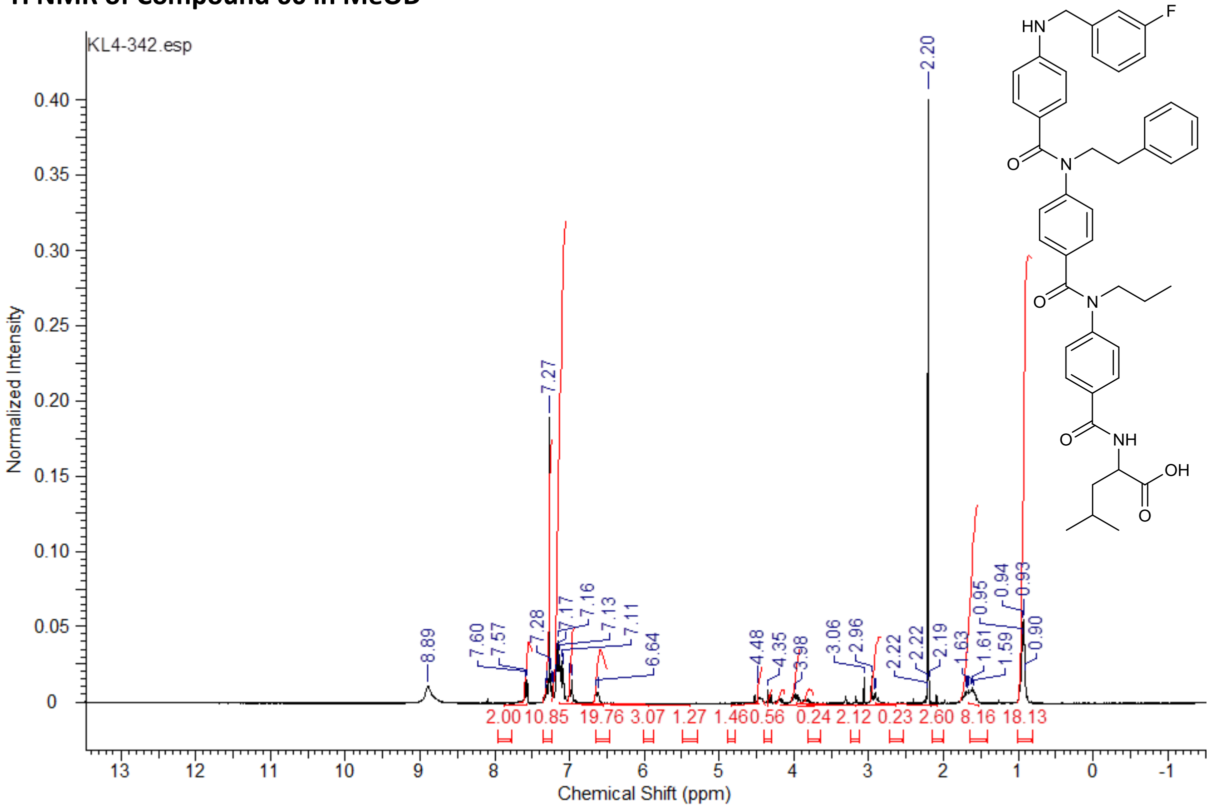

^{1}H NMR of Compound 61 in MeOD

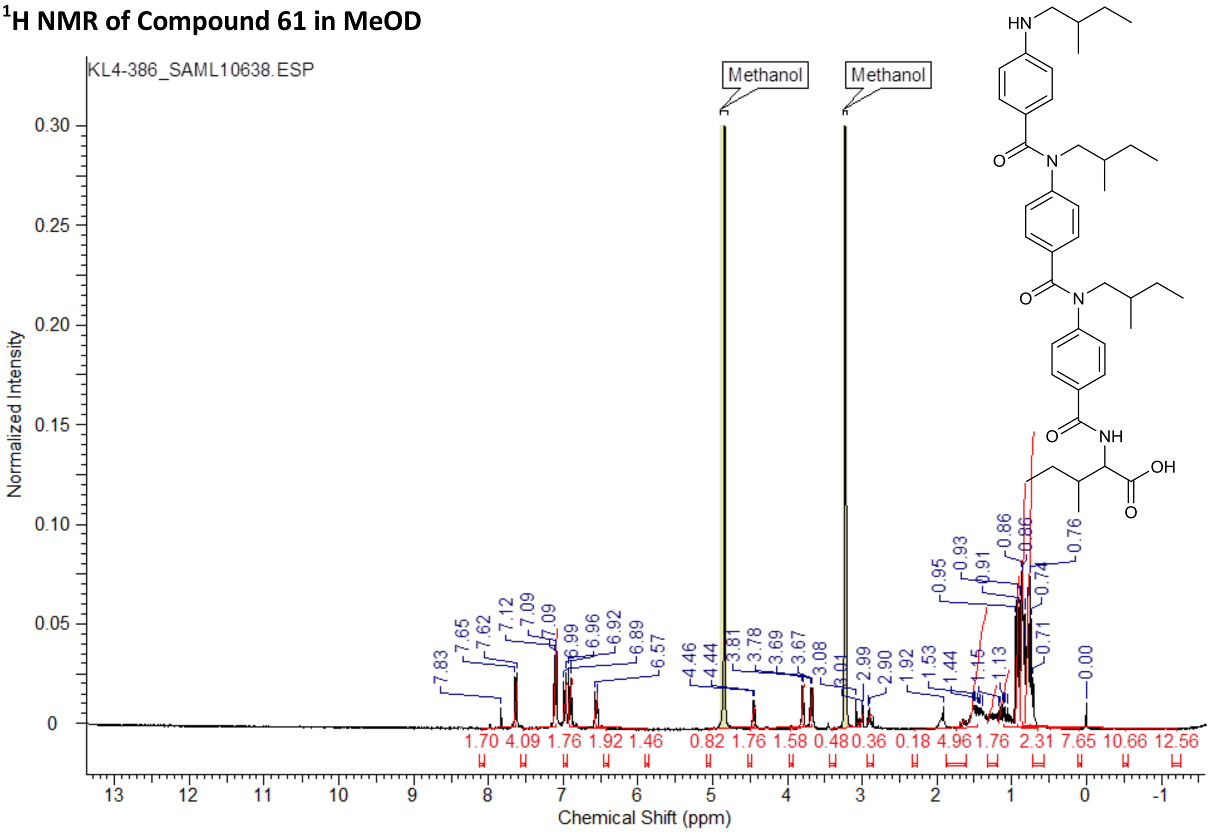

^{1}H NMR of Compound 62 in MeOD

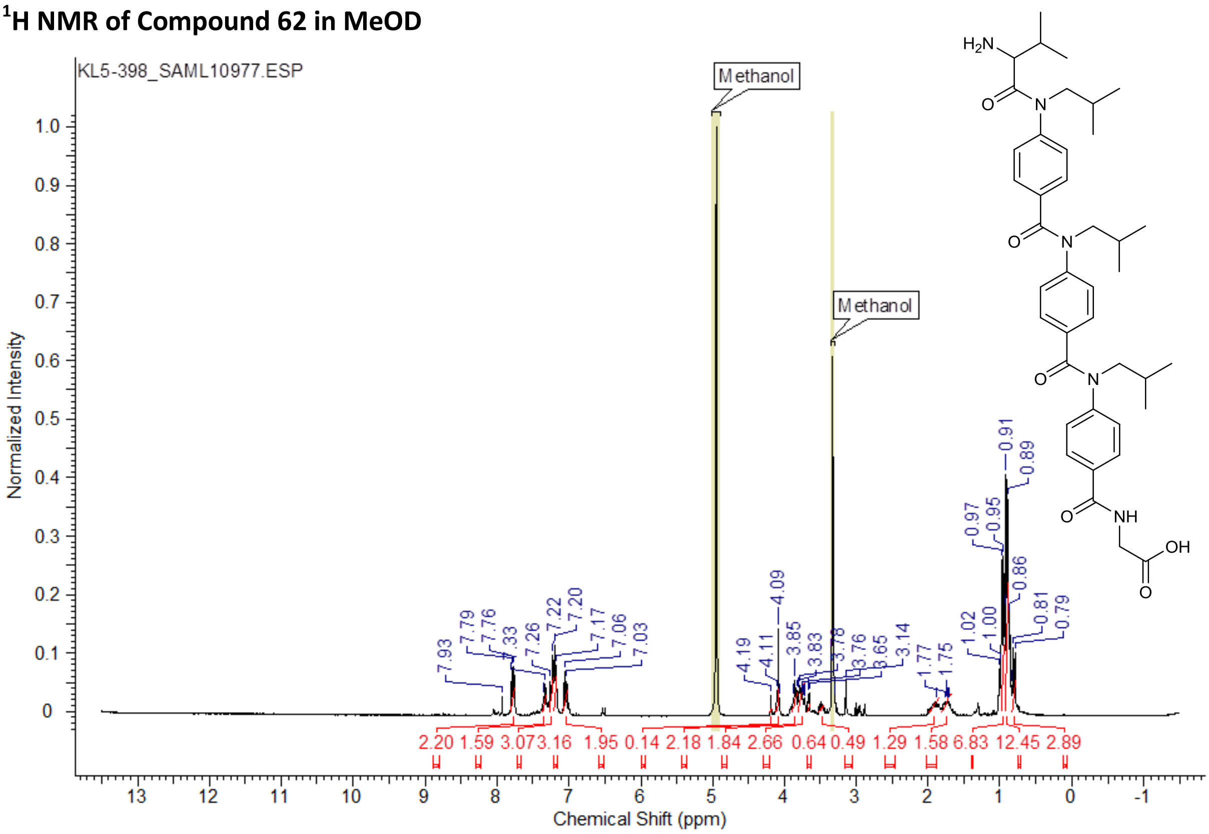

^{1}H NMR of Compound 63 in MeOD

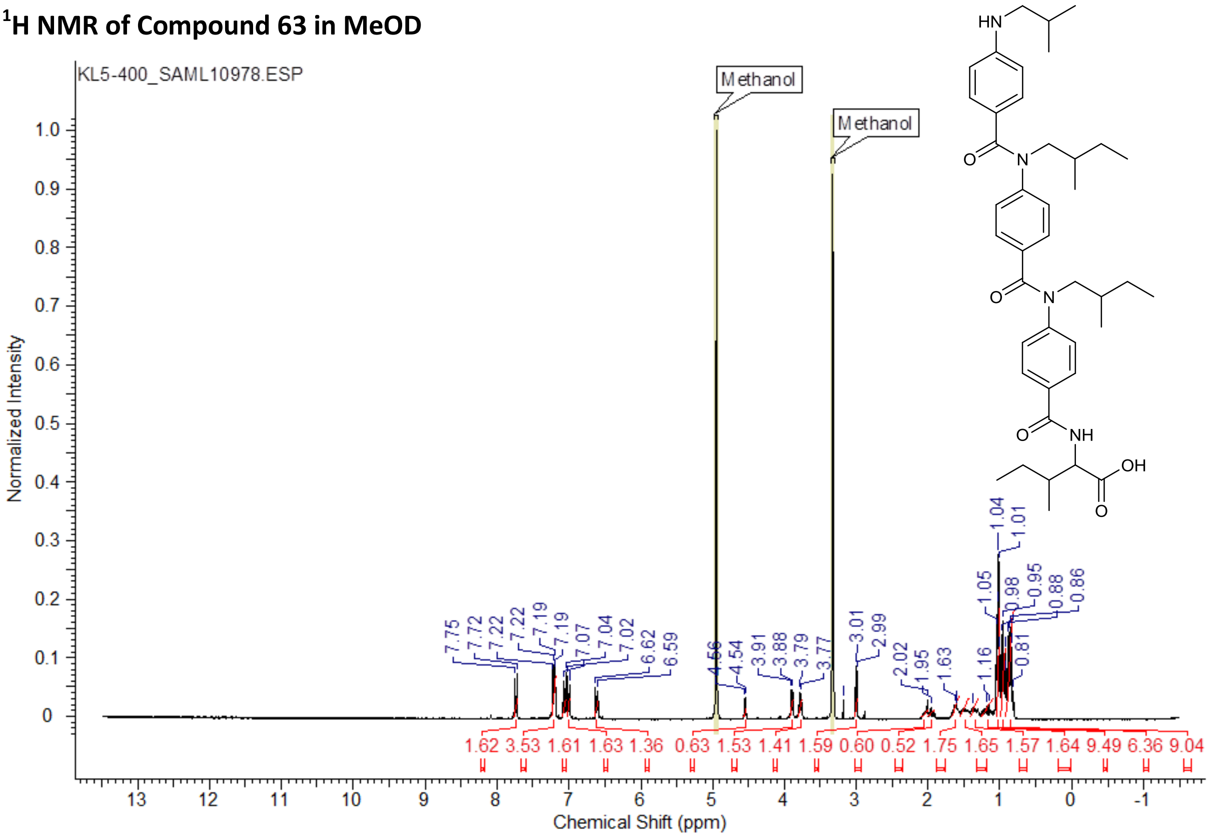

^{1}H NMR of Compound 64 in MeOD

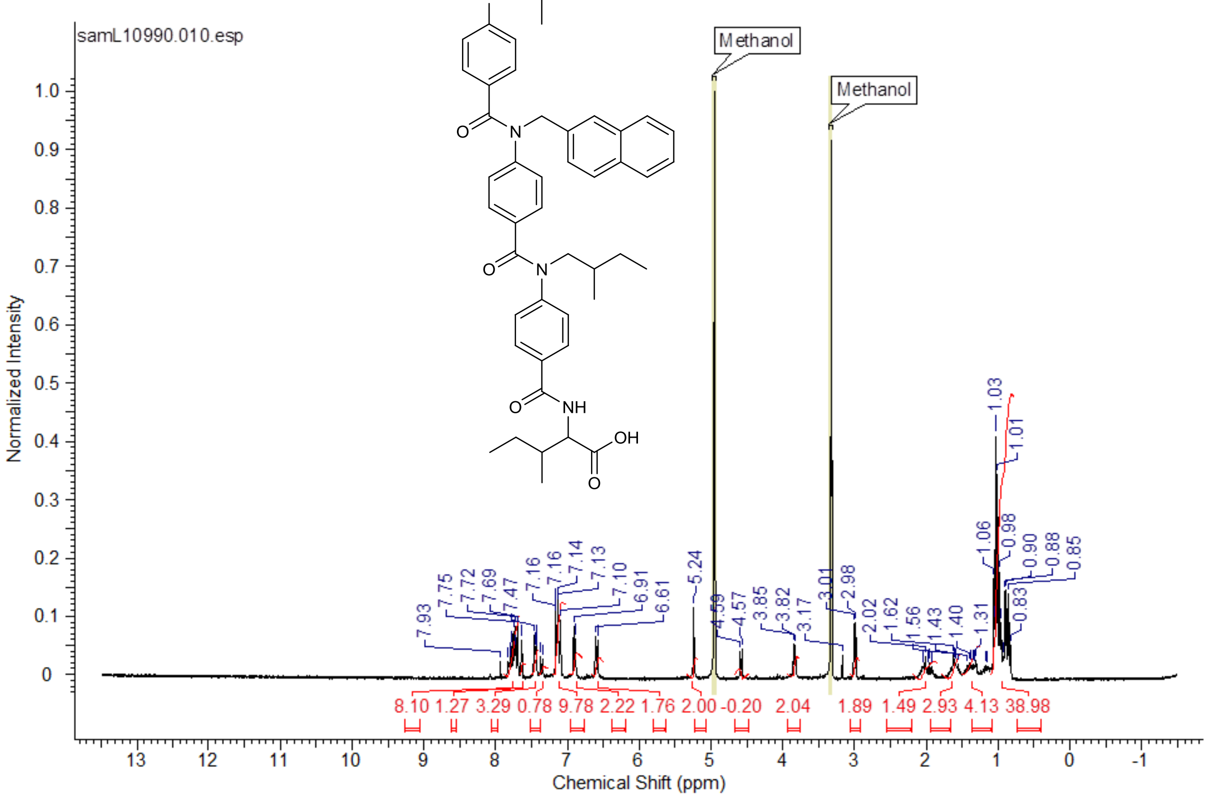

^{1}H NMR of Compound 65 in MeOD

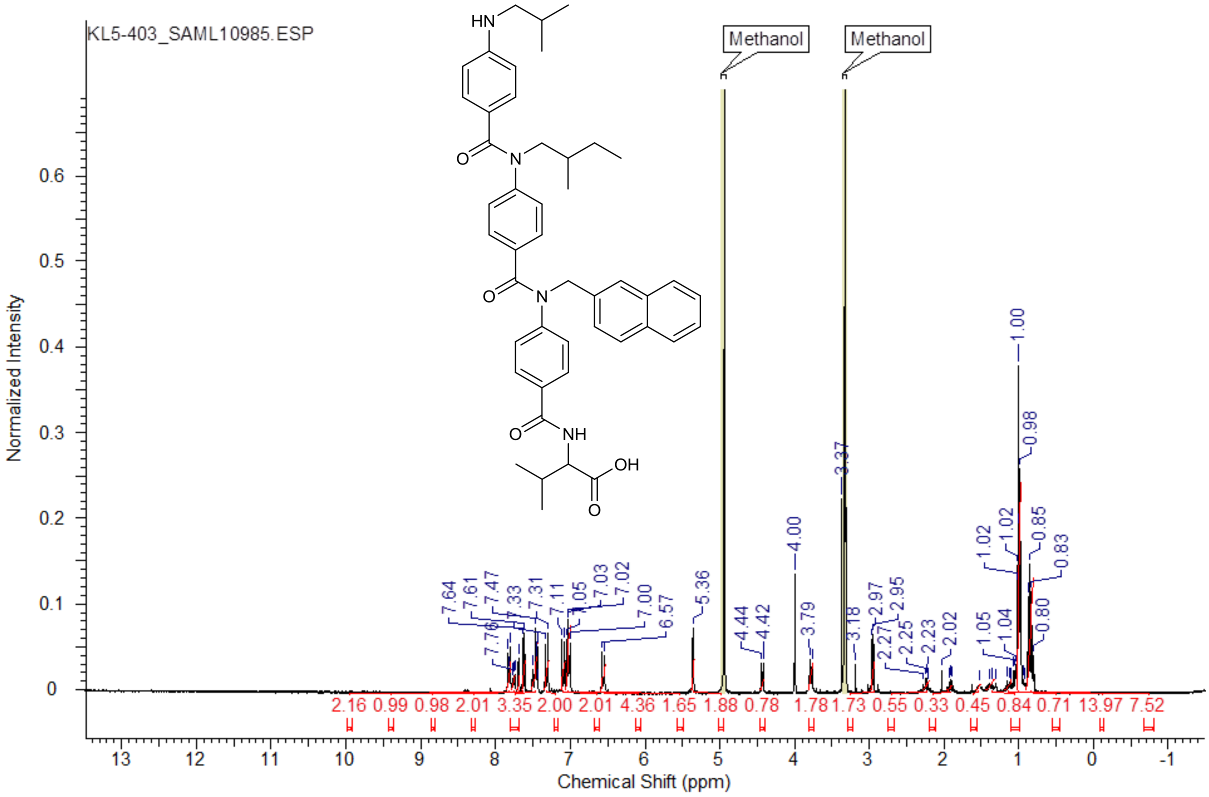

^{1}H NMR of Compound 66 in MeOD

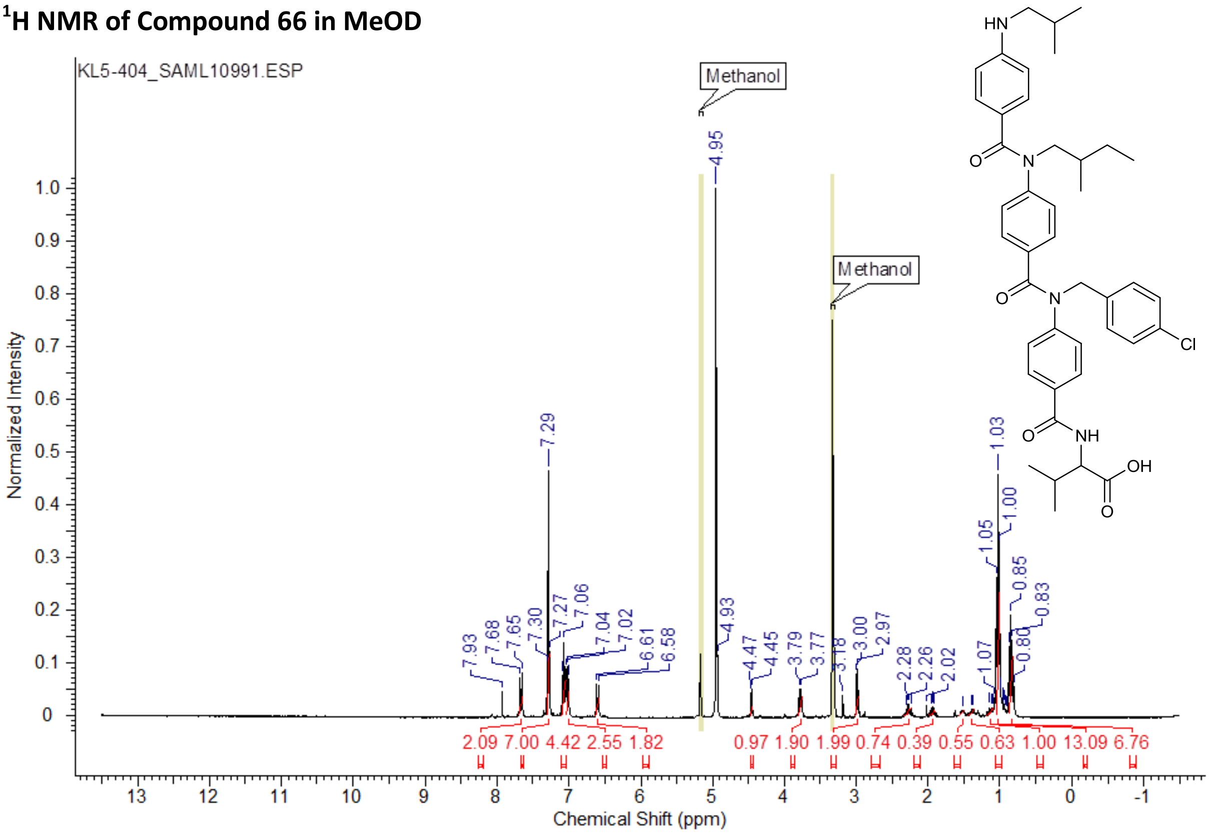

^{1}H NMR of Compound 67 in MeOD

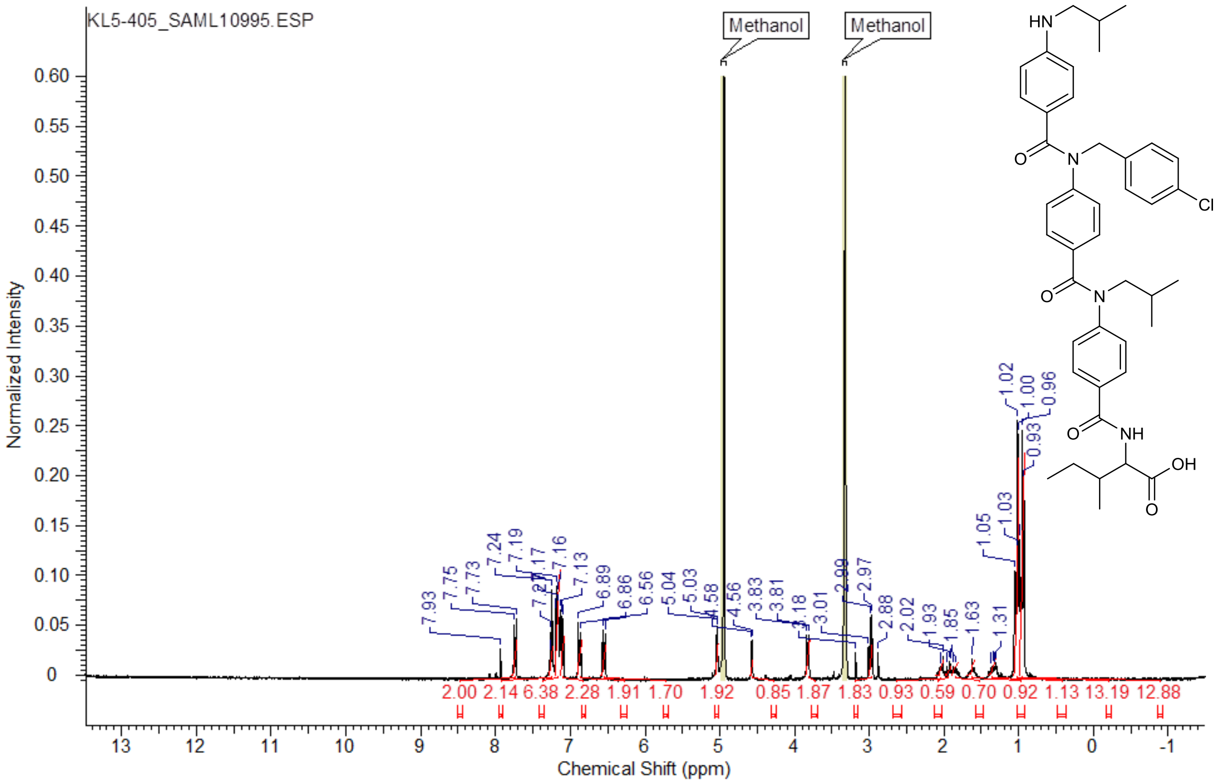

^{1}H NMR of Compound 68 in MeOD

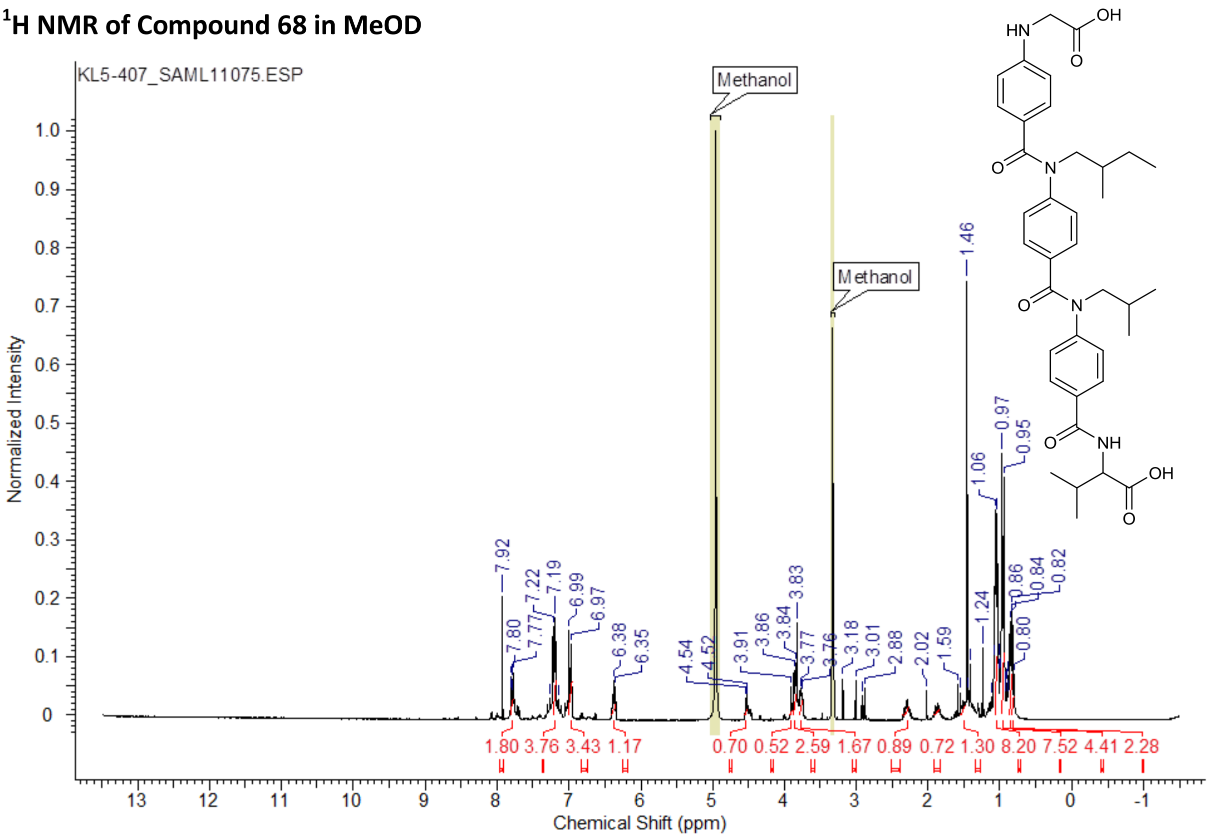

^{1}H NMR of Compound 69 in MeOD

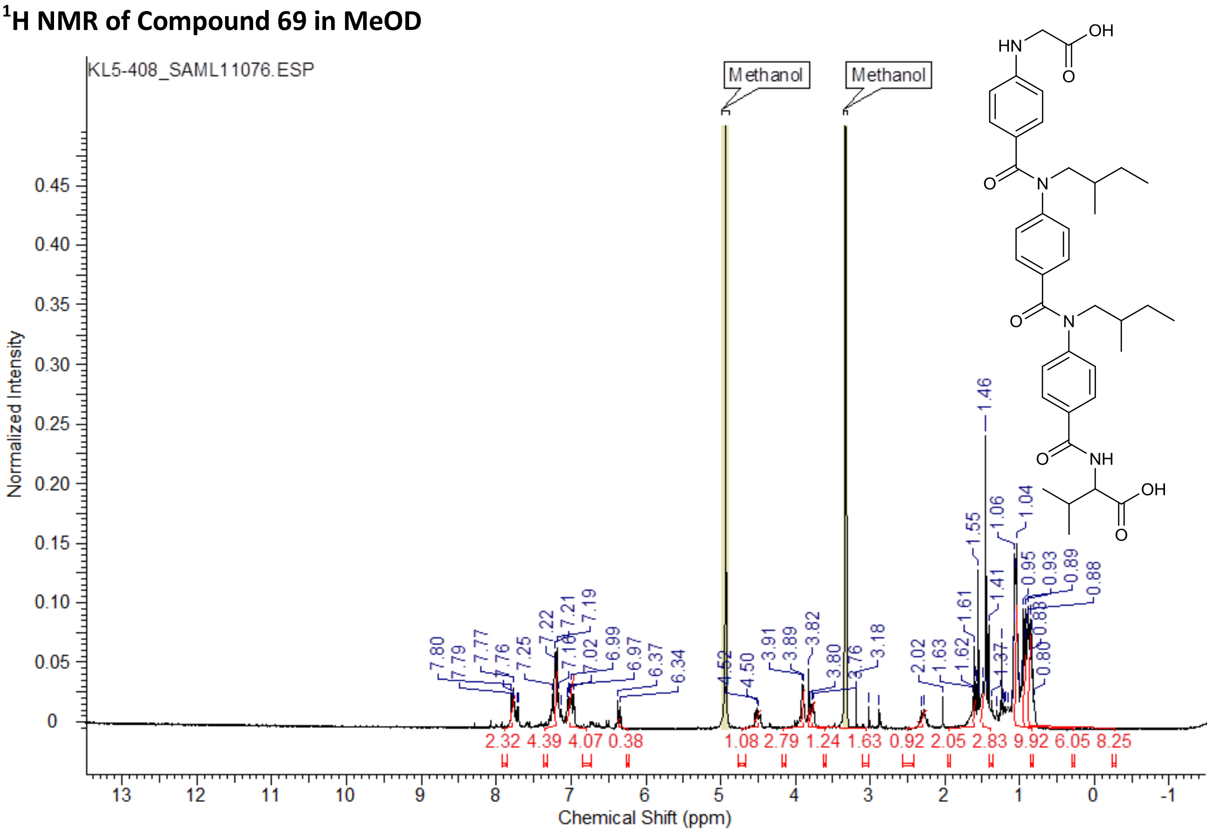

^{1}H NMR of Compound 70 in MeOD

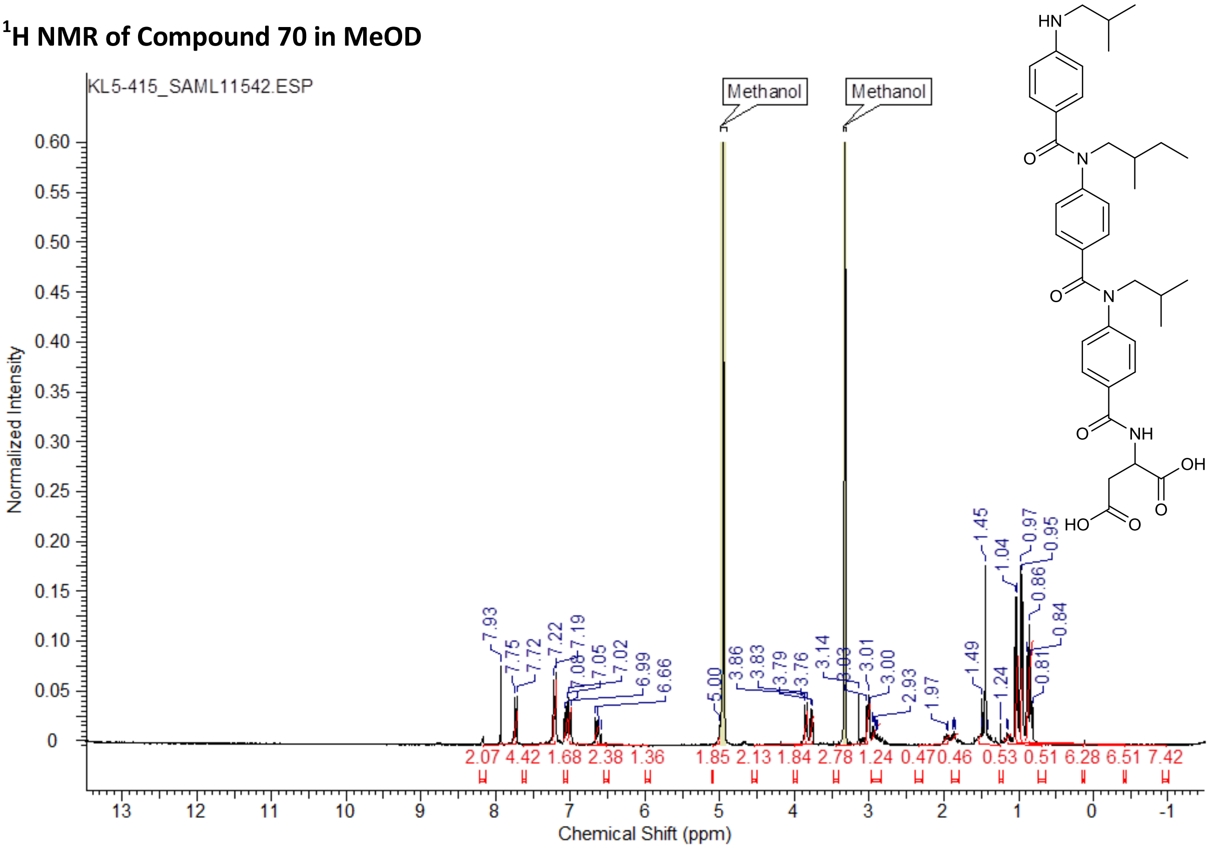

^{1}H NMR of Compound 71 in MeOD

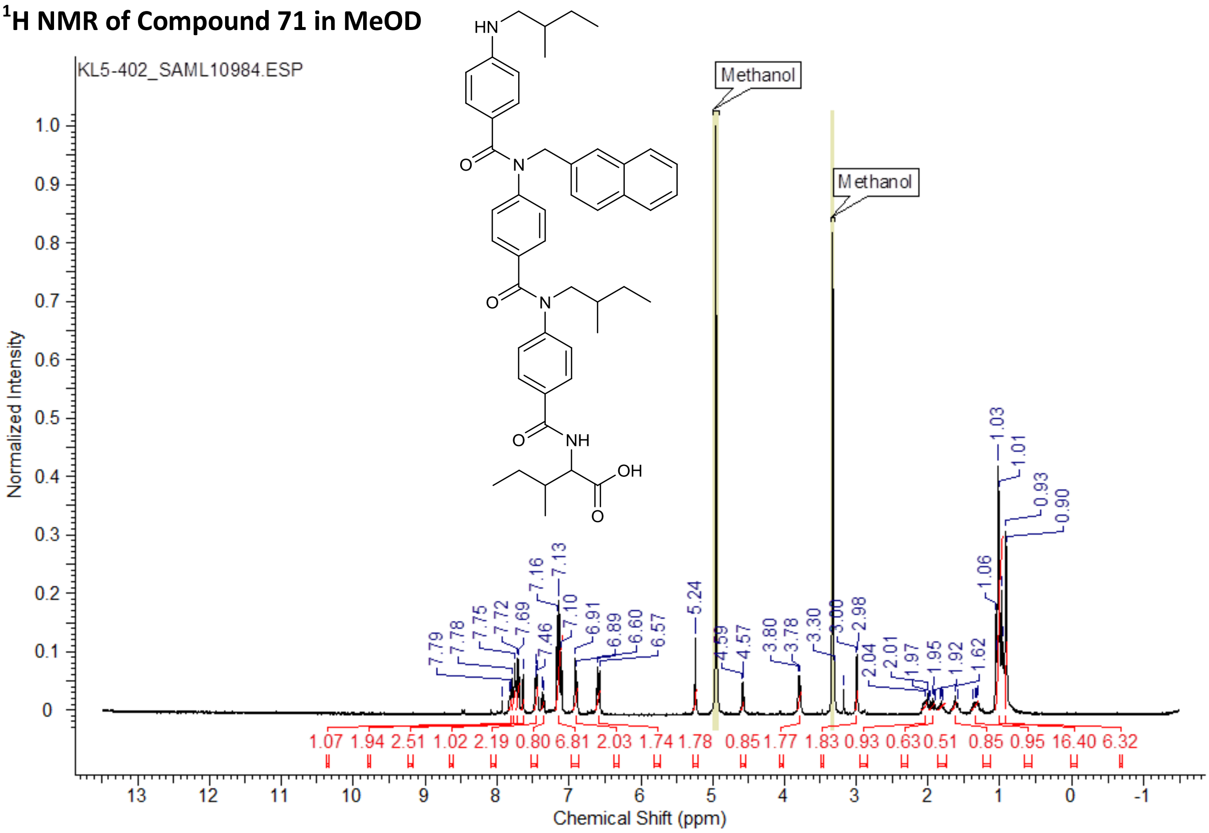

^{1}H NMR of Compound 73 in MeOD

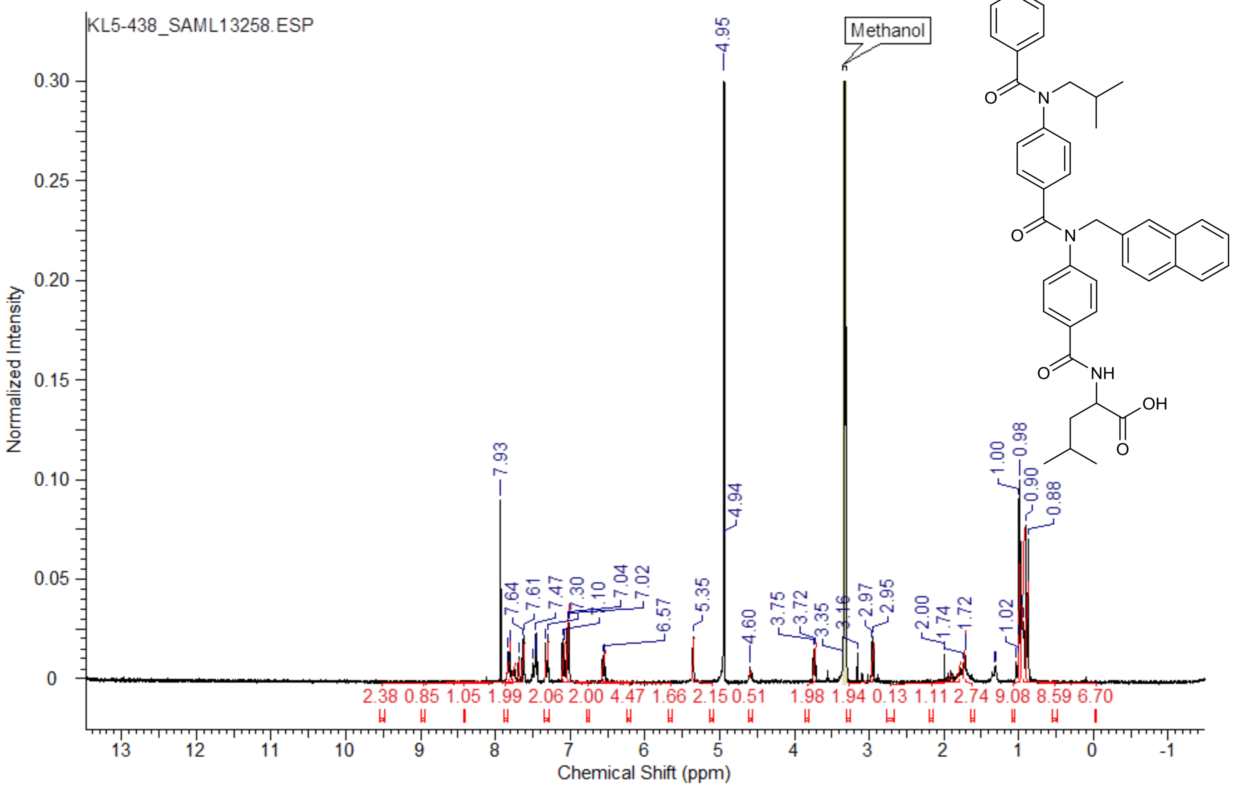

^{1}H NMR of Compound 74 in DMSO-d_6

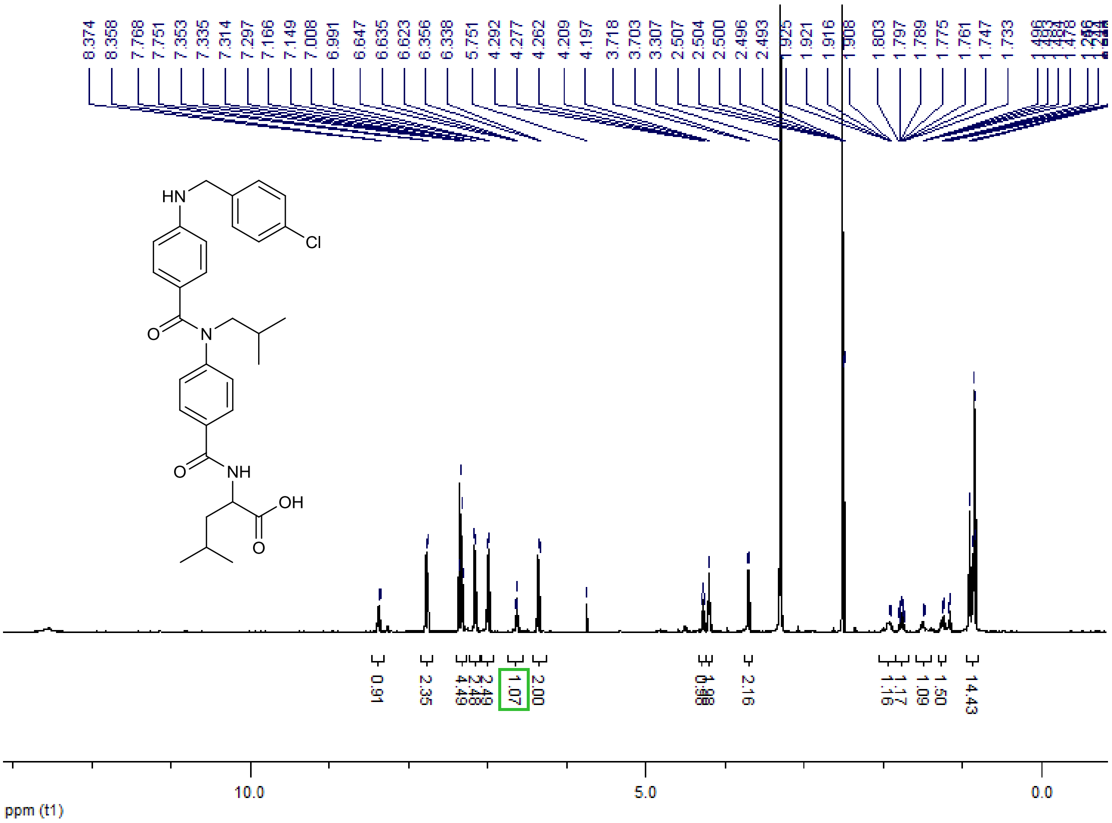

^{1}H NMR of Compound 75 in DMSO-d_6

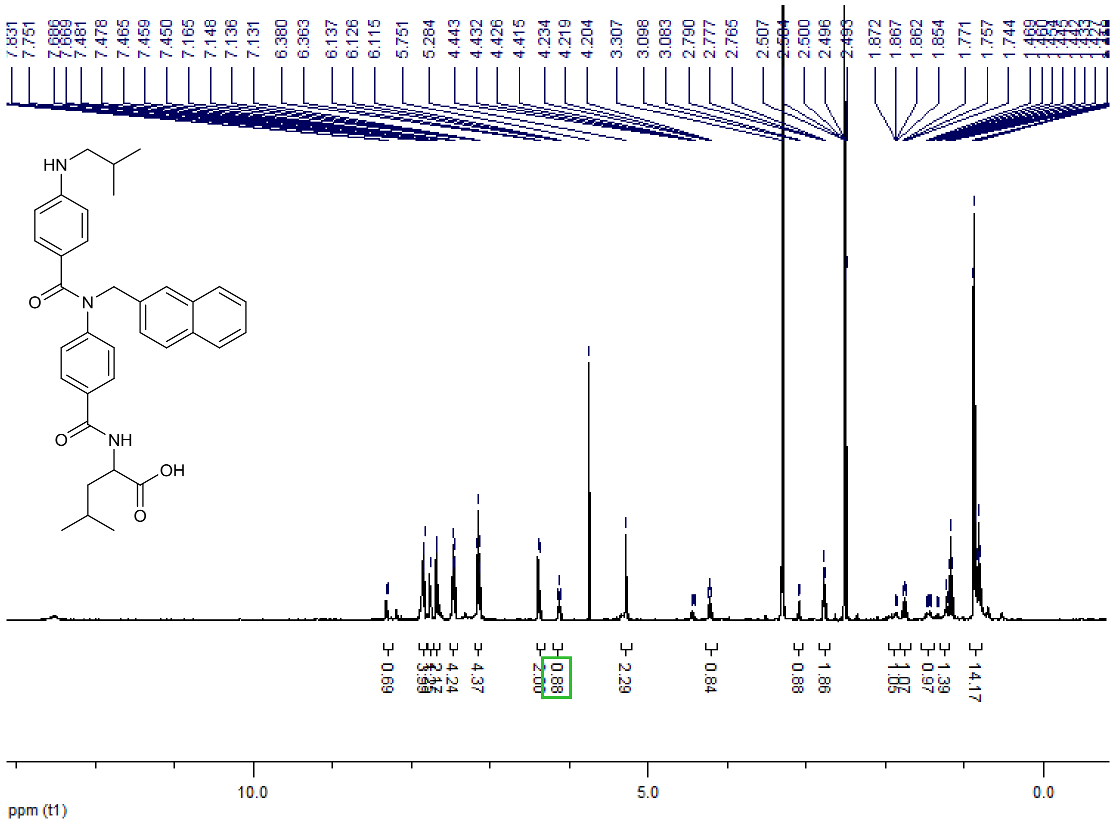

^{1}H NMR of Compound 76 in DMSO-d_6

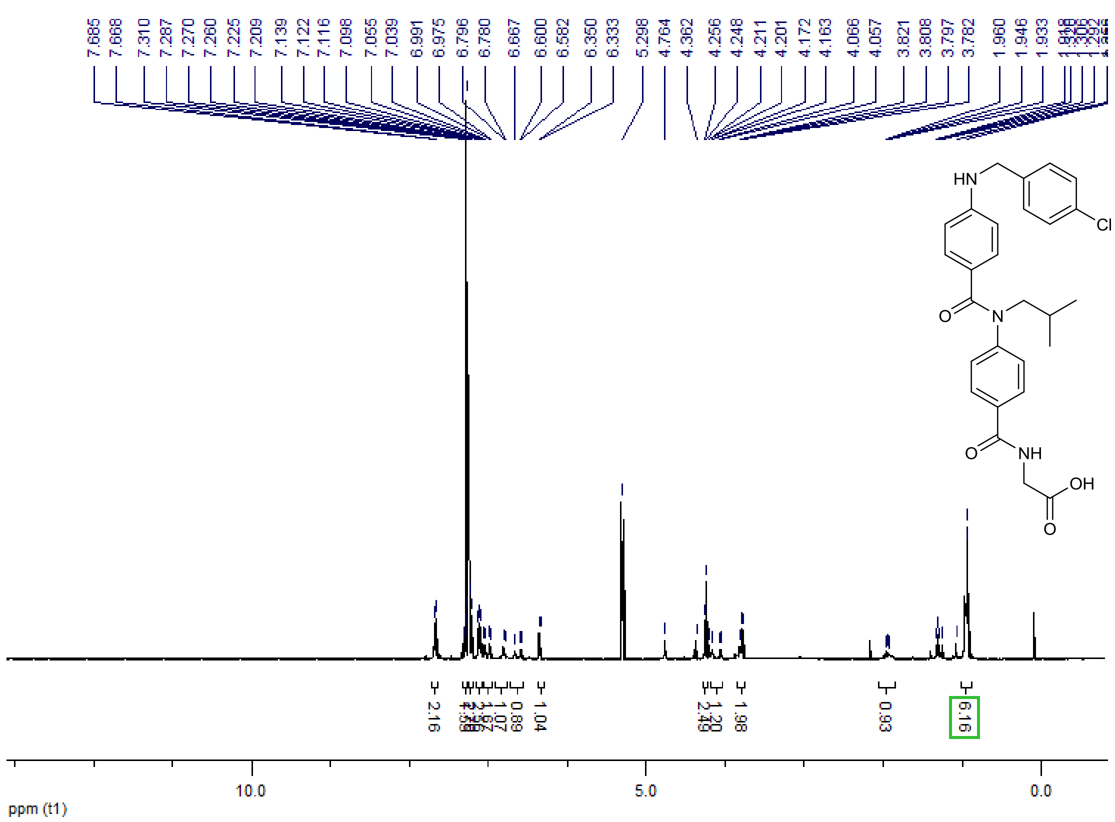

^{1}H NMR of Compound 77 in DMSO-d$_6$

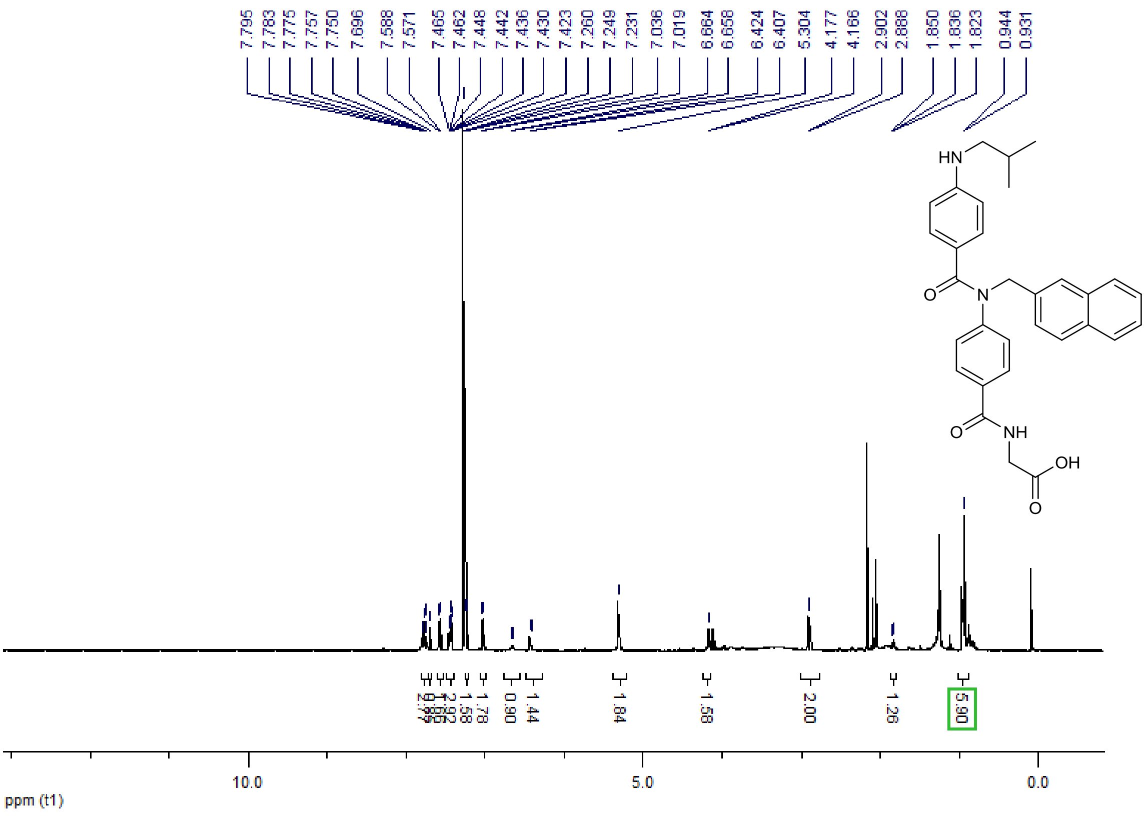

Modified Trimers

^{1}H NMR of 1-Biotin-3-azidopropylamine in MeOD

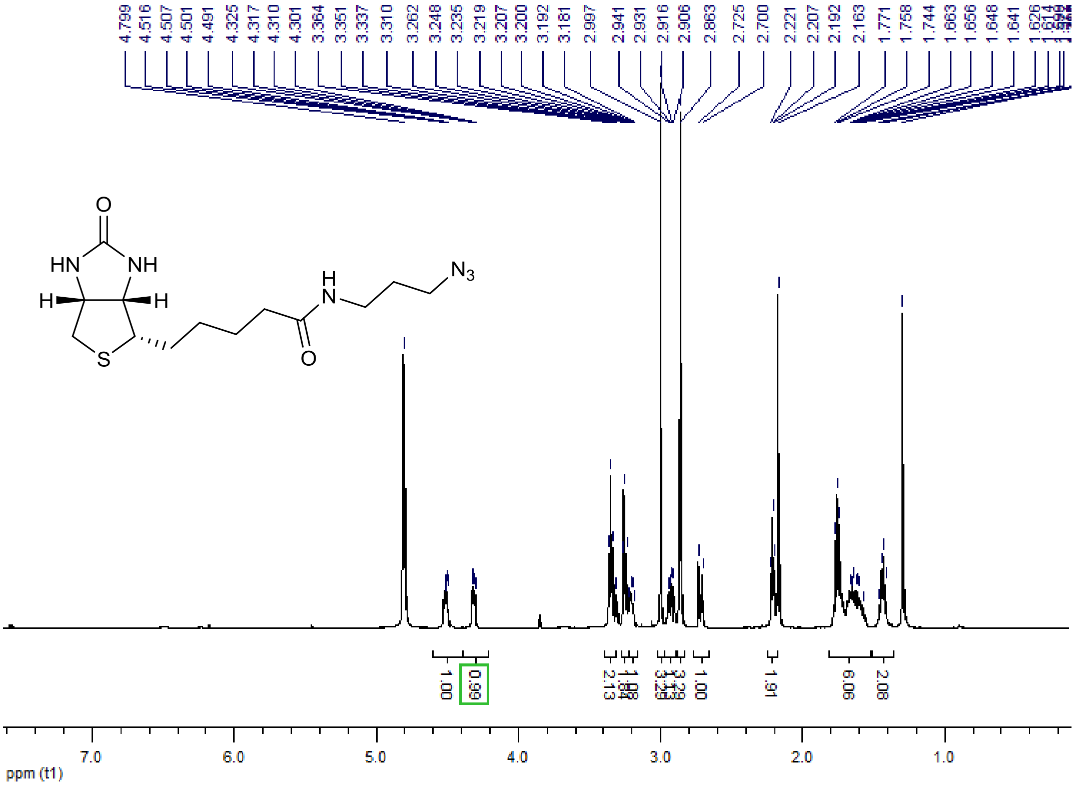

^{1}H NMR of Biotin-8 in DMSO-d_6

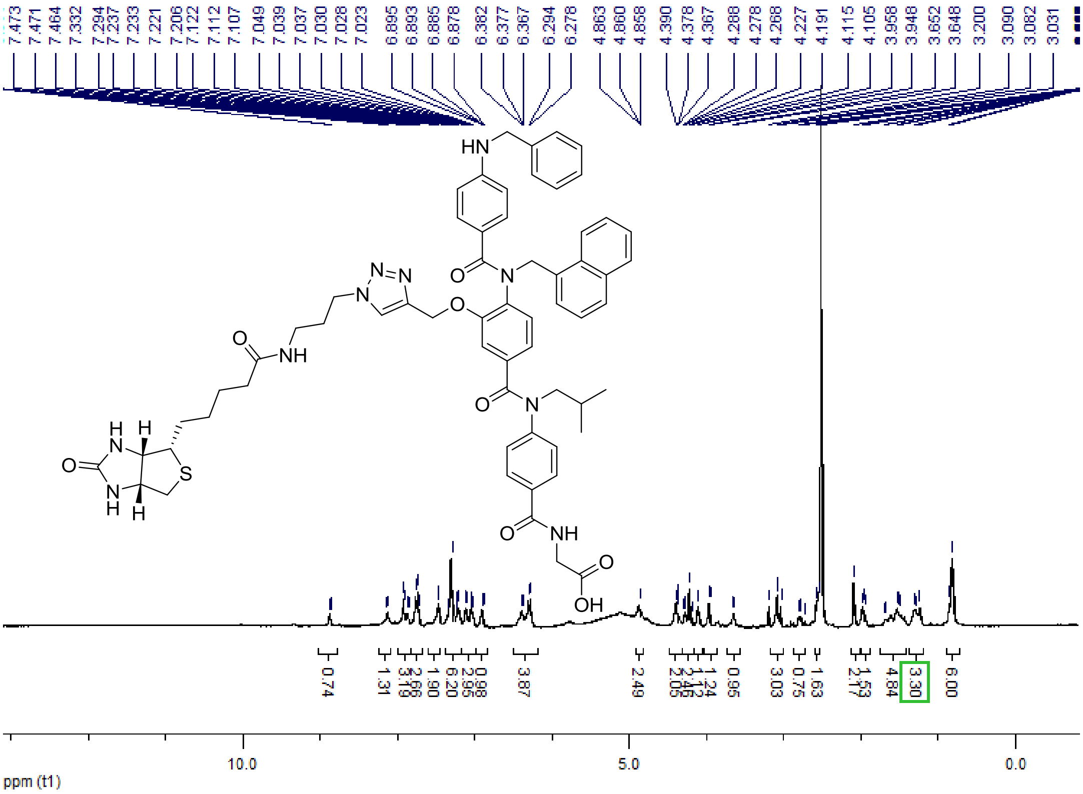

^{1}H NMR of Biotin-31 in DMSO-d_6

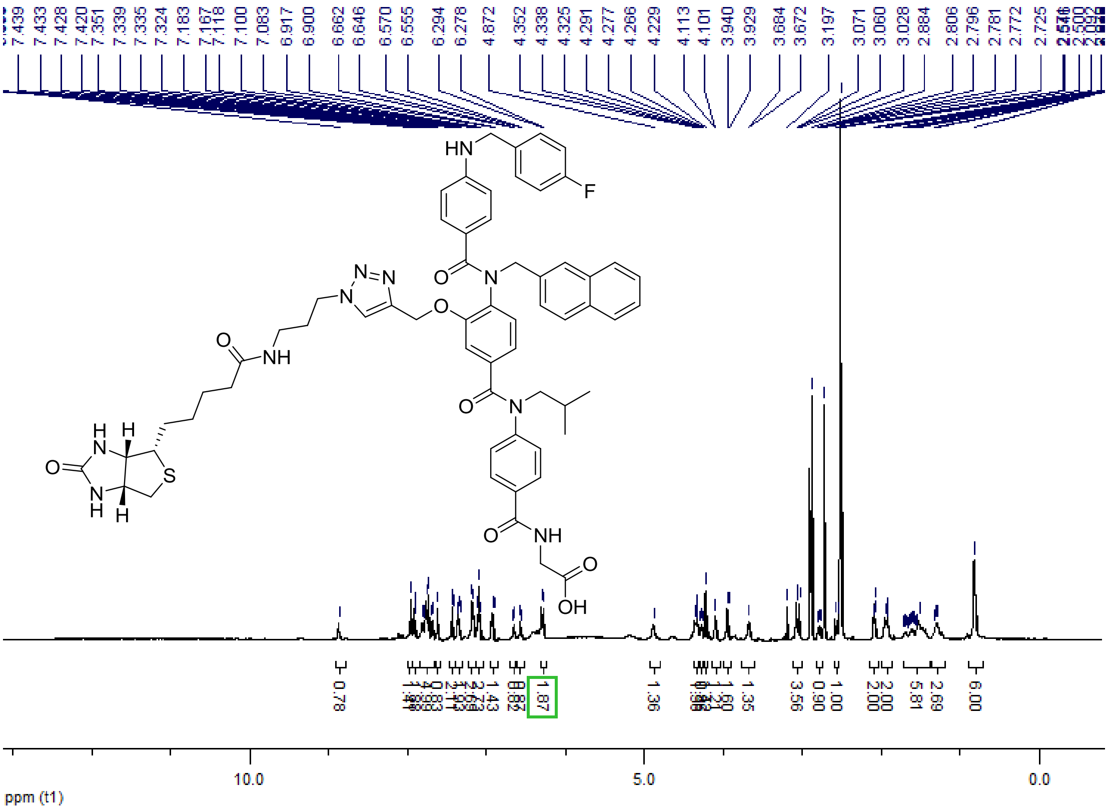

^{1}H NMR of Biotin-48 in DMSO-d_6

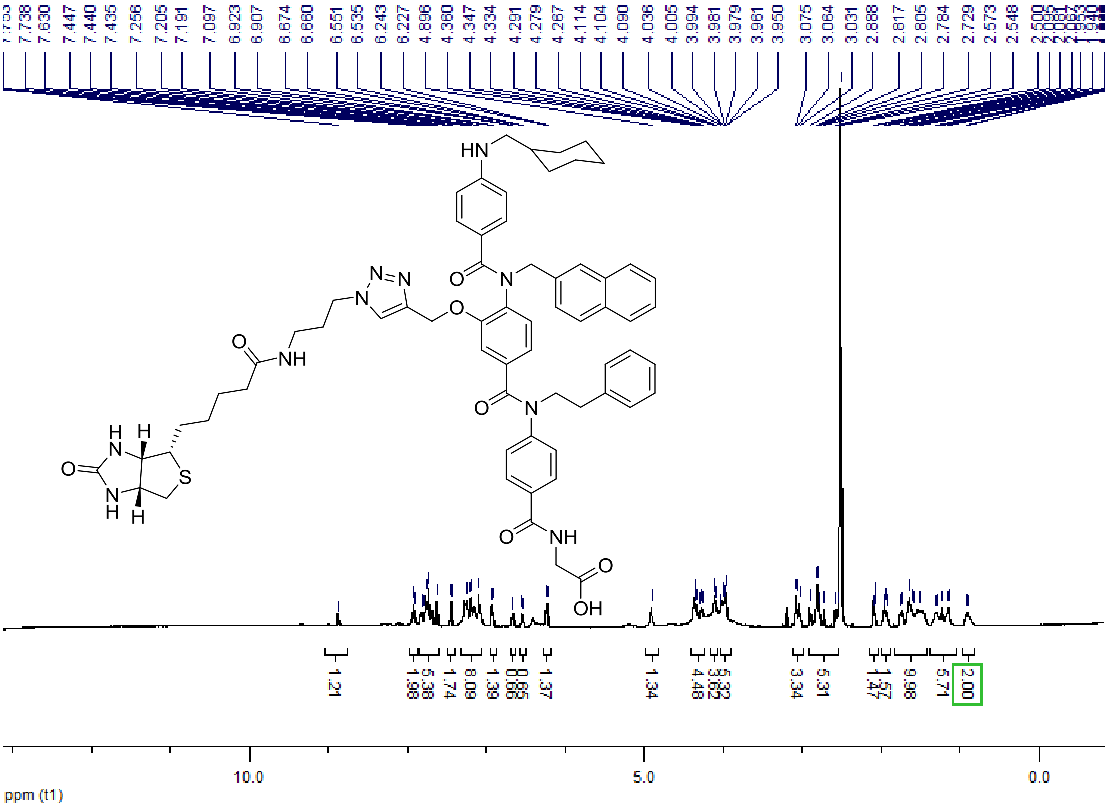

^{1}H NMR of Biotin-64 in DMSO-d_6

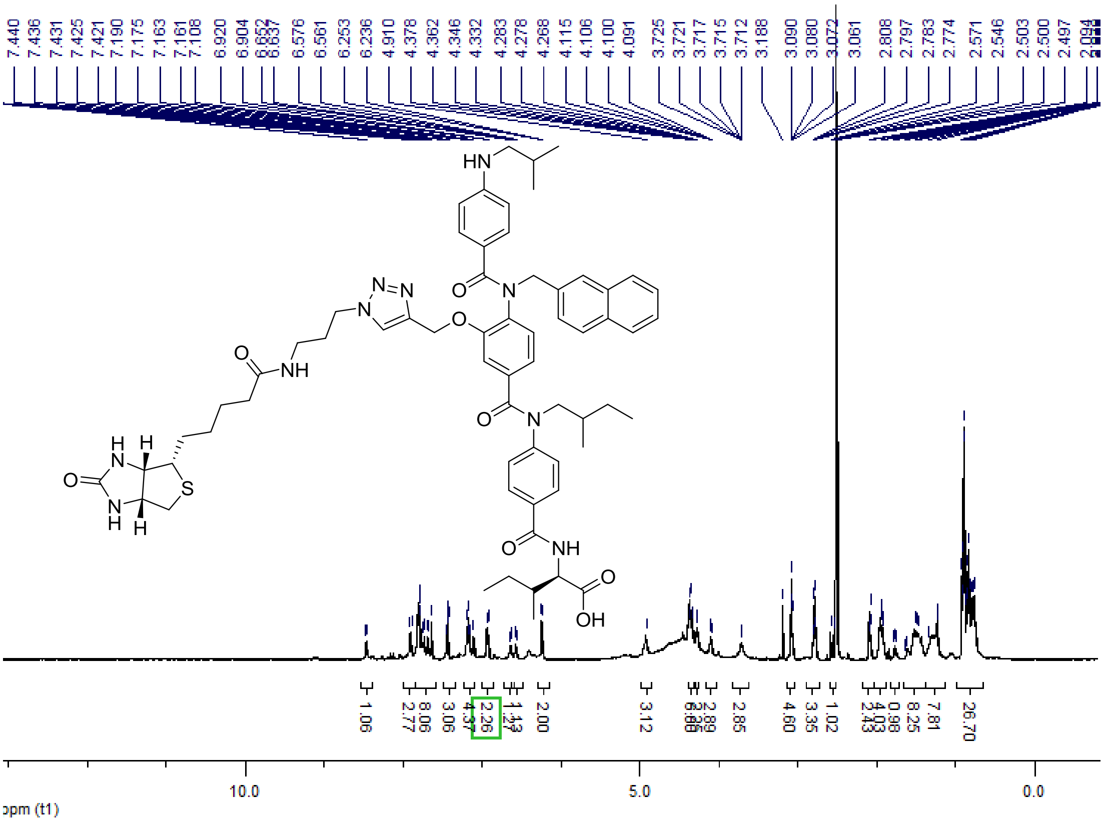

^{1}H NMR of Biotin-67 in DMSO-d$_6$

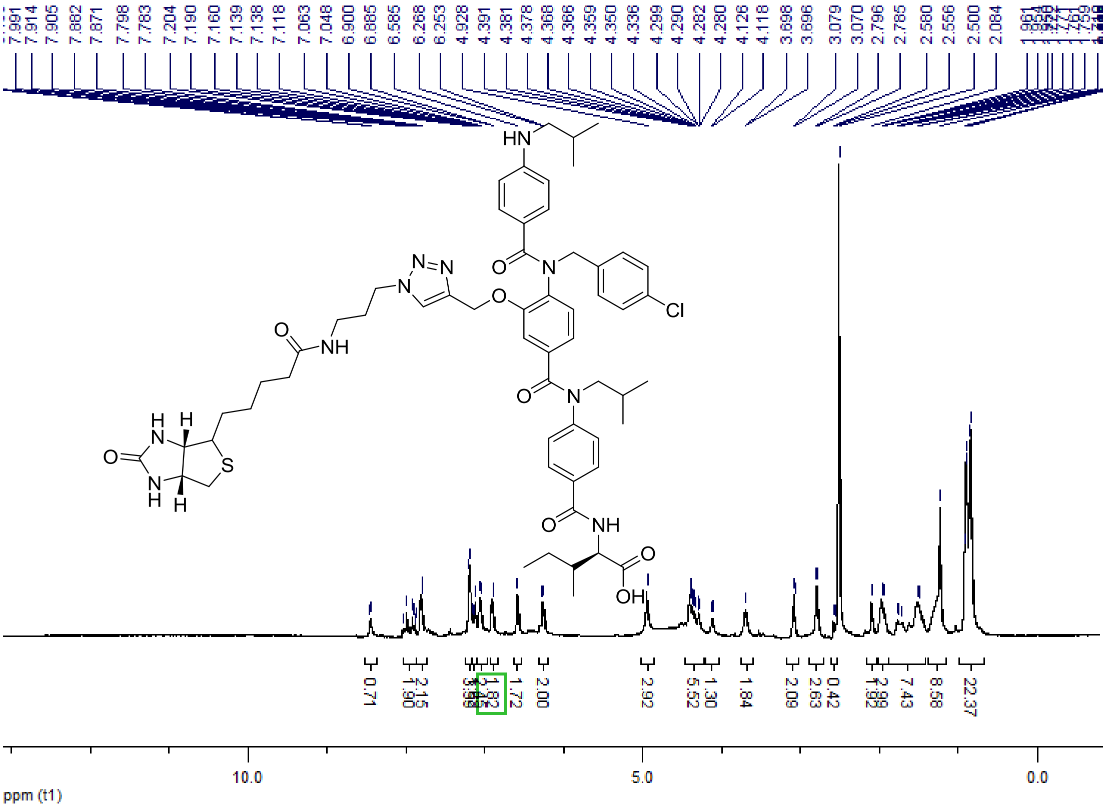

^{1}H NMR of FITC-31 in DMSO-d$_6$

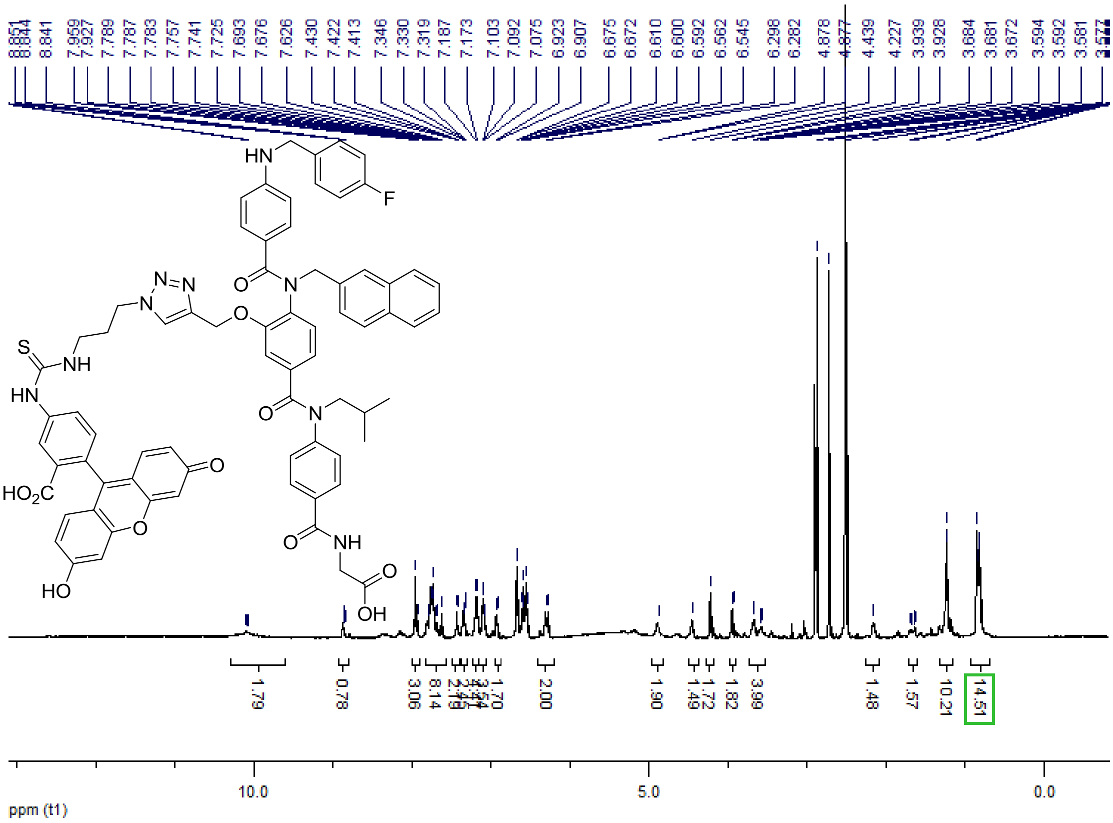

^{1}H NMR of FITC-48 in DMSO-d_6

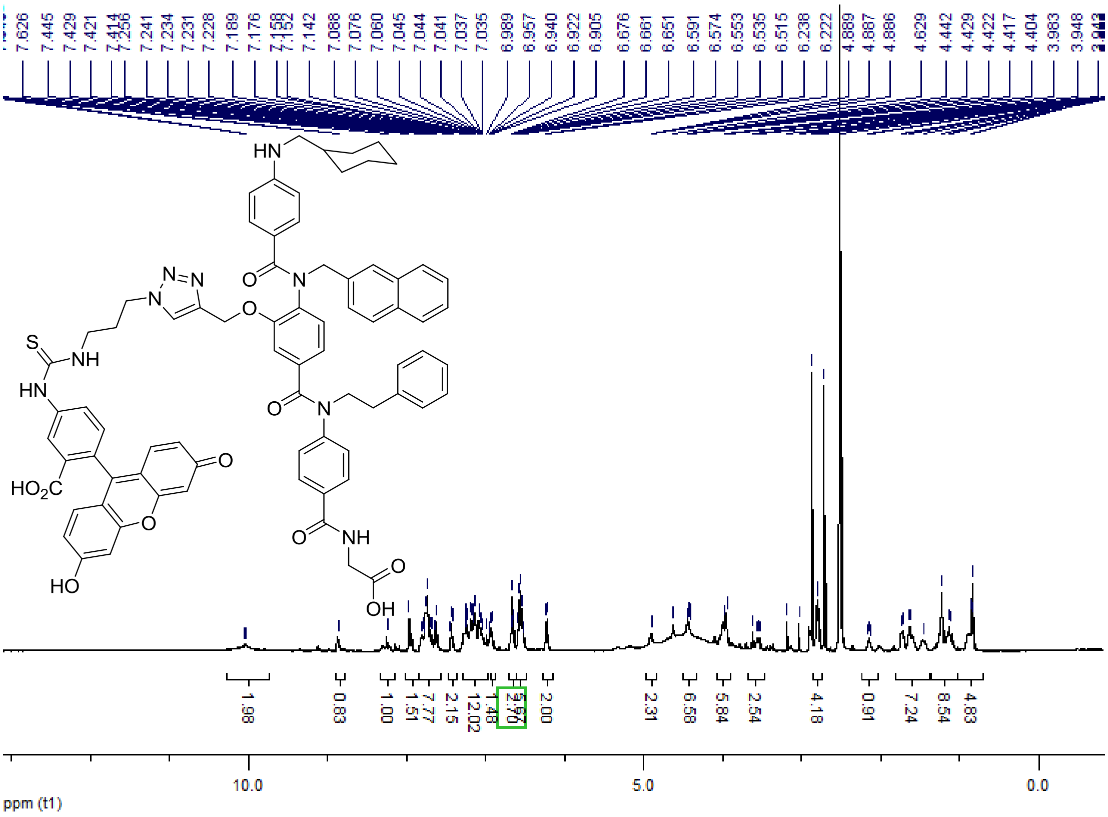

^{1}H NMR of FITC-64 in DMSO-d_6

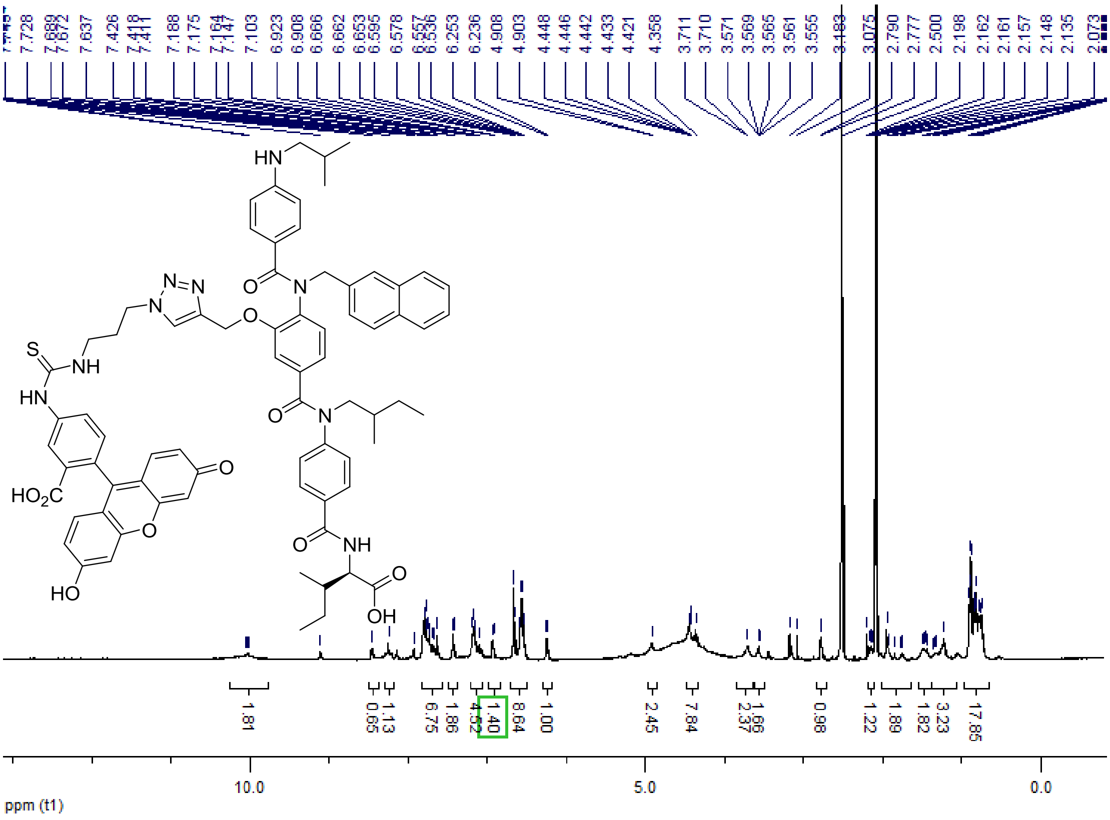

^{1}H NMR of FITC-67 in DMSO-d_6

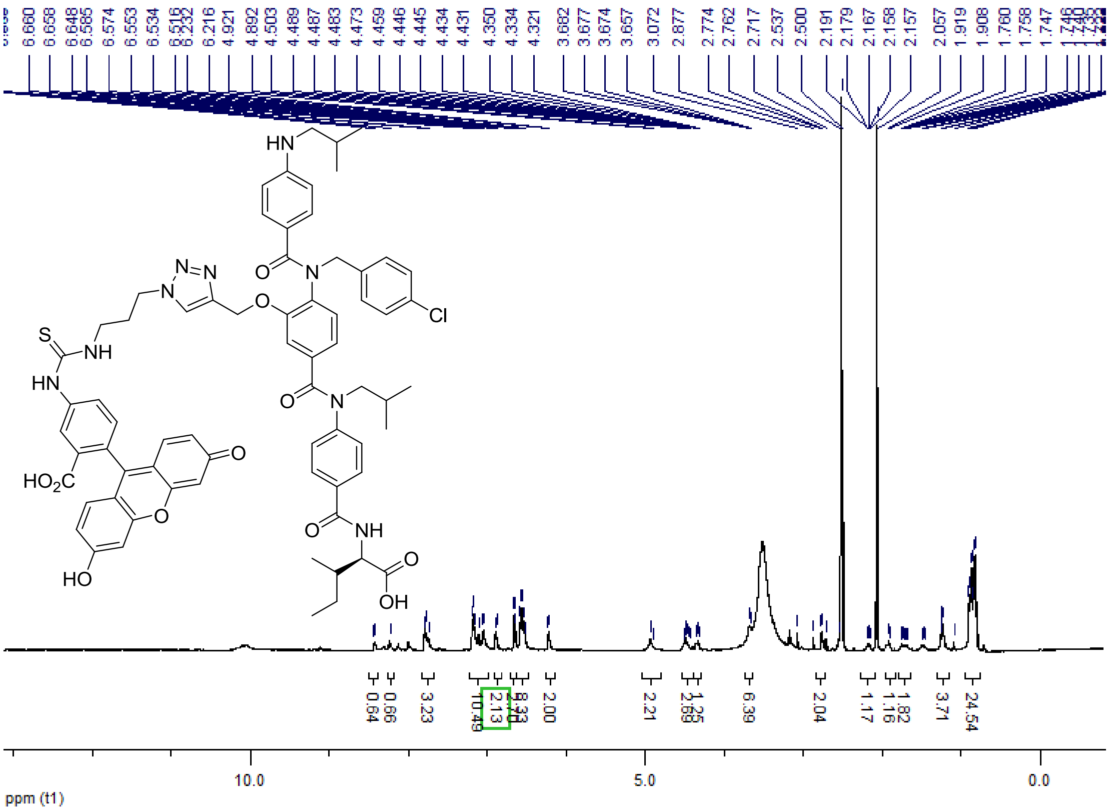

LC-MS

LC-MS of Monomer b

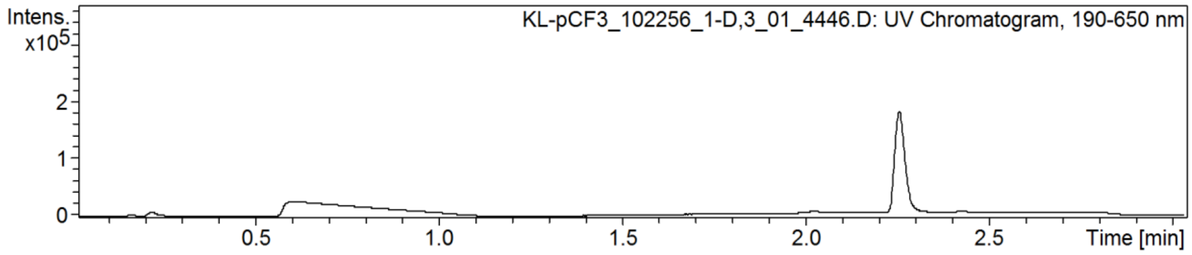

LC-MS of Monomer d

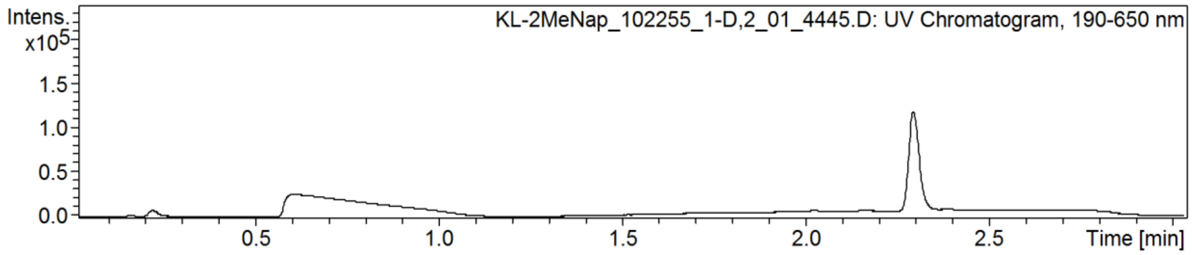

LC-MS of Monomer i

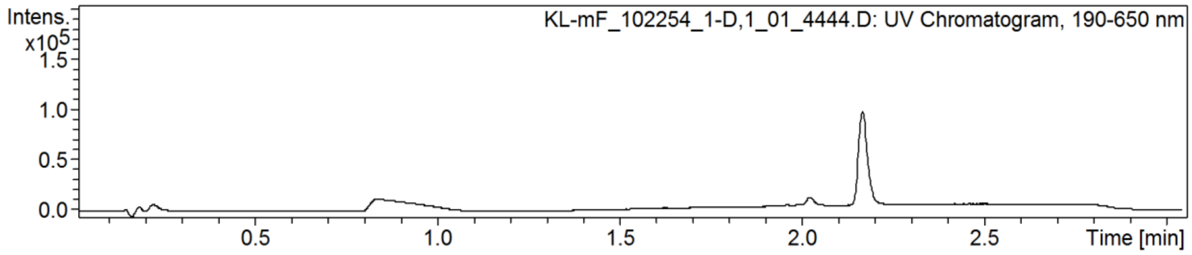

LC-MS of Monomer p

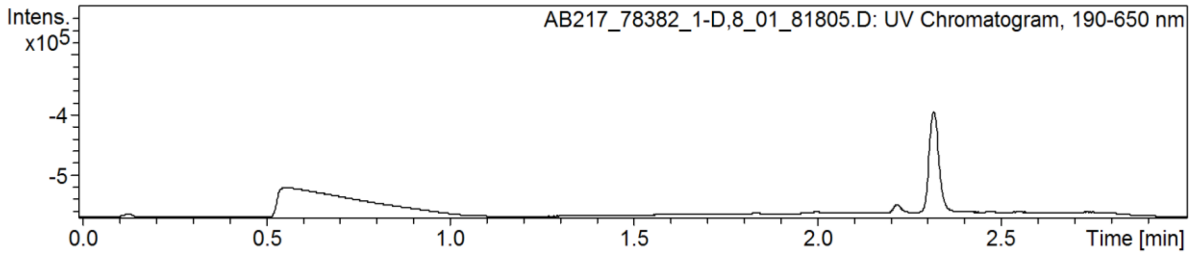

LS-MS of Monomer v

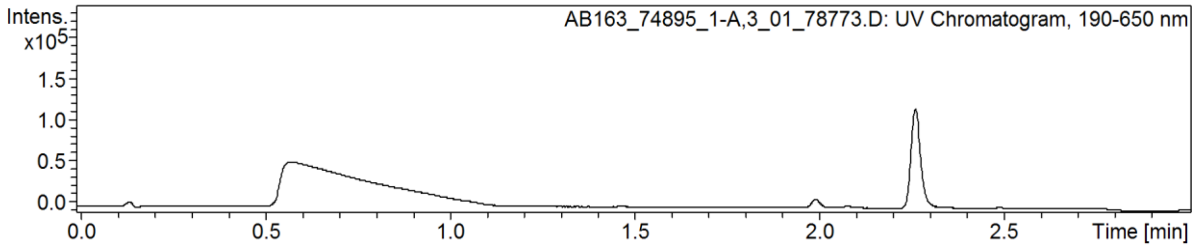

LS-MS of Monomer w

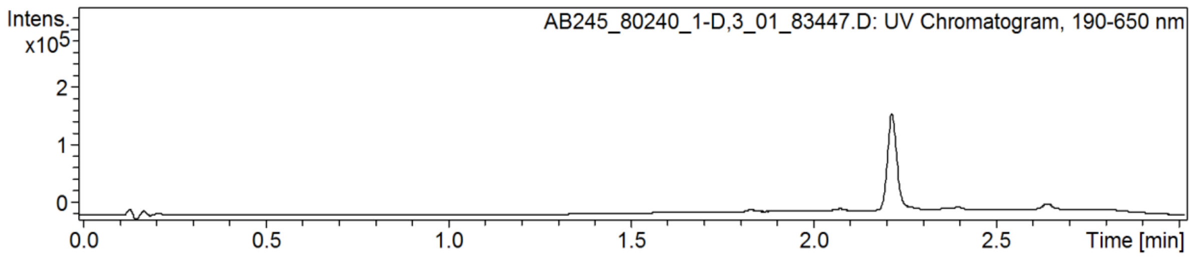

LC-MS of Compound 1

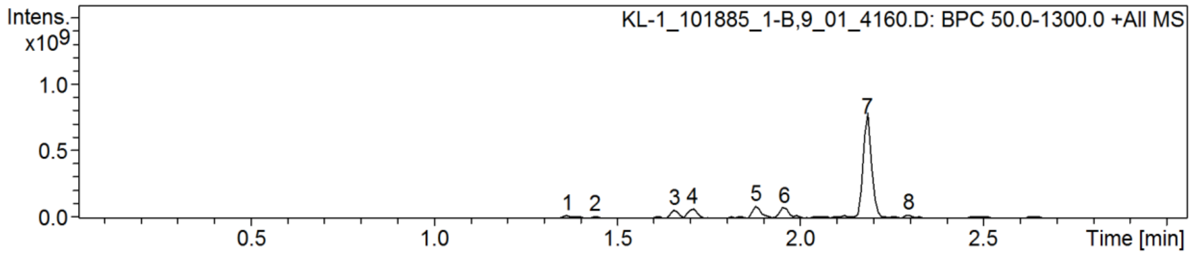

LC-MS of Compound 2

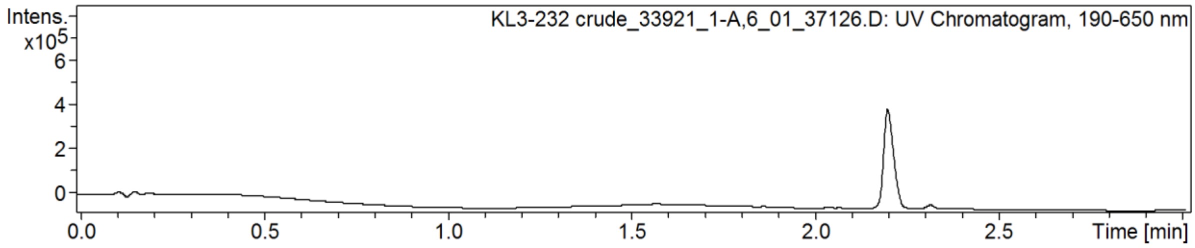

LC-MS of Compound 3

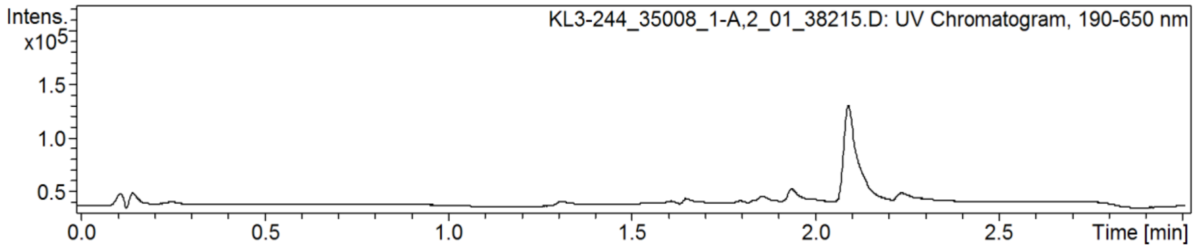

LC-MS of Compound 4

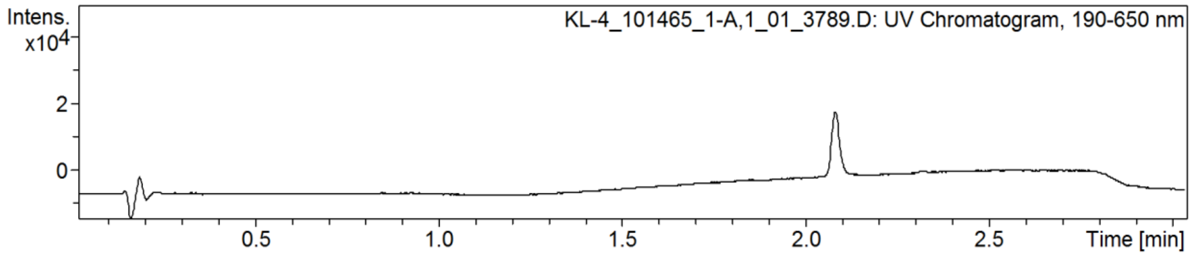

LC-MS of Compound 5

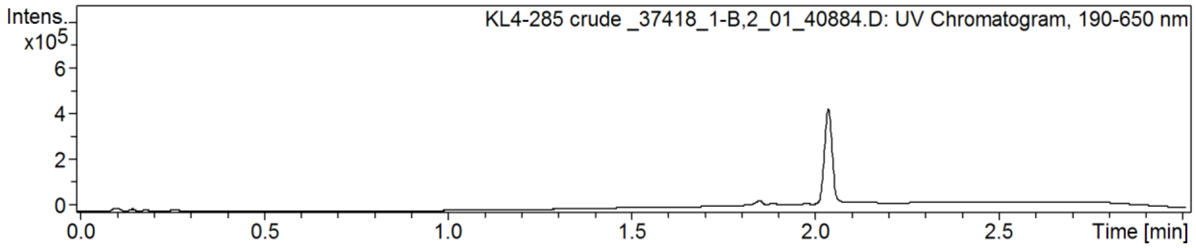

LC-MS of Compound 7

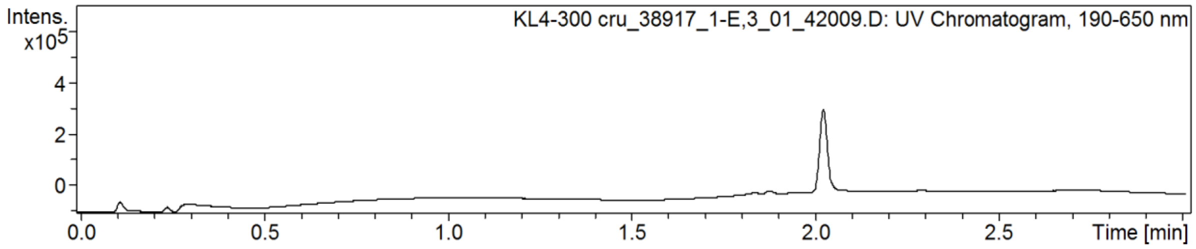

LC-MS of Compound 8

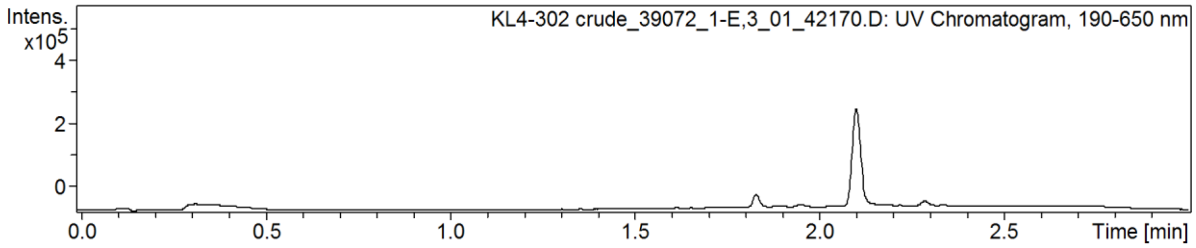

LC-MS of Compound 9

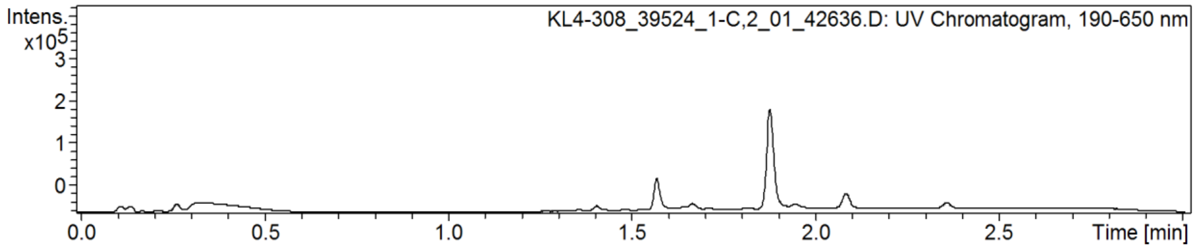

LC-MS of Compound 10

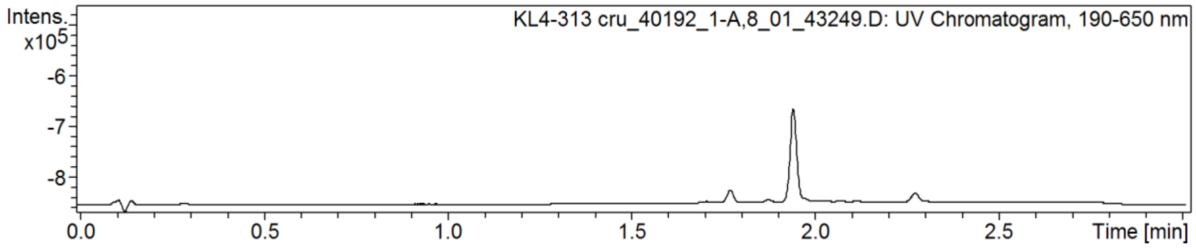

LC-MS of Compound 11

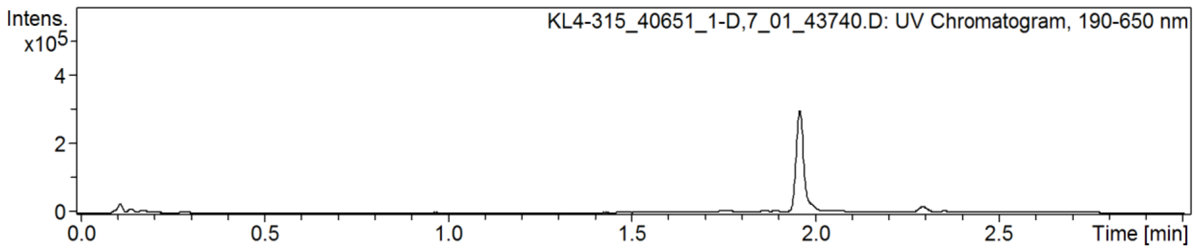

LC-MS of Compound 12

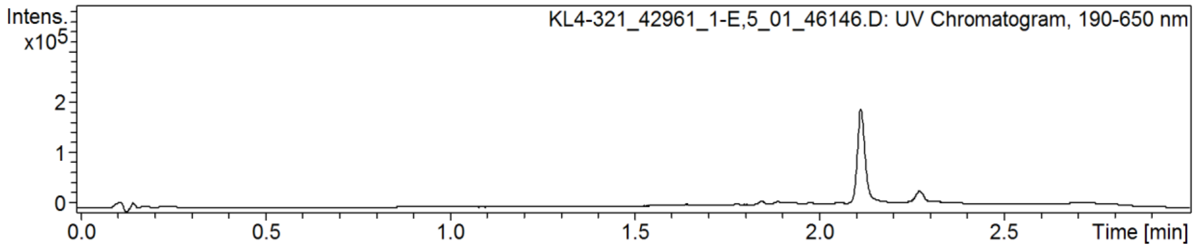

LC-MS of Compound 13

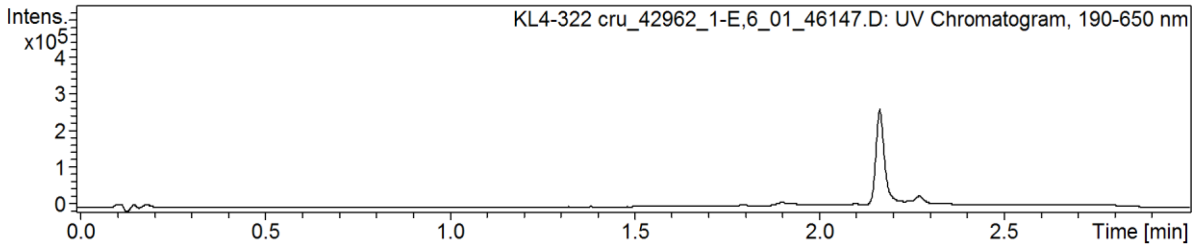

LC-MS of Compound 14

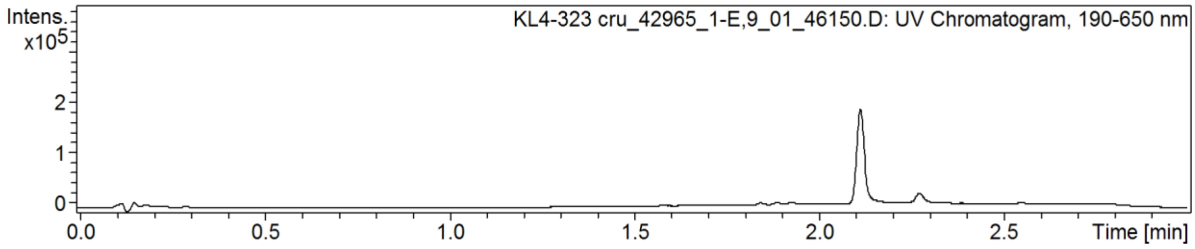

LC-MS of Compound 15

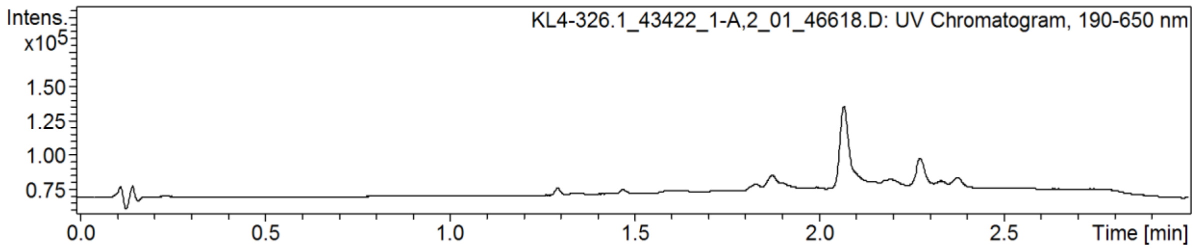

LC-MS of Compound 16

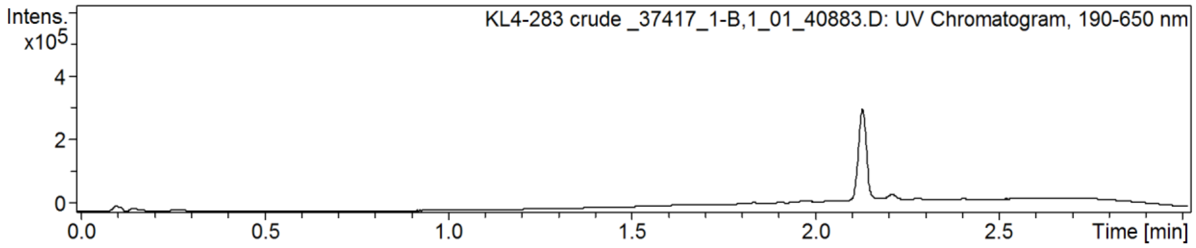

LC-MS of Compound 18

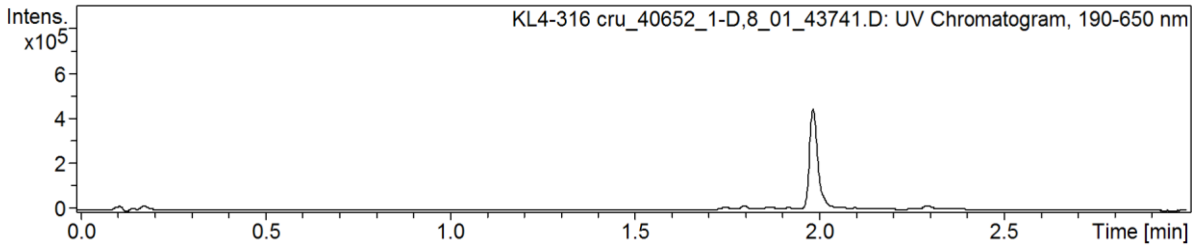

LC-MS of Compound 20

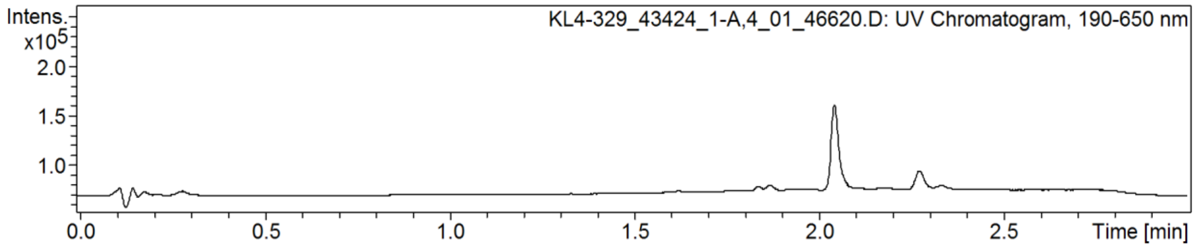

LC-MS of Compound 21

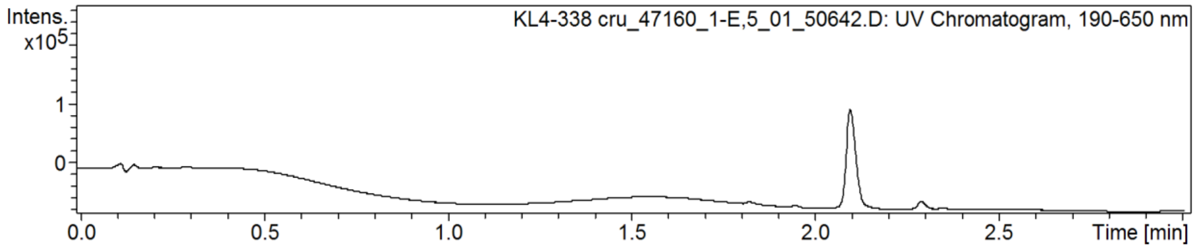

LC-MS of Compound 22

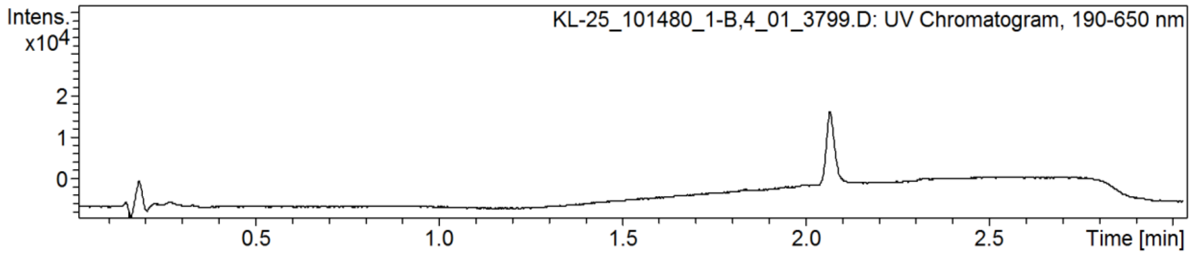

LC-MS of Compound 23

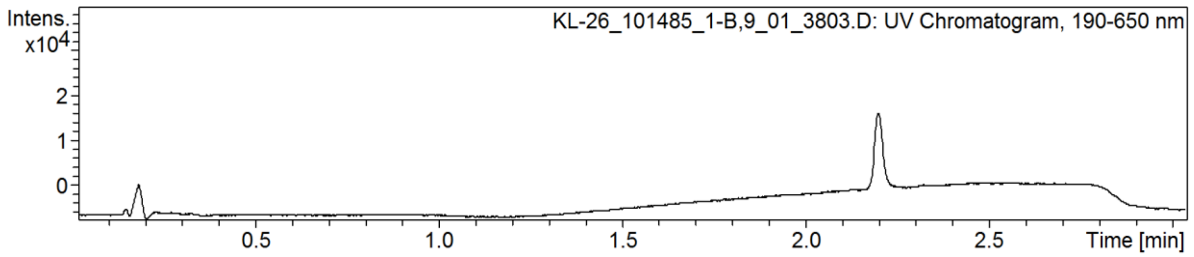

LC-MS of Compound 24

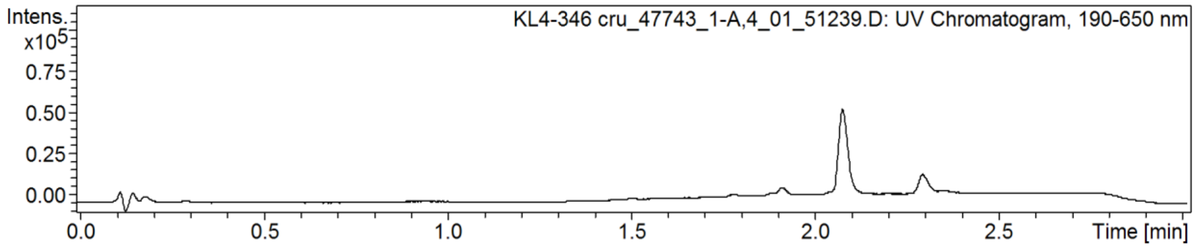

LC-MS of Compound 27

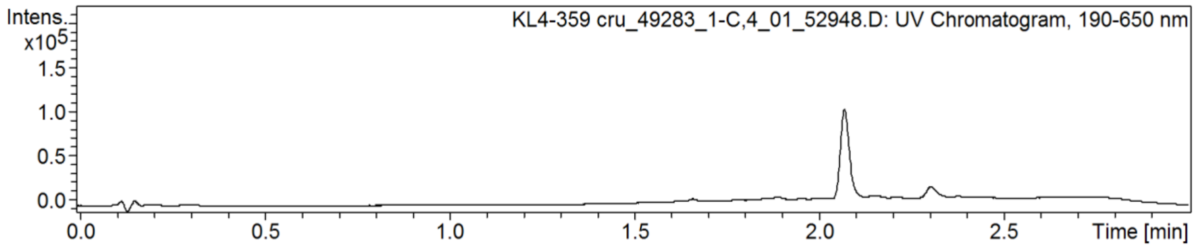

LC-MS of Compound 28

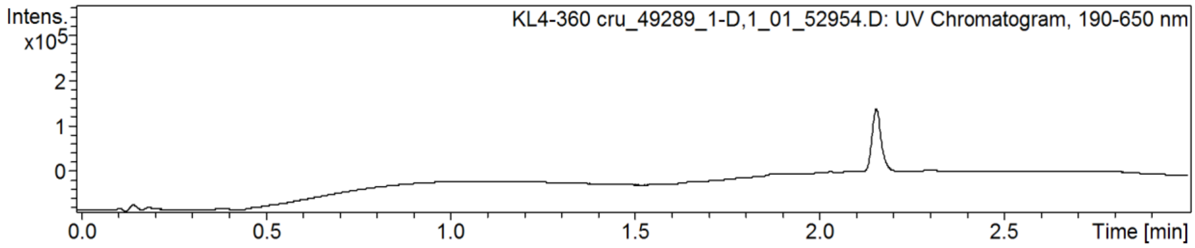

LC-MS of Compound 29

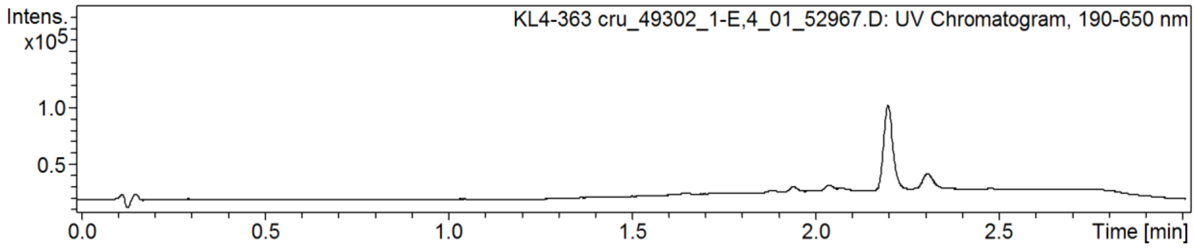

LC-MS of Compound 30

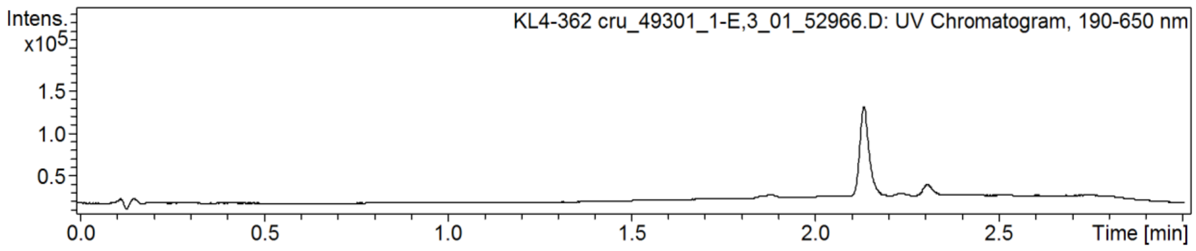

LC-MS of Compound 31

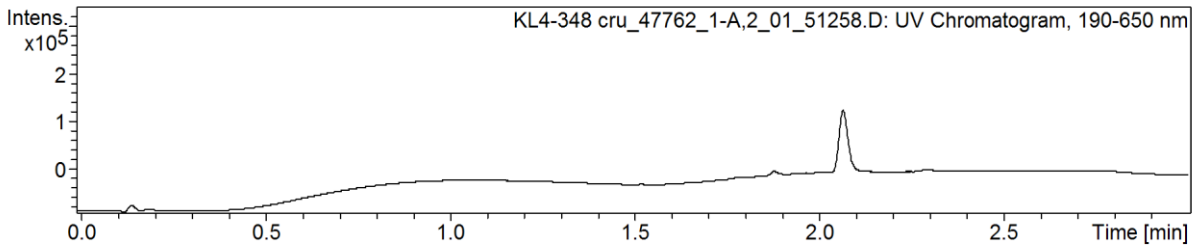

LC-MS of Compound 32

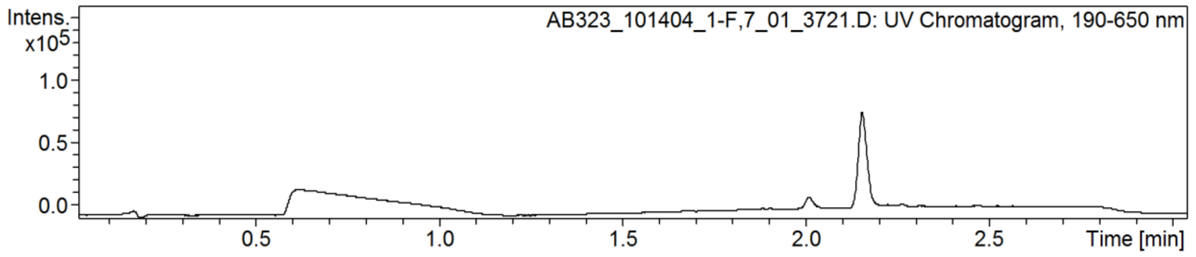

LC-MS of Compound 33

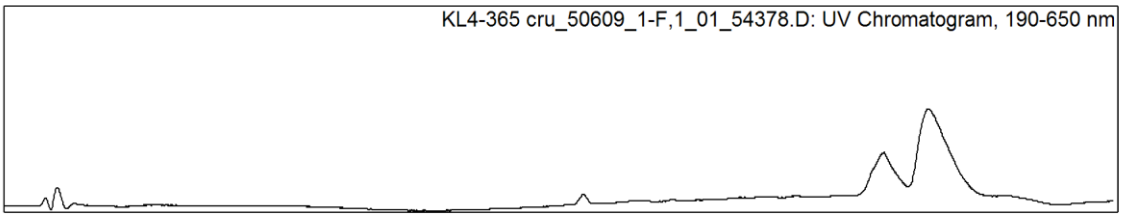

LC-MS of Compound 34

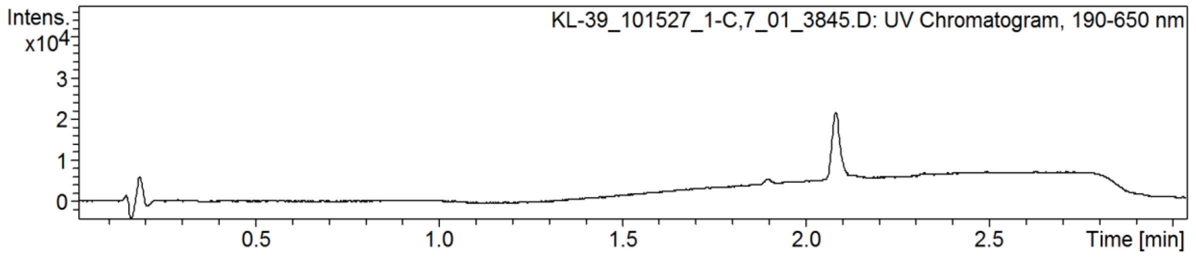

LC-MS of Compound 35

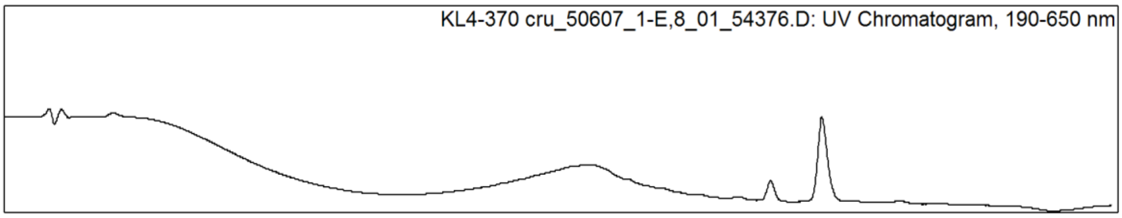

LC-MS of Compound 36

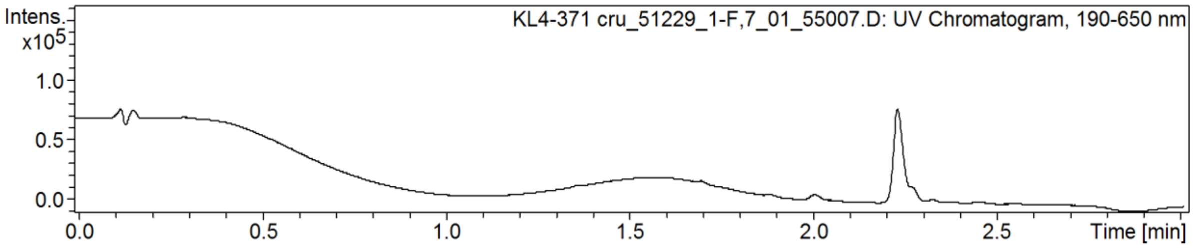

LC-MS of Compound 37

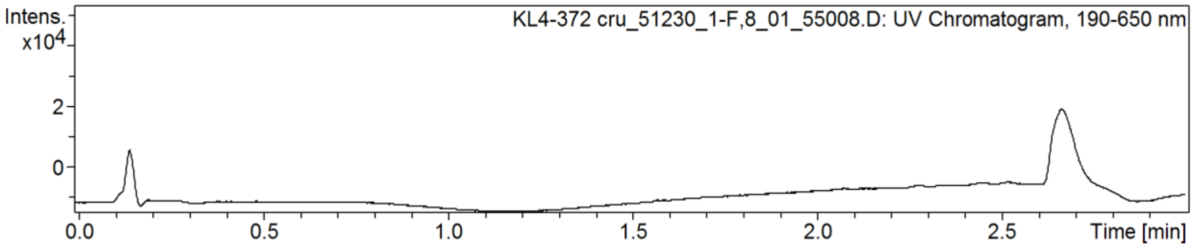

LC-MS of Compound 39

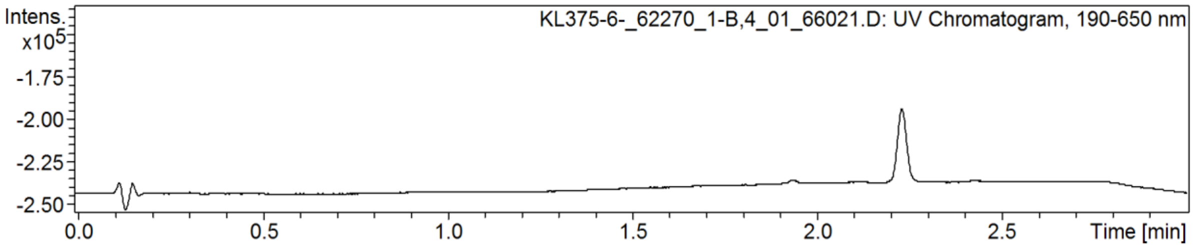

LC-MS of Compound 40

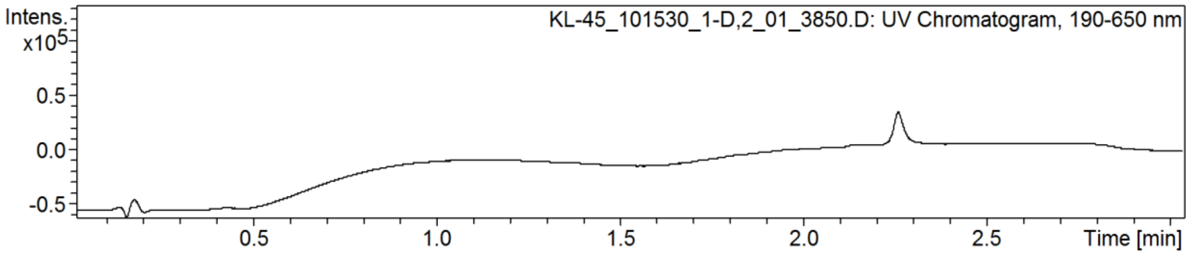

LC-MS of Compound 41

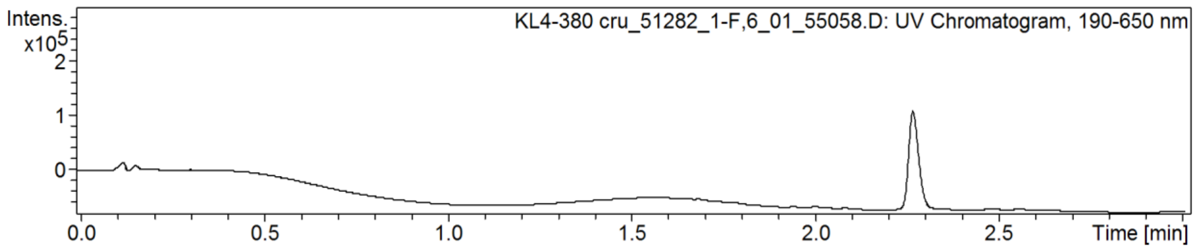

LC-MS of Compound 42

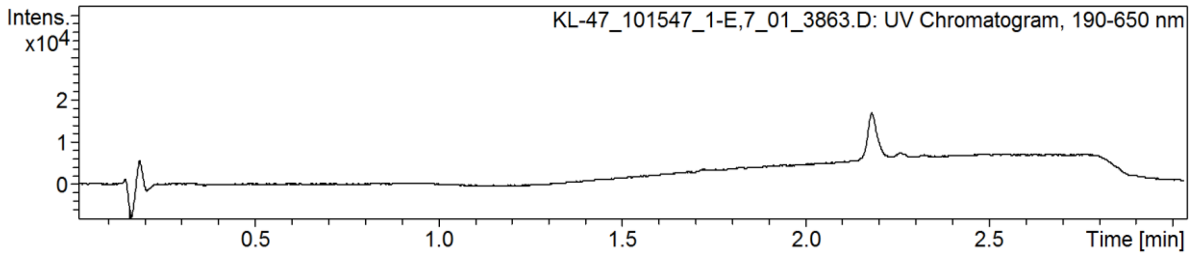

LC-MS of Compound 43

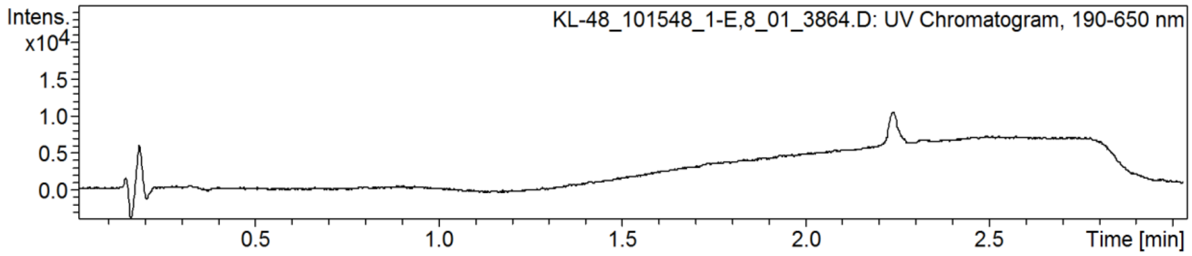

LC-MS of Compound 45

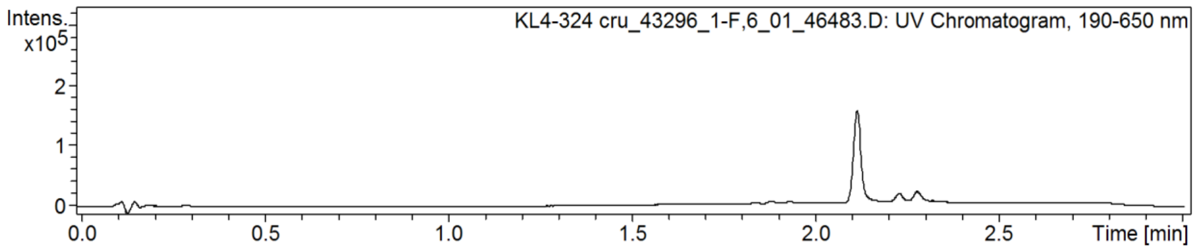

LC-MS of Compound 46

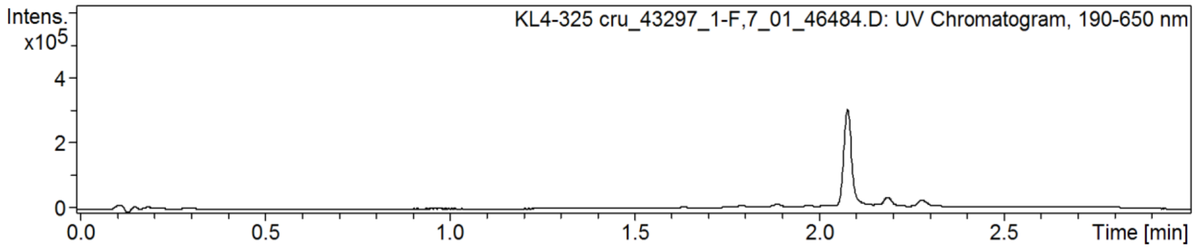

LC-MS of Compound 47

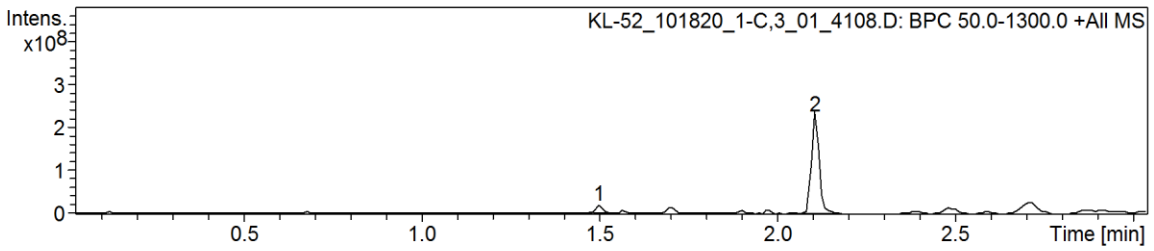

LC-MS of Compound 48

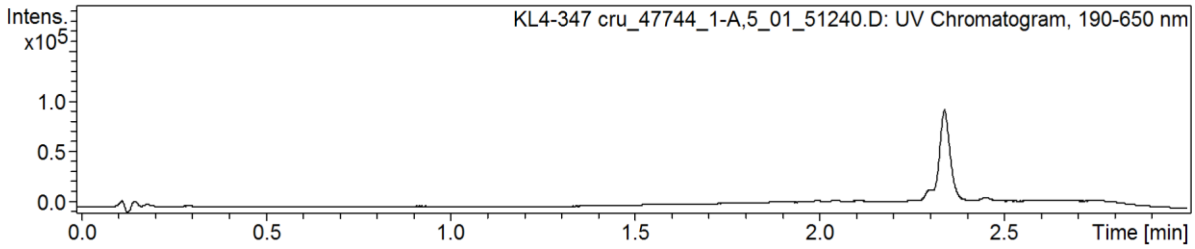

LC-MS of Compound

49

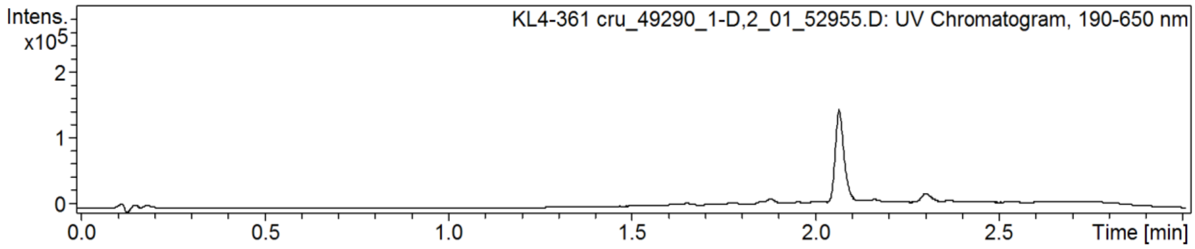

LC-MS of Compound 50

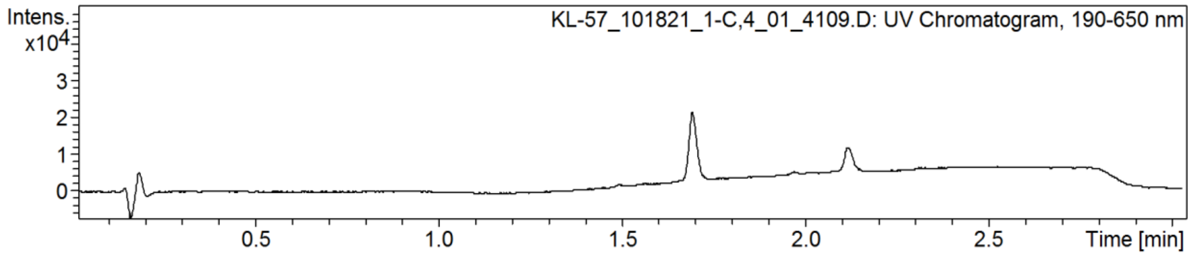

LC-MS of Compound 51

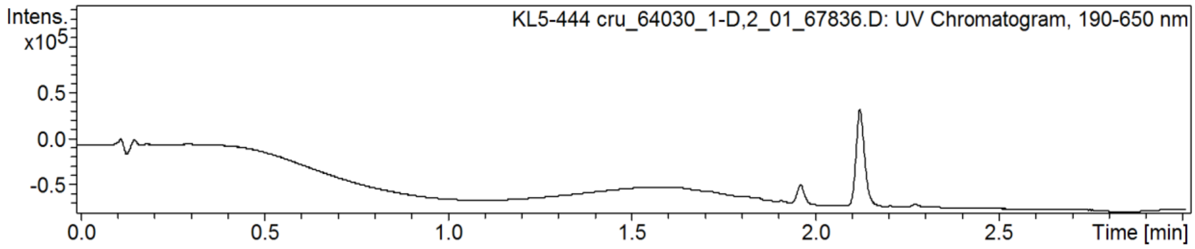

LC-MS of Compound 52

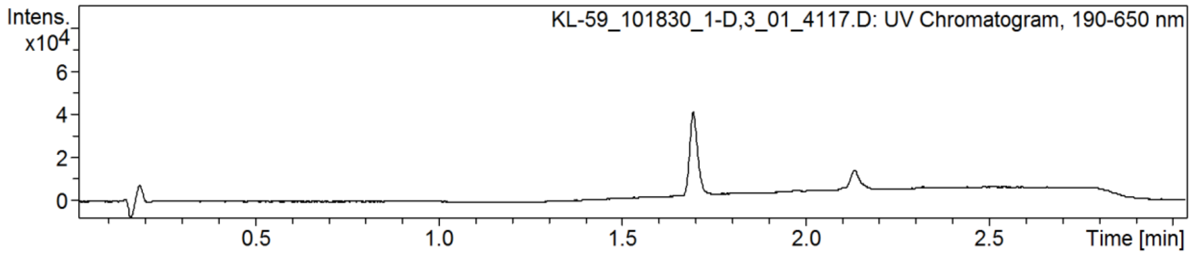

LC-MS of Compound 53

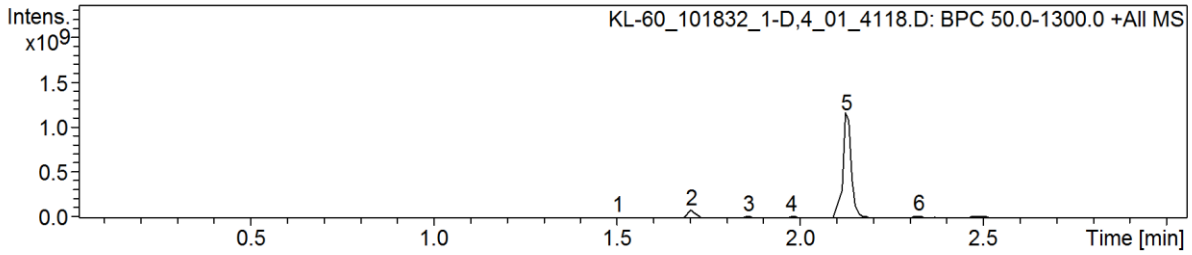

LC-MS of Compound 54

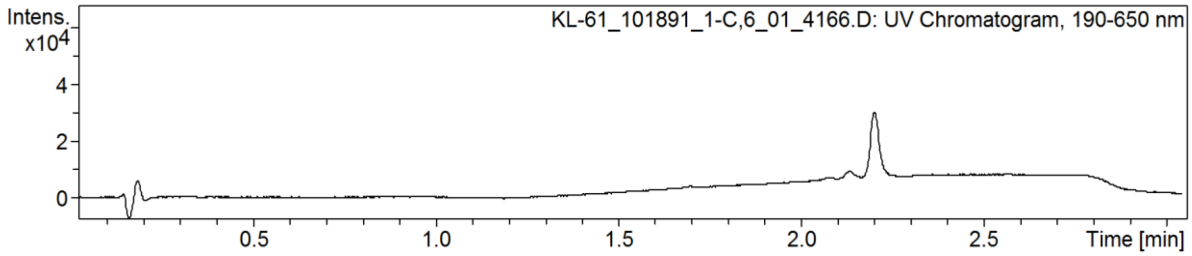

LC-MS of Compound 55

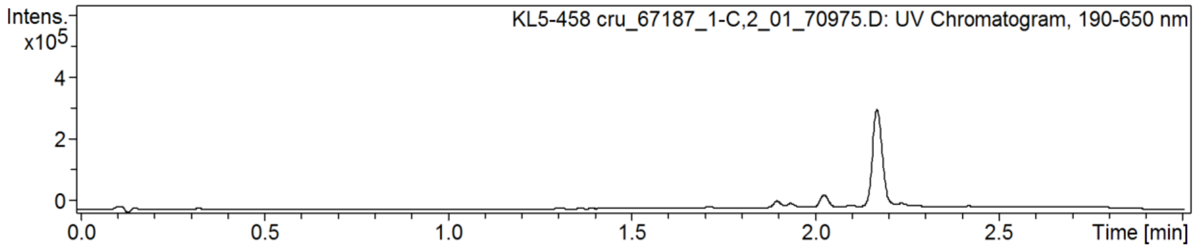

LC-MS of Compound 58

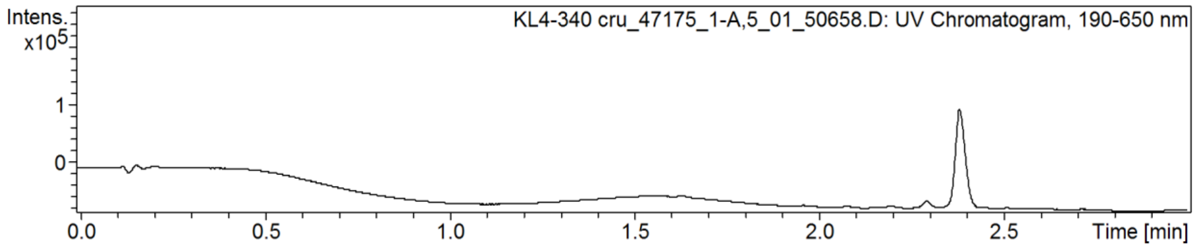

LC-MS of Compound 59

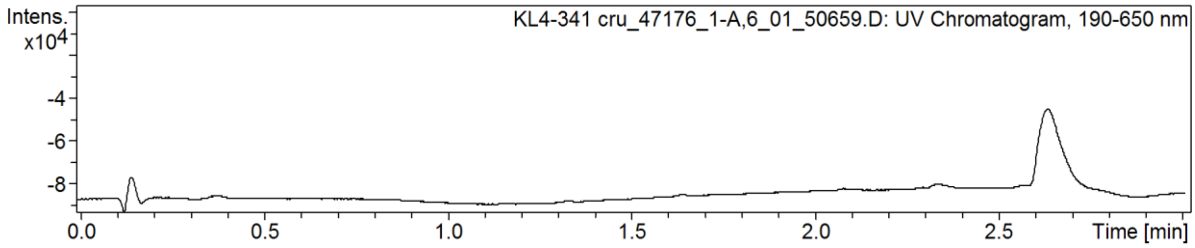

LC-MS of Compound 60

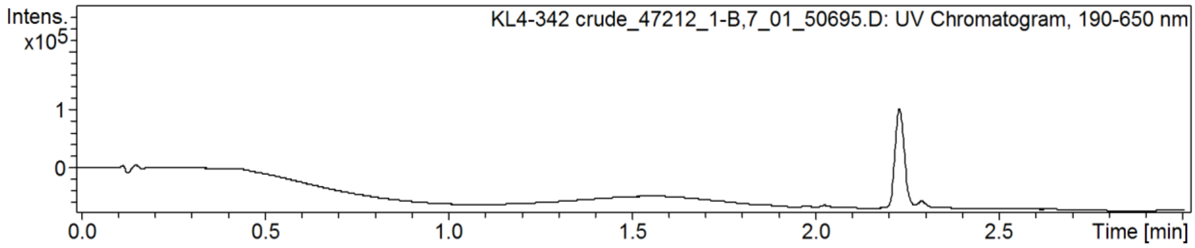

LC-MS of Compound 61

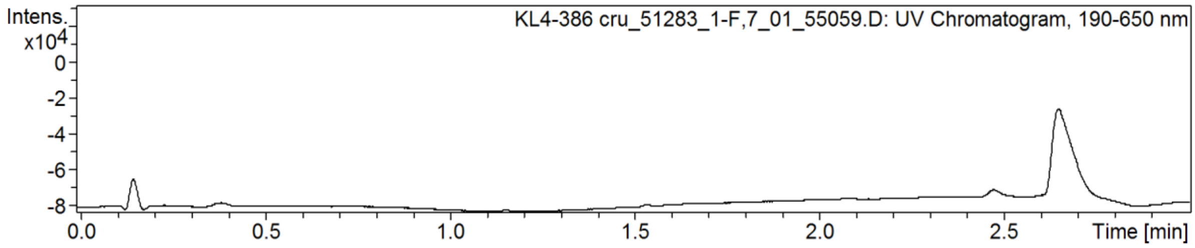

LC-MS of Compound 62

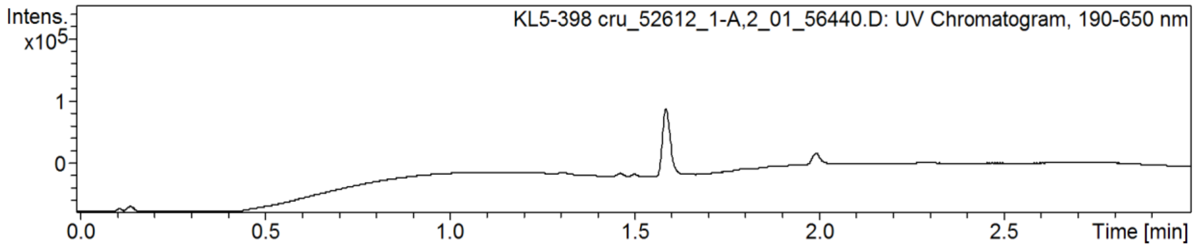

LC-MS of Compound 63

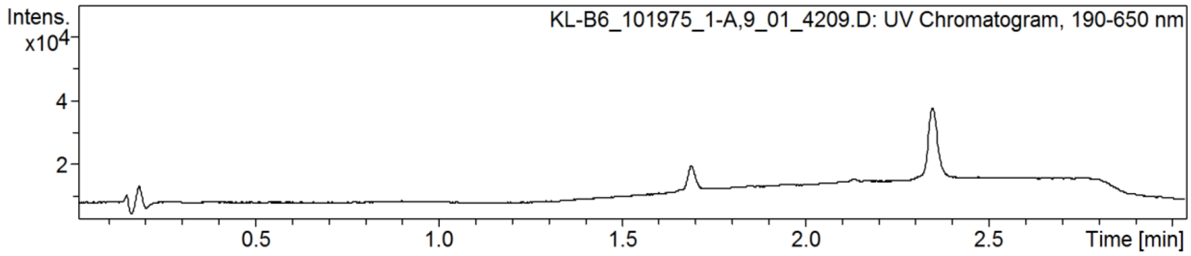

LC-MS of Compound 64

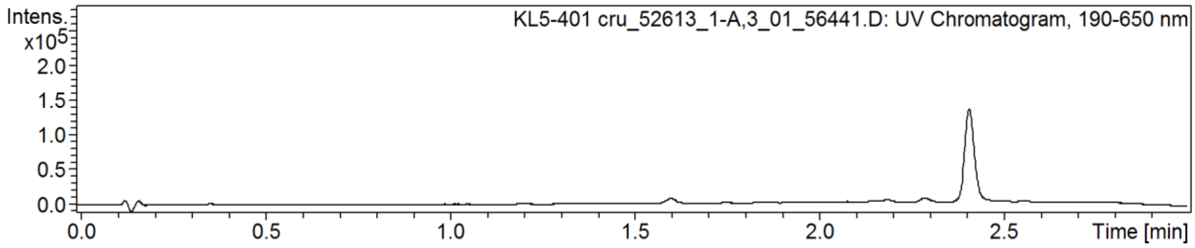

LC-MS of Compound 65

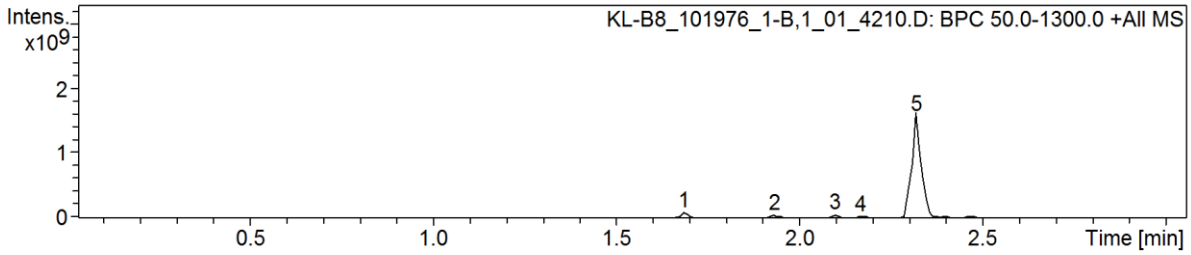

LC-MS of Compound 66

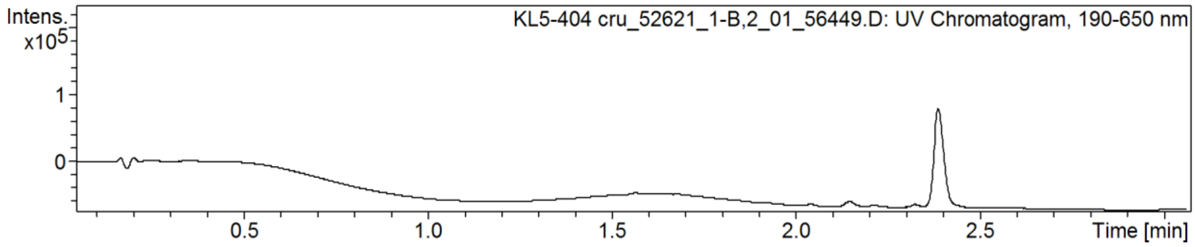

LC-MS of Compound 67

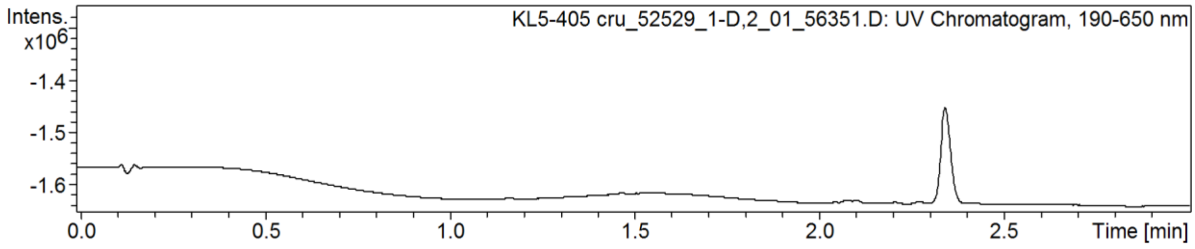

LC-MS of Compound 68

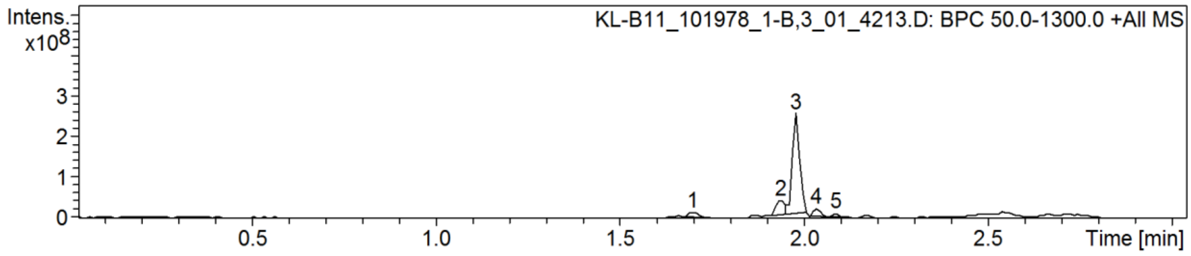

LC-MS of Compound 69

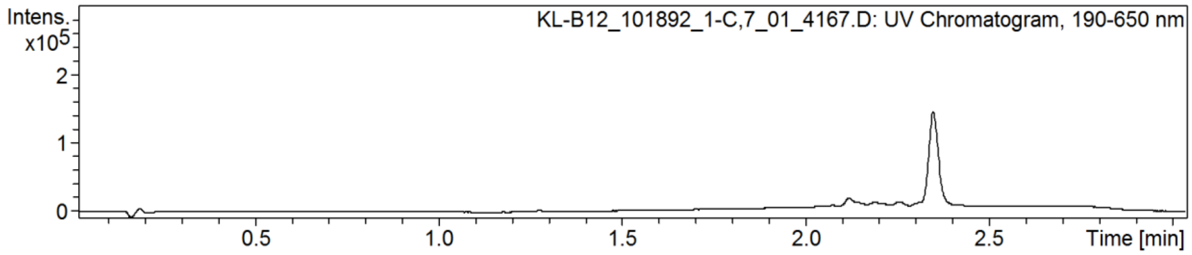

LC-MS of Compound 70

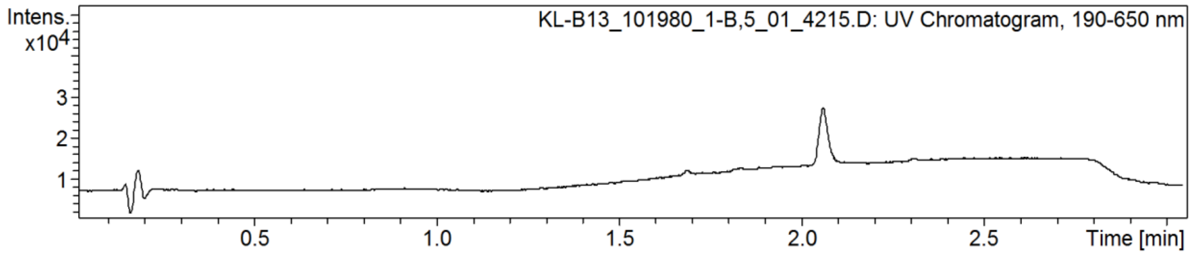

LC-MS of Compound 71

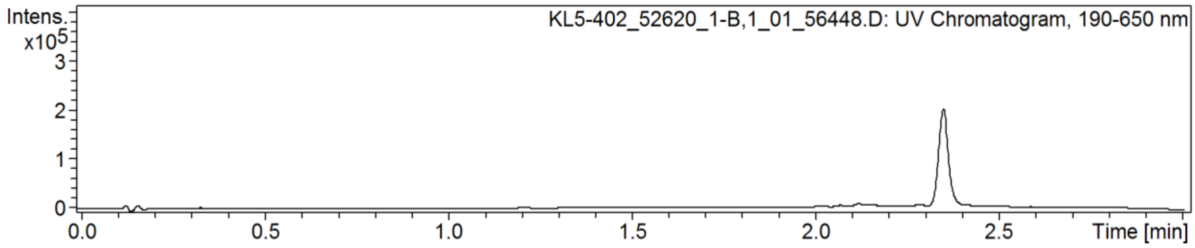

LC-MS of Compound 73

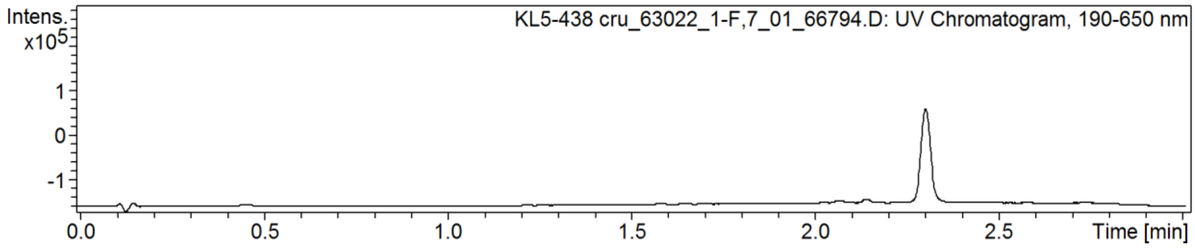

LC-MS of Compound 74

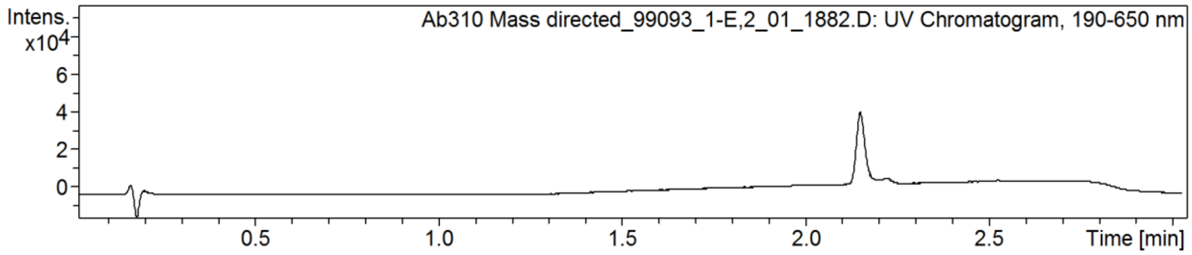

LC-MS of Compound 75

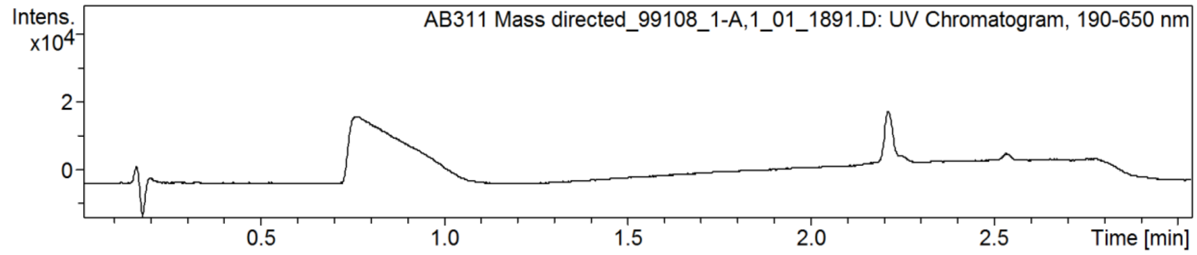

LC-MS of Compound 76

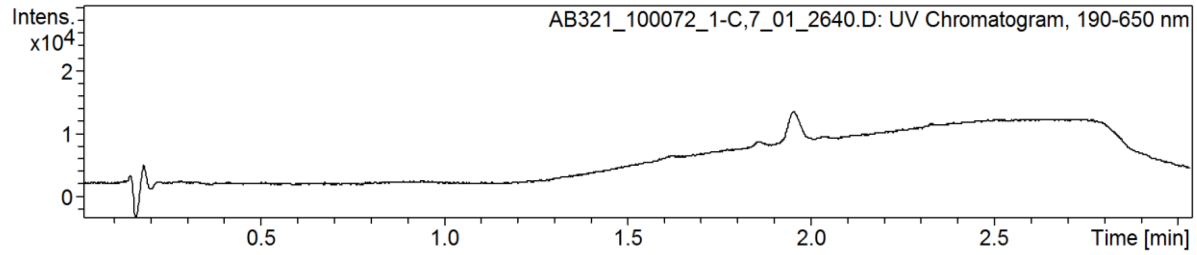

LC-MS of Compound 77

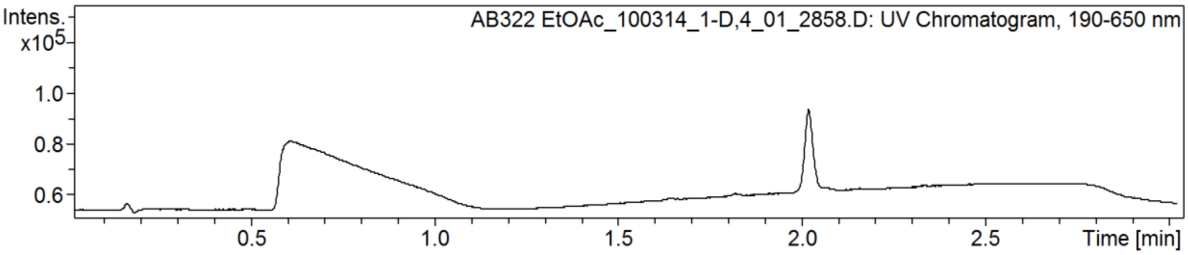

Modified Trimers

LC-MS of 1-Biotin-3-azidopropylamine

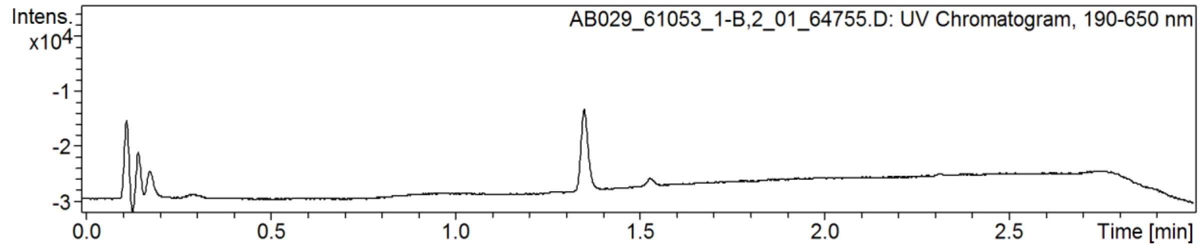

LC-MS of Biotin-8

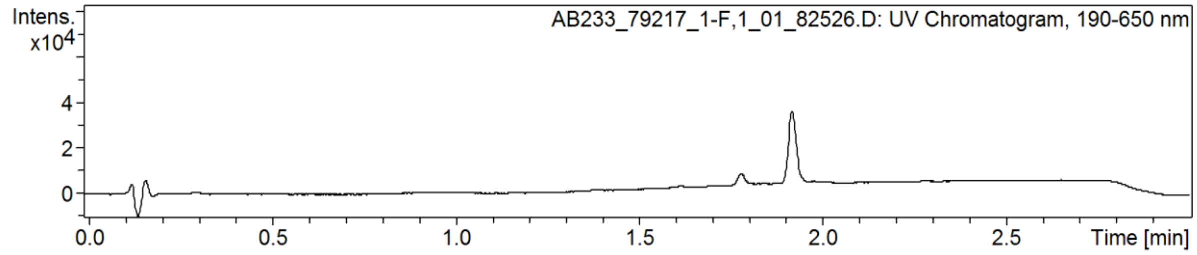

LS-MS of Biotin-31

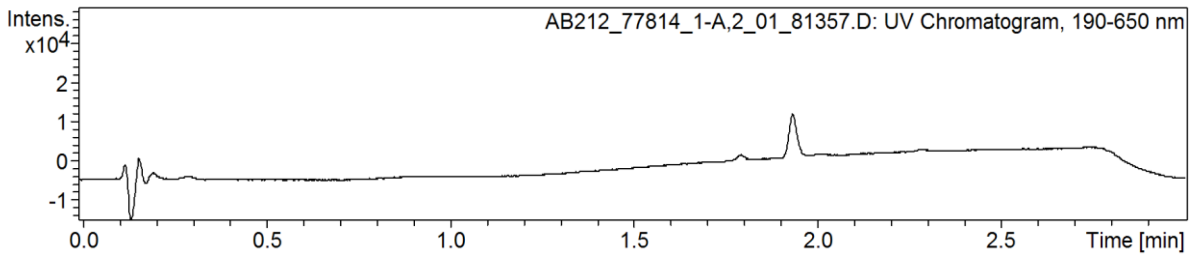

LC-MS of Biotin-48

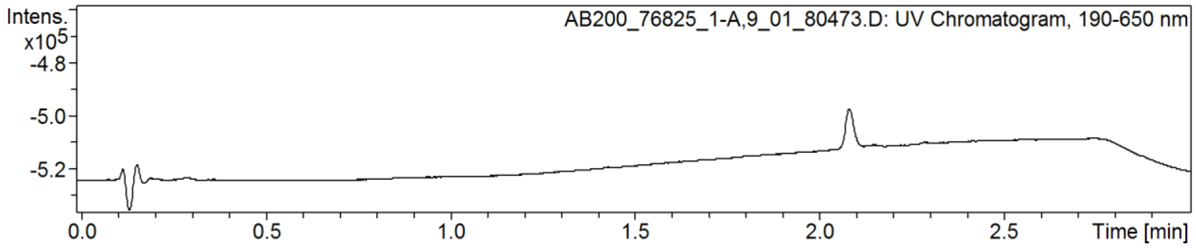

LC-MS of Biotin-64

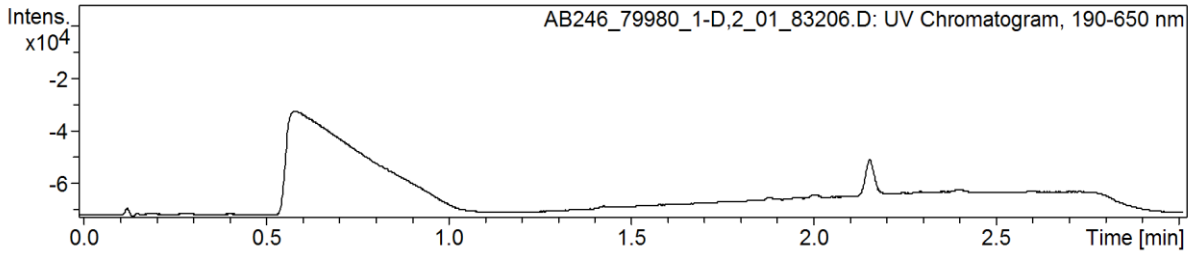

LC-MS of Biotin-67

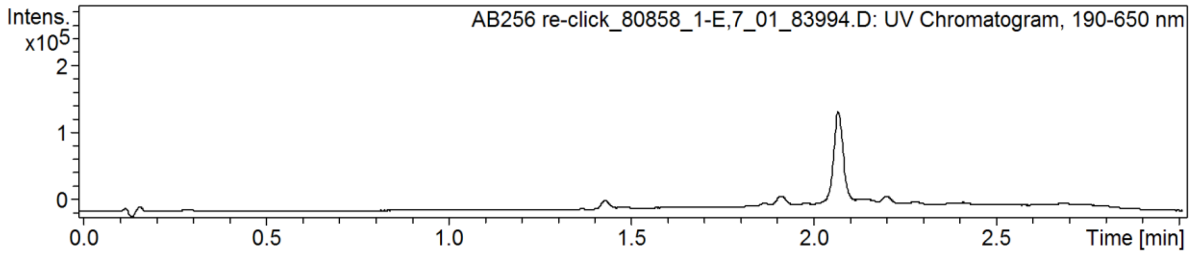

LC-MS of FITC-31

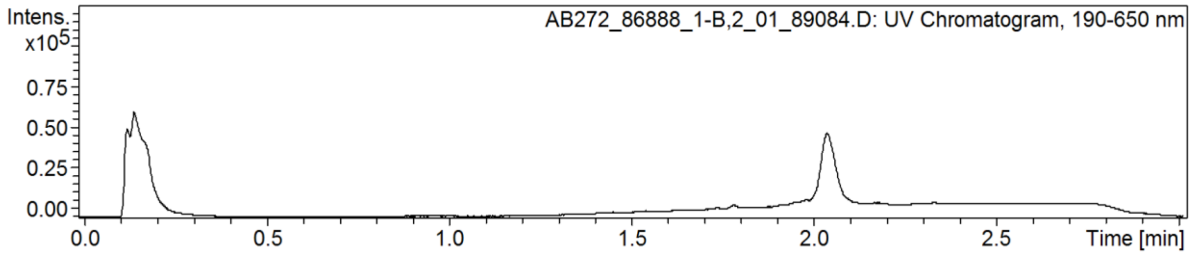

LC-MS of FITC-48

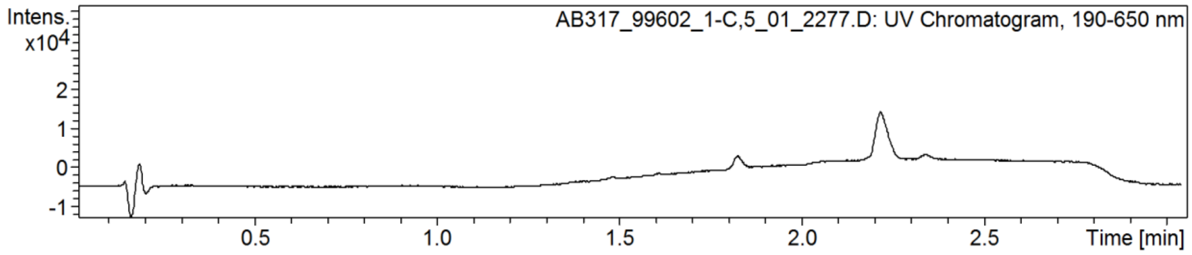

LC-MS of FITC-64

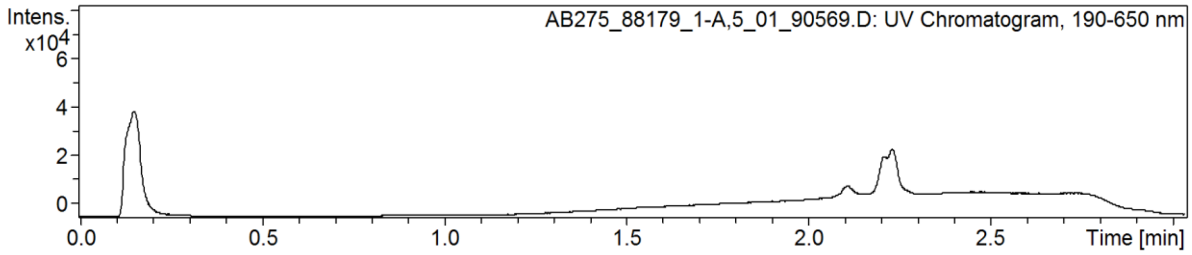

LC-MS of FITC-67

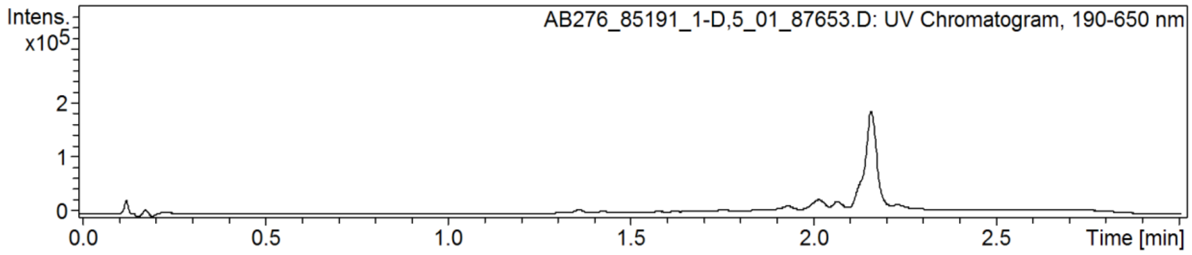

References

[1] K. Long, T. A. Edwards, A. J. Wilson, *Bioorg. Med. Chem.* **2013**, *21*, 4034.

[2] V. Azzarito, P. Prabhakaran, A. I. Bartlett, N. S. Murphy, M. J. Hardie, C. A. Kilner, T. A. Edwards, S. L. Warriner, A. J. Wilson, *Org. Biomol. Chem.* **2012**, *10*, 6469.

[3] D. J. Yeo, S. L. Warriner, A. J. Wilson, *Chem. Commun.* **2013**, *49*, 9131.

[4] A. Barnard, K. Long, D. J. Yeo, J. M. Miles, V. Azzarito, G. M. Burslem, P. Prabhakaran, T. A. Edwards, A. J. Wilson, *Org. Biomol. Chem.* **2014**.

[5] J. P. Plante, T. Burnley, B. Malkova, M. E. Webb, S. L. Warriner, T. A. Edwards, A. J. Wilson, *Chem. Commun.* **2009**, 5091.

[6] F. Campbell, J. P. Plante, T. A. Edwards, S. L. Warriner, A. J. Wilson, *Org. Biomol. Chem.* **2010**, *8*, 2344.

[7] M.-A. Bray, A. Carpenter, in *Assay Guidance Manual*, **2012**.

[8] J.-H. Zhang, T. D. Y. Chung, K. R. Oldenburg, *J. Biomol. Screen.* **1999**, *4*, 67.
